# Synthesis and Conformational Analysis of Naphthoxazine-Fused Phenanthrene Derivatives

**DOI:** 10.3390/molecules25112524

**Published:** 2020-05-28

**Authors:** Khadija Belasri, Leila Topal, Matthias Heydenreich, Andreas Koch, Erich Kleinpeter, Ferenc Fülöp, István Szatmári

**Affiliations:** 1 Institute of Pharmaceutical Chemistry and MTA-SZTE Stereochemistry Research Group, Hungarian Academy of Sciences, University of Szeged, Eötvös u. 6, H-6720 Szeged, Hungary; belasrikhadija15@gmail.com (K.B.); topal.leila@gmail.com (L.T.); fulop@pharm.u-szeged.hu (F.F.); 2 Institute of Pharmaceutical Chemistry, University of Szeged, Interdisciplinary excellence center, H-6720 Szeged, Hungary; 3 Department of Chemistry, University of Potsdam, Karl-Liebknecht-Str. 24-25, D-14476 Potsdam (Golm), Germany; mheydenr@uni-potsdam.de (M.H.); kochi@uni-potsdam.de (A.K.); ekleinp@uni-potsdam.de (E.K.)

**Keywords:** modified Mannich reaction, cyclic imines, [4 + 2] cycloaddition, NMR spectroscopy, conformational analysis, DFT calculations

## Abstract

The synthesis of new phenanthr[9,10-*e*][1,3]oxazines was achieved by the direct coupling of 9-phenanthrol with cyclic imines in the modified *aza*-Friedel–Crafts reaction followed by the ring closure of the resulting bifunctional aminophenanthrols with formaldehyde. Aminophenanthrol-type Mannich bases were synthesised and transformed to phenanthr[9,10-*e*][1,3]oxazines via [4 + 2] cycloaddition. Detailed NMR structural analyses of the new polyheterocycles as well as conformational studies including Density Functional Theory (DFT) modelling were performed. The relative stability of *ortho*-quinone methides (*o*-QMs) was calculated, the geometries obtained were compared with the experimentally determined NMR structures, and thereby, the regioselectivity of the reactions has been assigned.

## 1. Introduction

It is known that 9-phenanthrol is one of the most attractive structural units present in a large number of biologically active compounds. It is the identified inhibitor of the transient receptor potential melastatin (TRPM) 4 channels, a Ca^2+^ activated non-selective cation channel whose mechanism of action remains to be determined [1,2,3,4]. Subsequent studies have proven that it modulates smooth muscle contraction in bladder and cerebral arteries, affects spontaneous activity in neurons and in the heart, and reduces lipopolysaccharide-induced cell death [5,6,7].

The Mannich reaction is one of the most important reactions in organic synthesis to form C–C bonds [8,9,10]. This synthetic pathway is widely used in the formation of secondary and tertiary amine derivatives and it is a key step in the synthesis of numerous bioactive molecules and complex natural products [11,12]. In one of the special variations of the modified Mannich reaction (*m*MR), 1- and 2-naphthol were applied as electron-rich aromatic compounds [13,14]. Mechanistically, the modified *aza*-Friedel–Crafts reaction can be interpreted as a special *m*MR, where electron-rich aromatic compounds such as 1- and 2-naphthol and their *N*-containing analogues are reacted with a wide range of cyclic imines to furnish aminonaphthols [15,16], aminoquinolinols, or aminoisoquinolinols [17,18,19]. Accordingly, our first aim was to examine the reactivity of 9-phenanthrol with different cyclic imines in the modified *aza*-Friedel–Crafts reaction.

Recent publications pointed out that *ortho*-quinone methides (*o*-QMs) can also be generated from Mannich bases after thermal elimination of the amino group [20]. The reactive moiety thus formed can be stabilized by reactions with different dienophiles [14,21,22] or it can participate in [4 + 2] cycloadditions with cyclic imines to form new heterocycles [18,23,24]. Very recently the transformation of functionalised 1-aminobenzyl-2-naphthols via [4 + 2] cycloaddition has been examined [25]. It was found that the regio- and diastereoselectivity of the cycloaddition depend on the functional group of the phenyl substituent. Based on these findings, our aim was to synthesise aminobenzylphenanthrols or functionalised aminobenzylphenanthrols and to study their reactivity with cyclic imines as a novel precursor Mannich bases in [4 + 2] cycloaddition. Finally, we wanted to explore both the structure and conformational behaviour of the novel polyheterocycles by NMR spectroscopy and accompanying theoretical quantum chemical (QC) calculations.

## 2. Results

### 2.1. Synthesis

**Scheme 1:**
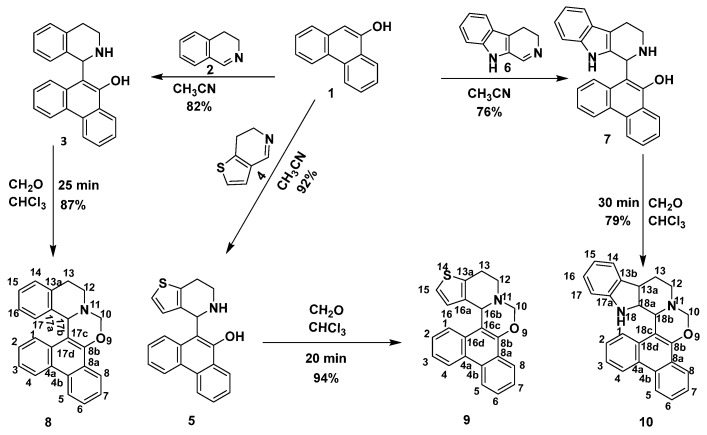
Syntheses and ring closures of bifunctional compounds **3**, **5** and **7.**

To examine the possibility of the extension of 9-phenanthrol (**1**) in the modified *aza*-Friedel–Crafts reaction, **1** was reacted with 1.5 equiv. of 3,4-dihydroisoquinoline (**2**) [26] (Scheme 1) under neat conditions at 80 °C. After a reaction time of 120 min, the desired product (**3**) was isolated only with a yield of 10%. By increasing the temperature to 100 °C the yield of **3** increased slightly to 19%. Yields found under these conditions were not satisfactory and the appearance of side products was also observed. By changing the solvent to acetonitrile, treatment at reflux temperature (90 °C) afforded **3** in an isolated yield of 49%. The product was easily separated out from the reaction mixture that is acetonitrile proved to be an optimal solvent. To further improve the yield, the reaction was repeated under microwave (MW) conditions. In this case the reaction was driven at 100 °C and after a short reaction time (10 min) **3** was isolated in a yield of 67%, that could be improved to 82% by increasing the reaction time to 20 min (Table 1). It should be noted that the optimal workup procedure remained the filtration of the formed product from the cold reaction mixture. Subsequently, with the satisfactory optimal reaction conditions in hand, the extension possibility of the reaction was tested by using various cyclic imines, including 6,7-dihydrothieno[3,2-*c*]pyridine (**4**) [27] or 3,4-dihydro-β-carboline (**6**) [28]. These reactions afforded new 1-(9-phenanthrol-10-yl)-thieonopyridine **5** and 1-(9-phenanthrol-10-yl)-β-carboline **7** in yields of 92% and 76%, respectively (Table 1).

In the next step, the ring closures of aminophenanthrols **3**, **5**, and **7** were performed with 35% aqueous formaldehyde in CHCl_3_ at room temperature. Reactions completed in relatively short time and phenanthrooxazines **8**–**10** were isolated in excellent yields by simple crystallization from *n*-hexane.

**Scheme 2:**
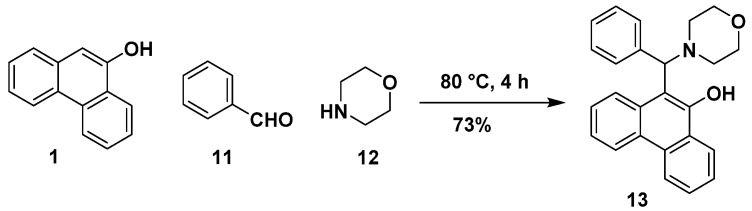
Synthesis of 10-morpholinobenzyl-9-phenanthrol (**13**).

To test aminophenanthrols in cycloaddition reaction, first the synthesis of precursor **13** was achieved by reacting 9-phenanthrol (**1**) with morpholine in the presence of benzaldehyde. The reaction was carried out under solvent-free conditions at 80 °C. After a 4-h reaction, the desired 10-morpholinobenzyl-9-phenanthrol (**13**) was isolated by crystallization with *n*-hexane (Scheme 2).

**Scheme 3:**
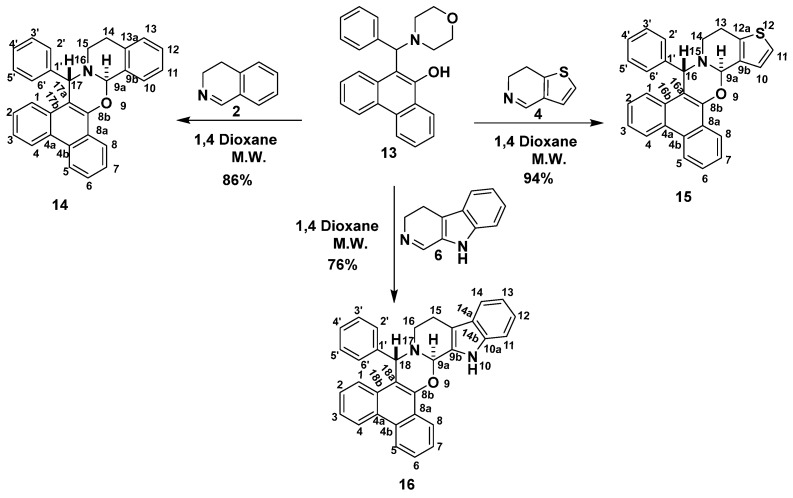
Synthesis of phenanthr[9,10-*e*]oxazine derivatives (**14**–**16**).

First, aminophenanthrol **13** was reacted with 3,4-dihydroisoquinoline **2** as dienophile. The reaction was performed in 1,4-dioxane under microwave irradiation at three different temperatures (60, 80, and 100 °C). In our first experiment, the reaction was performed at 60 °C and after a relatively short reaction time the desired product (**14**) was isolated only in a low yield (47%). Since the yield was not satisfactory, the reaction was repeated at 80 and 100 °C. As Table 2 shows, 80 °C and 15 min reaction time were found to be the optimal reaction conditions. The series of dienophiles was extended by using 6,7-dihydrothieno[3,2-*c*]pyridine **4** and 3,4-dihydro-β-carboline **6** (Scheme 3). The optimal conditions and related yields are listed in Table 2. Since two new stereogenic centres are generated during the reaction, two epimeric structures (**a** and **b**) can be obtained. The reaction was monitored by TLC and the compositions of the crude reaction mixtures were verified by ^1^H-NMR analysis. All reactions were found to be diastereoselective and the relative configuration of H-9a:H-17 (**14**), H-9a:H-16 (**15**) and H-9a:H-18 (**16**) proved to be *trans*, based on the detailed NMR analysis (vide infra).

**Scheme 4:**
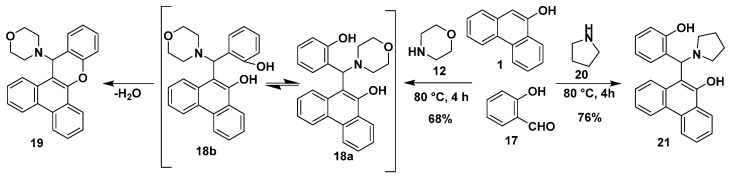
Synthesis of compounds **19** and **21**.

Next, we wanted to investigate how *o*-QMs generated from functionalised aminophenanthrol derivatives can influence the [4 + 2] cycloaddition reaction. Accordingly, 9-phenanthrol and salicylic aldehyde were reacted in the presence of morpholine. The reaction was carried out under neat conditions at 80 °C. The characteristic spot formed according to the TLC was isolated and the NMR spectra of the formed compound confirmed the structure of **19**. Formation of the dibenzo[*a*,*c*]xanthene (**19**) side-product can be explained by the elimination of water from diol 18. In this latter modified Mannich reaction, the availability of the nucleophile (morpholine) was postulated. Therefore, it was replaced by pyrrolidine applying again the above conditions (80 °C, neat). After a reaction time of 4 h, the expected phenanthrol derivative **21** was isolated in a yield of 76% (Scheme 4).

**Scheme 5:**
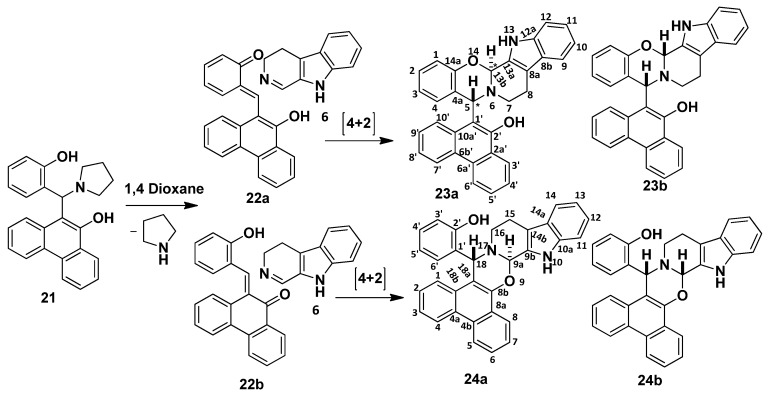
[4 + 2] Cycloaddition between **21** and 3,4-dihydro-β-carboline.

To test the scope and limitations of the [4 + 2] cycloaddition, precursor **21** was first reacted with 3,4-dihydro-β-carboline as dienophile. The reaction was performed in 1,4-dioxane at three different temperatures (60, 80, and 100 °C) under microwave irradiation (Table 3). After the thermal decomposition of starting material 21, two types of *o*-QMs (**22a** and **22b**) can be formed that can lead to the formation of two regioisomers and two diastereomers (Scheme 5) verified by ^1^H-NMR analysis of the crude reaction mixture. Then, various reaction conditions were applied as depicted in Table 3. In all cases, reactions were found to be regio- and diastereoselective. The detailed NMR analysis (vide infra) confirmed that the formed/isolated product **24a** is a phenanthroxazine and the relative configuration of H-9a:H-18 is *trans*.

**Scheme 6:**
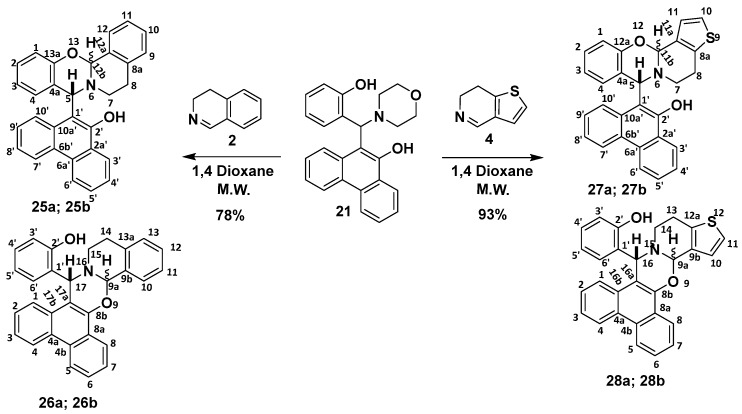
Reaction of functionalised aminophenanthrol **21** with cyclic imines.

The reaction was then extended by using 3,4-dihydroisoquinoline (**2**) and 6,7-dihydrothieno[3,2-*c*]pyridine (**4**) as cyclic imines (Scheme 6). In these cases, similar regio- and diastereoselectivity of the reactions were proved too. Detailed NMR analysis (vide infra) adequately supported that the isolated products are *trans*-phenanthroxazines (**26a**, **28a**, Scheme 6). The optimal reaction conditions together with the yields are summarised in Table 3.

### 2.2. Structural and Conformational Analysis

The complete ^1^H and ^13^C-NMR study of both diastereomeric phenanthr[9,10-*e*]oxazine derivatives **14**–**16** and regioisomeric/diastereotopic phenanthroxazine derivatives **24a**, **26a**, and **28a** showed identical stereoisomerism (only one set of NMR spectra obtained). The NMR analyses of β-carboline derivative **16** and thiophene compound **28a** are exemplarily utilised. The same stereoisomeric results were obtained for **14** and **24a,** as well as **15** and **26a**. For the corresponding structure elucidation, in addition to the NMR spectra (chemical shift, coupling constants, NOEs), quantum chemical calculations were also performed.

### 2.3. Detailed NMR Analysis of New Phenanthr[9,10-e]Oxazine ***16***

**Figure 1:**
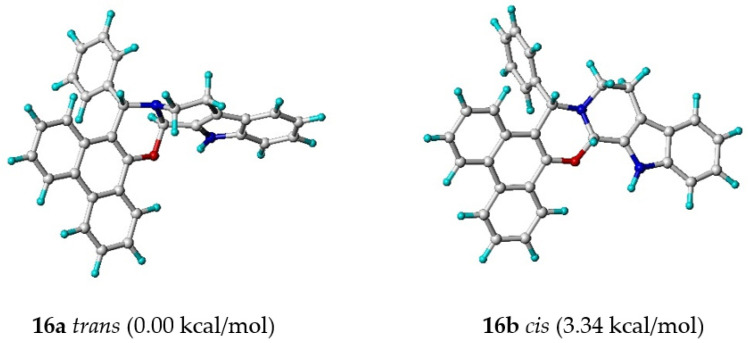
Most stable structures of the diastereotopic heterocycle **16** (DFT calculated relative energies on the B3LYP/6-311G(d,p) level of theory).

Determination of the relative configuration of **14**–**16** is based mainly on NOE interactions and their comparison with the corresponding calculated QC structures. As an example, β-carboline derivative **16** is examined (see Figure 1). Only one of the two possible diastereomers fits the experimental results: the position of methine hydrogens H-9a and H-18 was always found to be *trans*. The complete spatial information, found with the example of **16**, is collected in Table 4, comparing NOE results with the calculated distances from QM calculations.

### 2.4. Detailed NMR Analysis of New Phenanthr[9,10-e]Oxazines ***27a,b**−**28a,b***

In analogy to previous investigations [25] and to the results of the structural analyses of **14**–**16** (vide supra), reactions starting from **21**, in all cases, yielded *trans* isomers **24a**, **26a**, and **28a**. Their NMR spectra are akin to the corresponding spectra of the compounds without an OH group at position 2′ (**14**–**16**) and to compounds having a naphthalene moiety instead of phenanthrene [25].

**Figure 2:**
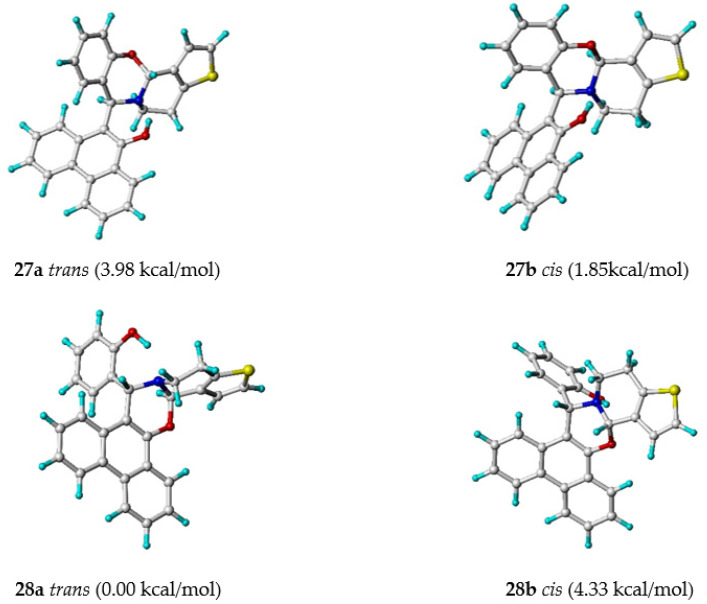
The most stable structures of regioisomeric heterocycles **27a**,**b** and **28a**,**b** including their *trans* (**a**) and *cis* (**b**) diastereomeric possibilities (DFT calculated relative energies on the B3LYP/6-311G(d,p) level of theory).

To get the preferred stereoisomers of **28a**,**b**, the *trans*/*cis* diastereomers of the regioisomers were calculated by the DFT method. Both the most stable structures and the corresponding energy differences are given in Figure 2. On the basis of these data, **28a** could be confirmed to be the most stable structure. Energy differences around 1 kcal/mol to the next coming structure are rather unequivocal. Consequently, this structure of *trans* isomer **28a** will be adjusted with the available experimental NMR [δ(^1^H)/ppm, δ(^13^C)/ppm, ^n^*J*_H,H_/Hz)] and spatial NMR information (qualitative NOE′s).

First, all ^1^H-NMR and ^13^C-NMR chemical shifts for **28a** could be unequivocally assigned. Hereby, the two singlets of protons H-16 and H-9a and the AX spin system of thiophene protons H-10 and H-11 can serve as useful starting points. The latter is the only spin system of aromatic protons with two doublets and with characteristic *ortho*-coupling constant of about 5.2 Hz. The high-field doublet (at 7.09 ppm) was assigned to the H-10 proton due to the substantial NOE to tertiary proton H-9a. On the other hand, no NOE between H-10 and the second tertiary proton (H-16) could be measured. The *trans* position of these two protons and, consequently, the *trans* stereochemistry of **28a** can be proved. Furthermore, the HMBC connectivity of the same proton singlet (H-16) established the access to both the ^1^H and ^13^C assignments of the present aromatic moieties ((i) to C-6′, C-1′, and C-2′ of the phenolic, and (ii) to C-16a and C-8b of the phenanthryl parts of the molecule), and to the piperidyl moiety via C-14. The *trans* stereochemistry of **28a** could be proved additionally by the strong NOEs of H-16 to one of the H-14 protons, to the aromatic proton H-6’ and to one of the H-14 protons. That is H-12a did not show any NOE to protons of the piperidyl ring.

The excellent agreement between the protons, proton distances, as calculated and the existence of strong NOEs in **28a** can serve as an excellent further proof of the present regio- and diastereoselectivity. Similar ^1^H/^13^C-NMR studies of the isoquinoline (**25a,b** and **26a,b**) and β-carboline analogues (**23a,b** and **24a,b**) allowed to arrive at the same conclusion. These compounds also prefer the same regioisomerism in *trans* configuration of **24a** and **26a**, respectively.

## 3. Materials and Methods

### 3.1. General Methods

Melting points were determined on a Hinotek X-4 (Ningbo, China) melting point apparatus. Elemental analyses were performed with a Perkin-Elmer 2400 CHNS elemental analyzer (Waltham, MA, USA) in the Institute of Pharmaceutical Chemistry, University of Szeged. Merck Kieselgel 60F254 plates (Budapest, Hungary)were used for TLC. Microwave reactions were performed with a CEM Discover SP microwave reactor (Matthwes, NC, USA).

The starting cyclic imines 3,4-dihydroisoquinoline (**2**) [26], 6,7-dihydrothieno[3,2-*c*]pyridine (**4**) [27] and 4,9-dihydro-β-carboline (**6**) [28] were synthesised according to literature processes.

Quantum chemical calculations were performed using the Gaussian 09 program package [29] and carried out on LINUX clusters. The various different conformations and configurations of the studied compounds were optimised [30]. The B3LYP density functional method was selected for all calculations. The method is based on Becke′s three-parameter hybrid functionals [31] and the correlation functional of Lee et al. [32]. All optimizations were carried out without any restriction at this B3LYP/6-311G(d,p) level of theory [33,34,35].

The ^1^H and ^13^C-NMR spectra were recorded in CDCl_3_, CD_2_Cl_2_, or DMSO-*d*_6_ solution in 5 mm tubes at room temperature on a Bruker Avance NEO spectrometer (Karlsruhe, Germany) at 400.18 (^1^H) MHz, or on a Bruker Avance spectrometer (Karlsruhe, Germany) at 500.17 (^1^H) and 125.77 (^13^C) MHz, or on a Bruker Avance III spectrometer (Karlsruhe, Germany) at 600.13 (^1^H) and 150.61 (^13^C) MHz, with the deuterium signal of the solvent as the lock and TMS as internal standard. All spectra (^1^H, ^13^C, gs-*H*,*H* COSY, edited HSQC, gs-HMBC and NOESY) were acquired and processed with the standard BRUKER software (TopSpin 3.6 or TopSpin 4.0).

### 3.2. Procedures

#### 3.2.1. General Procedure for the Synthesis of Hydroxyphenanthryl-Isoquinoline, -Thienopyridine and -β-Carboline (**3**, **5** and **7**)

The mixture of the cyclic imine [3,4-dihydroisoquinoline (**2**), 6,7-dihydrothieno[3,2-c]pyridine (**4**) or 4,9-dihydro-β-carboline (**6**) 0.51 mmol] and 9-phenanthrol (**1**: 100 mg, 0.51 mmol) in acetonitrile (5 mL) was placed in a 10-mL pressurised reaction vial and heated under microwave conditions at 100 °C. After the reaction completed, the mixture was cooled down and the formed crystals were filtered and washed with cold acetonitrile (2 × 5 mL).

*1-(9-Hydroxyphenanthr-10-yl)-1,2,3,4-tetrahydroisoquinoline (* **3** *)*

Reaction time: 20 min; recrystallised from iPr_2_O (5 mL); R_f_ = 0.70 (*n*-hexane/EtOAc, 2:1); 135 mg (82%); white crystals; m.p.: 147–149 °C; ^1^H-NMR (DMSO-*d*_6_, 500 MHz): δ 8.80 (1H, d, *J* = 8.3 Hz, H-4 or H-5), 8.80 (1H, d, *J* = 8.3 Hz, H-4 or H-5), 8.23(1H, d, *J* = 8.3 Hz, H-8), 8.17 (1H, d, *J* = 8.2 Hz, H-1), 7.67 (1H, m, H-6), 7.66 (1H, m, H-2), 7.60 (1H, t, *J* = 7.5 Hz, H-7), 7.52 (1H, t, *J* = 7.7 Hz, H-3), 7.18 (1H, d, *J* = 7.5 Hz, H-5′), 7.09 (1H, t, *J* = 7.4 Hz, H-6′), 6.86 (1H, t, *J* = 7.4 Hz, H-7′), 6.58 (1H, d, *J* = 7.9 Hz, H-8′), 6.10 (1H, s, H-1′), 3.44 (1H, m, H-3′), 3.20 (2H, m, H-3′, H-4′), 2.90 (1H, d, *J* = 14.1 Hz, H-4′); elemental analysis calcd (%) for C_23_H_19_NO (323.41): C 84.89, H 5.89, N 4.30; found: C 84.82, H 5.90, N 4.26. (Figures S1–S9).

*4-(9-Hydroxyphenanthr-10-yl)-4,5,6,7-tetrahydrothieno[3,2-c]pyridine (* **5** *)*

Reaction time: 35 min; recrystallised from iPr_2_O (4 mL); R_f_ = 0.75 (*n*-hexane/EtOAc, 2:1); 155 mg (92%); Light beige crystals; m.p.: 191–188 °C; ^1^H-NMR (DMSO-d_6_, 500 MHz): δ 14.33 (1H, d, *J* = 7.7 Hz, H-4 or H-5), 8.78 (1H, d, *J* = 7.9 Hz, H-4 or H-5), 8.19 (1H, d, *J* = 8.7 Hz, H-1), 8.17 (1H, dd, *J* = 8.2, 1.2 Hz, H-8), 7.66 (1H, m, H-6), 7.65 (1H, m, H-2), 7.60 (1H, t, *J* = 7.5 Hz, H-7), 7.51 (1H, t, *J* = 7.7 Hz, H-3), 7.06 (1H, d, *J* = 5.2 Hz, H-6′), 6.16 (1H, d, *J* = 5.2 Hz, H-7′), 6.01 (1H, s, H-1′), 4.74 (1H, br s, H-2′), 3.53 (1H, m, H-3′), 3:13 (2H, m, H-3′, H-4′), 2.94 (1H, m, H-4′); elemental analysis calcd (%) for C_21_H_17_NOS (331.43): C 76.10, H 5.17, N 4.23; found: C 76.25, H 5.17, N 4.13 (Figures S10–S18).

*1-(9-Hydroxyphenanthr-10-yl)-1,2,3,4-tetrahydro-β* *-carboline (* **7** *)*

Reaction time: 40 min; recrystallised from iPr_2_O (6 mL); R_f_ = 0.73 (*n*-hexane/EtOAc, 2:1); 146 mg (76%); brown crystals; m.p.: 205–207 °C; ^1^H-NMR (DMSO-d_6_, 500 MHz): δ 9.76 (1H, s, 9-OH), 8.83 (1H, d, *J* = 8.2 Hz, H-4 or H-5), 8.81 (1H, d, *J* = 8.2 Hz, H-4 or H-5), 8.29 (1H, d, *J* = 8.3 Hz, H-8), 8.17 (1H, dd, *J* = 8.1, 0.9 Hz, H-1), 7.68 (2H, m, H-6, H-7), 7.59 (1H, t, *J* = 7.6 Hz, H-2), 7.55 (1H, t, *J* = 7.5 Hz, H-3), 7.44 (1H, m, H-5′); 7.15 (1H; m; H-8′), 6.95 (2H, m, H-6′, H-7′), 6.24 (1H, s, H-1′), 3.55 (1H, dd, *J* = 11.5, 5.1 Hz, H-3′), 3.18 (1H, ddd, *J* = 11.6, 11.6, 5.1 Hz, H-3′), 3.02 (1H, m, H-4′); 2:87 (1H, dd, *J* = 15.4 Hz, H-4′); elemental analysis calcd (%) for C_25_H_20_N_2_O (364.45): C 82.39, H 5.53, N 7.69; found: C 82.25, H 5.49, N 7.72 (Figures S19–S26).

#### 3.2.2. General Procedure for the Synthesis of Phenanthr[9,10-*e*][1,3]Oxazinoisoquinoline,-Thienopyridine and -β-Carboline (**8**–**10**)

Aminophenanthrols (**3**, **5** and **7**; 0.25 mmol) was dissolved in 10 mL CHCl_3_, aqueous solution of formaldehyde (20 mg, 0.66 mmol, 35%) was then added and the mixture was stirred at room temperature. The mixture was next extracted with 10 mL distilled water. The organic phase was collected and then dried on Na_2_SO_4_. The solvent was evaporated off and the residue was crystallised from n-hexane (10 mL).

*Phenanthr[9,10-e][1,3]* *oxazino[4,3-a]isoquinoline (* **8** *)*

Reaction time: 25 min; recrystallised from *n*-hexane: iPr_2_O (4:1, 5 mL); R_f_ = 0.65 (*n*-hexane/EtOAc, 2:1); 73 mg (87%); Purple crystals. m.p.: 139–141 °C ^1^H-NMR (CD_2_Cl_2_, 500 MHz): δ 8.71 (2H, m, H-4, H-5), 8.28 (1H, d, *J* = 8.1 Hz, H-8), 7.73 (1H, m, H-1), 7.70 (1H, m, H-6), 7.63 (1H, t, *J* = 7.5 Hz, H-7), 7.54 (2H, m, H-2, H-3), 7.24 (1H, d, *J* = 7.3 Hz, H-14), 7.20 (1H, t, *J* = 7.3 Hz, H-15), 6.97 (1H, t, *J* = 7.4 Hz, H-16), 6.85 = (1H, d, *J* = 7.7 Hz, H-17), 5.54 (1H, s, H-17b), 4.76 (1H, d, *J* = 7.0 Hz, H-10), 4.68 (1H, d, *J* = 6.9 Hz, H-10), 3.87 (1H, m H-12), 2.99 (3H, m, H-12, H-13); elemental analysis calcd (%) for C_24_H_19_NO (337.42): C 85.43, H 5.68, N 4.15; found: C 85.35, H 5.56, N 4.20 (Figures S27–S35).

*Phenanthr[9,10-e][1,3]* *oxazino[4,3-a]thieno[3,2-c]pyridine (* **9** *)*

Reaction time: 20 min; recrystallised from *n*-hexane: iPr_2_O (4:1, 4 mL); R_f_ = 65 (*n*-hexane/EtOAc, 2:1); 80 mg (94%); Yellow crystals. m.p.: 186–188 °C ^1^H-NMR (CD_2_Cl_2_, 500 MHz): δ 8.70 (1H, d, *J* = 8.1 Hz, H-4), 8.69 (1H, d, *J* = 8.2 Hz, H-5), 8.26 (1H, d, *J* = 8.1 Hz, H-8), 7.99 (1H, d, *J* = 8.1 Hz, H-1), 7.69 (1H, t, *J* = 7.5 Hz, H-6), 7.63 (2H, m, H-2, H-7), 7.58 (1H, t, *J* = 7.7 Hz, H-3), 6.95 (1H, d, *J* = 5.2 Hz, H-15), 6.66 (1H, d, *J* = 5.2 Hz, H-16), 5.67 (1H, s, H-16b), 4.96 (1H, d, *J* = 6.9 Hz, H-10), 4.93 (1H, d, *J* = 6.9 Hz, H-10), 3.75 (1H, ddd, *J* = 14.2, 12.2, 6.0 Hz, H-12), 3.66 (1H, dd, *J* = 14.3, 6.8 Hz, H-12), 3.03 (1H, m, H-13), 2.87 (1H, dd, *J* = 17.1, 5.7 Hz, H-13); elemental analysis calcd (%) for C_22_H_17_NOS (343.44): C 76.94, H 4.99, N 4.08; found: C 76.86, H 5.02, N 4.13 (Figures S36–S45).

*Phenanthr[9,10-e][1,3]* *oxazino[4,3-a]-β* *-carboline (* **10** *)*

Reaction time: 30 min; recrystallised from *n*-hexane: iPr_2_O (4:1, 5 mL); R_f_ = 0.60 (*n*-hexane/EtOAc, 2:1); 74 mg (79%); Dark pink crystals. m.p.: 183–185 °C ^1^H-NMR (CD_2_Cl_2_, 500 MHz): δ 8.78 (1H, d, *J* = 8.2 Hz, H-4), 8.71 (1H, d, *J* = 8.3 Hz, H-5), 8.28 (1H, dd, *J* = 8.2, 0.7 Hz, H-8), 8.15 (1H, d, *J* = 8.1 Hz, H-1), 7.85 (1H, br s, H-18), 7.76 (1H, t, *J* = 7.5 Hz, H-2), 7.71 (1H, t, *J* = 7.6 Hz, H-6), 7.66 (1H, t, *J* = 7.5 Hz, H-3), 7.64 (1H, t, *J* = 7.6 Hz, H-7), 7.51 (1H, m, H-14), 7.11 (1H, m, H-17), 7.06 (2H, m, H-15, H-16), 5.92 (1H, s, H-18b), 5.01 (1H, d, *J* = 7.1 Hz, H-10), 4.95 (1H, d, *J* = 7.1 Hz, H-10), 3.77 (1H, ddd, *J* = 14.2, 12.0, 5.7 Hz, H-12), 3.71 (1H, dd, *J* = 14.3, 6.3 Hz, H-12), 2.99 (1H, dddd, *J* = 16.2, 11.9, 6.7, 2.4 Hz, H-13), 2.83 (1H, dd, *J* = 16.3, 5.1 Hz, H-13); elemental analysis calcd (%) for C_26_H_20_N_2_O (376.46): C 82.95, H 5.36, N 7.44; found: C 83.03, H 5.45, N 7.32 (Figures S46–S56).

*10-[(Phenyl)-morpholin-4-yl-methyl]-9-phenanthrol (* **13** *)*

Briefly, 9-phenanthrol (1.0 g, 5.13 mmol), benzaldehyde (0.54 g, 5.13 mmol), and morpholine (0.45 g, 5.13 mmol) were stirred and heated at 80 °C under neat conditions for 4 h. The residue was crystallized with *n*-hexane (14 mL) and recrystallised from iPr_2_O (9 mL) R_f_ = 0.75 (*n*-hexane/EtOAc, 2:1); 1.38g (73%); Light beige crystals; m.p.: 107–109 °C; ^1^H-NMR (DMSO-d_6_, 500 MHz): δ 14.37 (1H, s, 9-OH), 8.75 (1H, d, *J* = 7.6 Hz, H-5), 8.71 (1H, d, *J* = 7.8 Hz, H-4), 8.39 (1H, m, H-8), 8.07 (1H, d, *J* = 8.3 Hz, H-1), 7.69 (4H, m, H-6, H-7, H-2′), 7.51 (1H, t, *J* = 7.7 Hz, H-2), 7.42 (1H, t, *J* = 7.5 Hz, H-3), 7.31 (2H, t, *J* = 7.5 Hz, H-3′); 7.23 (1H, t, *J* = 7.3 Hz, H-4′), 5.45 (1H, s, H-11), 3.72 (4H, m, H-3”), 3.72 (1H, m, H-2”); elemental analysis calcd (%) for C_25_H_23_NO_2_ (369.46): C 81.27, H 6.28, N 3.79; found: C 81.31, H 6.12, N 3.71 (Figures S57–S65).

#### 3.2.3. General Procedure for the Synthesis of Phenanthr[9,10-*e*][1,3]Oxazines (**14**, **15** and **16**)

A mixture of the appropriate aminophenanthrol **13** (60 mg 0.16 mmol) and 0.13 mmol of the cyclic imine (3,4-dihydroisoquinoline (**2**), 6,7-dihydrothieno[3,2-c]pyridine (**4**), or 4,9-dihydro-β-carboline (**6**)) in 1,4-dioxane (5 mL) was placed in a 10 mL pressurized reaction vial and heated under microwave conditions at 80 °C for 20 min. The solvent was removed under reduced pressure, and the residue was isolated by crystallisation with MeOH (5 mL).

9aR*,17S*-15-(Phenyl)-phenanthr[9,10-e]oxazino[2,3-a]isoquinoline ( **14** *)*

Recrystallised from *n*-hexane: iPr_2_O (2:1, 4 mL); R_f_ = 0.62 (*n*-hexane/EtOAc, 2:1); 56 mg (86%); Light yellow crystals. m.p.: 209–211 °C ^1^H-NMR (CD_2_Cl_2_, 500 MHz): δ 8.72 (1H, d, *J* = 8.3 Hz, H-5), 8.69 (1H, d, *J* = 8.4 Hz, H-4), 8.28 (1H, dd, *J* = 8.2, 0.9 Hz, H-8), 7.70 (1H, ddd, *J* = 8.3, 7.0, 1.3 Hz, H-6), 7.61 (1H, ddd, *J* = 8.1, 7.0, 1.1 Hz, H-7), 7.51-7.41 (6H, m, H-1, H-2, H-3, H-13, H-2′); 7:37-7:24 (6H, m, H-10, H-11, H-12, H-3′, H-4′), 5.83 (1H, s, H-9a), 5.49 (1H, s, H-17), 3.40 (1H, m, H-15-ψ-ax), 3.31 (1H, m, H-14-ψ- ax), 3.18 (1H, dd, *J* = 10.4, 5.7 Hz, H-15-ψ-eq), 2.89 (1H, dd, *J* = 16.0, 2.8 Hz, H-14-ψ-eq);^13^C-NMR (CD_2_Cl_2_, 125 MHz): δ 148.1 (C-8b), 142.9 (C-1′), 135.7 (C-13a), 133.5 (C-9b), 131.8 (C-4a or 4b), 131.3 (C-4a or 4b), 129.8 (C-2′), 129.3 (C-10 or 12 or 13), 129.2 (C-10 or 12 or 13), 129.1 (C-10 or 12 or 13), 128.6 (C-3′), 127.8 (C-4′), 127.5 (C-6), 127.3 (C-2), 126.9 (C-7), 126.5 (C-8a), 126.4 (C-11), 125.9 (C-17b), 124.3 (C-3), 123.7 (C-1), 123.2 (C-4), 122.9 (C-5), 122.8 (C-8), 107.8 (C-17a), 82.9 (C-9a), 63.3 (C-17), 45.9 (C-15), 29.8 (C-14); elemental analysis calcd (%) for C_30_H_23_NO (413.52): C 87.14, H 5.61, N 3.39; found: C 87.09, H 5.45, N 3.32 (Figures S66–S78).

9aR*,16S*-16-(Phenyl)-phenanthr[9,10-e]oxazino[2,3-a]thieno[3,2-c]pyridine ( **15** *)*

Recrystallised from *n*-hexane: iPr_2_O (2:1, 4 mL); R_f_ = 0.68 (*n*-hexane/EtOAc, 2:1); 63 mg (94%); white crystals. m.p.: 216–218 °C ^1^H-NMR (CD_2_Cl_2_, 500 MHz): δ 8.71 (1H, d, *J* = 8.3 Hz, H-5), 8.67 (1H, d, *J* = 8.5 Hz, H-4), 8.29 (1H, dd, *J* = 8.1, 0.9 Hz, H-8), 7.70 (1H, ddd, *J* = 8.3, 7.0, 1.3 Hz, H-6), 7.62 (1H, ddd, *J* = 8.1, 7.0, 1.1 Hz, H-7), 7.47 (2H, m, H-1, H-3), 7.42 (1H, m, H-2), 7.37 (2H, m, H-2′), 7.29 (2H, m, H-3′), 7.25 (1H, m, H-4′), 7.21 (1H, d, *J* = 5.2 Hz, H-11), 7.11 (1H, d, *J* = 5.2 Hz, H-10), 5.83 (1H, s, H-9a), 5.51 (1H, s, H-16), 3.42 (1H, m, H-14-ψ-ax), 3.24 (2H, m, H-14-ψ-eq, H-13-ψ-ax), 2.94 (1H, m, H-13-ψ-eq);^13^C-NMR (CD_2_Cl_2_, 125 MHz): δ 147.9 (C-8b), 142.9 (C-1′), 138.7 (C-12a), 133.8 (C-9b), 131.7 (C-4a), 131.3 (C-4b), 129.8 (C-2′), 128.6 (C-3′), 127.9 (C-4′), 127.6 (C-6), 127.3 (C-2), 126.9 (C-7), 126.5 (C-8a), 126.3 (C-10), 125.8 (C-16b), 124.3 (C-3), 123.8 (C-1), 123.6 (C-11), 123.2 (C-4), 122.8 (C-5. 8), 107.8 (C-16a), 79.6 (C-9a), 62.9 (C-16), 46.7 (C-14), 26.5 (C-13); elemental analysis calcd (%) for C_28_H_21_NOS (419.54): C 80.16, H 5.05, N 3.34; found: C 83.10, H 5.15, N 3.32 (Figures S79–S91).

9aR*,18S*-19-(Phenyl)-phenanthr[9,10-e]oxazino[2,3-a]-β *-carboline (* **16** *)*

Recrystallised from *n*-hexane: iPr_2_O (2:1, 4 mL); R_f_ = 0.70 (*n*-hexane/EtOAc, 2:1); 55 mg (76%); Light yellow crystals. m.p.: 180–182 °C ^1^H-NMR (CD_2_Cl_2_, 500 MHz): δ 8.72 (1H, *J* = 8.3 Hz, H-5), 8.68 (1H, d, *J* = 7.9 Hz, H-4), 8.31 (1H, br s, H-10), 8.30 (1H, d, *J* = 8.3 Hz, H-8), 7.70 (1H, ddd, *J* = 8.3, 7.0, 1.3 Hz, H-6), 7.61 (1H, m, H-7), 7.58 (1H, d, *J* = 8.1 Hz, H-14), 7.51 (1H, m, H-1), 7.49 (1H, m, H-3), 7.45 (1H, m, H-2), 7.42 (1H, m, H-11), 7.41 (2H, m, H-2′), 7.30 (2H, m, H-3′), 7.27 (1H, m, H-4′), 7.23 (1H, ddd, *J* = 8.2, 7.1, 1.1 Hz, H-12), 7.14 (1H, t, *J* = 7.5 Hz, H-13), 5.98 (1H, s, H-9a), 5.58 (1H, s, H-18), 3.46 (1H, m, H-16-ψ-ax), 3.32 (1H, dd, *J* = 10.9, 5.5 Hz, H-16-ψ-eq), 3.13 (1H, ddd, *J* = 15.5, 11.8, 5.9 Hz, H-15-ψ-ax), 2.89 (1H, ddd, *J* = 15.4, 4.2, 1.3 Hz, H-15-ψ-eq); ^13^C-NMR (CD_2_Cl_2_, 125 MHz): δ 47,6 (C-8b), 142,8 (C-1′), 136,9 (C-10a), 131,7 (C-4a), 131,3 (C-4b), 130,9 (C-9b), 129,8 (C-2′), 128,7 (C-3′), 128,0 (C-4′), 127,6 (C-6), 127,4 (C-2), 126,9 (C-7), 126,6 (C-8a or 14a), 126,5 (C-8a or 14a), 125,8 (C-18b), 124,4 (C-3), 123,6 (C-1), 123,3 (C-4), 123,1 (C-12), 122,8 (C-5), 122,7 (C-8), 120,0 (C-13), 119,4 (C-14), 111,8 (C-11), 111,5 (C-14b), 108,2 (C-18a), 78,7 (C-9a), 62,8 (C-18), 47,3 (C-16), 22,6 (C-15); elemental analysis calcd (%) for C_32_H_26_N_2_O (454.57): C 84.55, H 5.77, N 6.16; found: C 84.49, H 5.67, N 6.21 (Figures S92–S104).

*14-Morpholin-4-yl-dibenzo[a,c]xanthene (* **19** *)*

Briefly, 9-phenanthrol (100. mg, 0.51 mmol), salicylic aldehyde (62 mg, 0.51 mmol), and morpholine (44 mg, 0.51 mmol) were stirred and heated at 80 °C under neat conditions for 4 h. The residue was crystallised with iPr_2_O (15 mL) and recrystallised from *n*-hexane: EtOAc (10 mL; 10:1) R_f_ = 0.65 (*n*-hexane/EtOAc, 2:1); 127 mg (68%); Browne crystals; m.p.: 176–178 °C; ^1^H-NMR (CDCl_3_, 500 MHz): δ 8.68 (2H, m, H4, H-5), 8.58 (1H, m, H-1), 8.22 (1H, d, *J* = 7.5 Hz, H-8), 7.71 (1H, m, H-2), 7.66 (2H, m, H-3, H-7), 7.60 (1H, m, H-6), 7.40 (3H, m, H-11, H-12, H-14), 7.23 (1H, m, H-13), 5.52 (1H, s, H-15), 3.52 (4H, m, H-2′), 2.52 (2H, m H-3′), 2.40 (2H, m H-3′); ^13^C-NMR (CDCl_3_, 125 MHz) δ 153.0 (C-10a), 147.2 (C-9), 131.3 (C-4a or C-4b), 131.2 (C-4a or C-4b), 130.0 (C-14), 128.8 (C-12), 128.3 (C-15b), 127.7 (C-2 or C-3 or C-7), 127.2 (C-2 or C-3 or C-7), 127.0 (C-2 or C-3 or C-7), 125.2 (C-6), 124.6 (C-8a), 124.5 (C-8), 123.2 (C-13), 123.0 (C-1 or C-4 or C-5), 122.8 (C-1 or C-4 or C-5), 122.7 (C-1 or C-4 or C-5), 117.6 (C-14a), 116.5 (C-11), 110.5 (C-15a), 67.5 (C-3′), 57.7 (C-15), 48.7 (C-2′);. elemental analysis calcd (%) for C_25_H_21_NO_2_ (367.45): C 81.72, H 5.76, N 3.81; found: C 81.34, H 5.65, N 3.77 (Figures S105–S113).

*10-[(2-Hydroxyphenyl)-pyrrolidin-1-yl-methyl]-9- phenanthrol (* **21** *)*

Briefly, 9-phenanthrol (500 mg, 2.56 mmol), salicylic aldehyde (312 mg, 2.56 mmol), and pyrrolidine (182 mg, 2.56 mmol) were stirred and heated at 80 °C under neat conditions for 4 h. The residue was crystallised with *n*-hexane (16 mL) and recrystallised from iPr_2_O (11 mL) R_f_ = 0.70 (*n*-hexane/EtOAc, 2:1); 718 mg (76%); Light beige crystals; m.p.: 172–174 °C; ^1^H-NMR (DMSO-d_6_, 500 MHz): δ 15.32 (1H, br s, OH), 10.12 (1H, br s, OH), 8.74 (1H, d, *J* = 8.4 Hz, H-5), 8.68 (1H, d, *J* = 8.1 Hz, H-4), 8.38 (1H, d, *J* = 8.5 Hz, H-8), 7.94 (1H, d, *J* = 8.3 Hz, H-1), 7.67 (2H, m, H-6, H-7), 7.45 (1H, t, *J* = 7.5 Hz, H-2), 7.38 (2H, m, H-3, H-6′), 7.04 (1H, t, *J* = 7.4 Hz, H-4′), 6.91 (1H, d, *J* = 8.1 Hz, H-3′), 6.66 (1H, t, *J* = 7.4 Hz, H-5′), 5.82 (1H, s, H-11), 3.16 (2H, m, H-2”), 2.66 (2H, m, H-3”), 2.49 (2H, m, H-3”), 2.40 (2H, m, H-2”); ^13^C-NMR (DMSO-d_6_, 125 MHz): δ 154.3 (C-2′), 151.9 (C-9), 131.3 (C-4a), 130.3 (C-4b), 128.7 (2C, C-4′, C-6′), 126.9 (C-8a), 126.9 (2C, C-2, C-6), 126.2 (C-7), 124.9 (C-10a), 123.0 (C-4), 122.9 (C-3), 122.6 (C-8), 122.4 (C-5), 121.5 (C-1), 119.7 (C-5′), 115.5 (C-3′), 112.4 (C-10), 61.1 (C-11), 54.1 (C-2”), 49.6 (C-3”); elemental analysis calcd (%) for C_25_H_23_NO_2_ (369.46): C 81.27, H 6.28, N 3.79; found: C 81.31, H 6.15, N 3.76 (Figures S114–S125).

#### 3.2.4. General Procedure for the Synthesis of Phenanthr[9,10-*e*][1,3]Oxazines (**24**, **26** and **28**)

The mixture of aminophenanthrol **21** (50 mg 0.13 mmol) and 0.13 mmol of the correspondent cyclic imine (3,4-dihydroisoquinoline (**2**), 6,7-dihydrothieno[3,2-c]pyridine (**4**), or 4,9-dihydro-β-carboline (**6**))_was dissolved in 1,4-dioxane (5 mL) and placed in a 10 mL pressurized reaction vial. The mixture was heated at 80 °C for 15 min under microwave conditions. The solvent was removed in vacuo, and the residue was isolated by crystallisation with MeOH (5 mL).

9aR*,18S*-19-(2-Hydroxyphenyl)-phenanthr[9,10-e]oxazino[2,3-a]-β *-carboline (* **24** *)*

Recrystallised from iPr_2_O (7 mL); R_f_ = 0.38 (n-hexane/EtOAc, 2:1); 47 mg (78%). Brown crystals; m.p.: 163–165 °C ^1^H-NMR (CDCl_3_, 500 MHz): δ 8.69 (2H, br s, H-4 and H-5), 8.21 (2H, br s, H-8 and H-10), 7.69 (1H, t, *J* = 7.1 Hz, H-6), 7.57 7.54 (5H, m, H-1, H-2, H-3, H-7 and H-14), 7.41 (1H, d, *J* = 7.8 Hz, H-11), 7.16 (2H, m, H-13 and H-4′), 6.95 (1H, d, *J* = 7.7 Hz, H-3′), 6.64 (2H, br s, H-5′ and H-6′), 6.12 (1H, s, H-9a), 5.82 (1H, s, H-18), 3.44 (1H, m, H-16-ψ-ax), 3.31 (1H, m, H-16-ψ-eq), 3.21 (1H, m, H-15-ψ-ax), 2.97 (1H, dd, *J* = 15.7, 3.1 Hz, H-15-ψ-eq); ^13^C-NMR (CDCl_3_, 125 MHz): δ 156.8 (C-2′), 146.6 (C-8b), 136.8 (C-10a), 131.5 (C-4a or 4b), 131.4 (C-4a or 4b), 131.0 (C-6′), 129.9 (C-9b), 129.8 (C-4′), 127.8 (C-6 or C-2), 127.7 (C-6 or C-2), 126.8 (C-7), 126.4 (C-14a or C-8a), 126.3 (C-14a or C-8a), 125.5 (C-18b), 125.2 (C-1′), 124.7 (C-3), 123.4 (C-12), 123.2 (C-1. 5), 122.7 (C-8 or C-4), 122.6 (C-8 or C-4), 120.3 (C-13), 120.1 (C-5′), 119.4 (C-14), 117.7 (C-3′), 111.7 (C-11), 110.9 (C-14b), 106.0 (C-18a), 77.7 (C-9a), 60.7 (C-18), 45.4 (C-16), 22.4 (C-15);. elemental analysis calcd (%) for C_32_H_24_N_2_O_2_ (468.56): C 82.03, H 5.16, N 5.98; found: C 82.12, H 5.09, N 5.82 (Figures S126–S139).

9aR*,17S*-15-(2-Hydroxyphenyl)-phenanthr[9,10-e]oxazino[2,3-a]isoquinoline ( **26** *)*

Recrystallised from iPr_2_O (5 mL); R_f_ = 0.35 (-*n*-hexane/EtOAc, 2:1); 47 mg (85%). Light beige crystals; m.p.: 194–196 °C ^1^H-NMR (CDCl_3_, 500 MHz): δ 9.67 (1H, br s, 2′-OH) 8.68 (2H, m, H-4 and H-6), 8.22 (1H, d, *J* = 8.2 Hz, H-8), 7.68 (1H, d, *J* = 7.6 Hz, H-6), 7.53 (4H, m, H-1, H-2, H-3 and H-7), 7.41 (1H, d, *J* = 7.5 Hz, H-10), 7.37 (1H, t, *J* = 7.6 Hz, H-12), 7.31 (1H, t, *J* = 7.4 Hz, H-11), 7.24 (1H, m, H-13), 7.19 (1H, m, H-4′), 6.95 (1H, d, *J* = 8.1 Hz, H-3′), 6.66 (2H, m, H-5′ and H-6′), 5.96 (1H, s, H-9a), 5.73, (1H, s, H-17), 3.37 (1H, m, H-15-ψ-ax), 3.32 (1H, m, H-14-ψ-ax), 3.18 (1H, m, H-15-ψ-eq), 2.95 (1H, m, H-14-ψ-eq); ^13^C-NMR (CDCl_3_, 125 MHz): δ (C-147.1 (C-8b), 134.3 (C-13a), 132.7 (C-9b), 131.6 (C-4a or C-4b), 131.4 (C-4a or C-4b), 131.2 (C-6′), 129.7 (C-4′), 129.5 (C-12), 129.3 (C-10), 129.0 (C-13), 127.7 (C-6), 127.6 (C-2), 126.8 (C-7), 126.7 (C-11), 126.4 (C-8a), 125.7 (C-17b), 125.4 (C-1′), 124.5 (C-3), 123.3 (C-1), 123.2 (C-4 or C-5), 122.9 (C-8), 122.6 (C-4 or C-5), 120.1 (C-5′), 117.6 (C-3′), 105.6 (C-17a), 81.9 (C-9a), 61.1 (C-17), 44.0 (C-15), 29.4 (C-14); elemental analysis calcd (%) for C_30_H_23_NO_2_ (429.52): C 83.89, H 5.40, N 3.26; found: C 83.78, H 5.35, N 3.29 (Figures S140–S152).

9aR*,16S*-16-(2-Hydroxyphenyl)-phenanthr[9,10-e]oxazino[2,3-a]thieno[3,2-c]pyridine ( **28** *)*

Recrystallised from iPr_2_O (4 mL); R_f_ = 0.40 (*n*-hexane/EtOAc, 2:1); 52 mg (93%). Light beige crystals; m.p.: 181–183 °C ^1^H-NMR (CDCl_3_, 500 MHz): δ 9.65 (1H, br s, 2′-OH), 8.69 (2H, m, H-4, H-5), 8.25 (1H, d, *J* = 8.1 Hz, H-8), 7.69 (1H, t, *J* = 7.5 Hz, H-6), 7.60 (1H, m, H-7), 7.55 (1H, m, H-1), 7.53 (1H, m, H-3), 7.50 (1H, m, H-2), 7.22 (1H, d, *J* = 5.3 Hz, H-11), 7.19 (1H, m, H-4′); 7:09 (1H, d, *J* = 5:1 Hz, H-10), 6:96 (1H, d, *J* = 8:0 Hz, H-3′), 6.65 (1H, m, H-5′), 6.63 (1H, m, H-6′), 5.98 (1H, s, H-9a), 5.77 (1H, s, H-16), 3.40 (1H, m, H-14-ψ-ax), 3.30 (1H, m, H-13-ψ-ax), 3.25 (1H, m, H-14-ψ-eq), 3.01 (1H, m, H-13-ψ-eq); ^13^C-NMR (CDCl_3_, 125 MHz): δ 156.6 (C- 2′), 146.9 (C- 8b), 137.4 (C- 12a), 133.2 (C- 9b), 131.6 (C- 4a or C-4b), 131.4 (C- 4a or C-4b), 131.1 (C- 6′), 129.7 (C- 4′), 127.7 (C- 6), 127.6 (C- 2), 126.8 (C- 7), 126.4 (C- 8a), 126.2 (C- 10), 125.6 (C- 16b or C-1′), 125.3 (C- 16b or C-1′), 124.6 (C- 3), 124.2 (C- 11), 123.2 (C- 5), 123.2 (C- 10), 122.8 (C- 4 or C-8), 122.6 (C- 4 or C-8), 120.1 (C- 5′), 117.6 (C- 3′), 105.5 (C- 16a), 78.7 (C- 9a), 60.7 (C- 16), 44.8 (C- 14), 26.2 (C- 13); elemental analysis calcd (%) for C_28_H_21_NO_2_S (435.54): C 77.22, H 4.86, N 3.22; found: C 77.12, H 4.75, N 3.31 (Figures S153–S165).

## 4. Conclusions

Herein, 9-phenanthrol, a unique electron-rich aromatic compound, was tested in the modified *aza*-Friedel–Crafts reaction. The reactions of 3,4-dihydroisoquinoline, 6,7-dihydrothieno[3,2-*c*]pyridine or 3,4-dihydro-β-carboline as cyclic imines led to the formation of the corresponding bifunctional phenanthrol derivatives that were further transformed to the new phenanthr[9,10-*e*][1,3]oxazines (**8**−**10**). Furthermore, 9-phenanthrol was aminoalkylated by using morpholine in the presence of benzaldehyde: The functionalised aminophenanthrol (**21**) could only be synthesised by using salicylic aldehyde in the presence of pyrrolidine. Morpholine in this latter modified Mannich reaction led to the formation of 14-morpholinyl-dibenzo[*a*,*c*]xanthene (**19**). Phenanthrol-based bifunctional Mannich products were further tested in [4 + 2] cycloaddition by using 3,4-dihydroisoquinoline, 6,7-dihydrothieno[3,2-*c*]pyridine or 3,4-dihydro-β-carboline as dienophiles. The regio- and diastereoselectivity of the reactions were proved by NMR spectroscopy and supported by DFT calculations. All products were found to have *trans*-configuration (**14**–**16**, **24a**, **26a**, and **28a**). Applying functionalised aminophenanthrol precursors in [4 + 2] cycloaddition, the reactions regioselectively led to the formation of new phenanthr[9,10-*e*][1,3]oxazines (**24a**, **26a**, and **28a**) in good yields.

## Figures and Tables

**Table 1:** Synthesis of aminophenanthrols **3**, **5** and **7** under varied reaction conditions.

Products	Type of Heating	Solvent	Reaction Time	Temperature	Yield (%)
3	Oil bath	-	120 min	80 °C	10
	Oil bath	-	60 min	100 °C	19
	Oil bath	Acetonitrile	60 min	90 °C	49
	MW	Acetonitrile	10 min	100 °C	67
	MW	Acetonitrile	20 min	100 °C	82
5	MW	Acetonitrile	20 min	100 °C	72
	MW	Acetonitrile	35 min	100 °C	92
7	MW	Acetonitrile	20 min	100 °C	63
	MW	Acetonitrile	40 min	100 °C	76

**Table 2:** Syntheses of phenanthr[9,10-*e*]oxazine derivatives (**14**–**16**) under varied reaction conditions.

Product	Reaction Time	Temperature (°C)	Yield (%)
**14**	15 min	60	47
		80	86
		100	29
**15**	15 min	60	52
		80	94
		100	37
**16**	15 min	60	21
		80	76
		100	32

**Table 3:** Optimizing reaction conditions for the synthesis of **24b**, **26b** and **28b.**

Products	Reaction Time	Temperature (°C)	Yield (%)
**23a,b** **24a,b**	15 min	60	48
		80	85
		100	33
**25a,b** **26a,b**	15 min	60	28
		80	78
		100	19
**27a,b** **28a,b**	15 min	60	51
		80	93
		100	38

**Table 4:** Experimental NOE′s and calculated distances in **16** (as DFT calculated on the B3LYP/6-311G(d,p) level of theory).

Positions	1/18	9a/18	18/16 (ř-eq)	9a/16 (ř-eq)	9a/16 (ř-ax)	9a/10	14/15 (ř-ax)	14/15 (ř-eq)
**Measured NOE**	strong	medium	strong	weak	weak	medium	weak	medium
**Calculated distances d [Ĺ]**	2.2 2.2(*cis*)	3.6 2.7(*cis*)	2.2 2.7(*cis*)	4.1 3.8(*cis*)	3.8 2.7(*cis*)	2.8 3.0(*cis*)	4.3 3.4(*cis*)	2.9 2.9(*cis*)
**NOE-estimated distances d [Ĺ]**	2.2	3.4	2.3	4.4	4.3	2.8	3.5	3.0

ψ-eq (smaller signal at higher field); ψ-ax (the corresponding broadened signal at lower field).

## Supplementary Materials

Supplementary materials are available online: Figures S1−S165.
