# Supplementary material for: Synthesis and Conformational Analysis of Naphthoxazine-Fused Phenanthrene Derivatives
Source: Molecules. 2020 May 28;25(11):2524. doi: 10.3390/molecules25112524 (PMC7321197; doi:10.3390/molecules25112524)
Supplement: Supplementary file 1 [file molecules-25-02524-s001.pdf]

Supplementary information

Synthesis and Conformational Analysis of Naphthoxazine-Fused Phenanthrene Derivatives

Khadija Belasri [1,2], Leila Topal [1], Matthias Heydenreich [3], Andreas Koch [3], Erich Kleinpeter [3] Ferenc Fülöp [1,2] and István Szatmári [1,2,*]

[1] Institute of Pharmaceutical Chemistry and MTA-SZTE Stereochemistry Research Group, Hungarian Academy of Sciences, University of Szeged, Eötvös u. 6, H-6720 Szeged, Hungary; belasrikhadija15@gmail.com (K.B.); topal.leila@gmail.com (L.T.); fulop@pharm.u-szeged.hu (F.F.)

[2] Institute of Pharmaceutical Chemistry, University of Szeged, Interdisciplinary excellence center, H-6720 Szeged, Hungary;

[3] Department of Chemistry, University of Potsdam, Karl-Liebknecht-Str. 24-25, D-14476 Potsdam (Golm), Germany; mheydenr@uni-potsdam.de (M.H.); kochi@uni-potsdam.de (A.K.); ekleinp@uni-potsdam.de (E.K.)

* Correspondence: szatmari.istvan@pharm.u-szeged.hu; Tel.: +36-62-341-966

1-(9-Hydroxyphenanthr-10-yl)-1,2,3,4-tetrahydroisoquinoline (3)

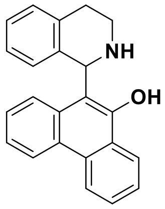

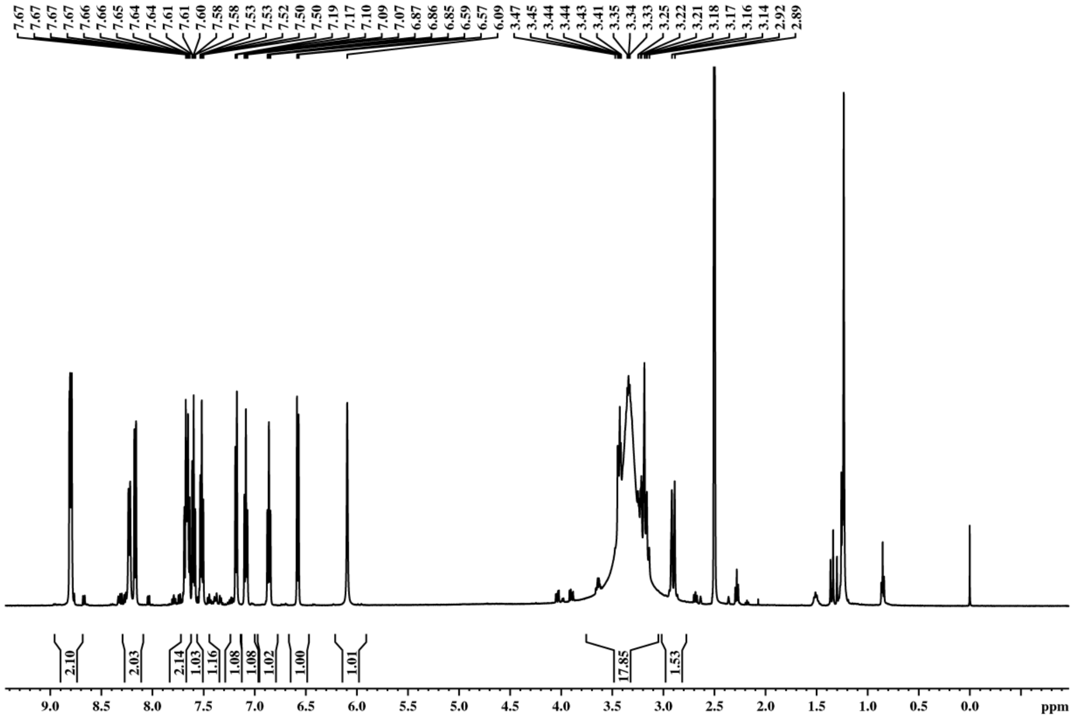

Figure S1. ^{1}H NMR spectrum of **3**

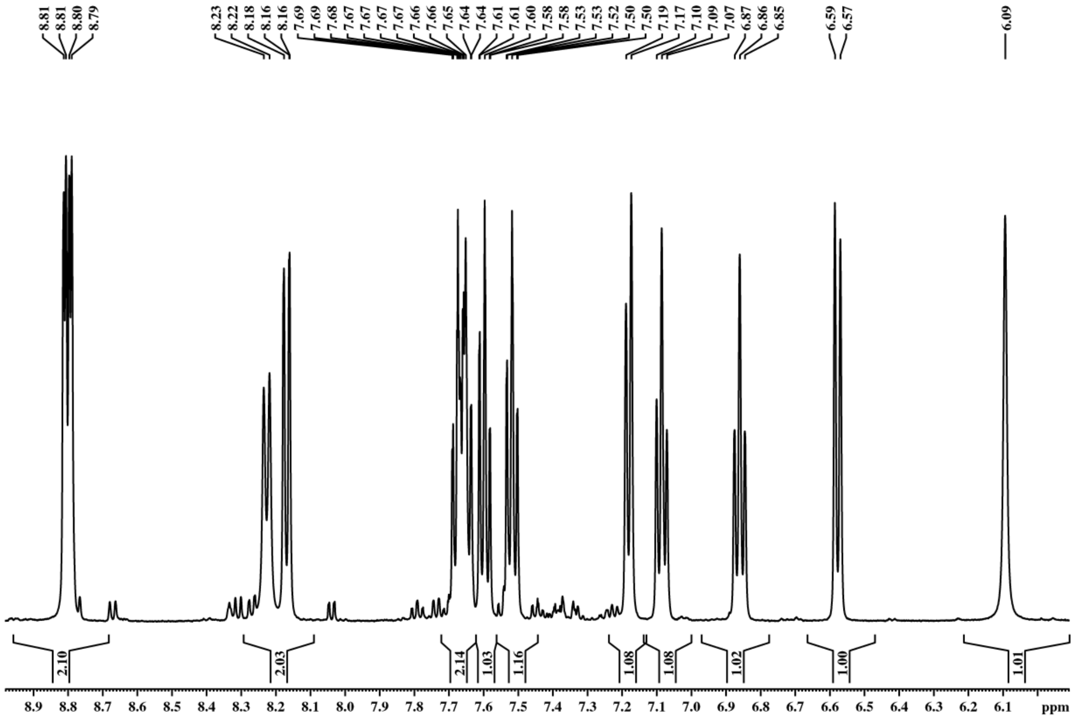

Figure S2. ^{1}H NMR spectrum of **3**, expansion

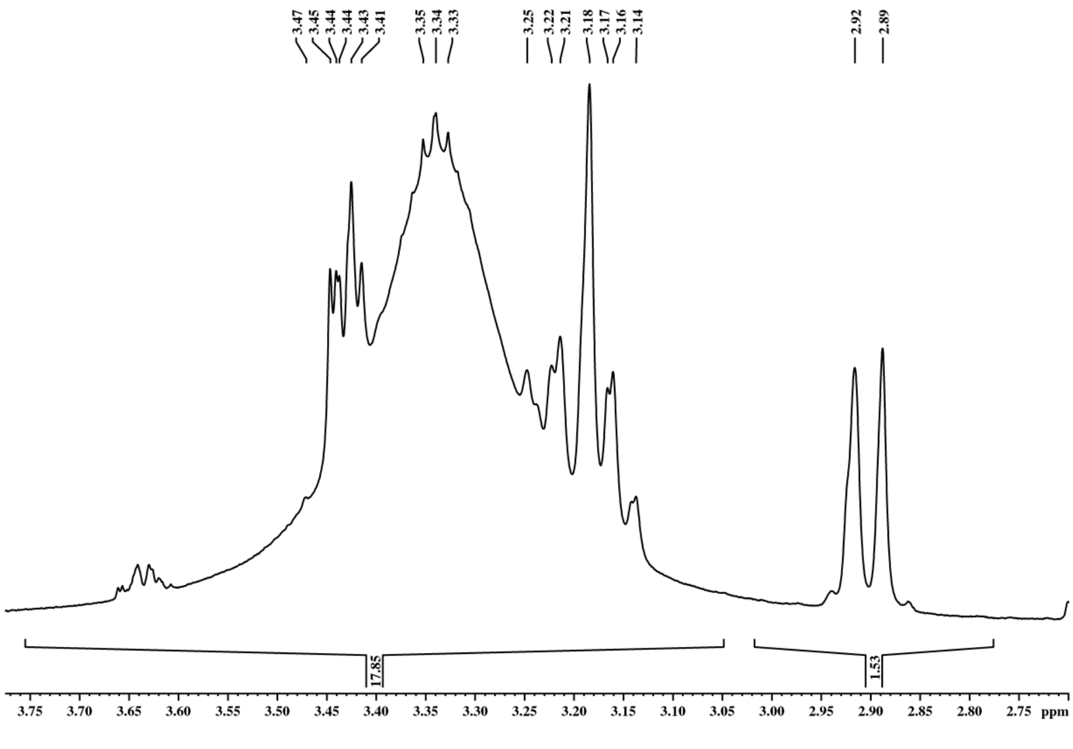

Figure S3. ^{1}H NMR spectrum of **3**, expansion

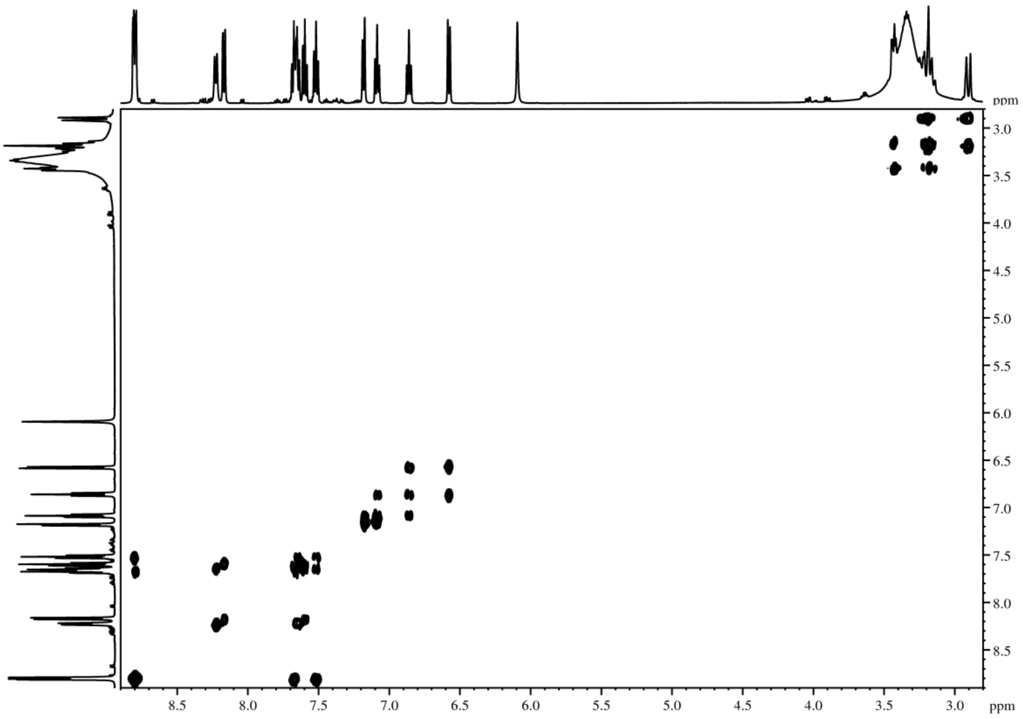

Figure S4. H,H-COSY NMR spectrum of **3**

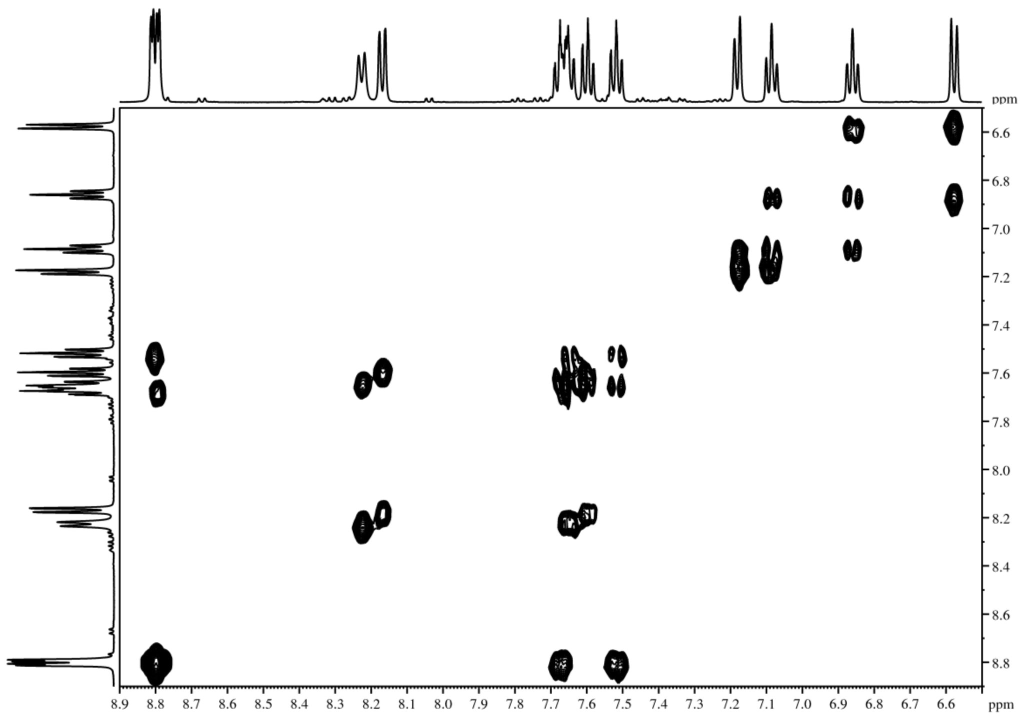

Figure S5. H,H-COSY NMR spectrum of **3**, expansion

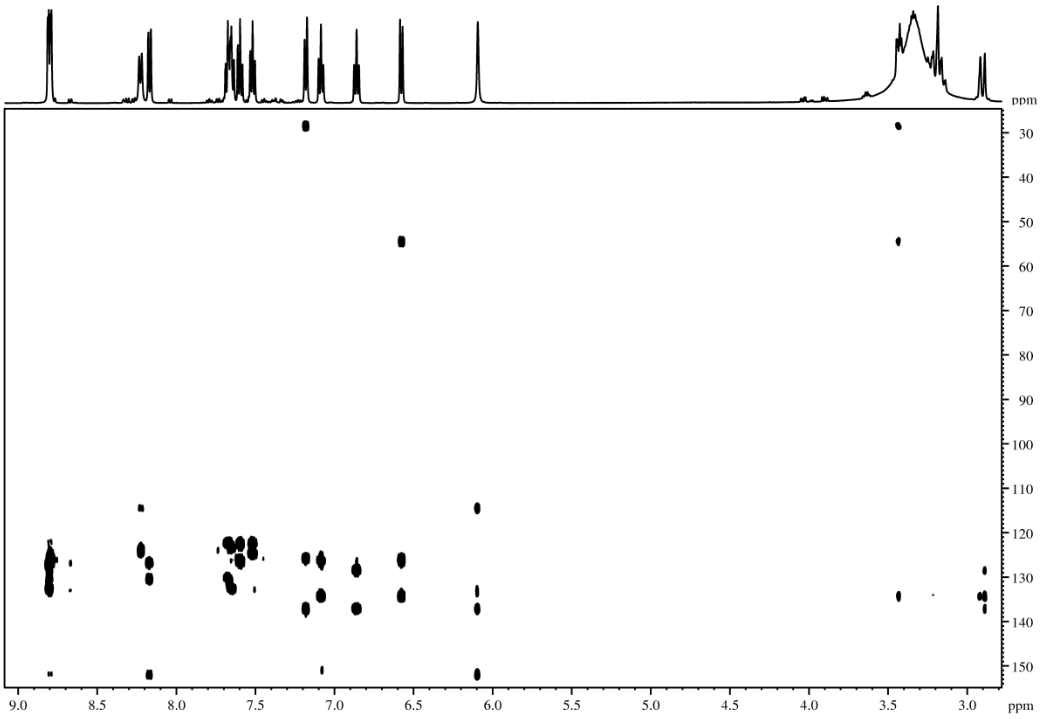

Figure S6. HMBC NMR spectrum of **3**

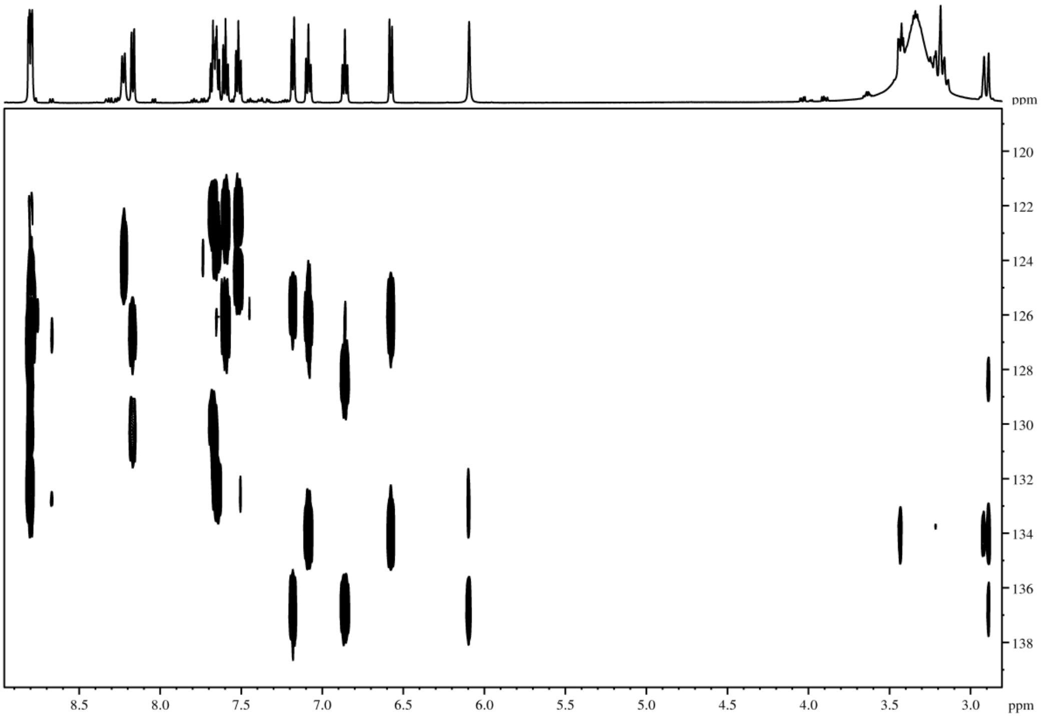

Figure S7. HMBC NMR spectrum of **3**, expansion

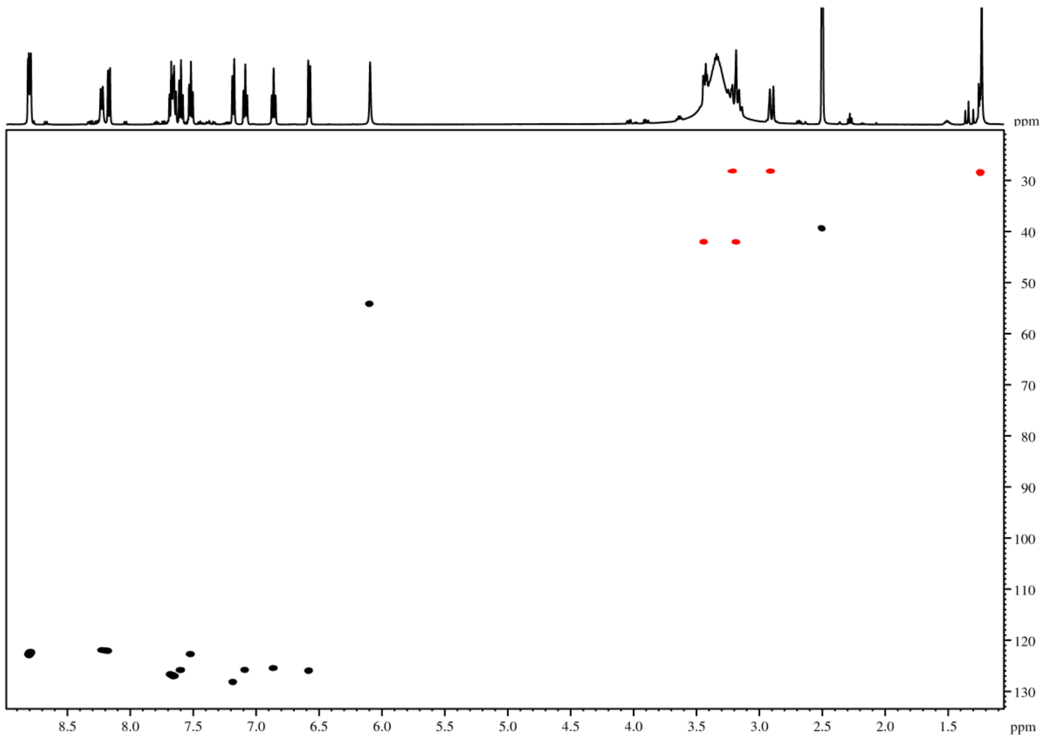

Figure S8. edited HSQC NMR spectrum of **3** (black: positive, red: negative signals)

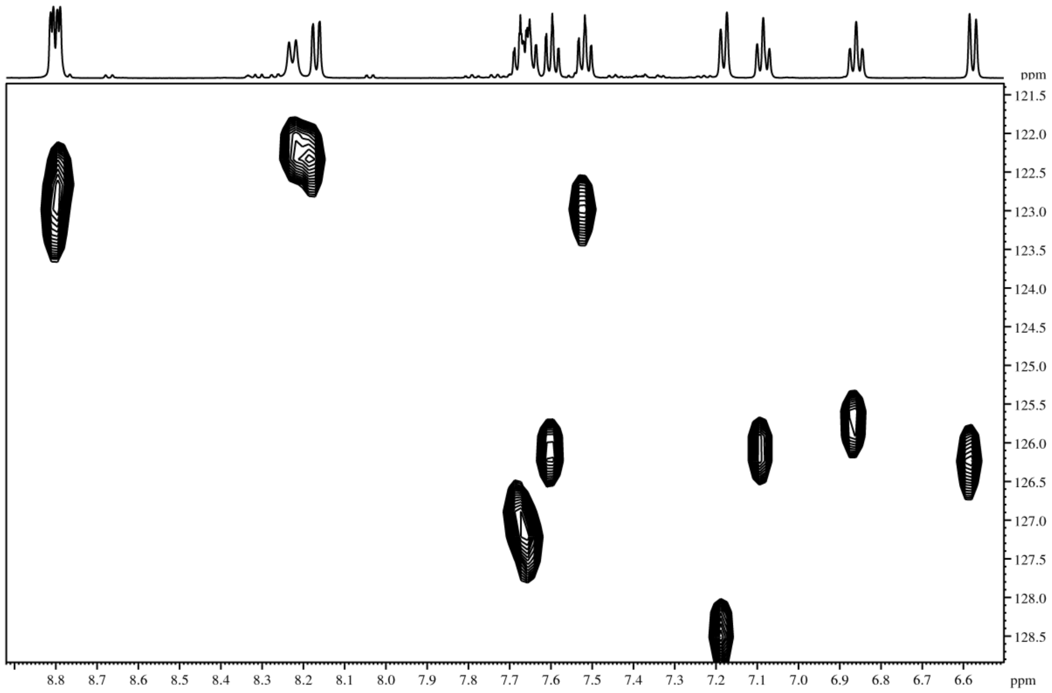

Figure S9. edited HSQC NMR spectrum of **3**, expansion (black: positive, red: negative signals)

4-(9-Hydroxyphenanthr-10-yl)-4,5,6,7-tetrahydrothieno[3,2-c]pyridine (5).

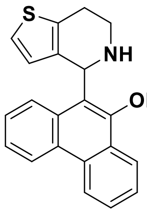

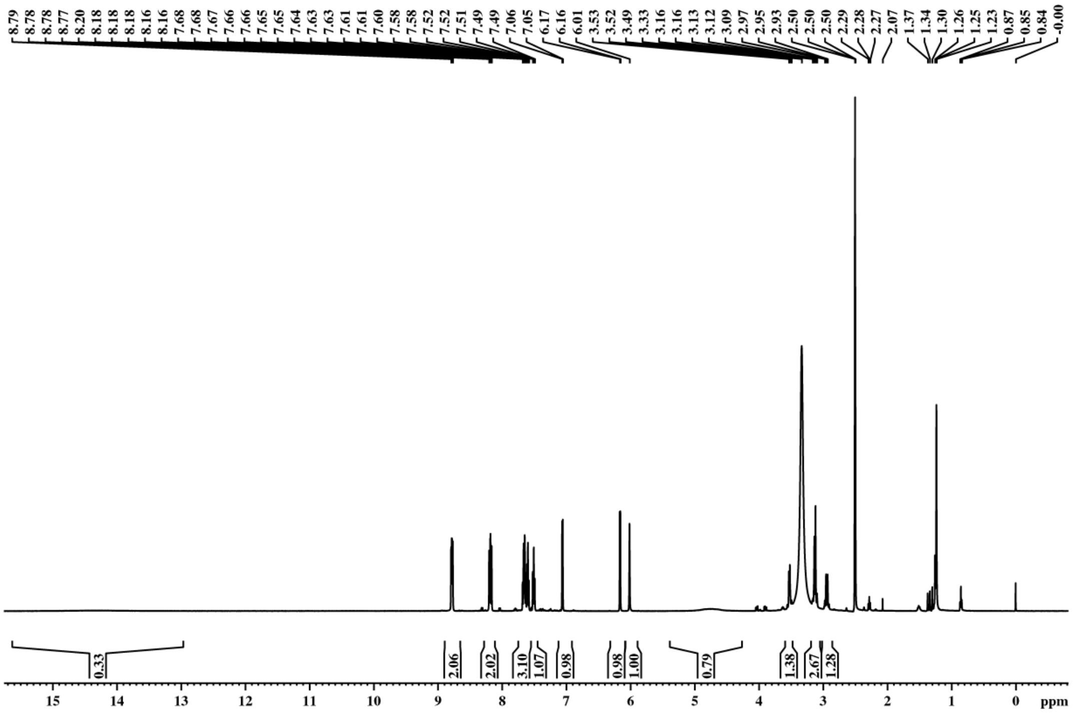

Figure S10. ^{1}H NMR spectrum of **5**

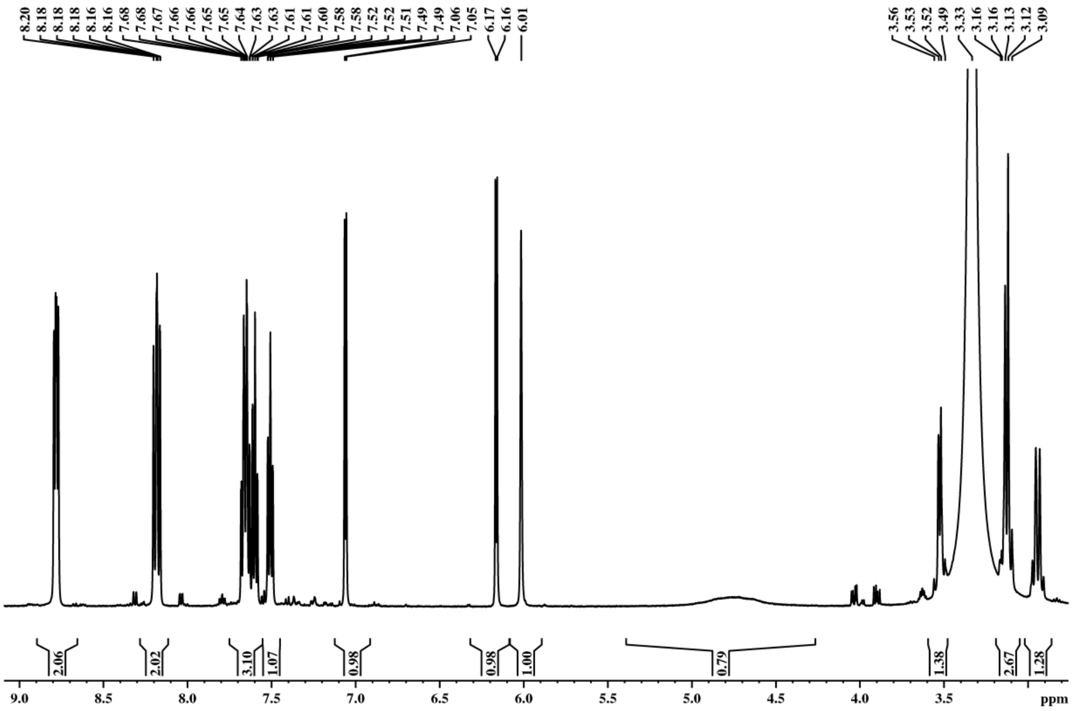

Figure S11. ^{1}H NMR spectrum of **5**, expansion

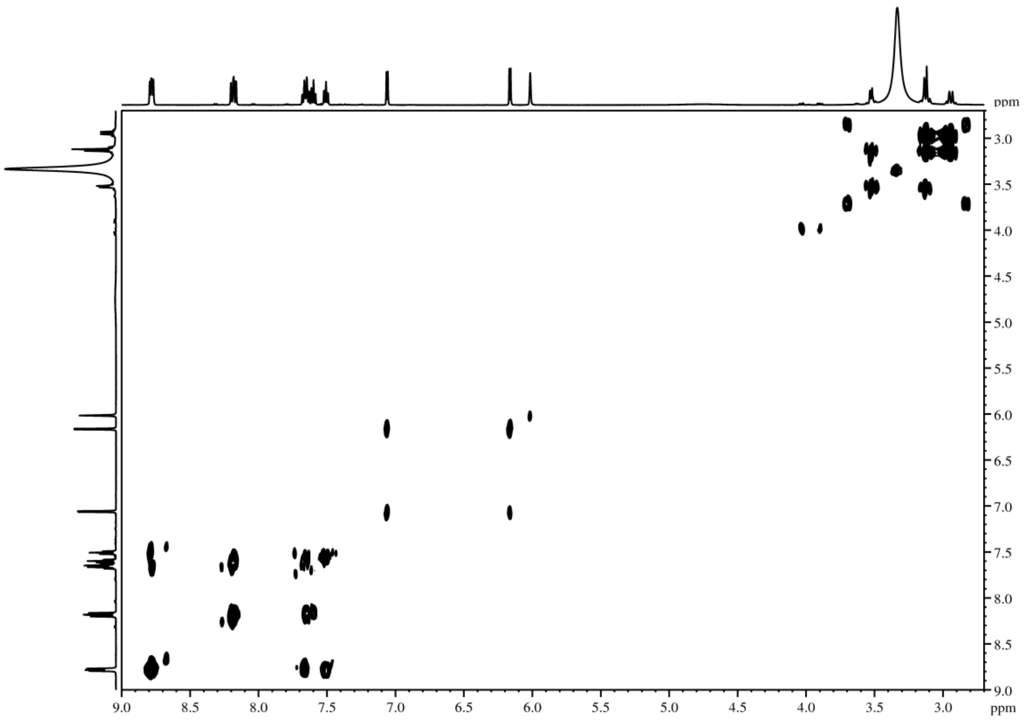

Figure S12. H,H-COSY NMR spectrum of **5**

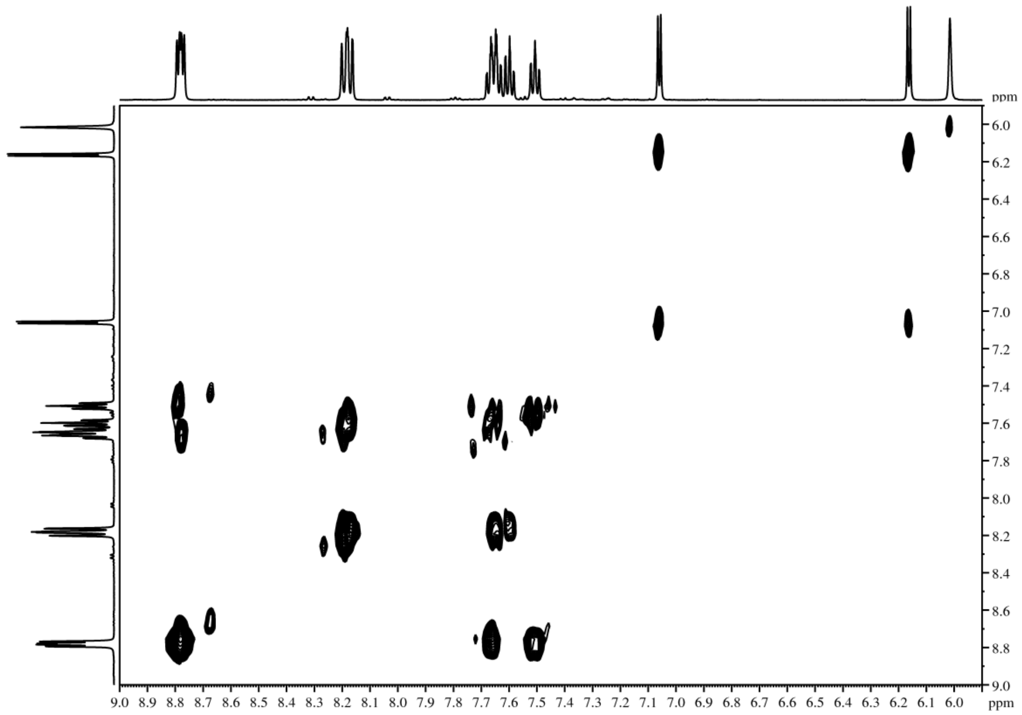

Figure S13. H,H-COSY NMR spectrum of **5**, expansion

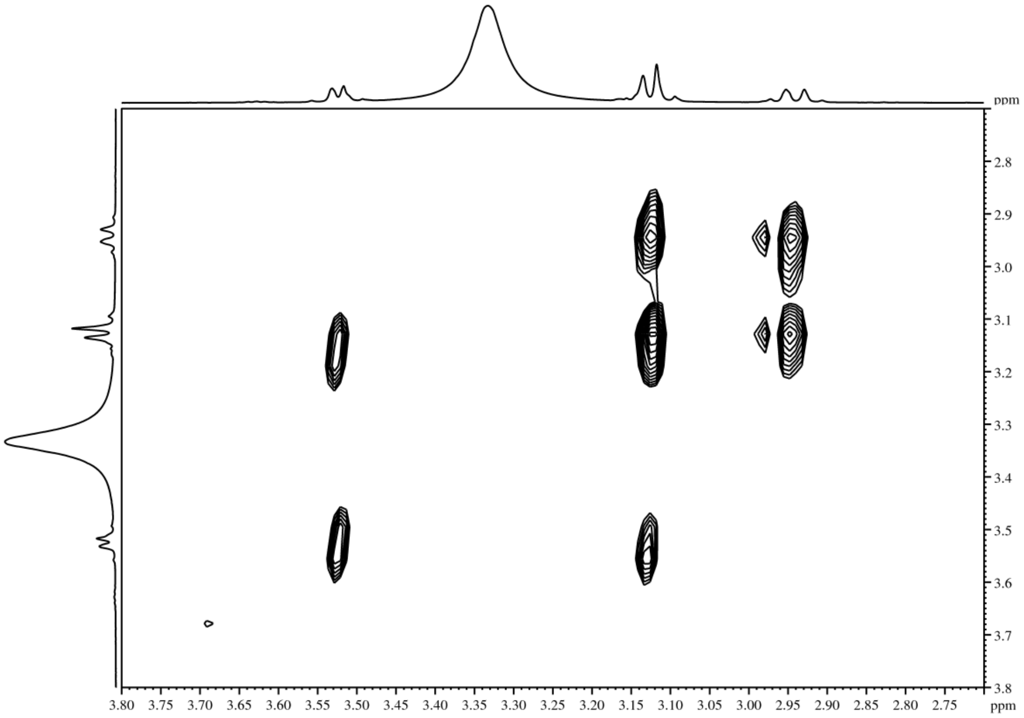

Figure S14. H,H-COSY NMR spectrum of **5**, expansion

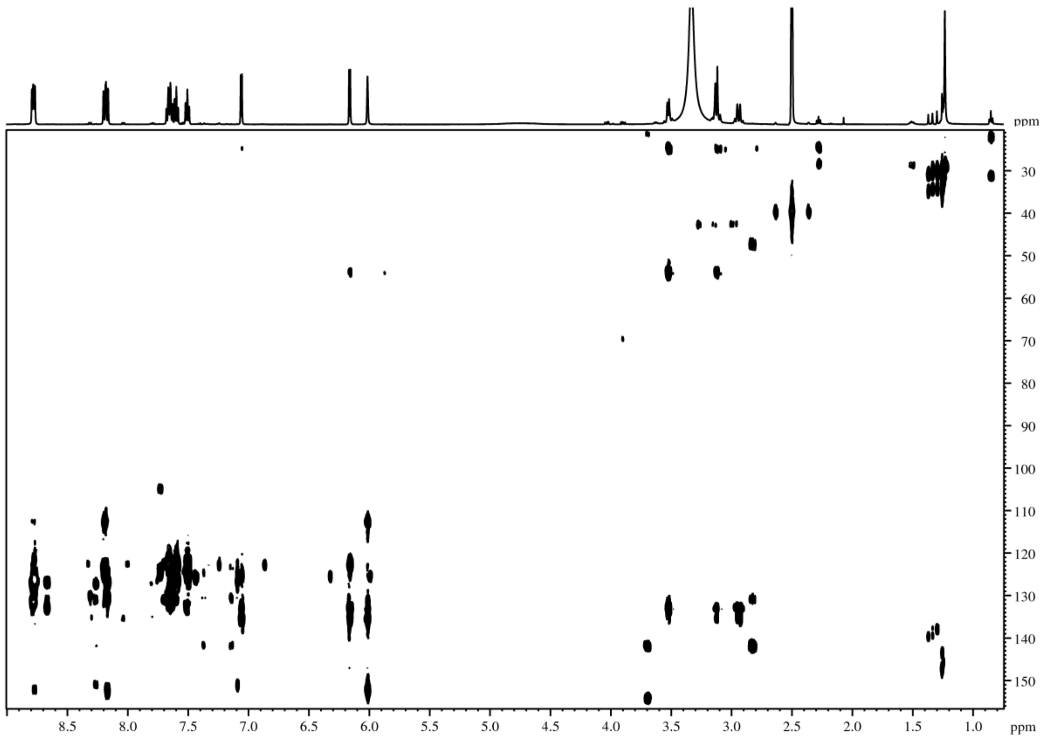

Figure S15. HMBC NMR spectrum of **5**

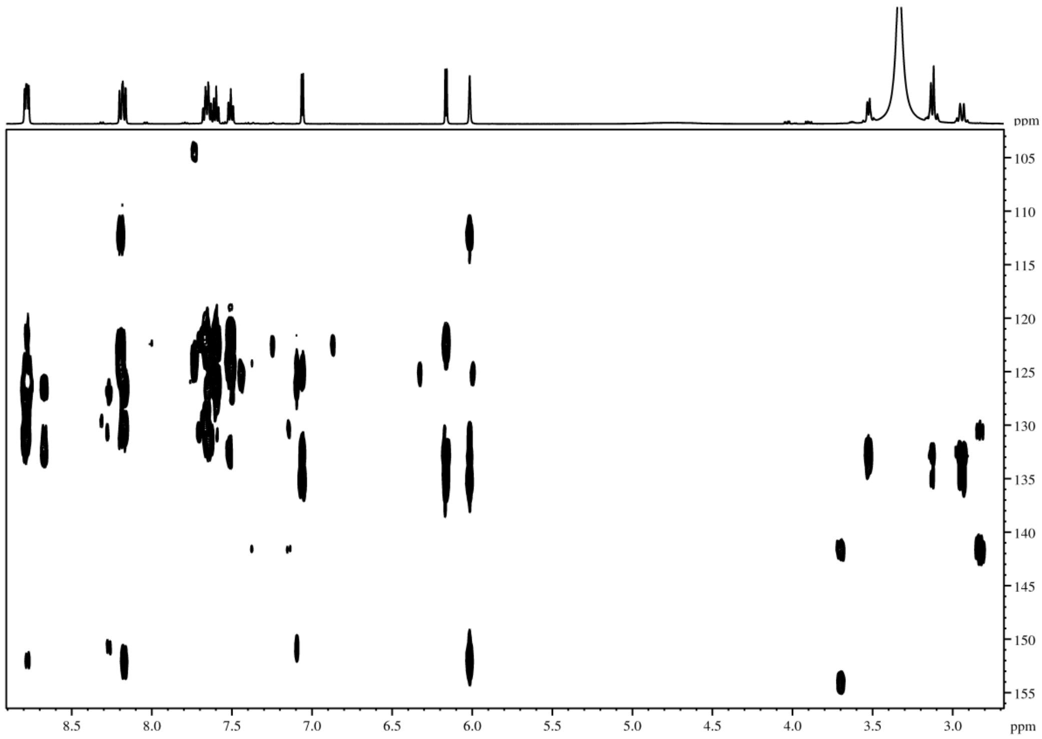

Figure S16. HMBC NMR spectrum of **5**, expansion

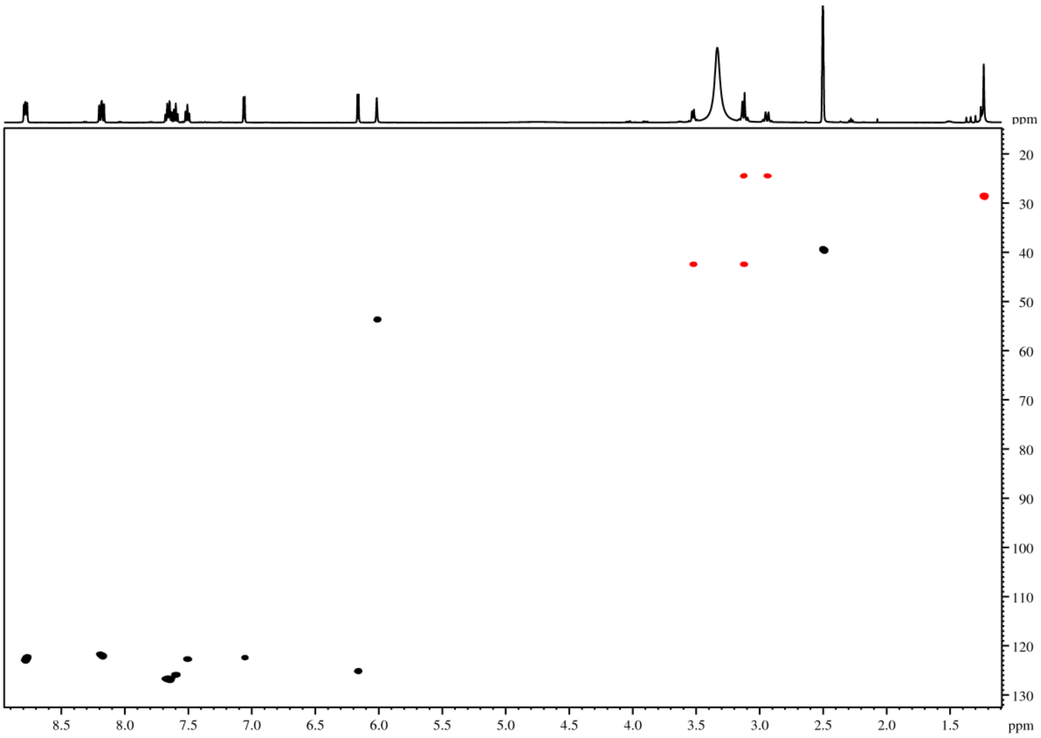

Figure S17. edited HSQC NMR spectrum of **5** (black: positive, red: negative signals)

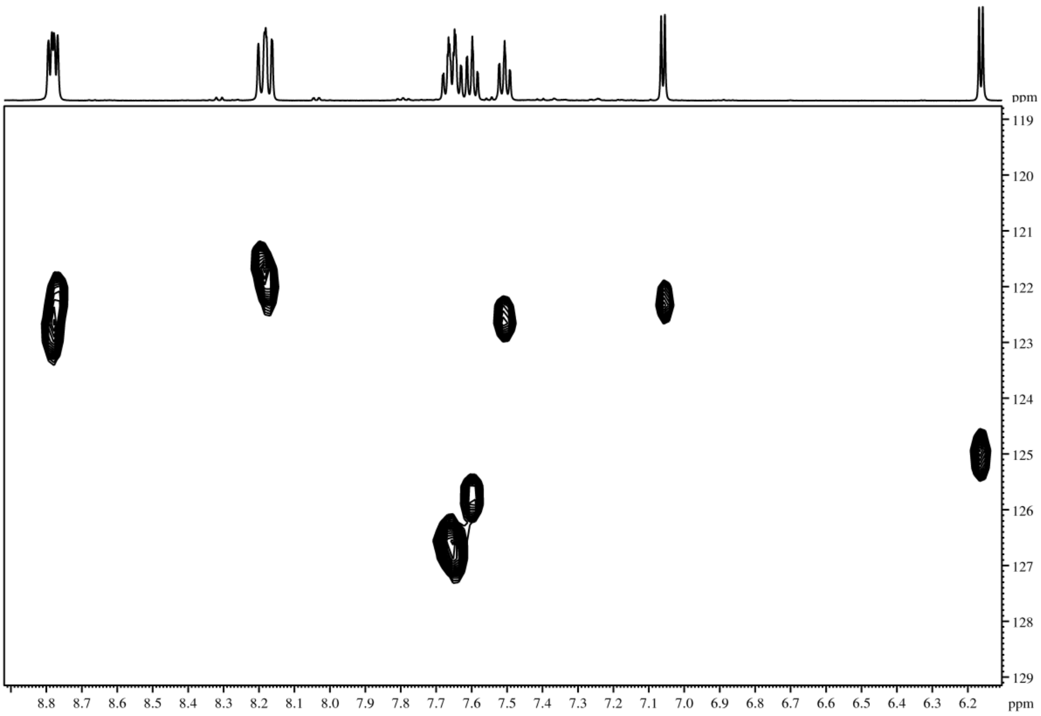

Figure S18. edited HSQC NMR spectrum of **5**, expansion (black: positive, red: negative signals)

1-(9-Hydroxyphenanthr-10-yl)-1,2,3,4-tetrahydro-β-carboline (7)

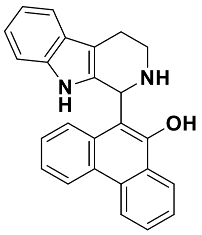

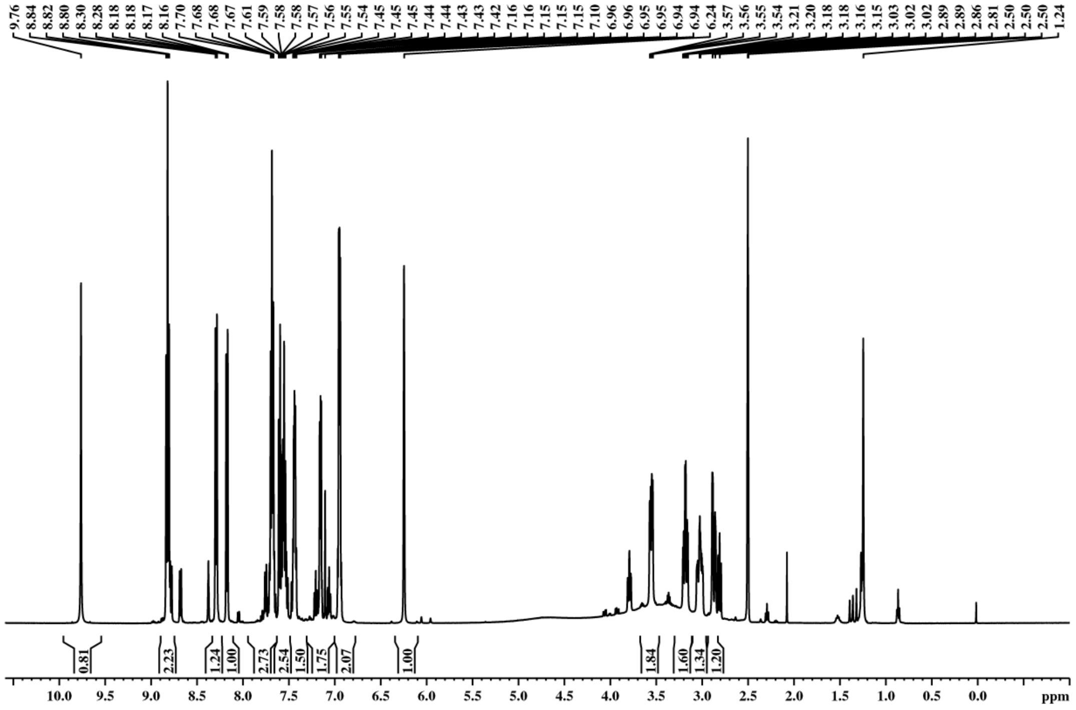

Figure S19. ^{1}H NMR spectrum of **7**

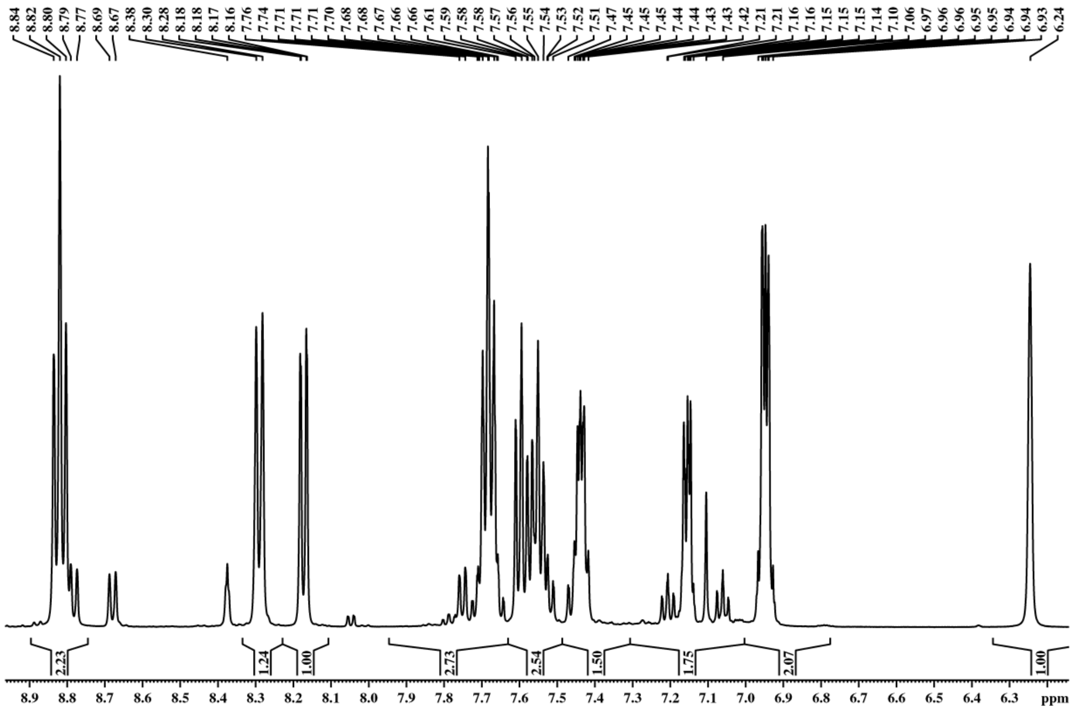

Figure S20. ^{1}H NMR spectrum of **7**, expansion

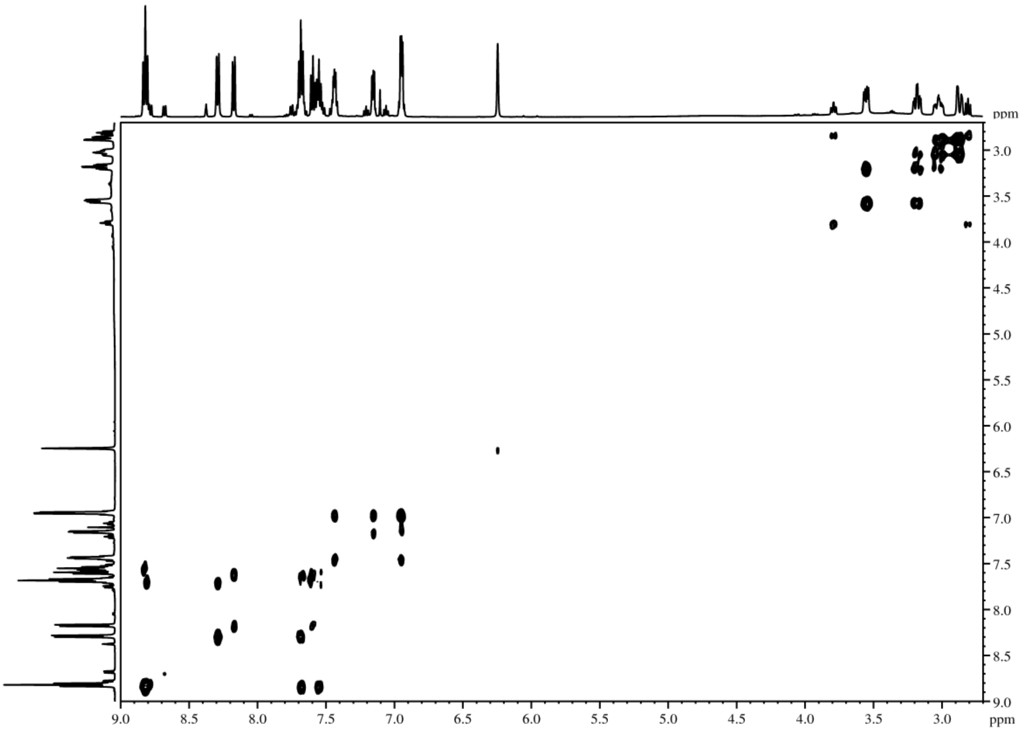

Figure S20. H,H-COSY NMR spectrum of **7**

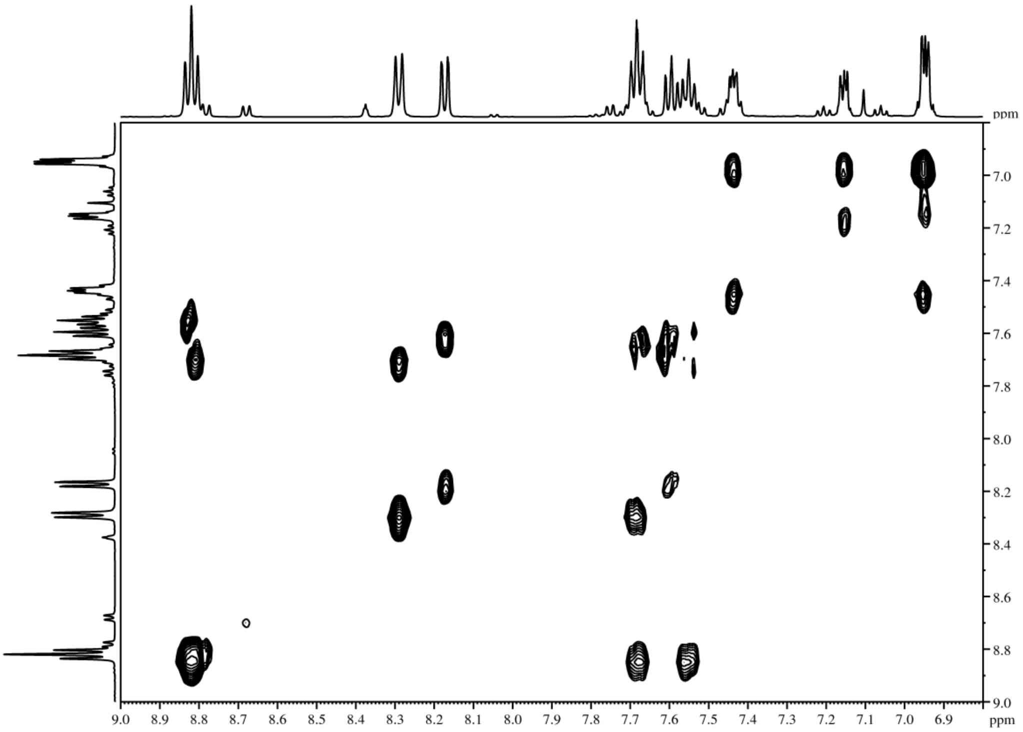

Figure S21. H,H-COSY NMR spectrum of **7**, expansion

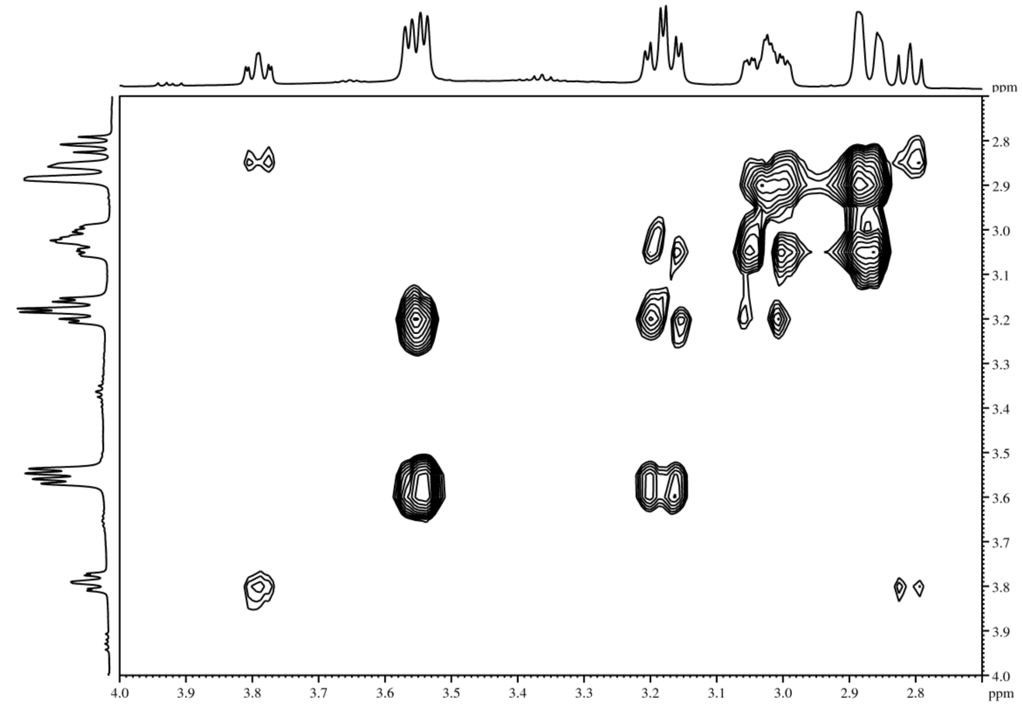

Figure S22. H,H-COSY NMR spectrum of **7**, expansion

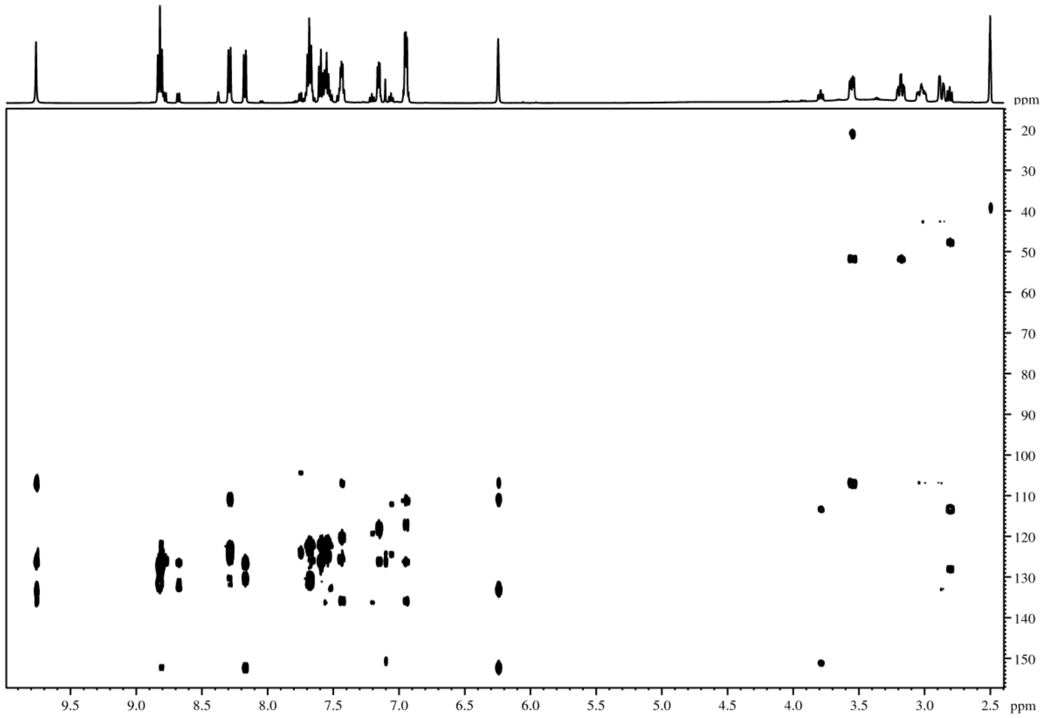

Figure S23. HMBC NMR spectrum of **7**

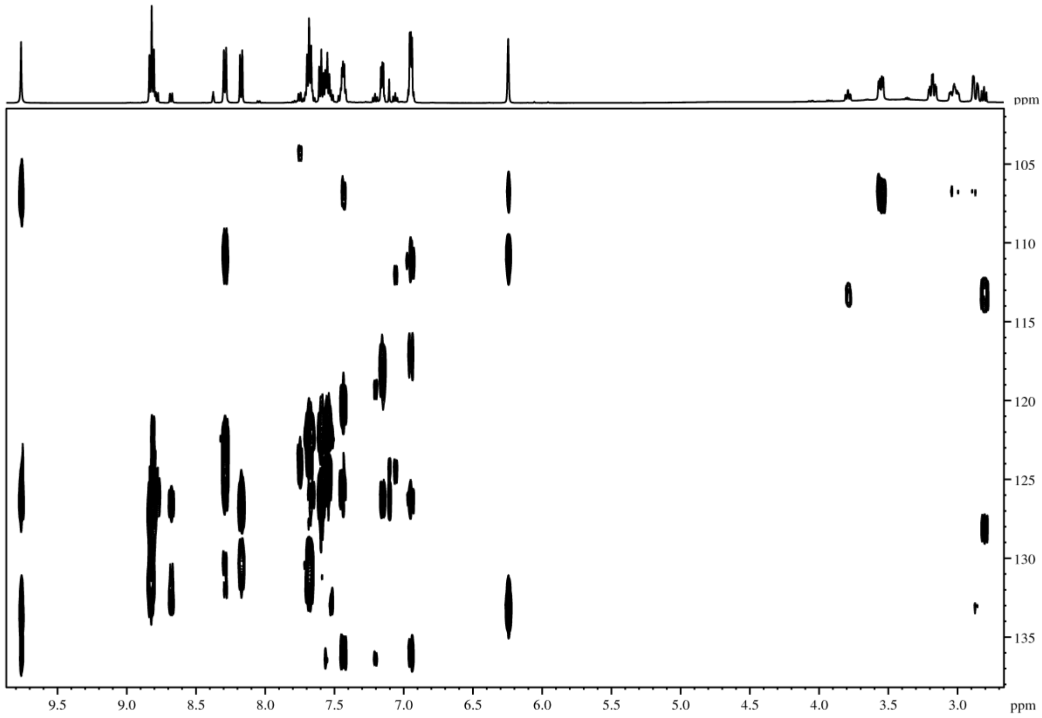

Figure S24. HMBC NMR spectrum of **7**, expansion

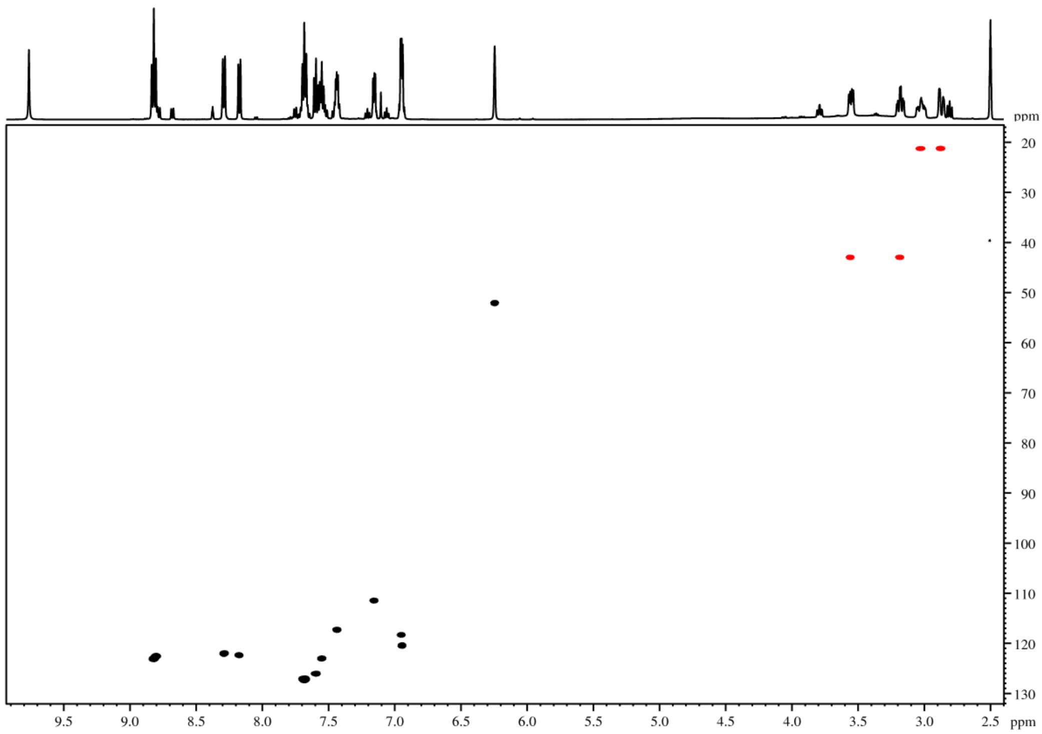

Figure S25. edited HSQC NMR spectrum of **7** (black: positive, red: negative signals)

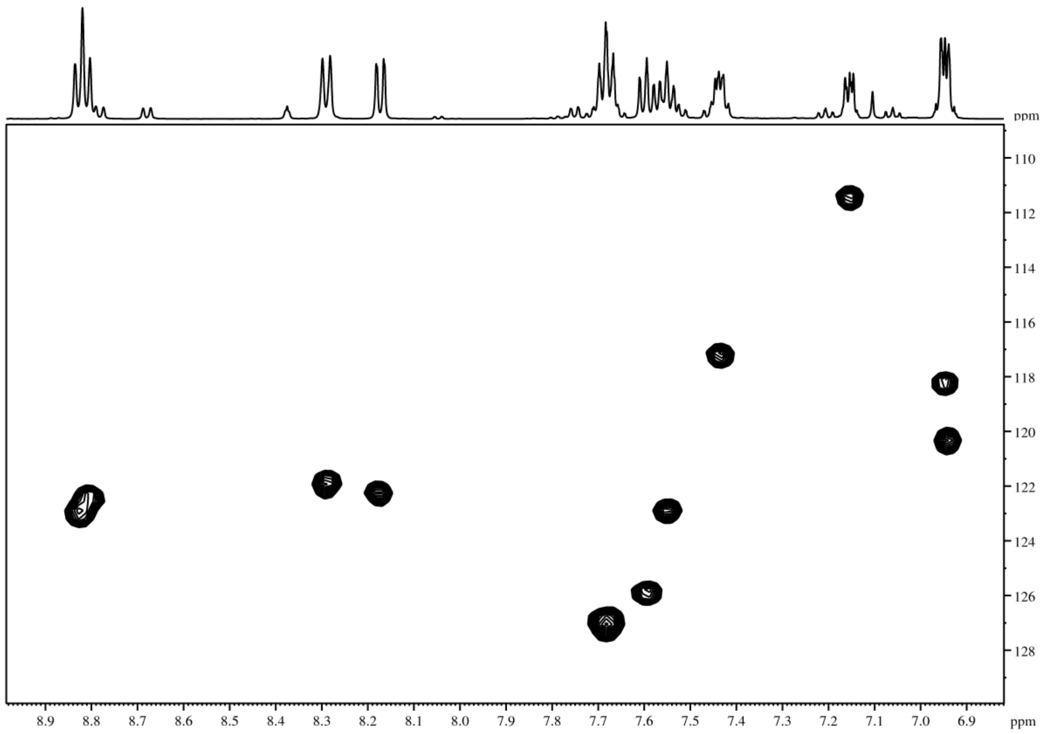

Figure S26. edited HSQC NMR spectrum of **7**, expansion (black: positive, red: negative signals)

Phenanthr[9,10-e][1,3]oxazino[4,3-a]isoquinoline (8)

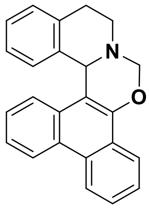

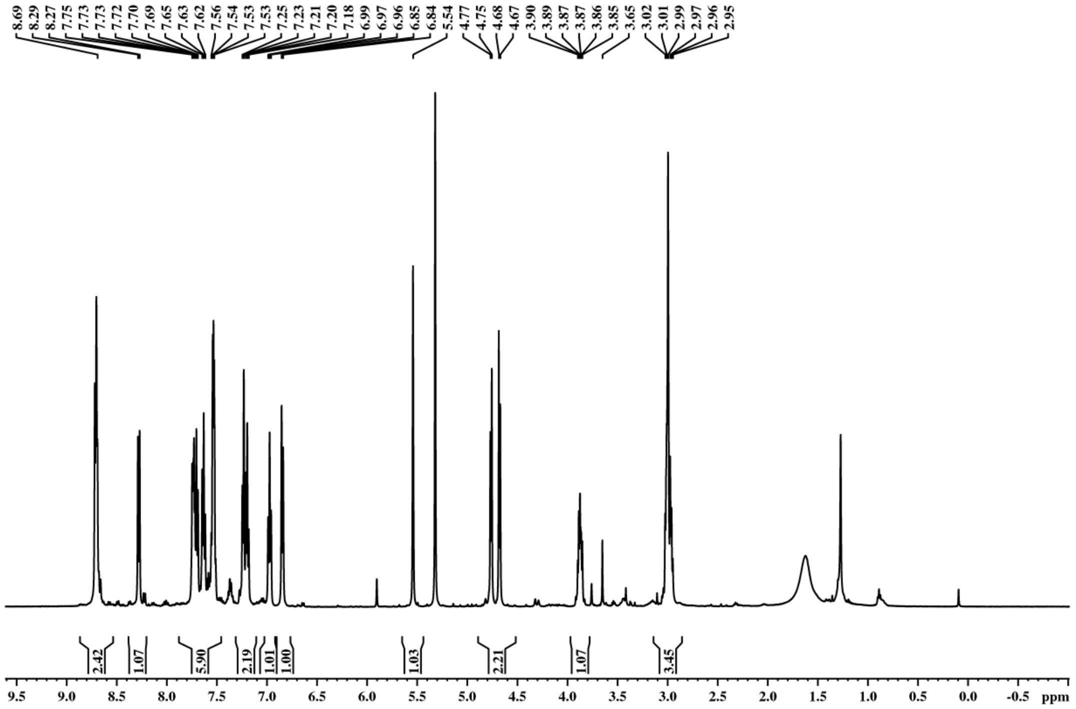

Figure S27. ^{1}H NMR spectrum of **8**

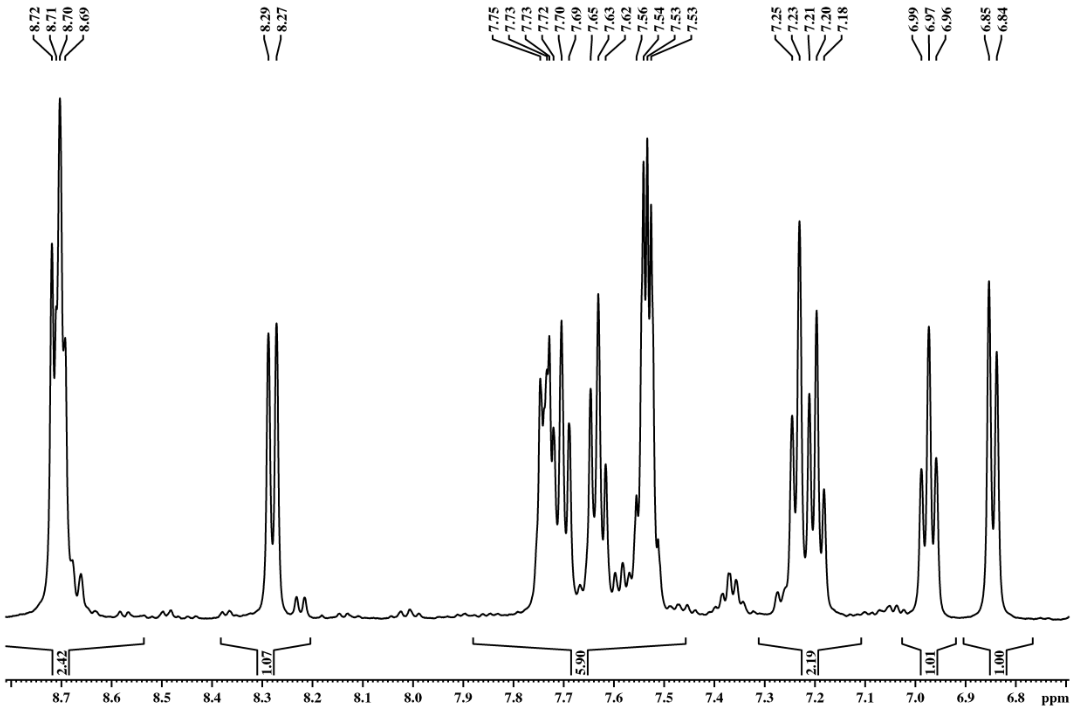

Figure S28. ^{1}H NMR spectrum of **8**, expansion

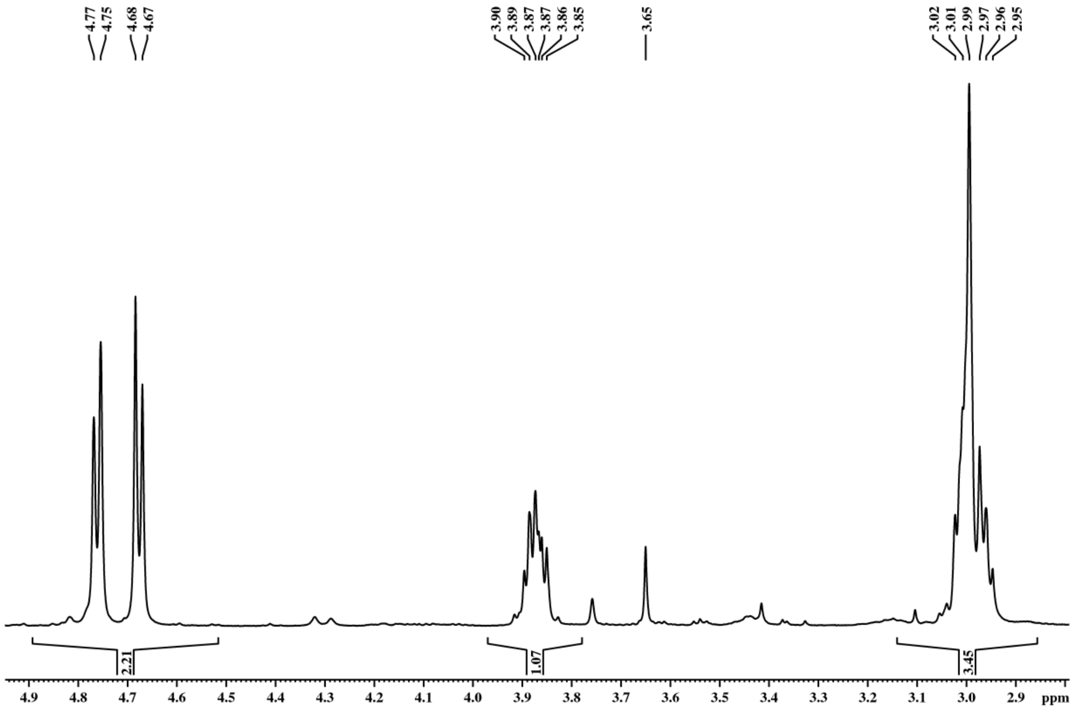

Figure S29. ^{1}H NMR spectrum of **8**, expansion

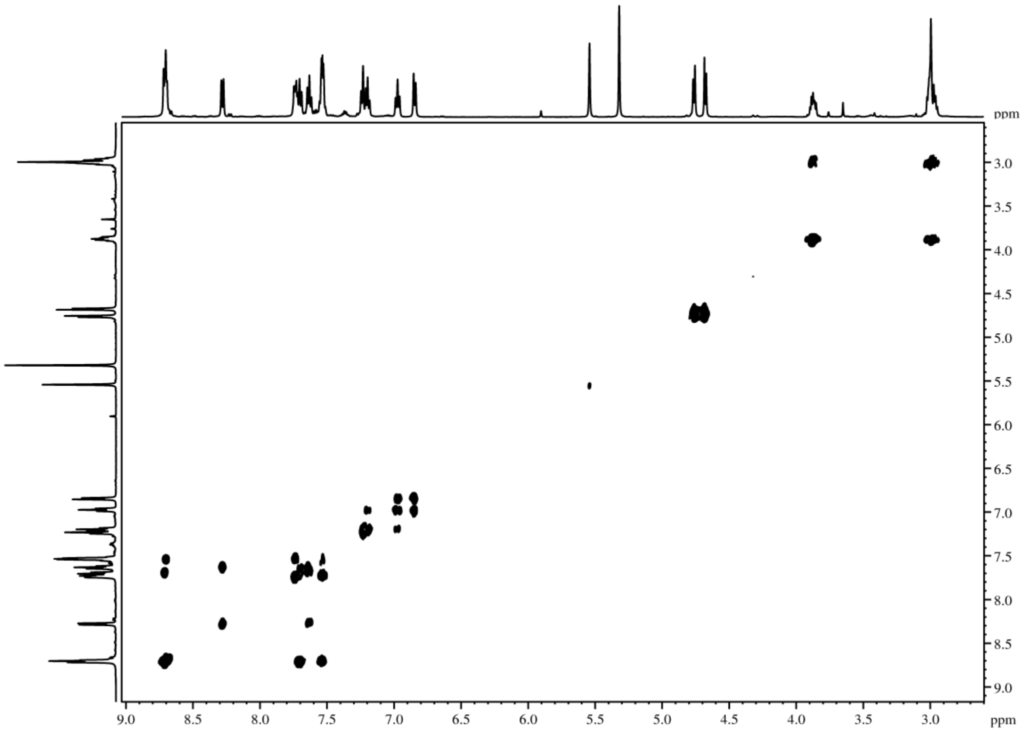

Figure S30. H,H-COSY NMR spectrum of **8**

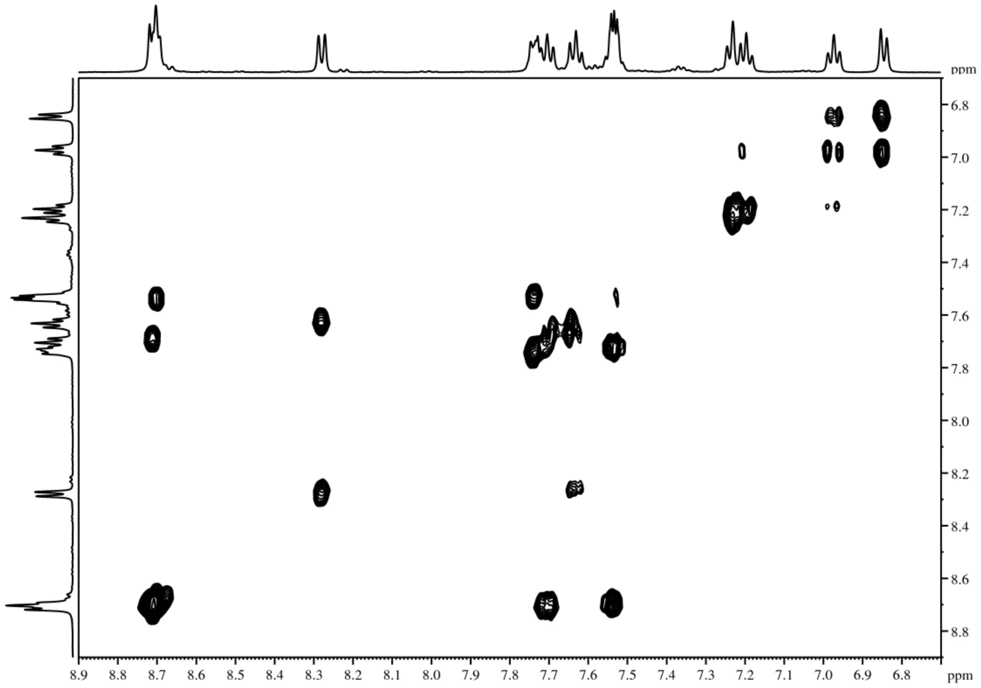

Figure S31. H,H-COSY NMR spectrum of **8**, expansion

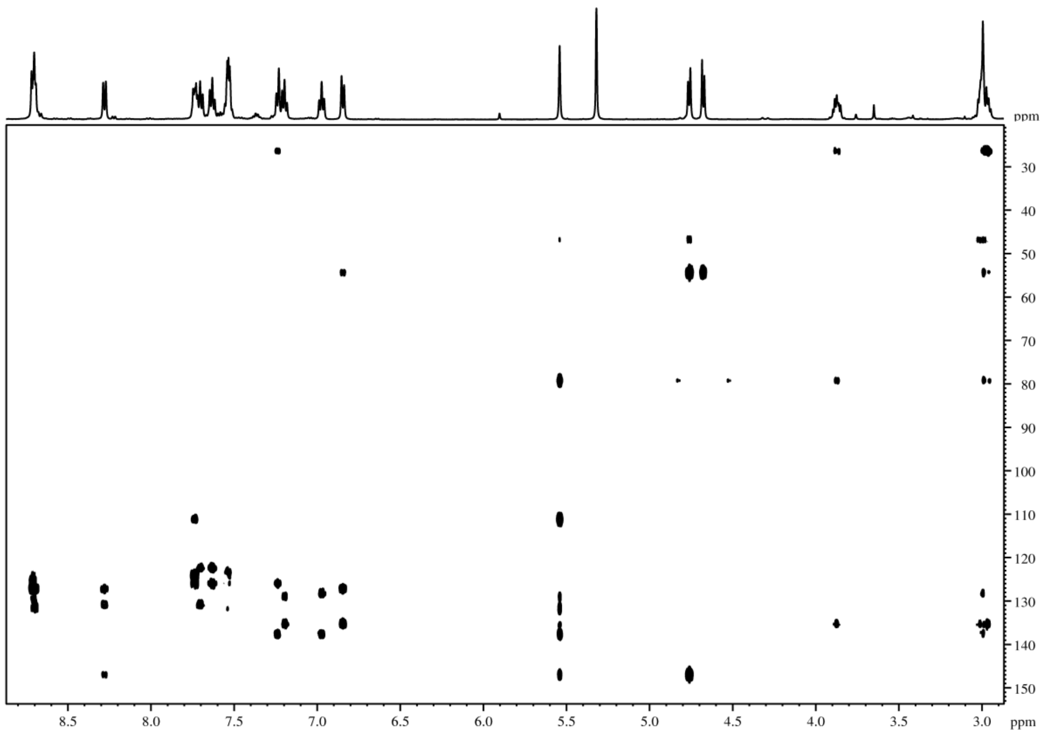

Figure S32. HMBC NMR spectrum of **8**

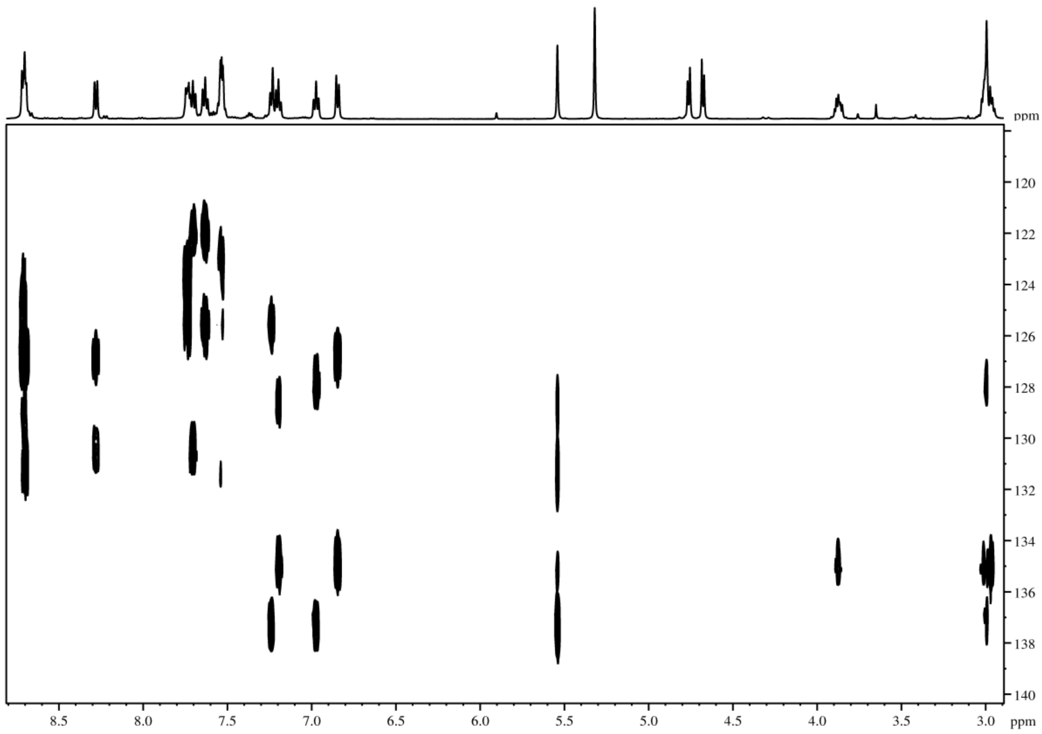

Figure S33. HMBC NMR spectrum of **8**, expansion

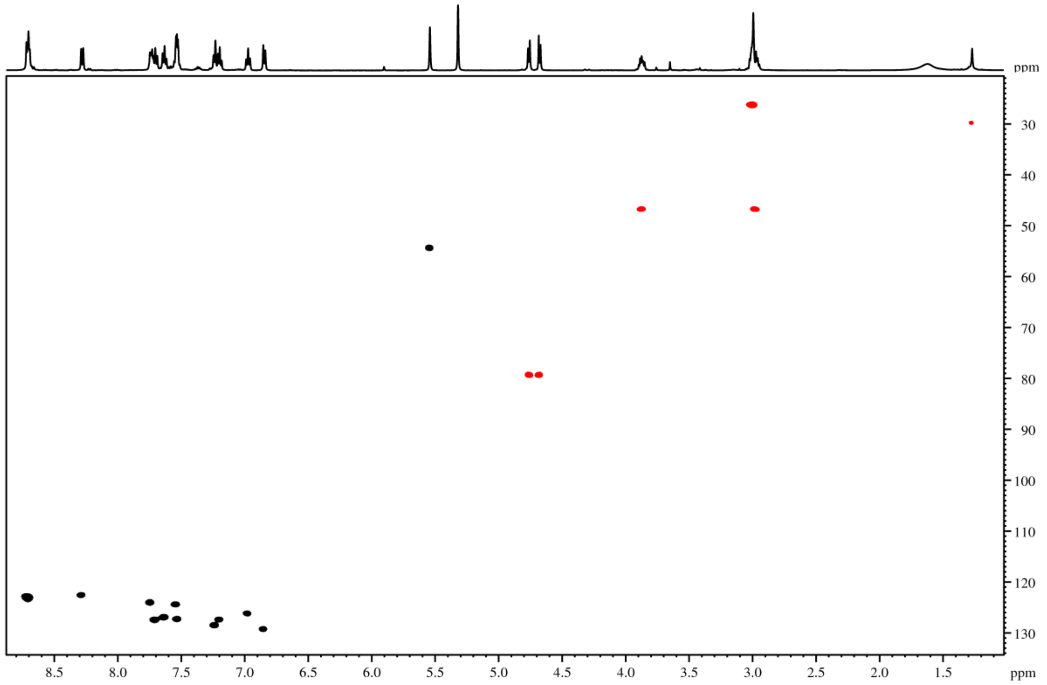

Figure S34. edited HSQC NMR spectrum of **8** (black: positive, red: negative signals)

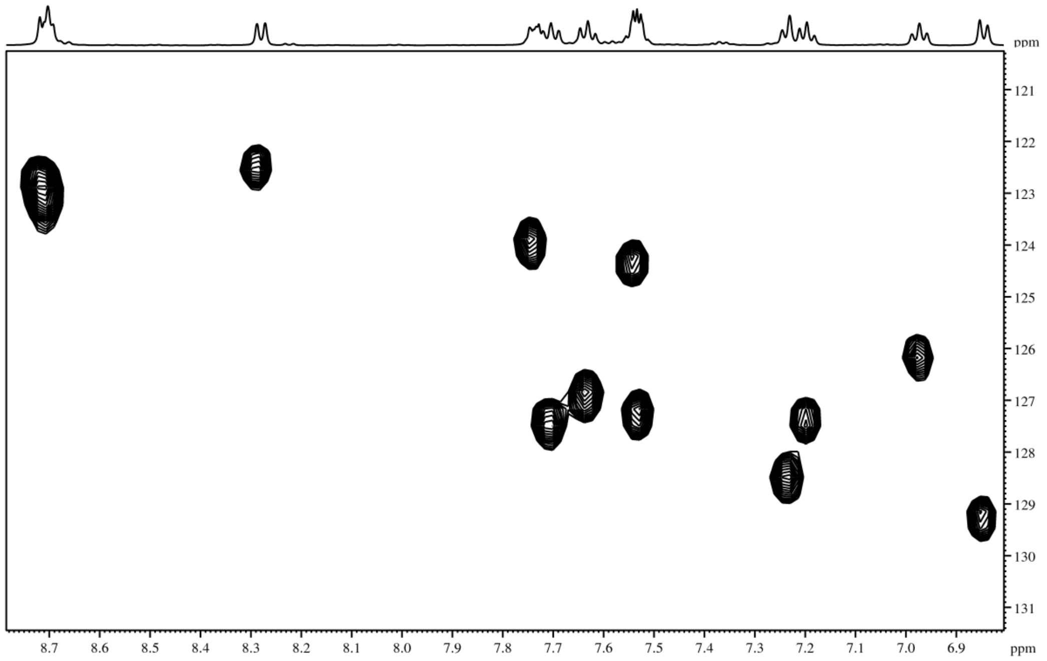

Figure S35. edited HSQC NMR spectrum of **8**, expansion (black: positive, red: negative signals)

Phenanthr[9,10-e][1,3]oxazino[4,3-a]thieno[3,2-c]pyridine (9)

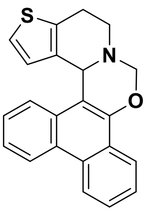

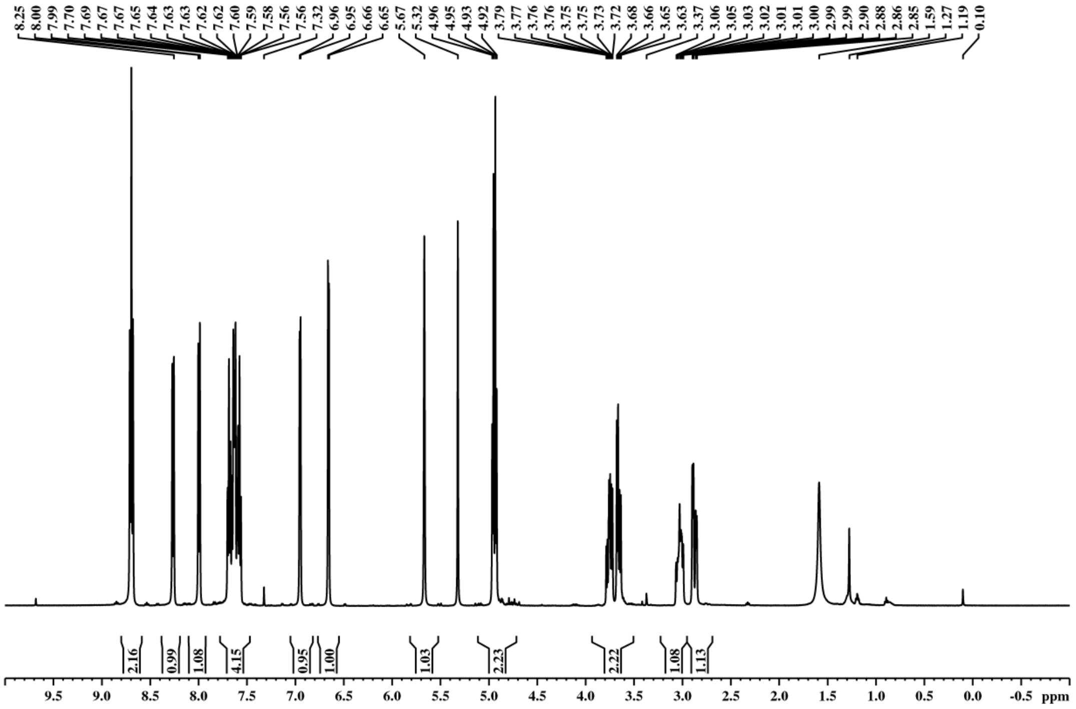

Figure S36. ^{1}H NMR spectrum of **9**

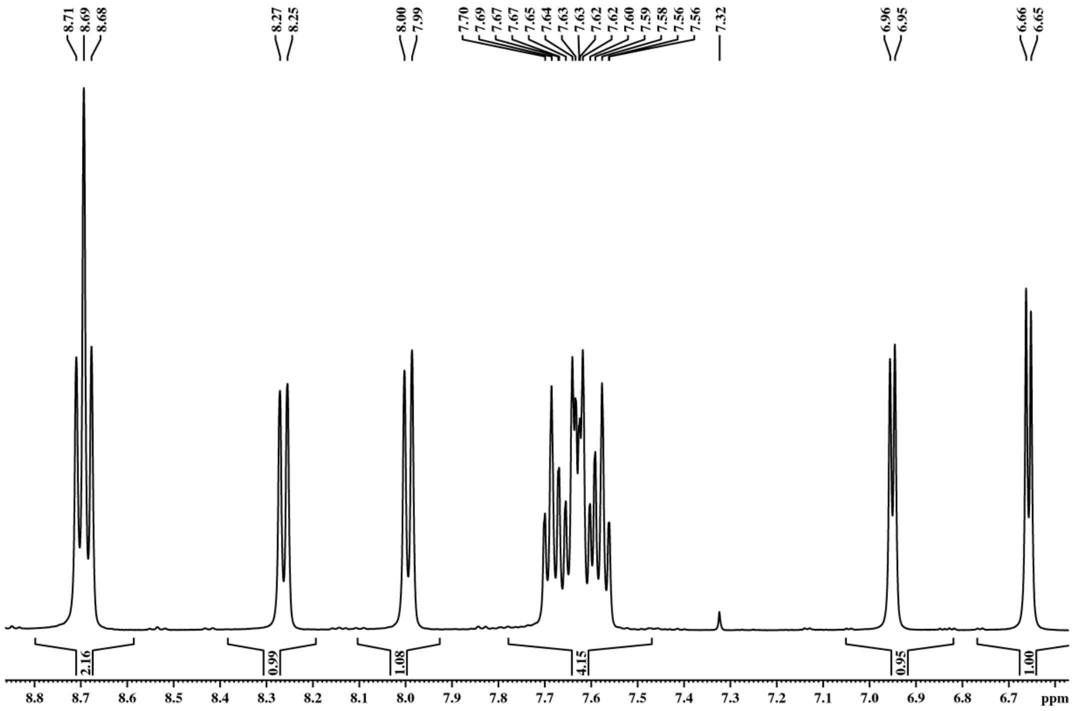

Figure S37. ^{1}H NMR spectrum of **9**, expansion

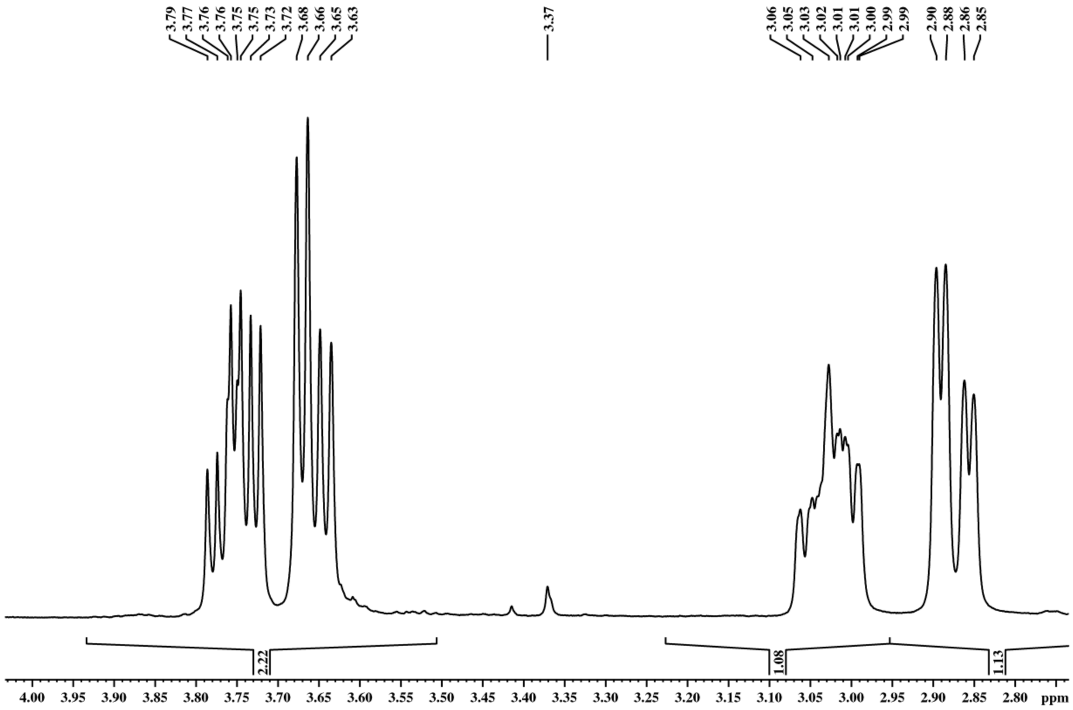

Figure S38. ^{1}H NMR spectrum of **9**, expansion

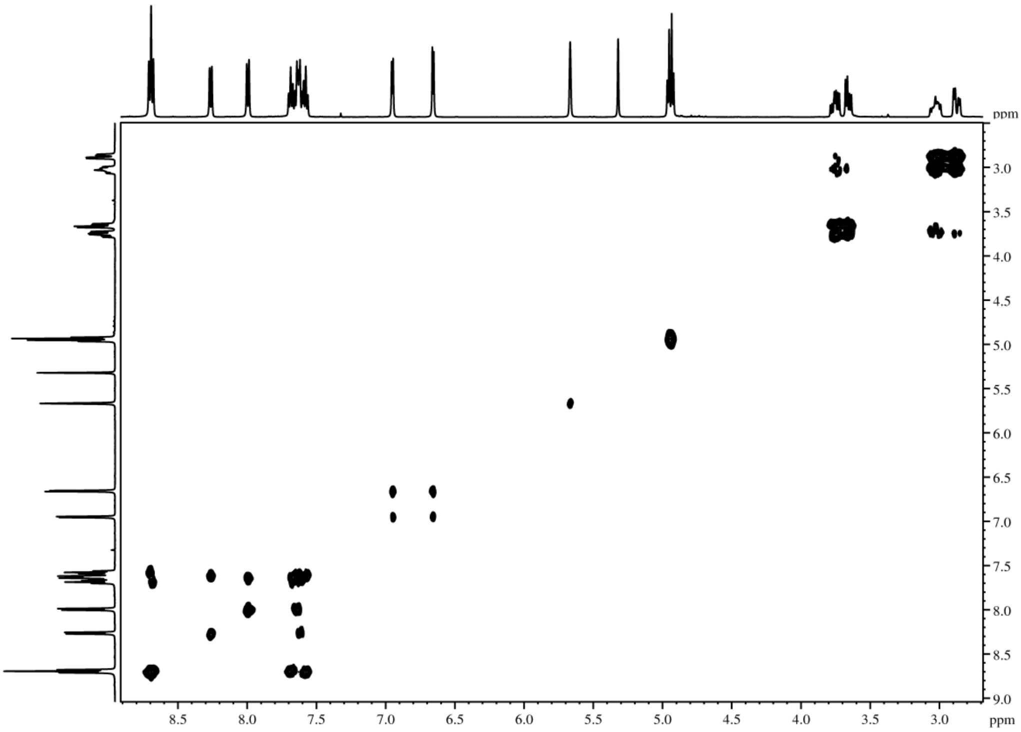

Figure S39. H,H-COSY NMR spectrum of **9**

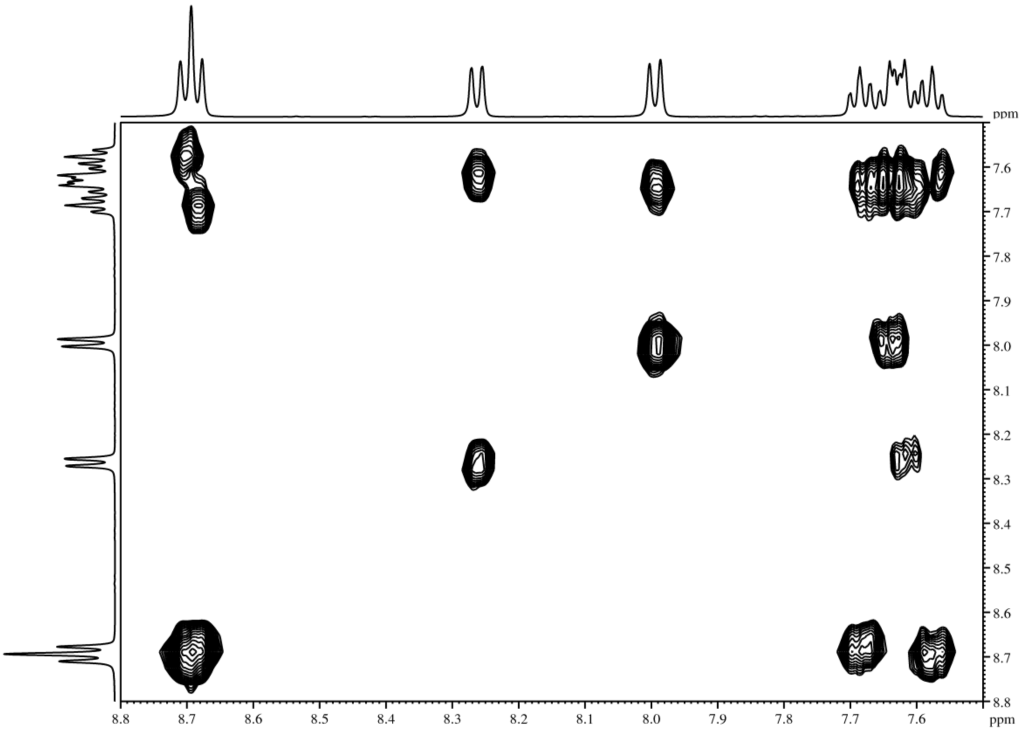

Figure S40. H,H-COSY NMR spectrum of **9**, expansion

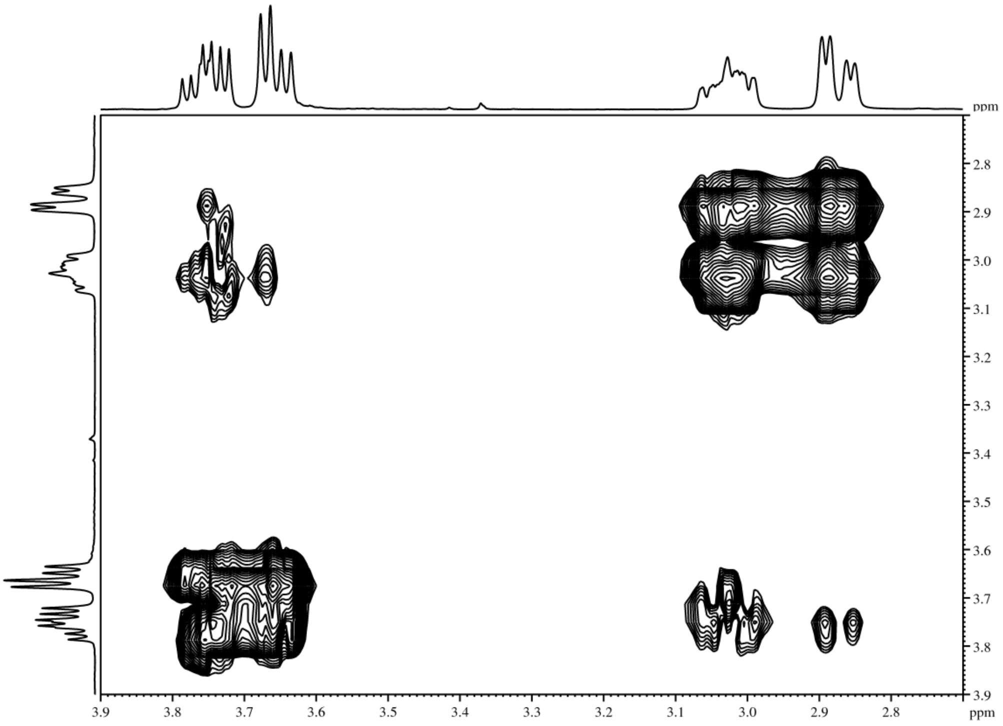

Figure S41. H,H-COSY NMR spectrum of **9**, expansion

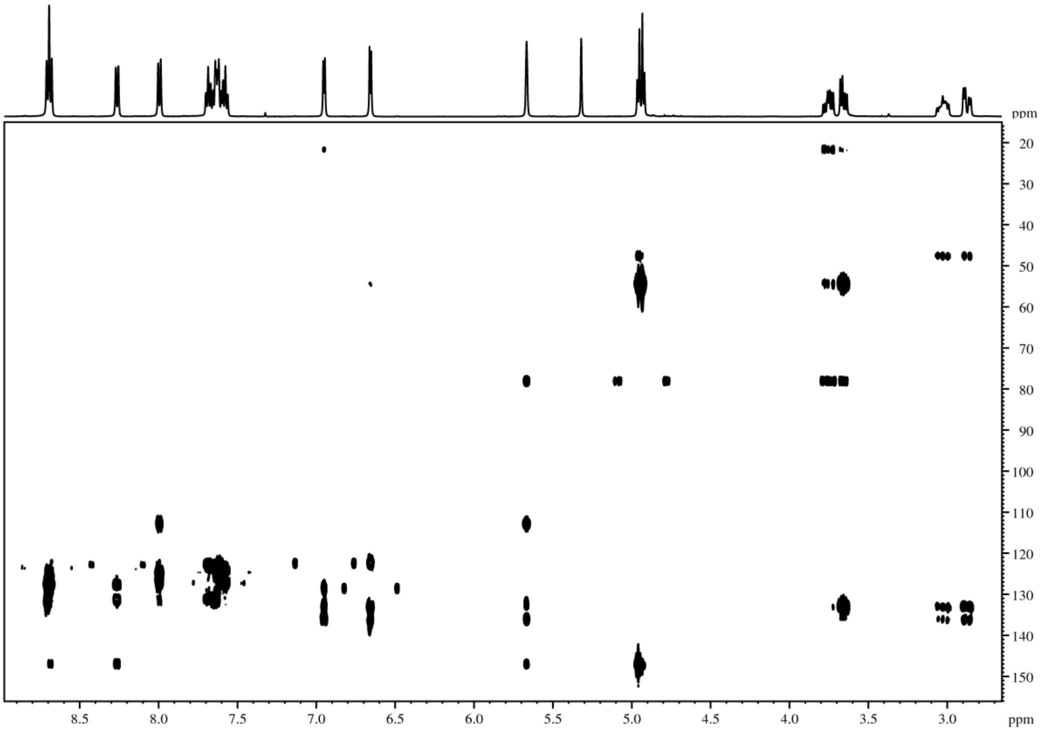

Figure S42. HMBC NMR spectrum of **9**

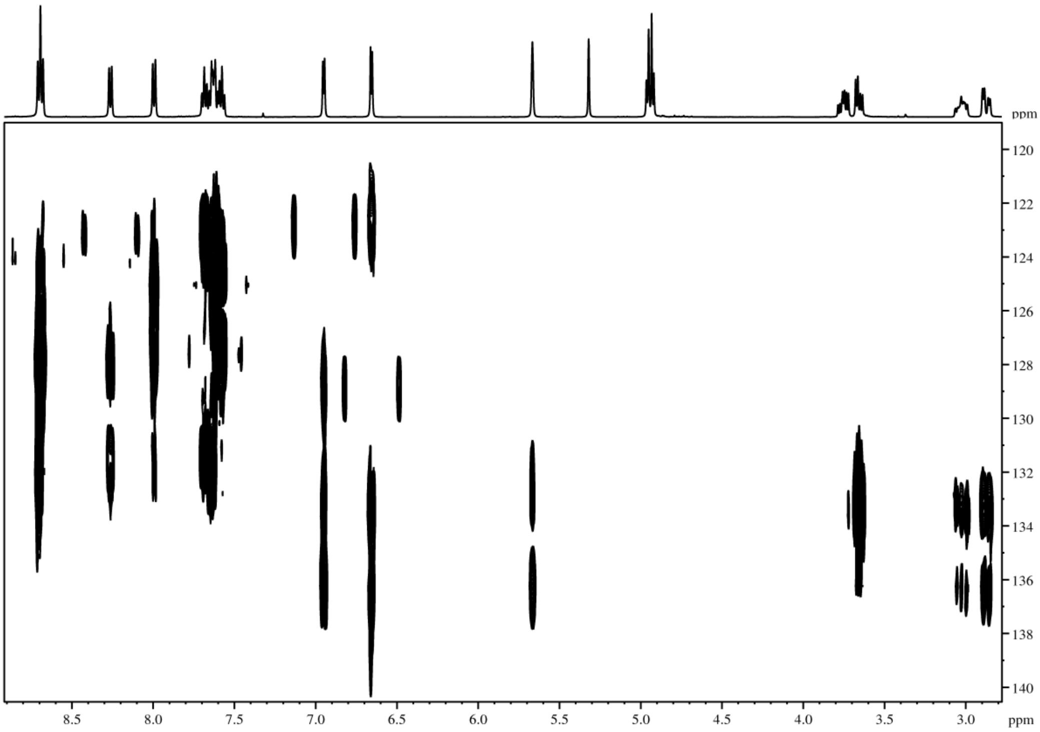

Figure S43. HMBC NMR spectrum of **9**, expansion

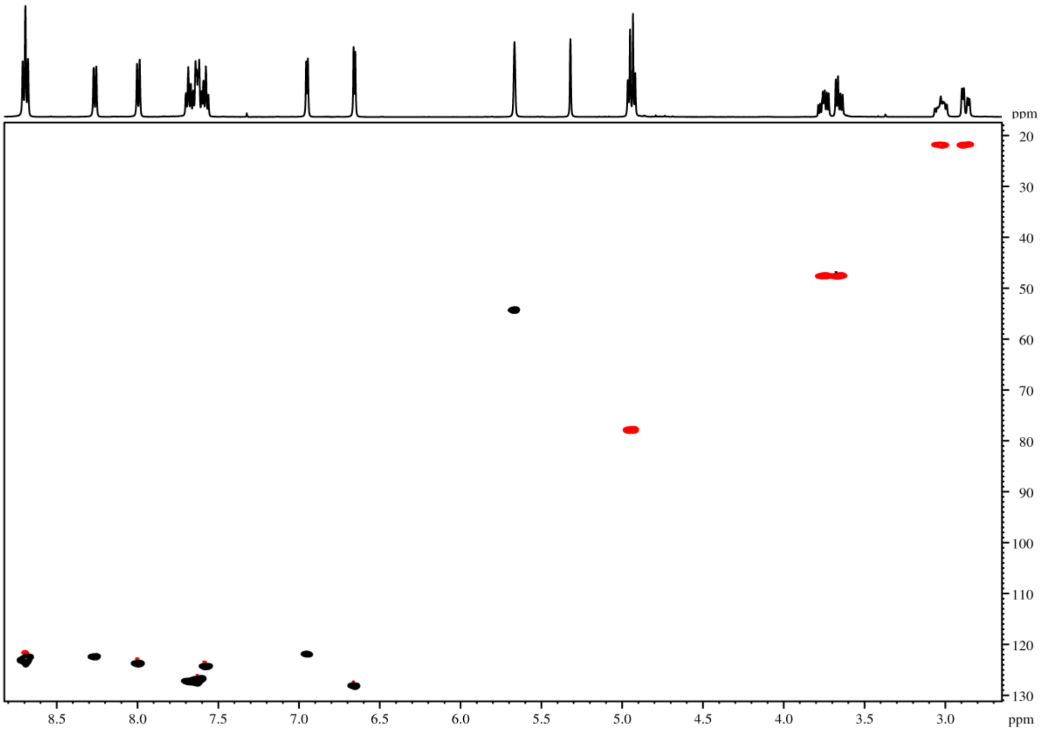

Figure S44. edited HSQC NMR spectrum of **9** (black: positive, red: negative signals)

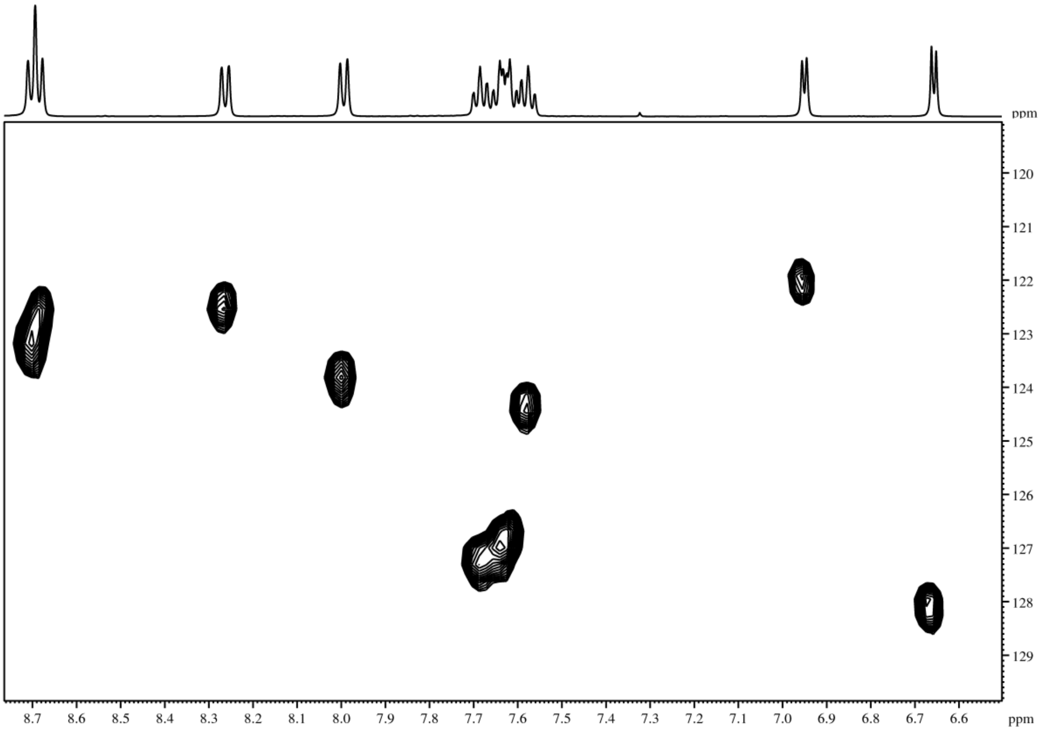

Figure S45. edited HSQC NMR spectrum of **9**, expansion (black: positive, red: negative signals)

Phenanthr[9,10-e][1,3]oxazino[4,3-a]-β-carboline (10)

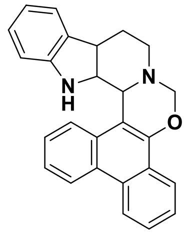

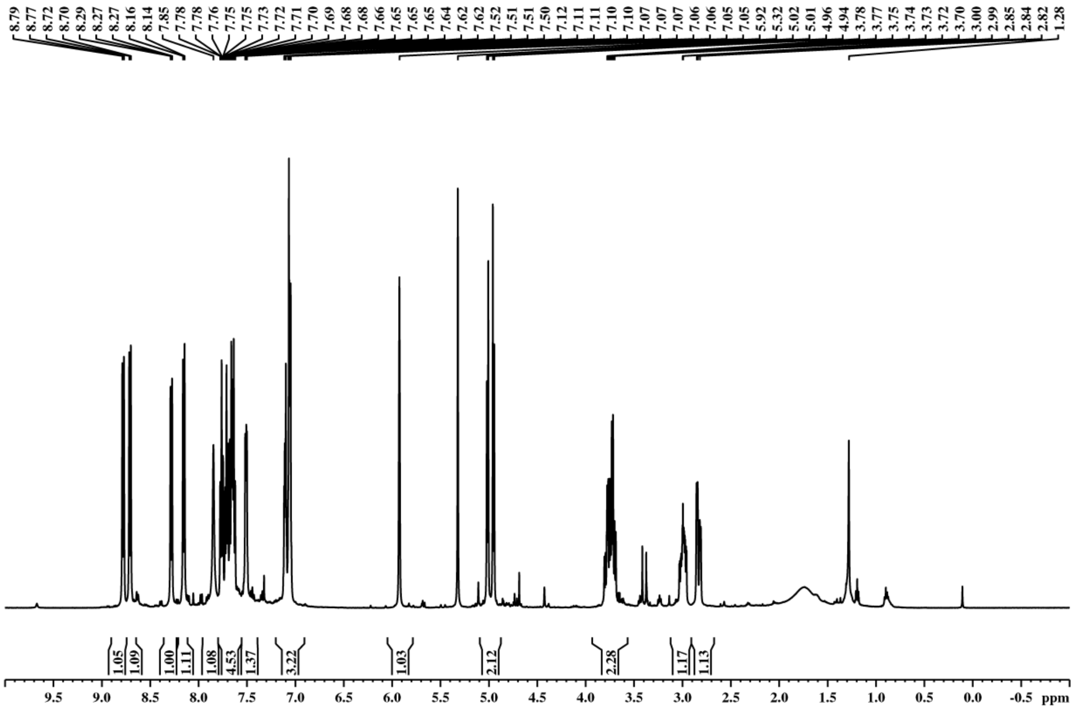

Figure S46. ^{1}H NMR spectrum of **10**

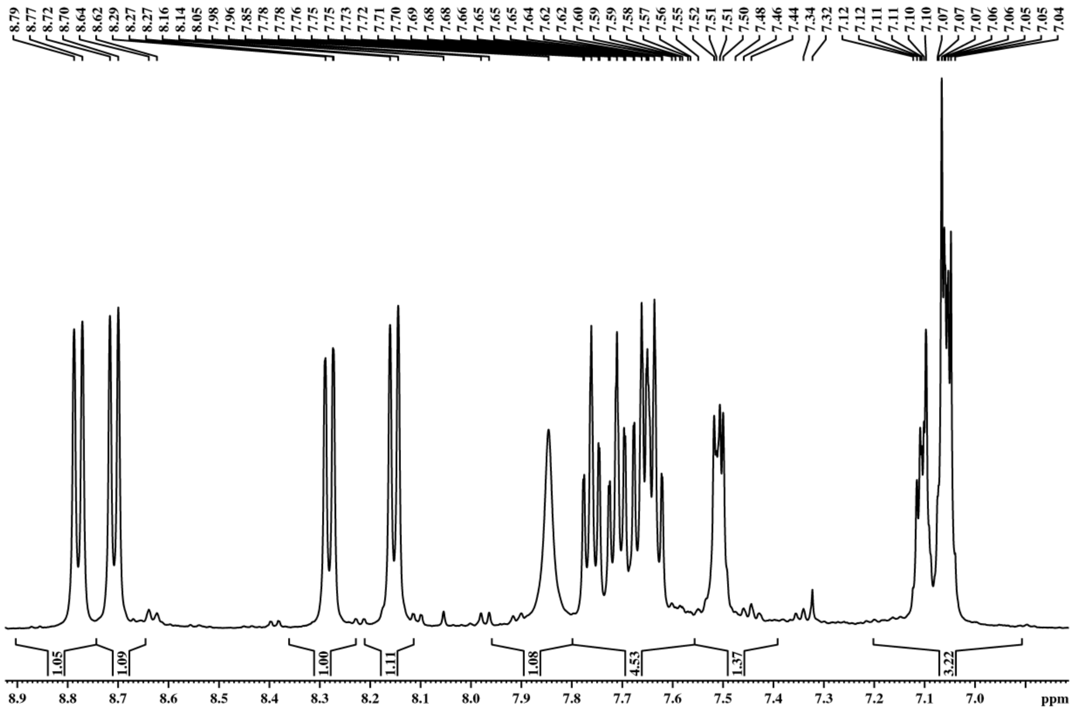

Figure S47. ^{1}H NMR spectrum of **10**, expansion

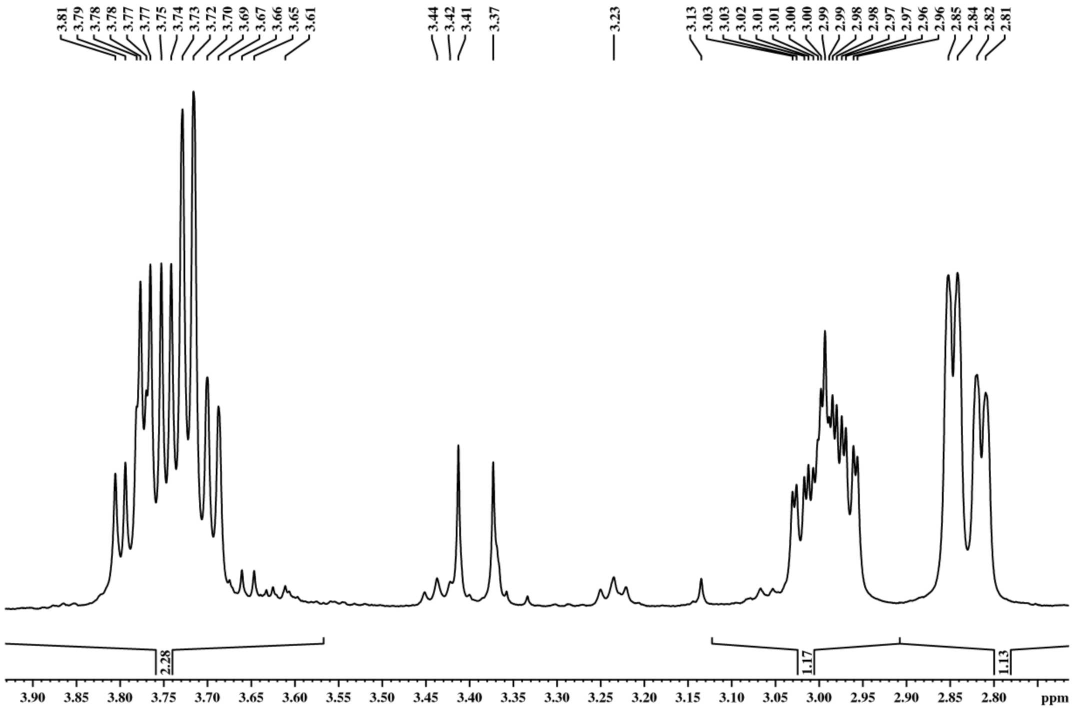

Figure S49. ^{1}H NMR spectrum of **10**, expansion

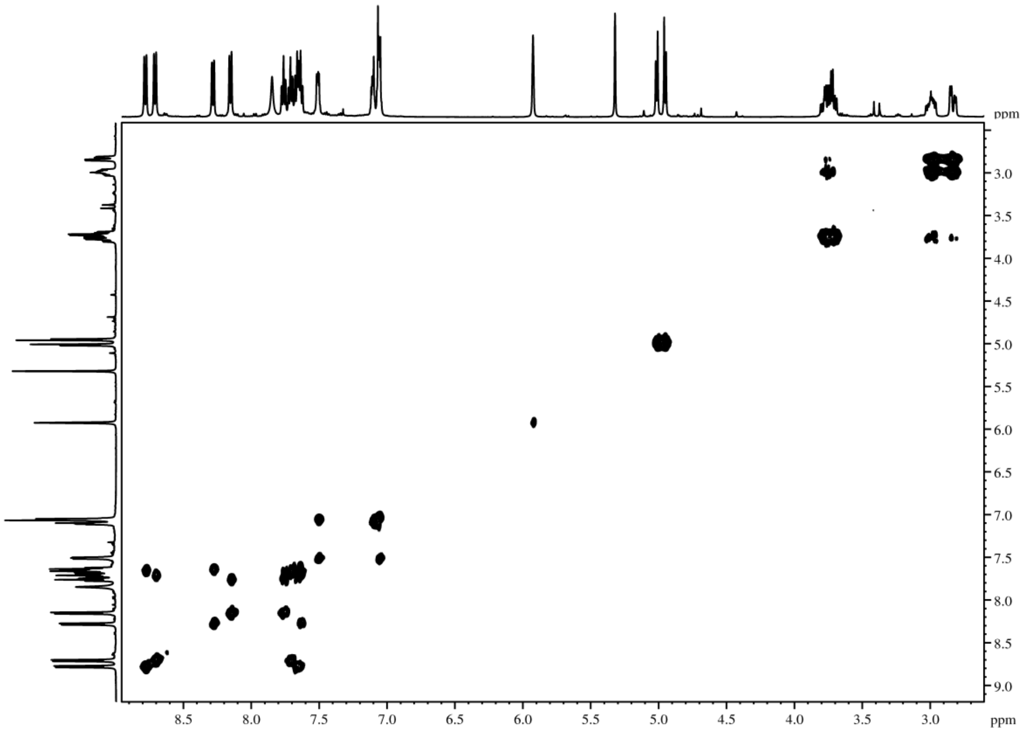

Figure S50. H,H-COSY NMR spectrum of **10**

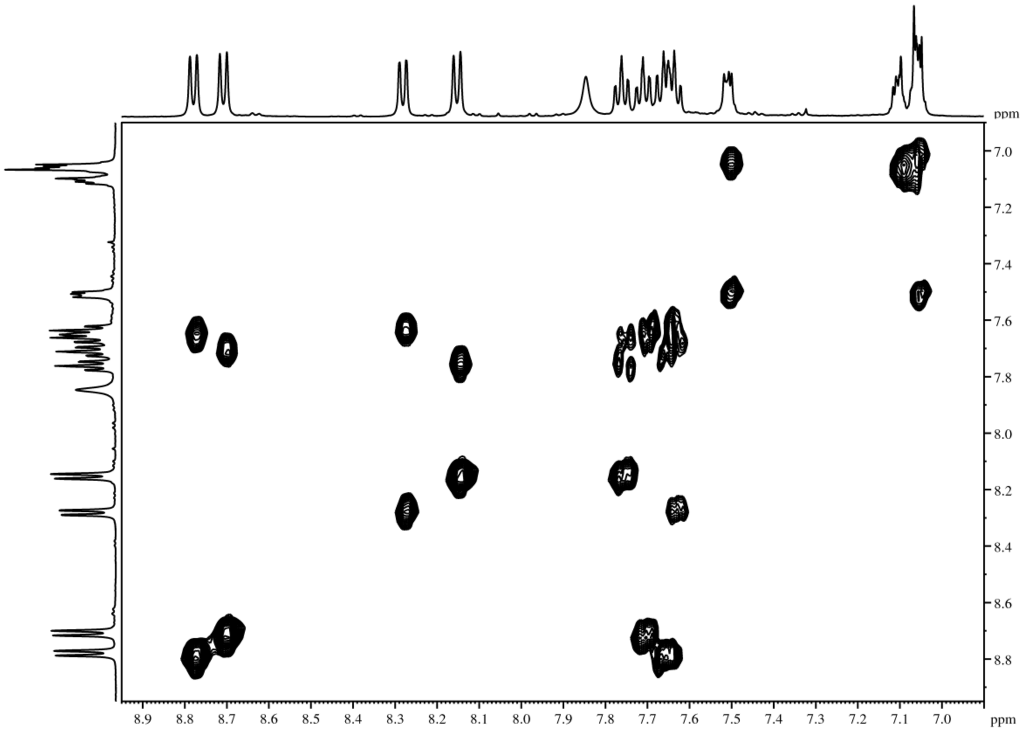

Figure S51. H,H-COSY NMR spectrum of **10**, expansion

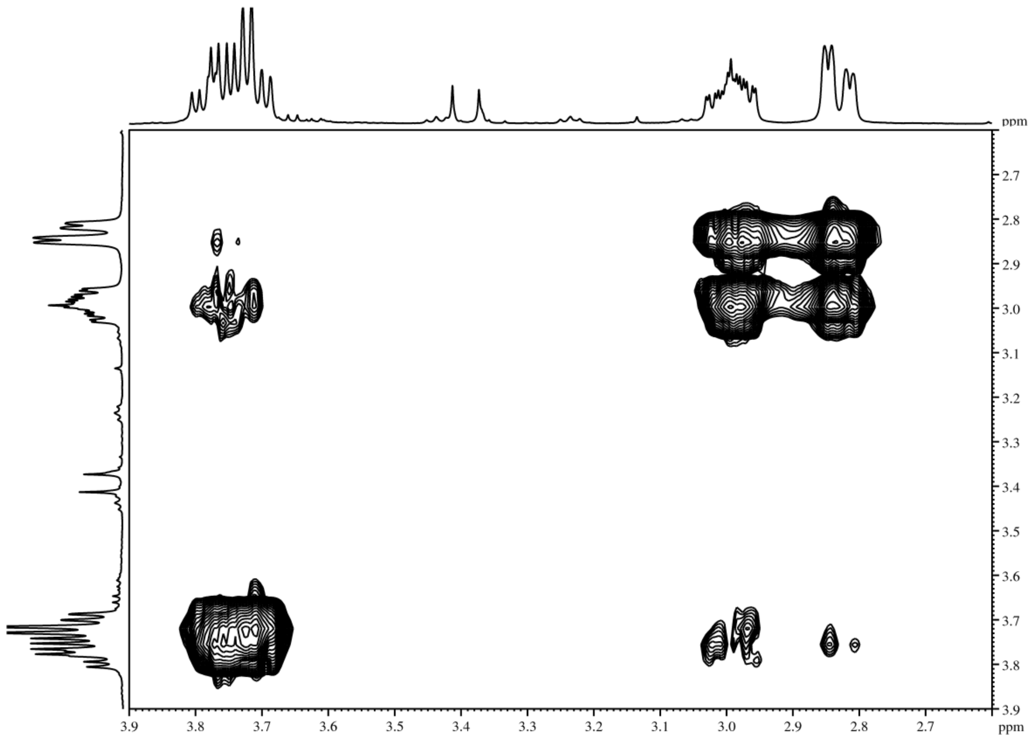

Figure S52. H,H-COSY NMR spectrum of **10**, expansion

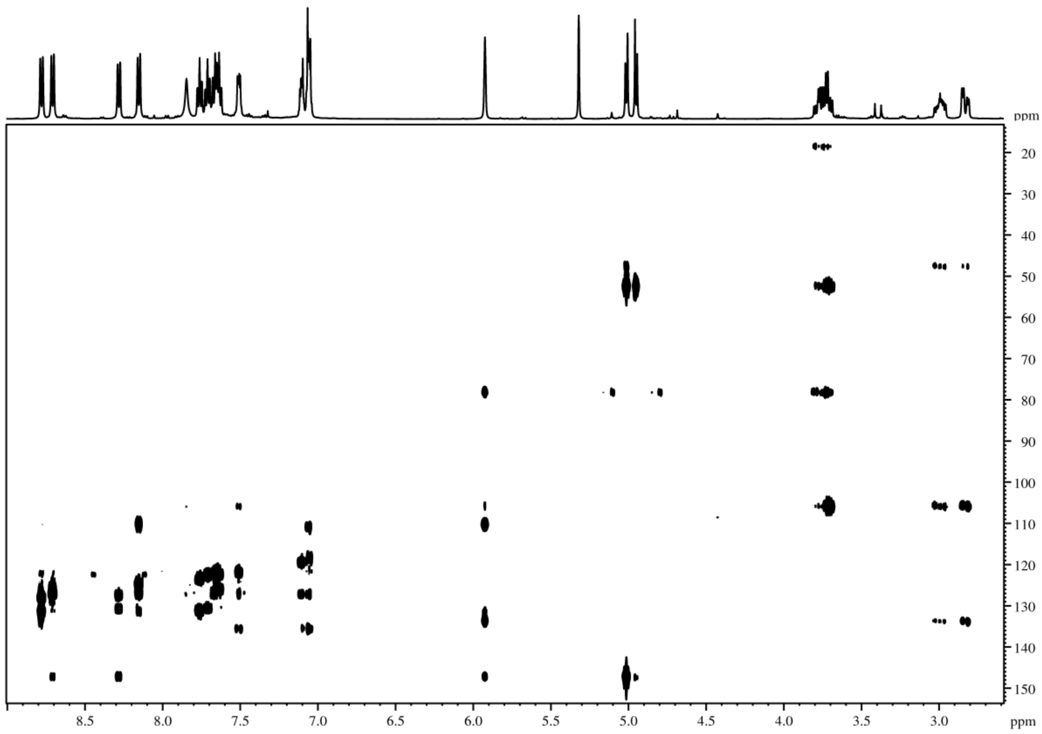

Figure S53. HMBC NMR spectrum of **10**

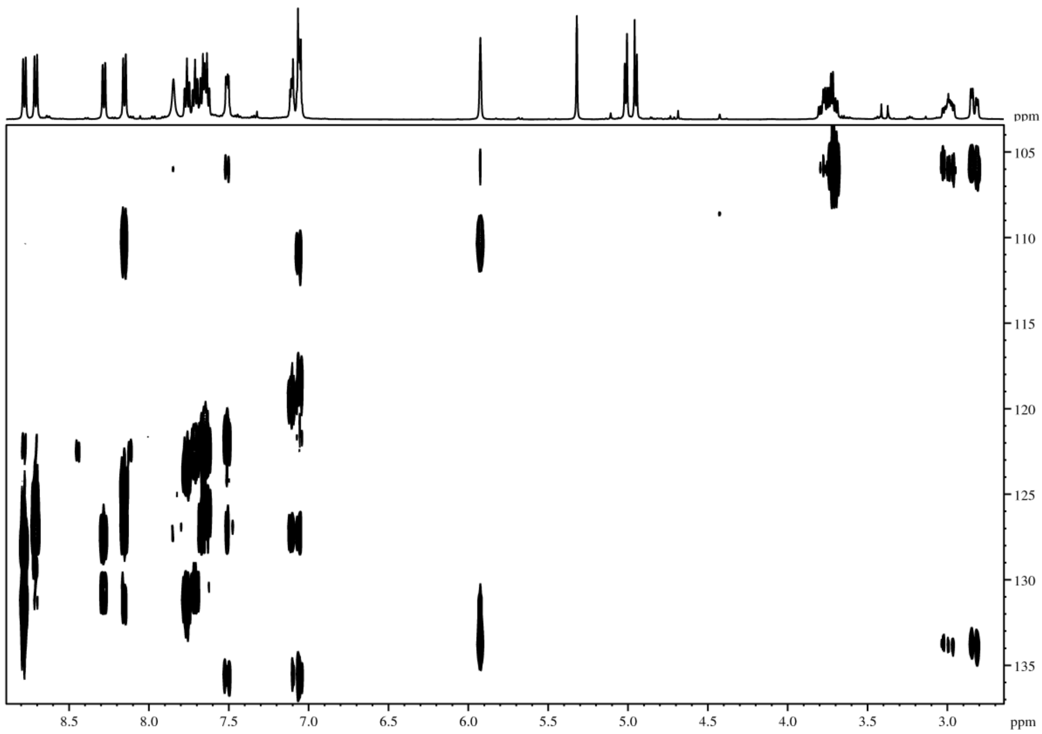

Figure S54. HMBC NMR spectrum of **10**, expansion

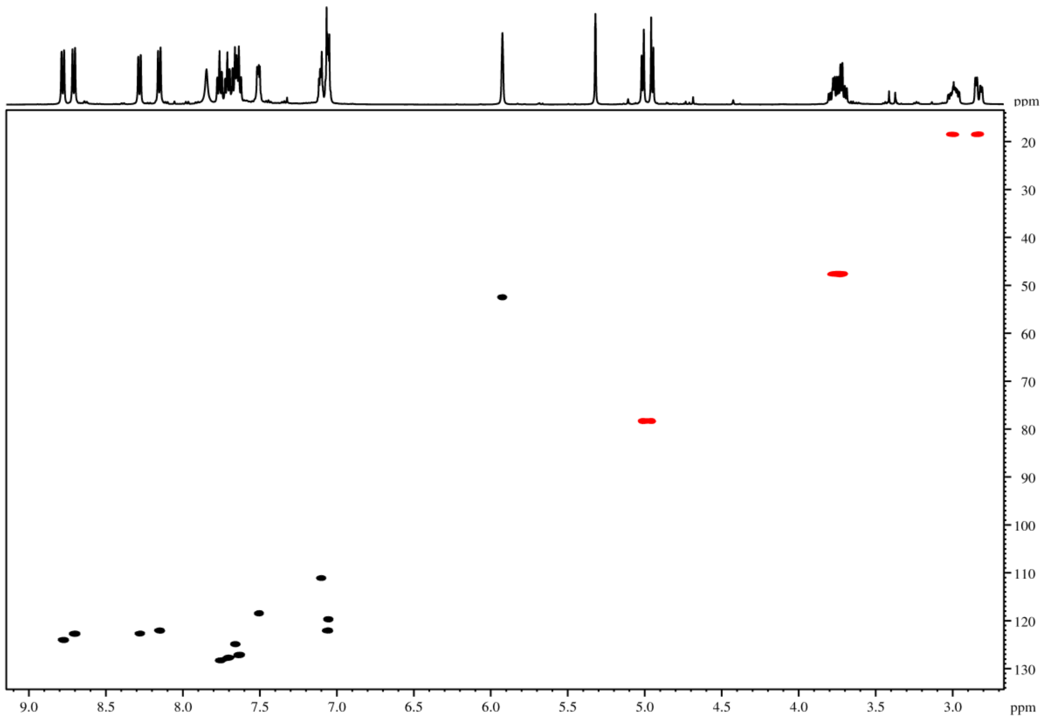

Figure S55. edited HSQC NMR spectrum of **10** (black: positive, red: negative signals)

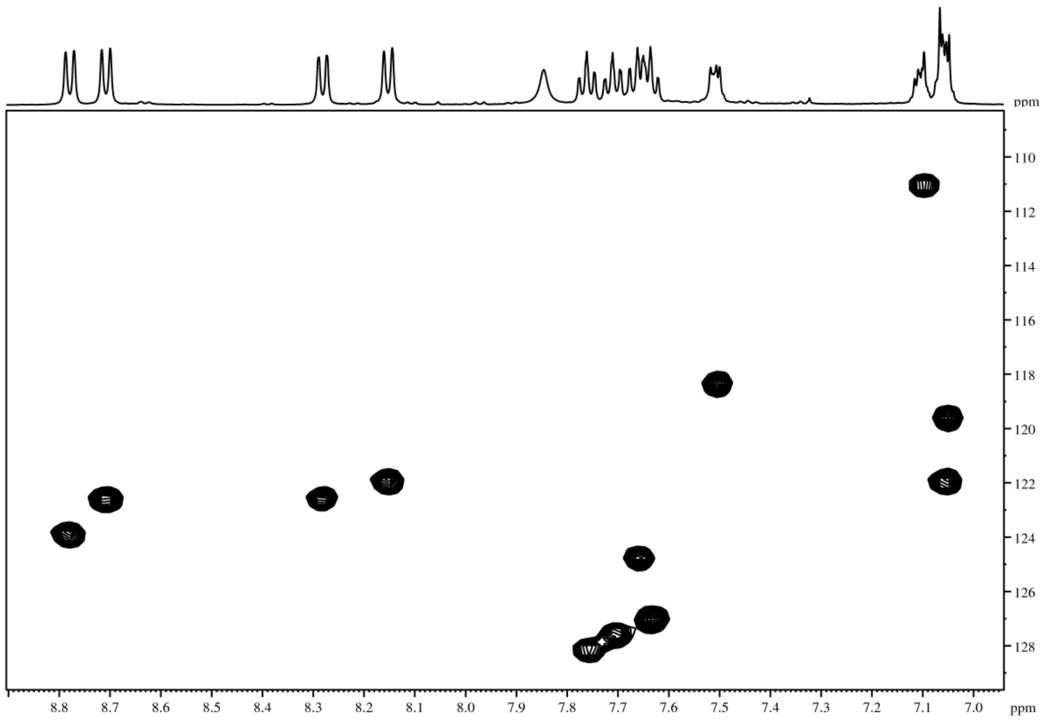

Figure S56. edited HSQC NMR spectrum of **10**, expansion (black: positive, red: negative signals)

10-[(Phenyl)-morpholin-4-yl-methyl]-9-phenanthrol (13)

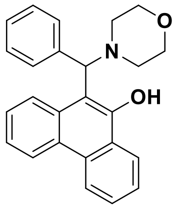

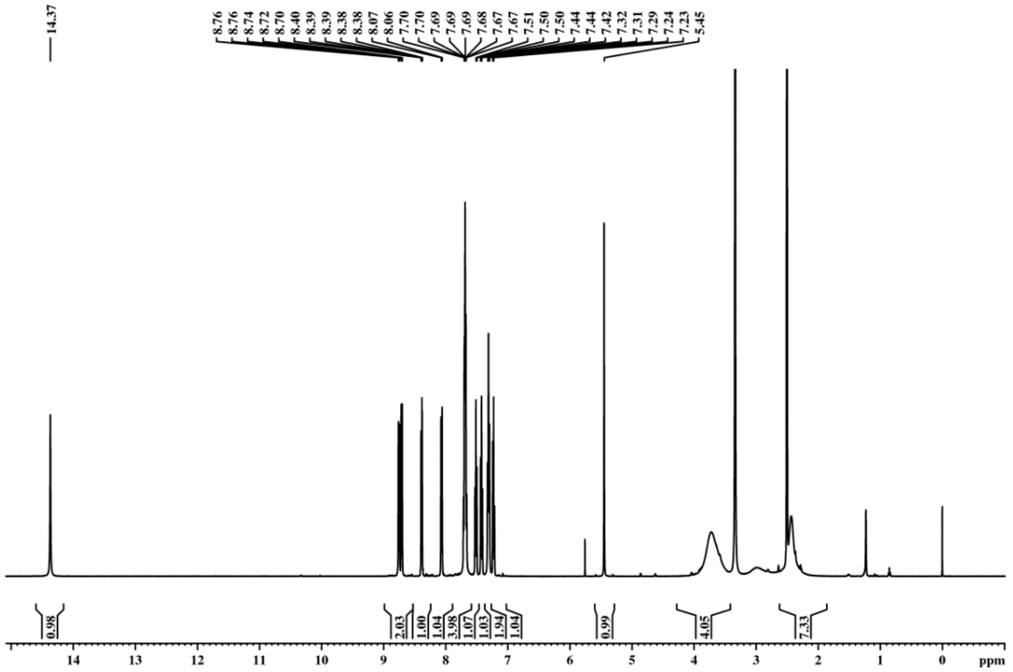

Figure S57. ^{1}H NMR spectrum of **13**

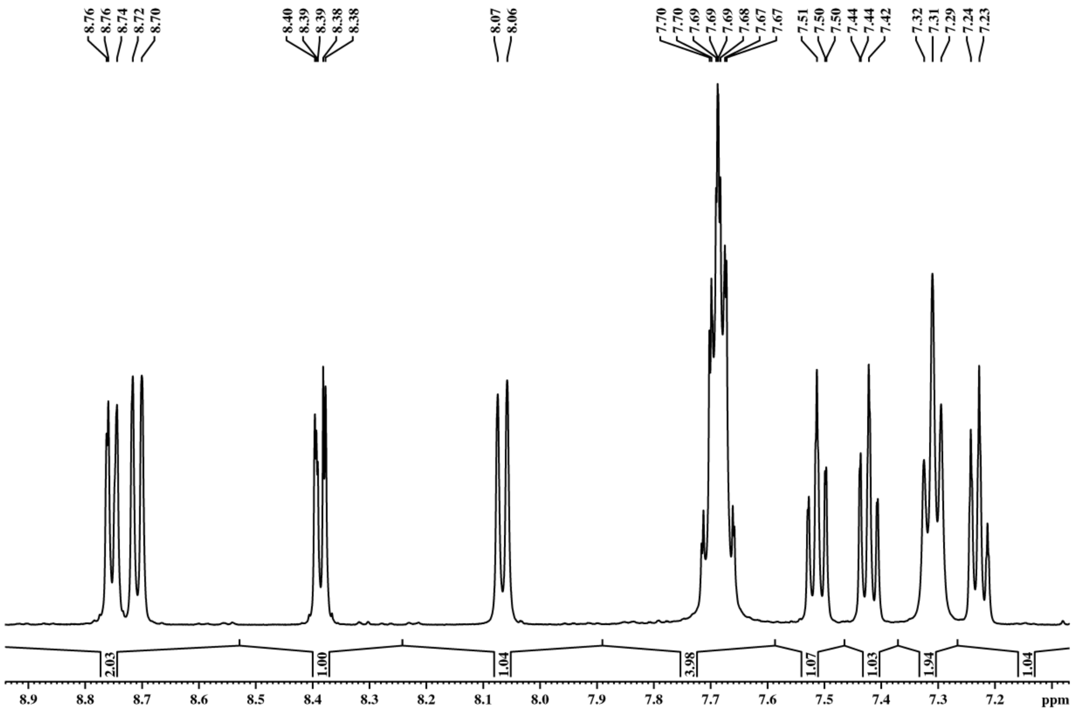

Figure S58. ^{1}H NMR spectrum of **13**, expansion

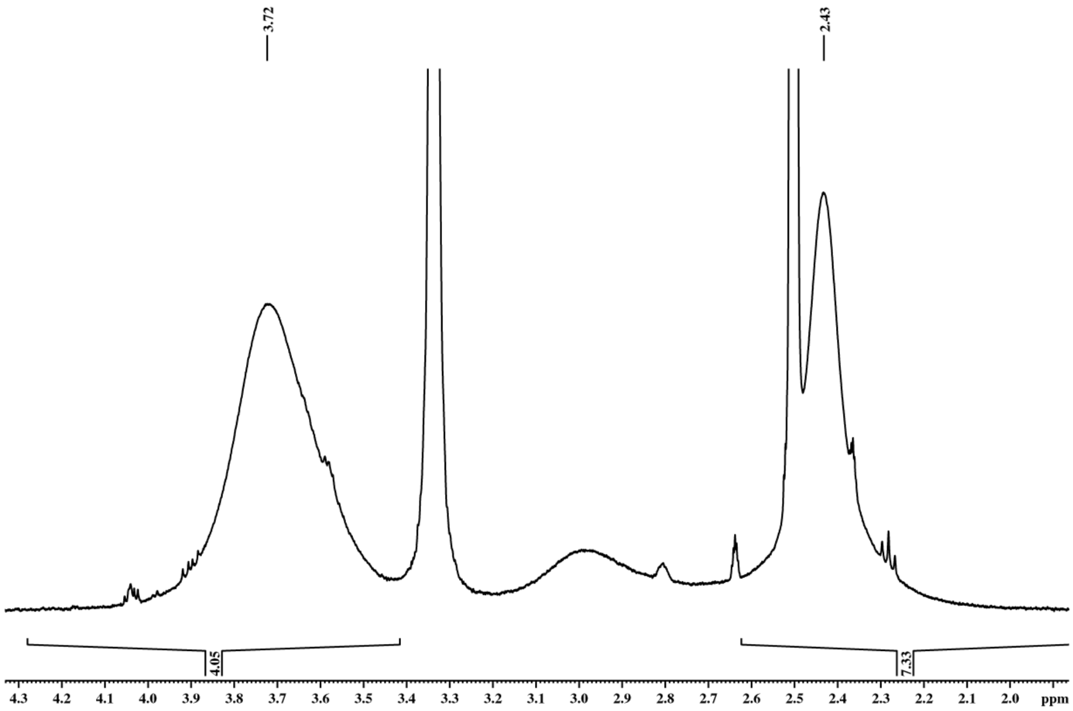

Figure S59. ^{1}H NMR spectrum of **13**, expansion

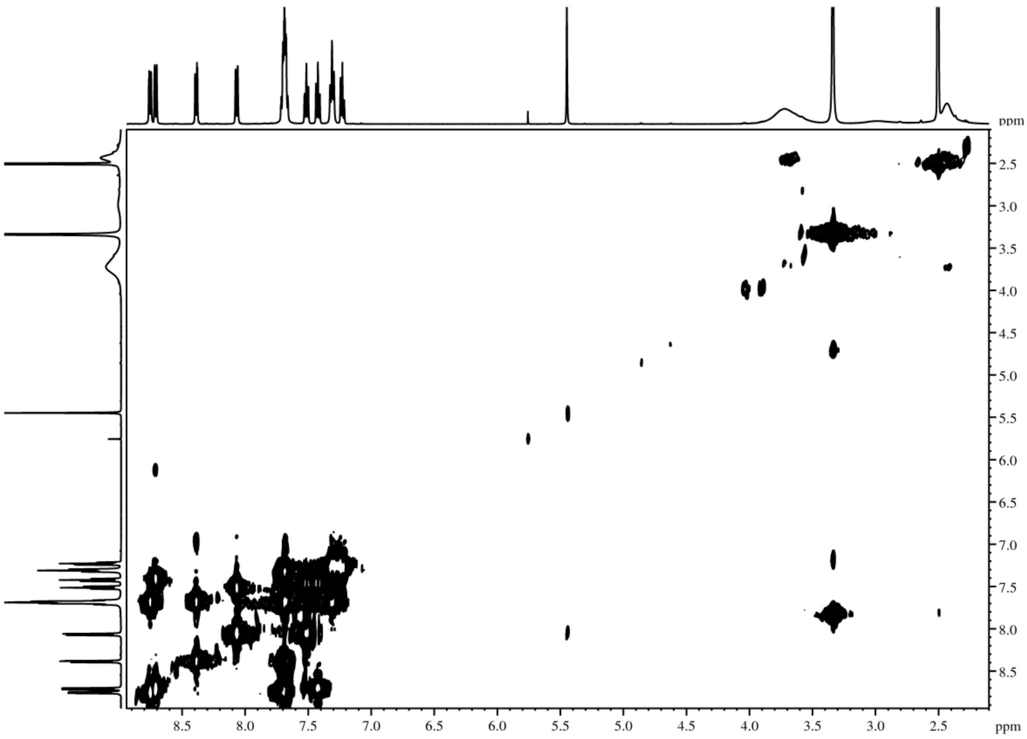

Figure S60. H,H-COSY NMR spectrum of **13**

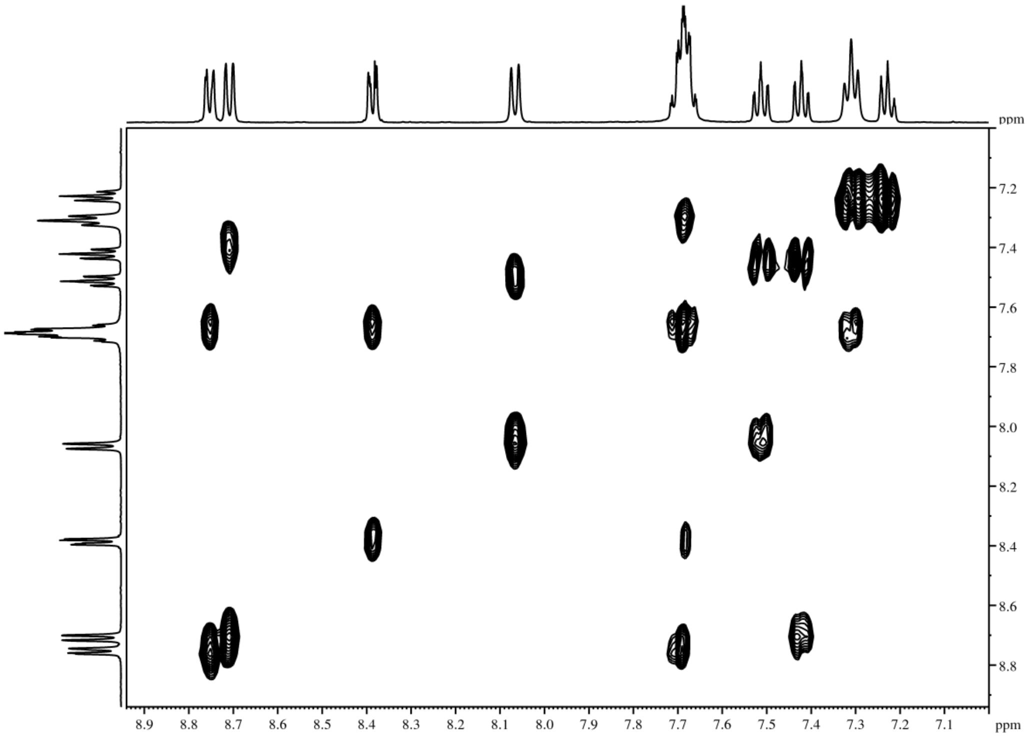

Figure S61. H,H-COSY NMR spectrum of **13**, expansion

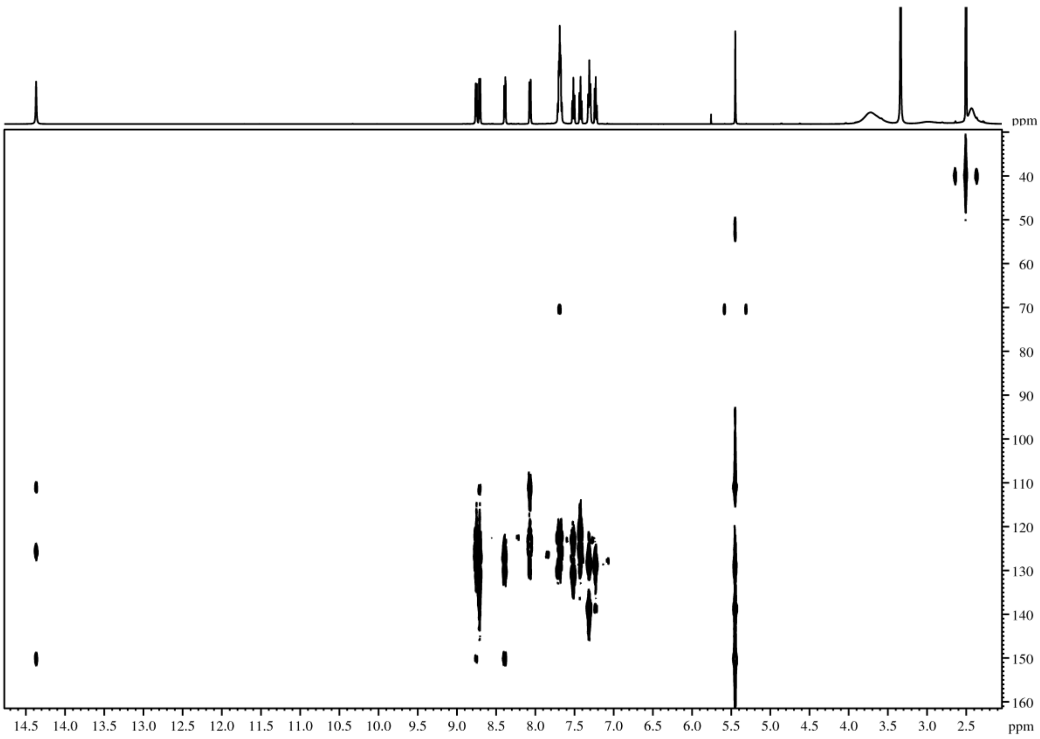

Figure S62. HMBC NMR spectrum of **13**

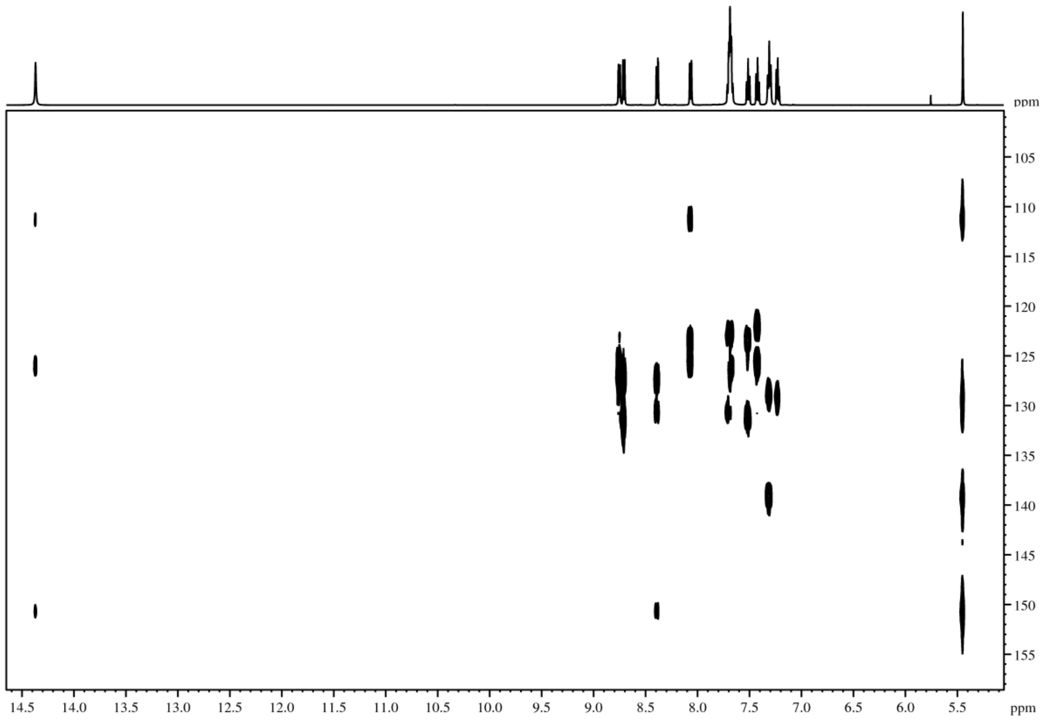

Figure S63. HMBC NMR spectrum of **13**, expansion

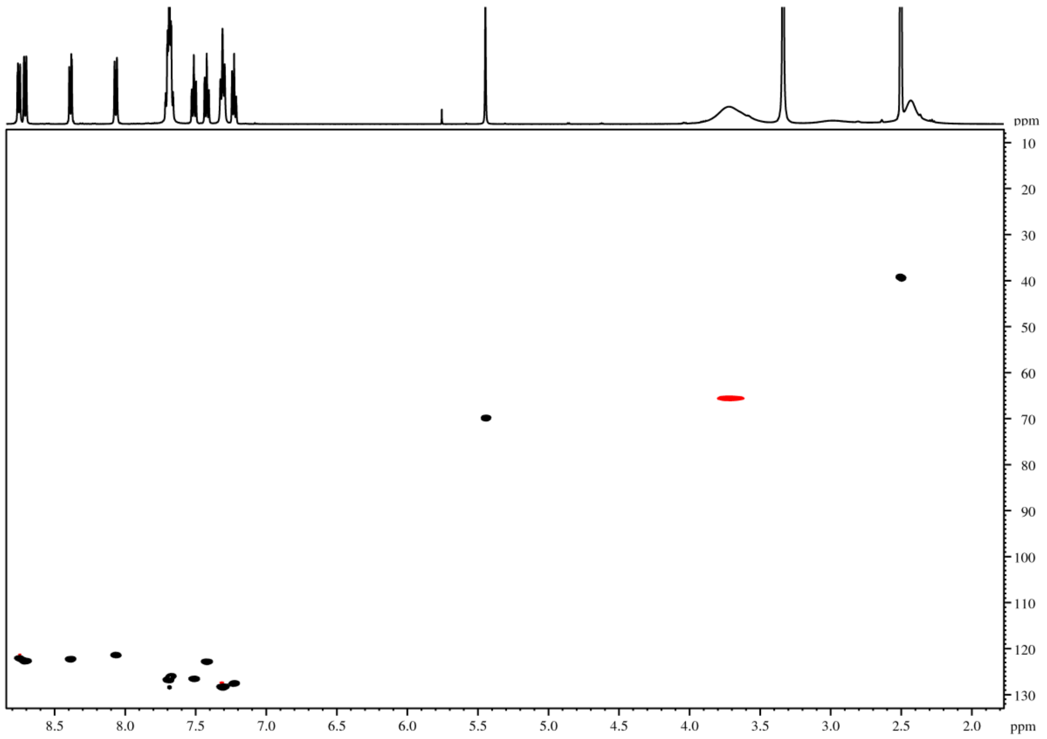

Figure S64. edited HSQC NMR spectrum of **13** (black: positive, red: negative signals)

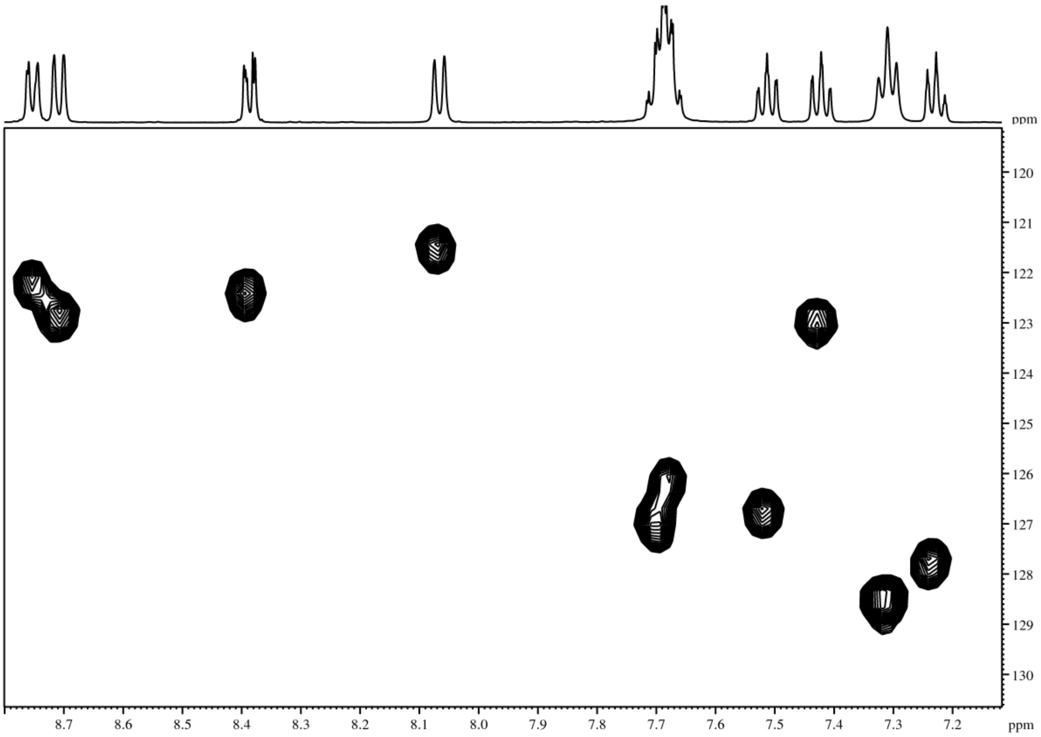

Figure S65. edited HSQC NMR spectrum of **13**, expansion (black: positive, red: negative signals)

9aR,17S*-15-(Phenyl)-phenanthr[9,10-e]oxazino[2,3-a]isoquinoline (14)*

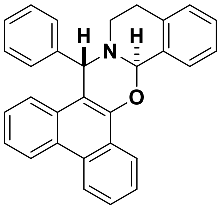

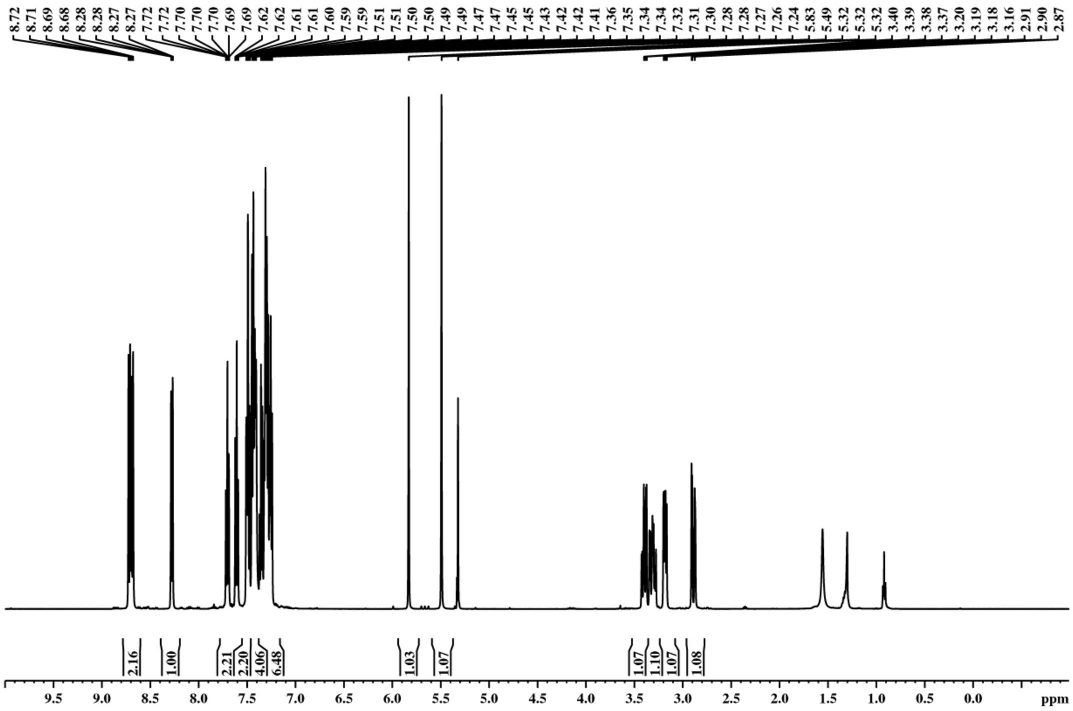

Figure S66. ^{1}H NMR spectrum of **14**

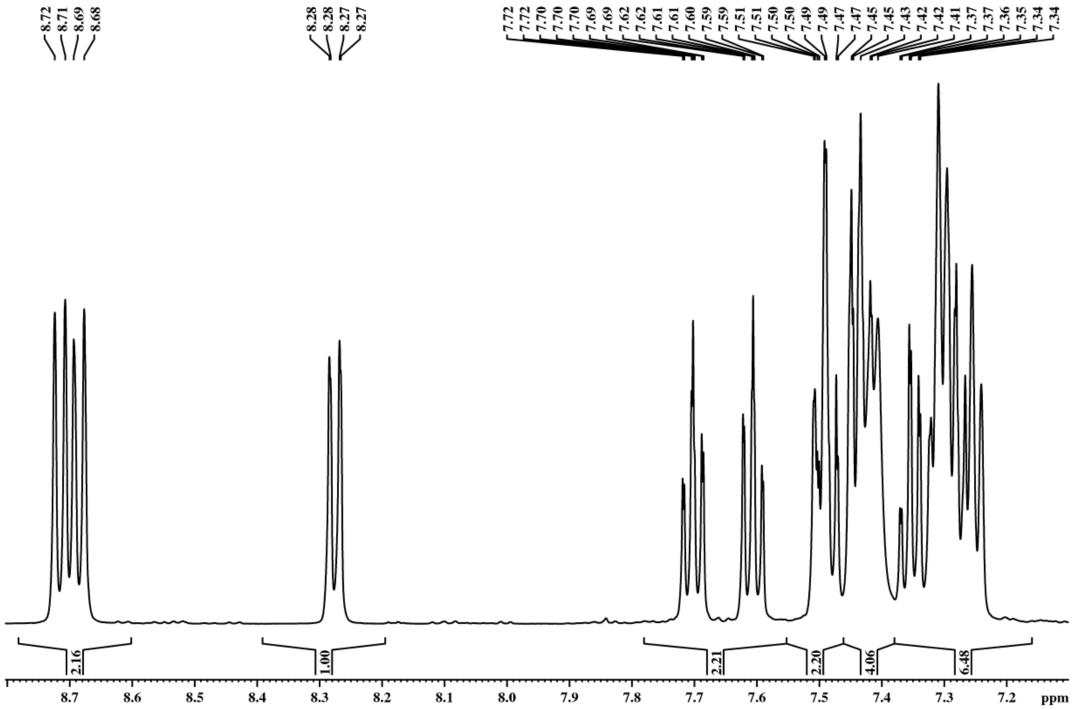

Figure S67. ^{1}H NMR spectrum of **14**, expansion

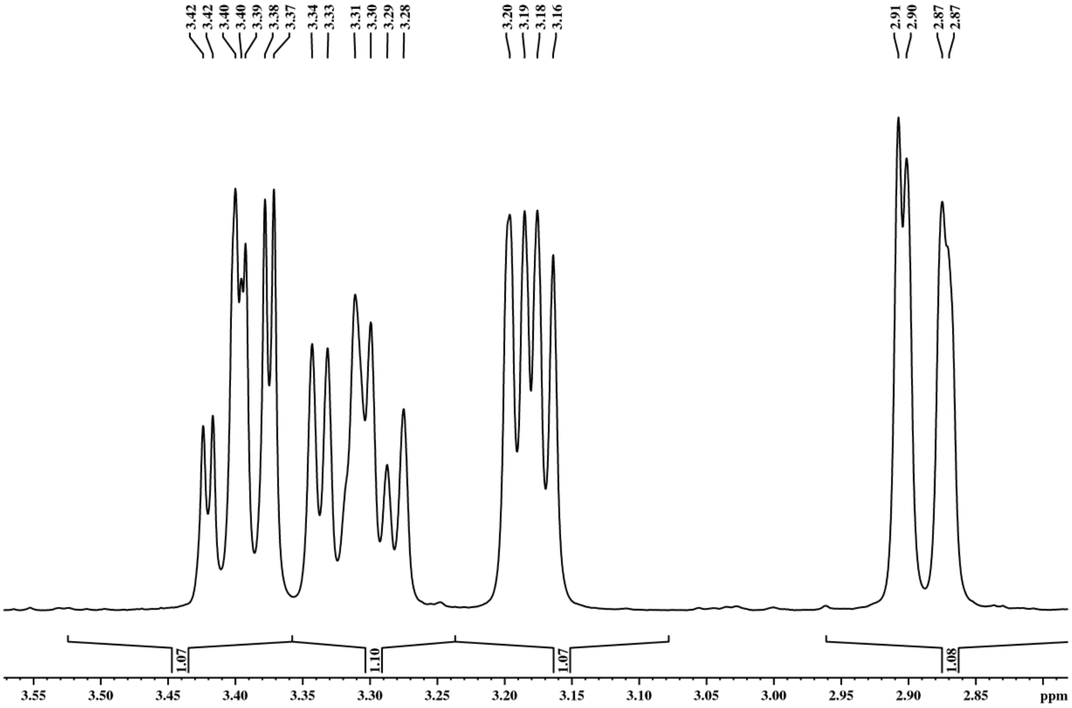

Figure S68. ^{1}H NMR spectrum of **14**, expansion

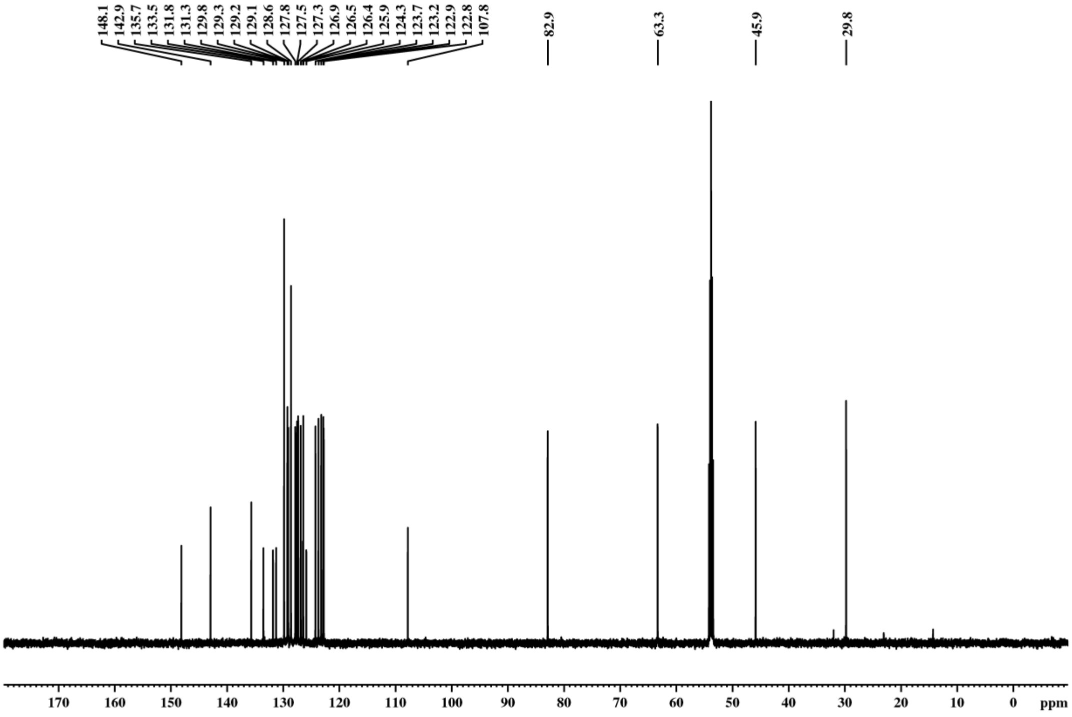

Figure S69. ^{13}C NMR spectrum of **14**

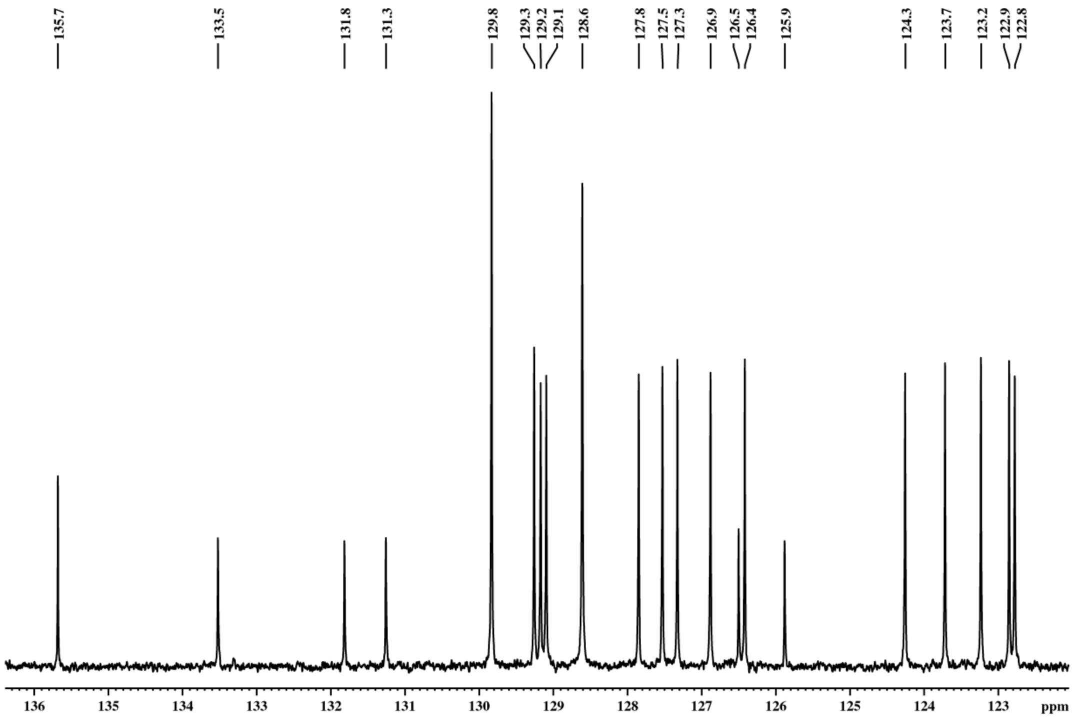

Figure S70. ^{13}C NMR spectrum of **14**, expansion

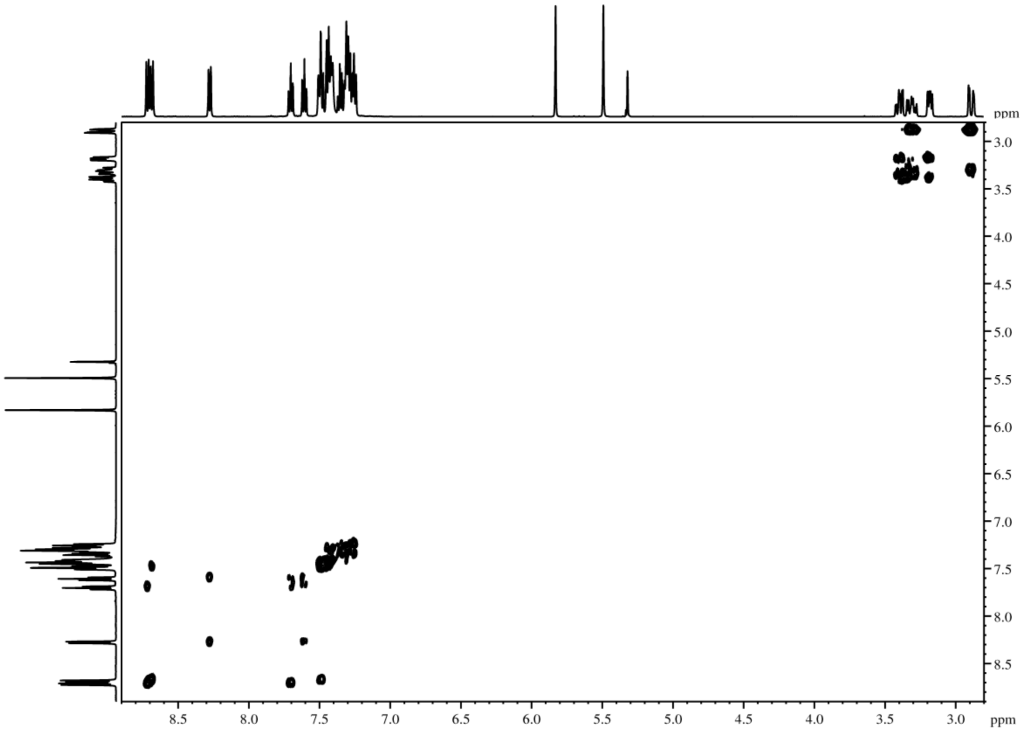

Figure S71. H,H-COSY NMR spectrum of **14**

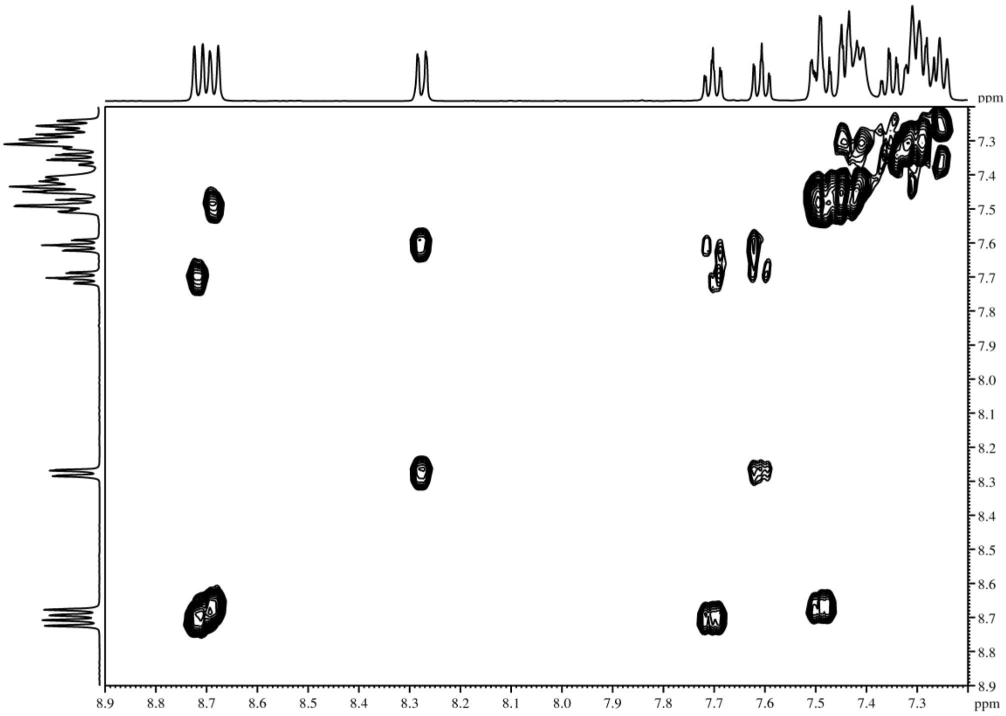

Figure S72. H,H-COSY NMR spectrum of **14**, expansion

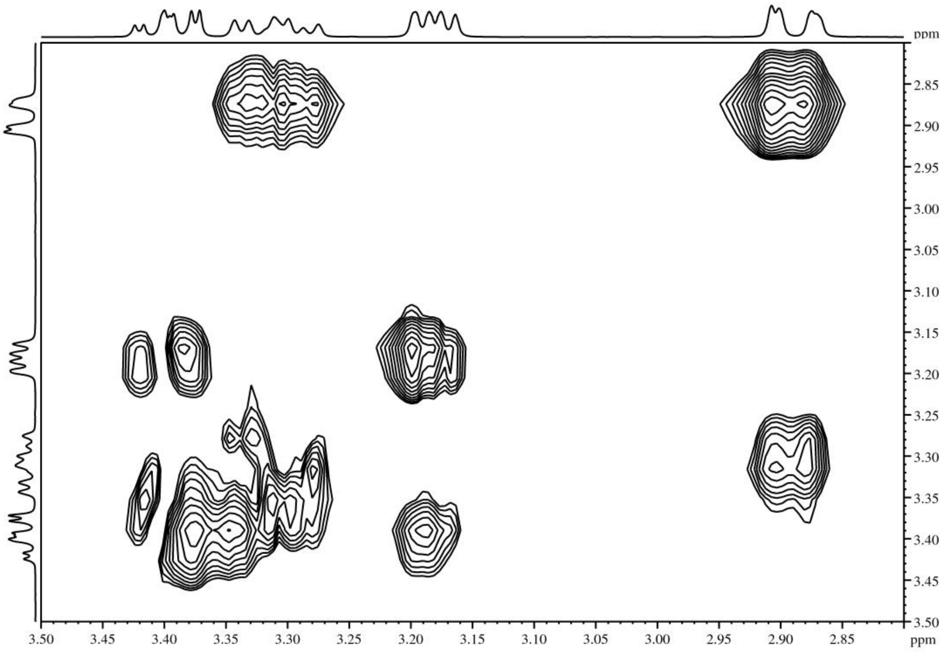

Figure S73. H,H-COSY NMR spectrum of **14**, expansion

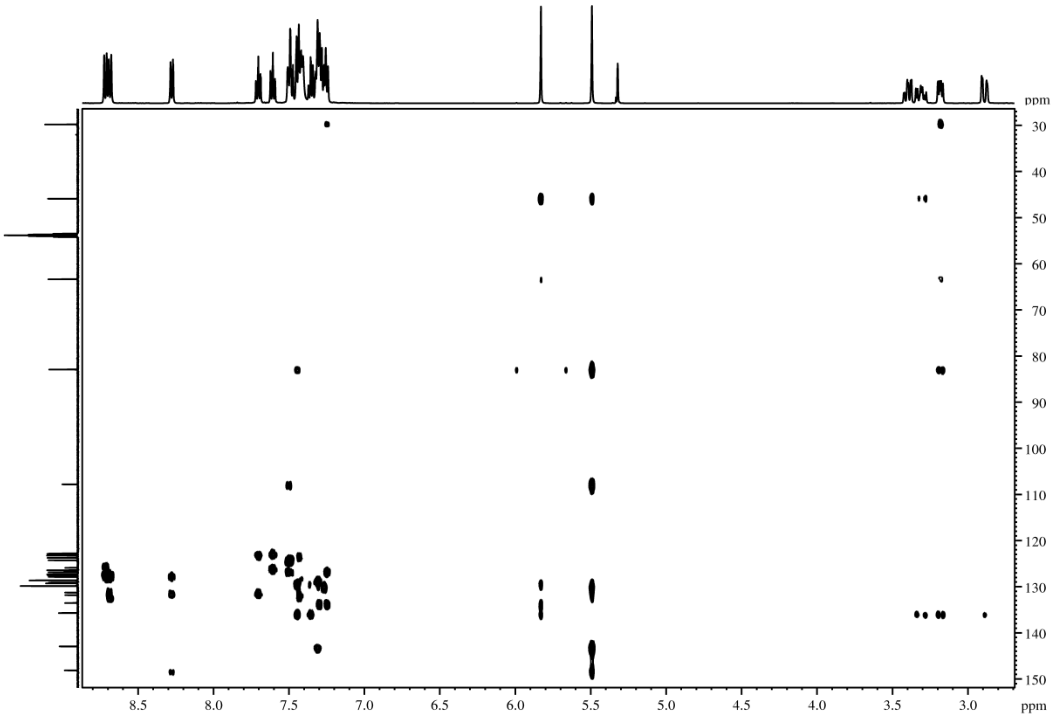

Figure S74. HMBC NMR spectrum of **14**

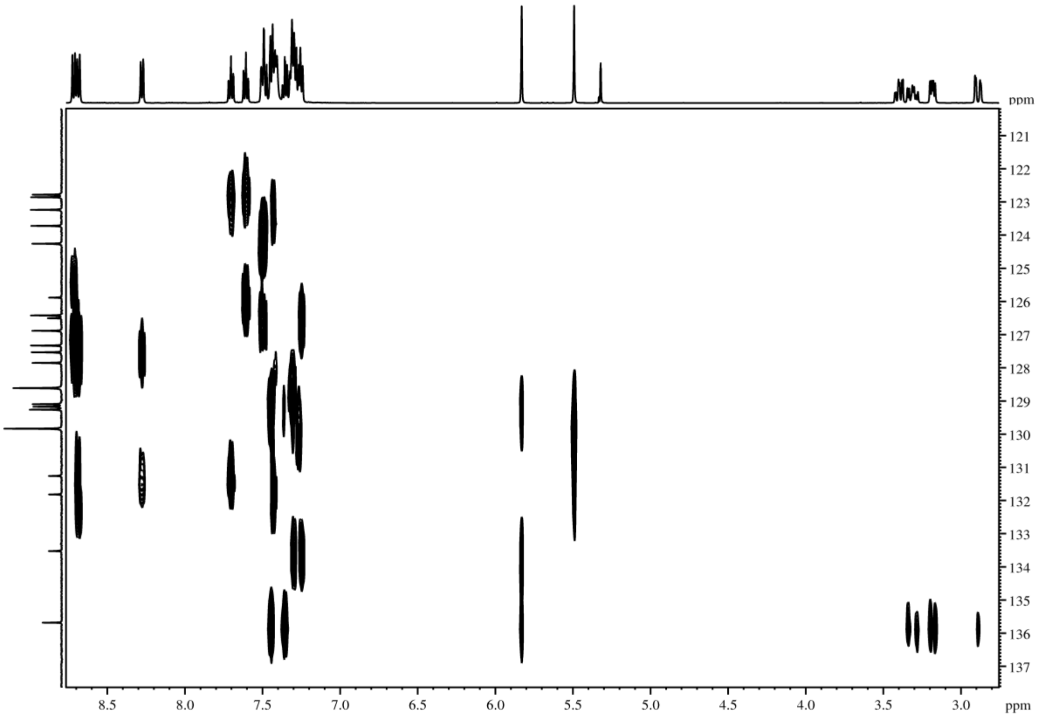

Figure S75. HMBC NMR spectrum of **14**, expansion

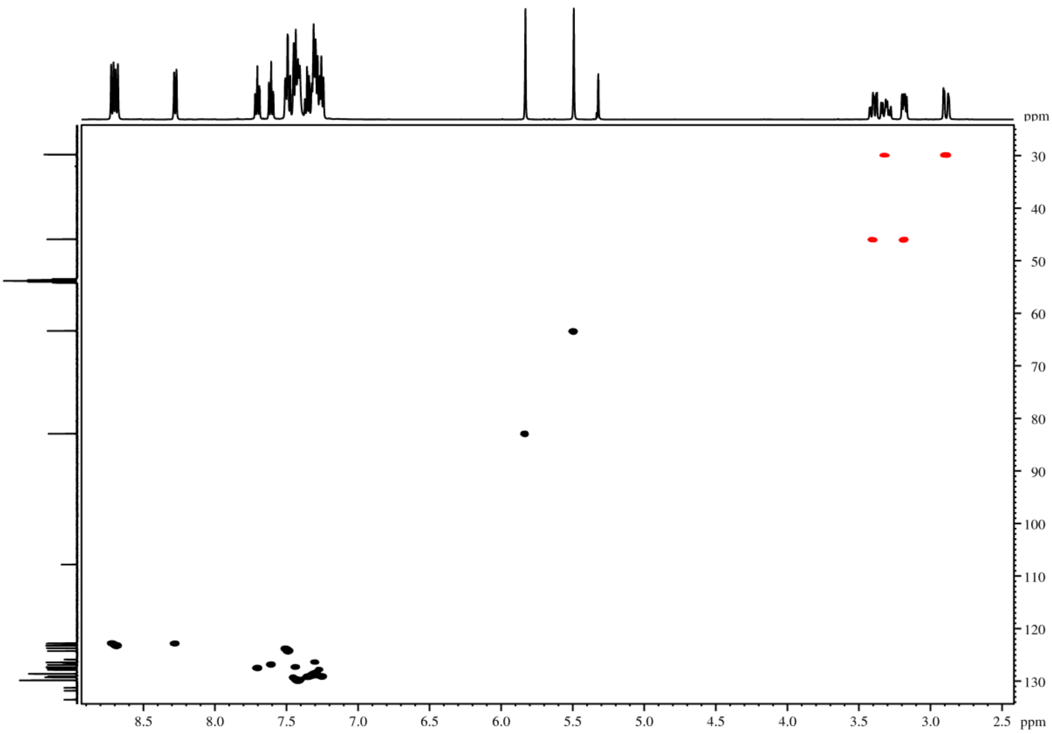

Figure S76. edited HSQC NMR spectrum of **14** (black: positive, red: negative signals)

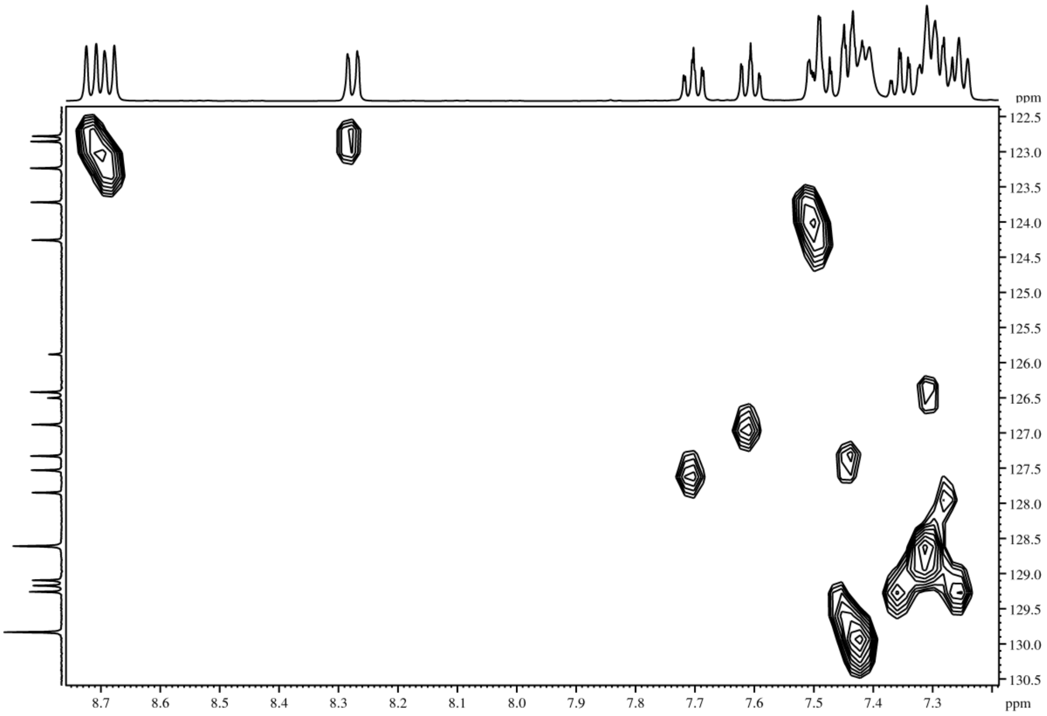

Figure S77. edited HSQC NMR spectrum of **14**, expansion (black: positive, red: negative signals)

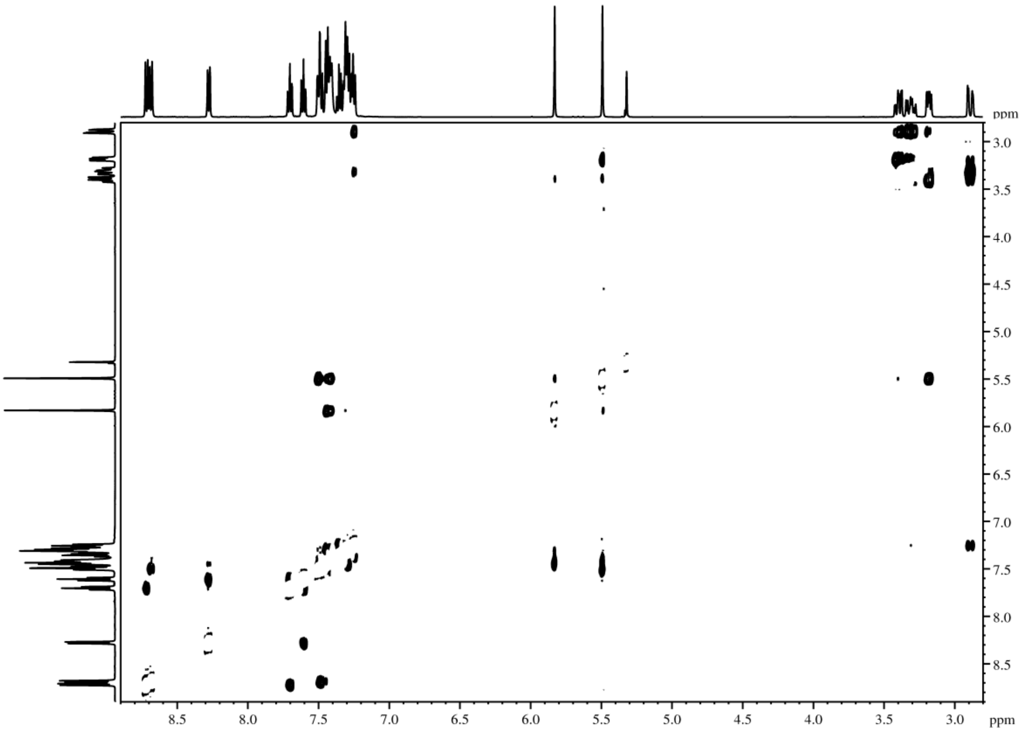

Figure S78. Phase-sensitive NOESY NMR spectrum of **14** (only positive signals, mixing time 800 ms)

9aR*,16S*-16-(Phenyl)-phenanthr[9,10-e]oxazino[2,3-a]thieno[3,2-c]pyridine (15)

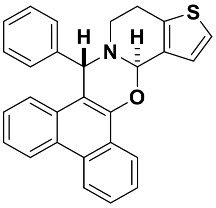

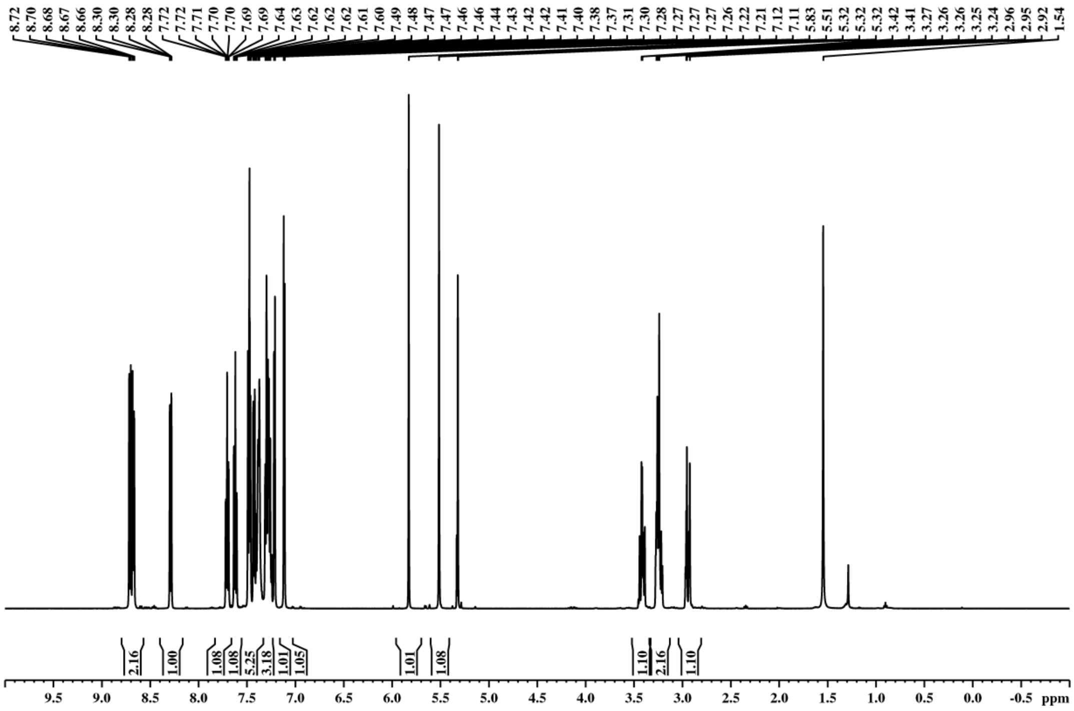

Figure S79. ^{1}H NMR spectrum of **15**

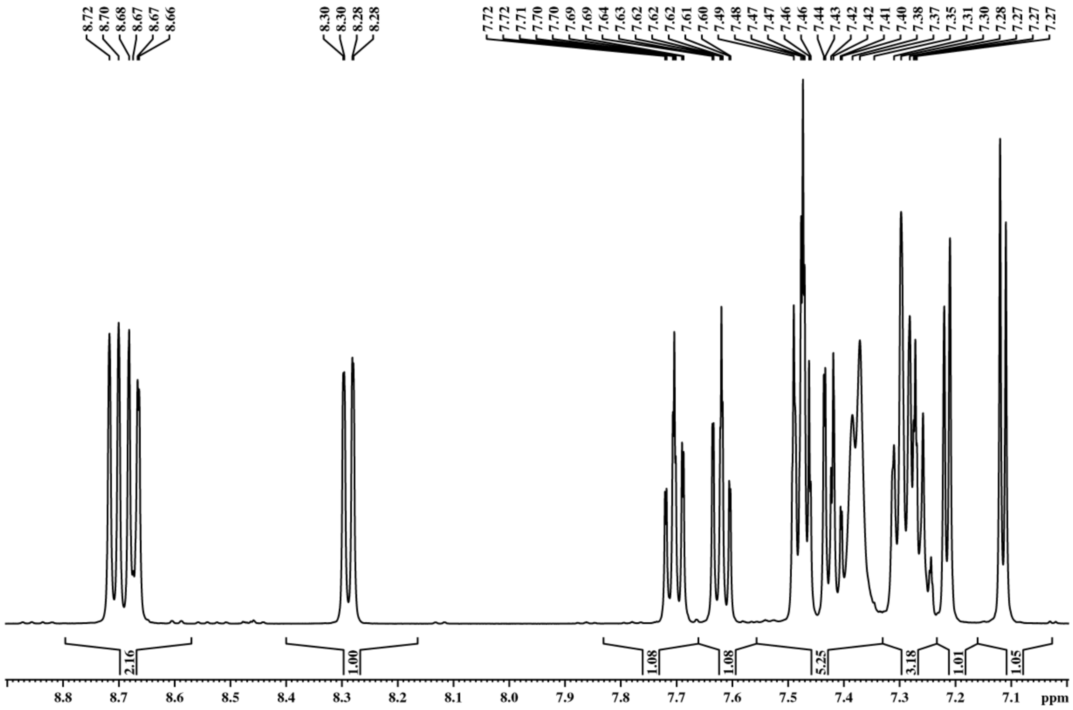

Figure S80. ^{1}H NMR spectrum of **15**, expansion

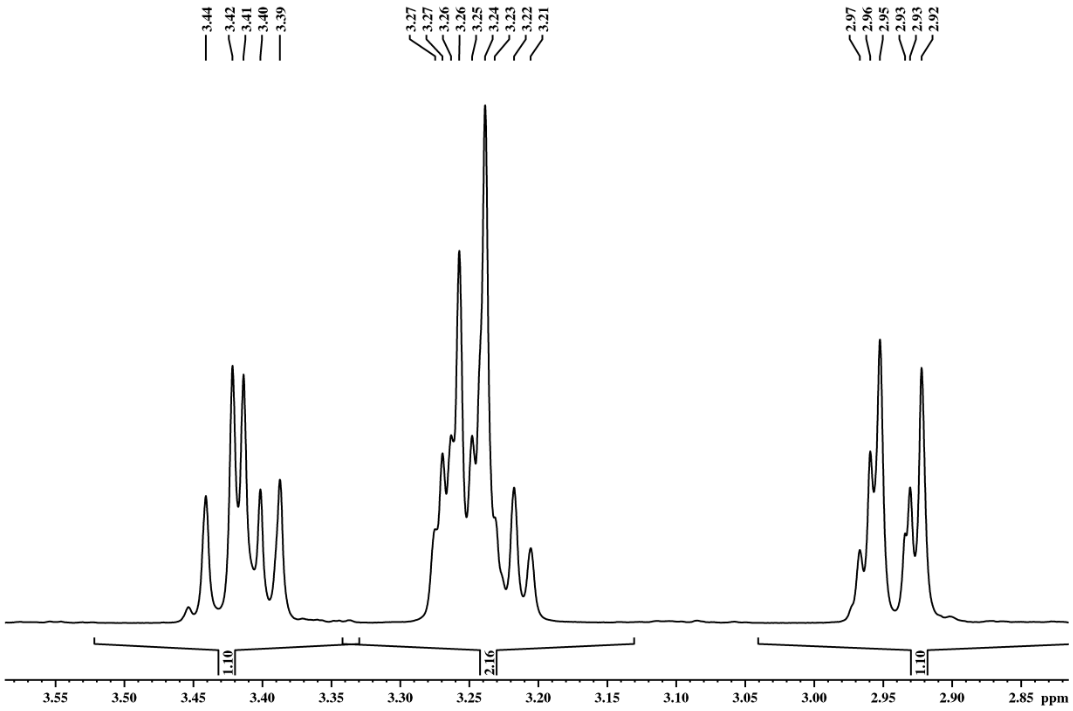

Figure S81. ^{1}H NMR spectrum of **15**, expansion

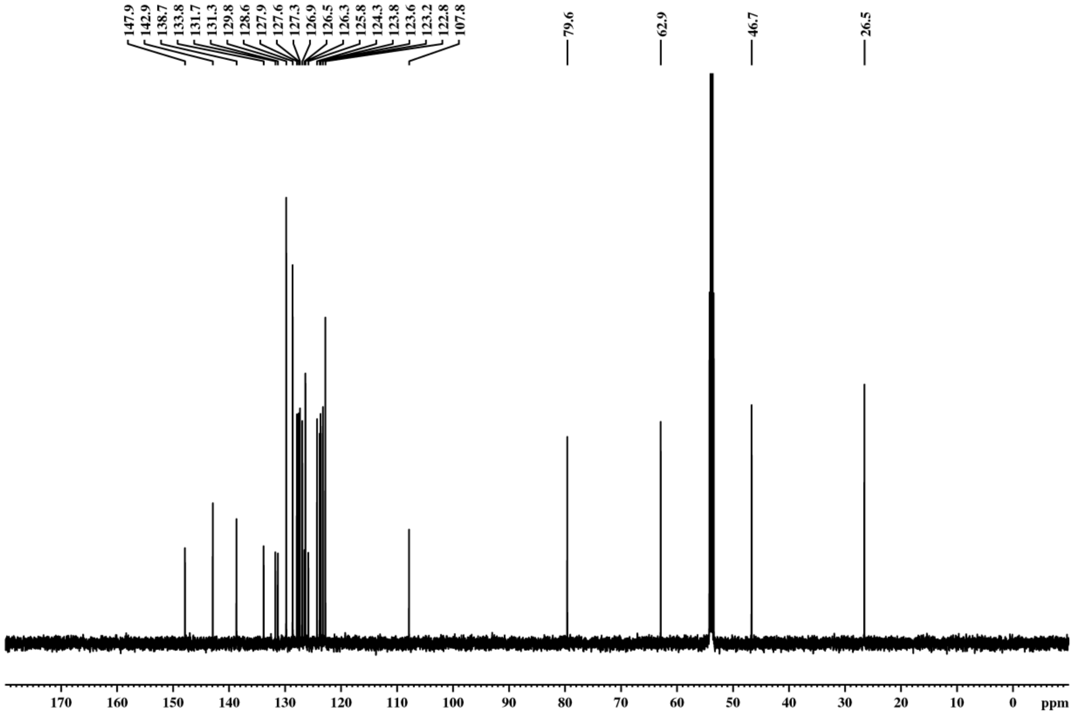

Figure S82. ^{13}C NMR spectrum of **15**

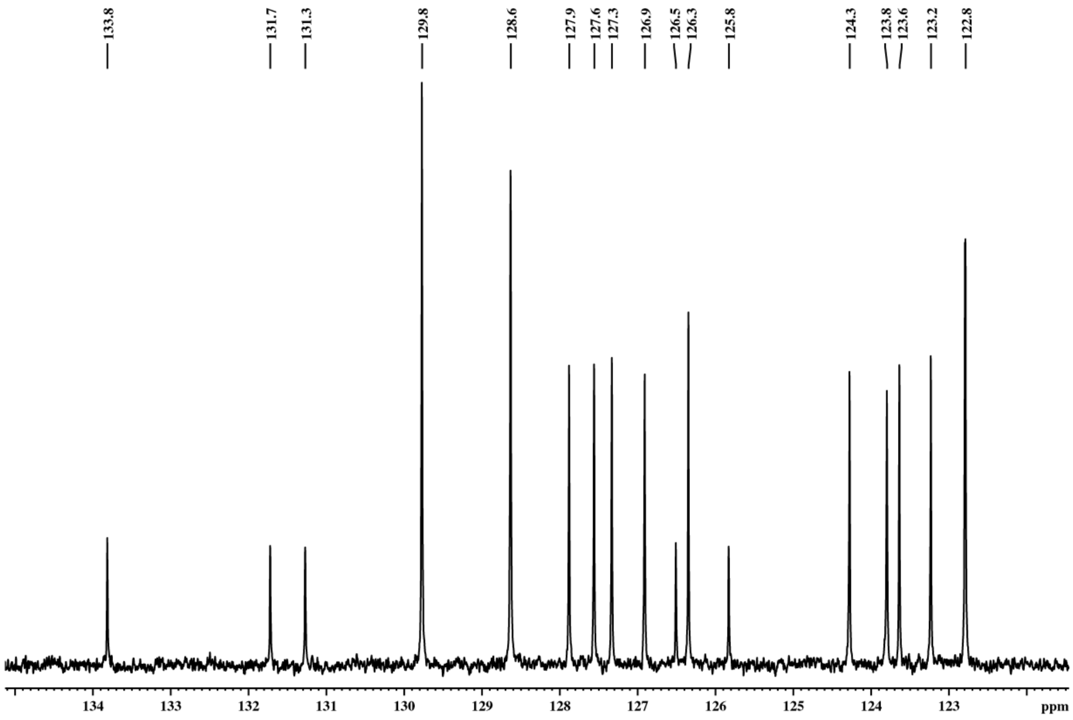

Figure S83. ^{13}C NMR spectrum of **15**, expansion

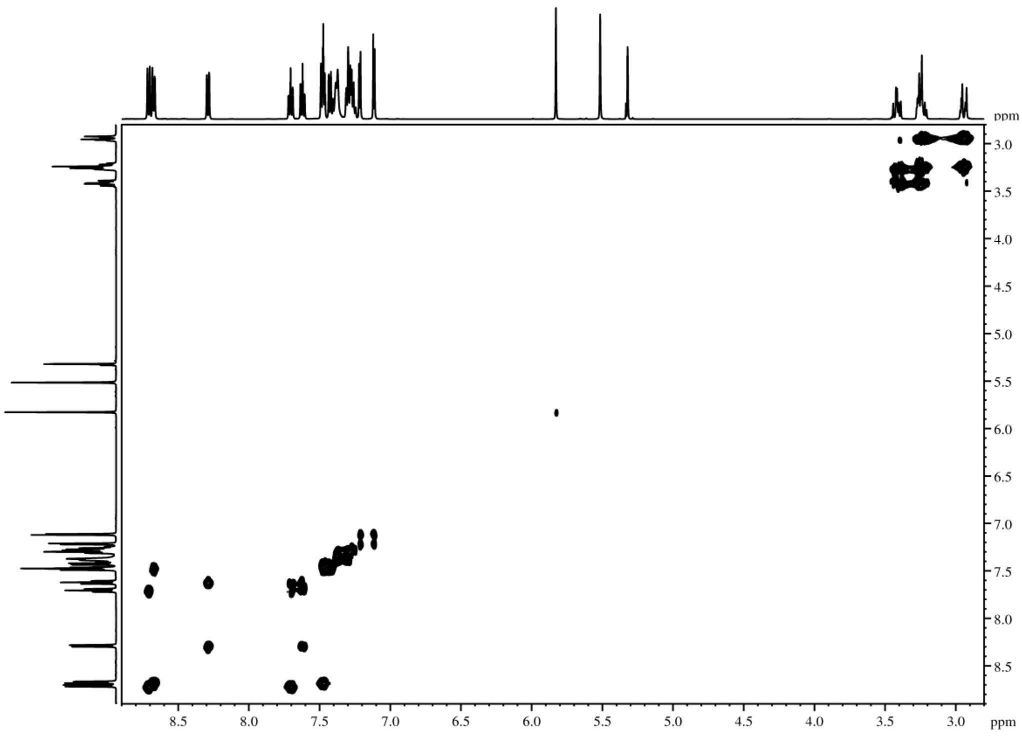

Figure S84. H,H-COSY NMR spectrum of **15**

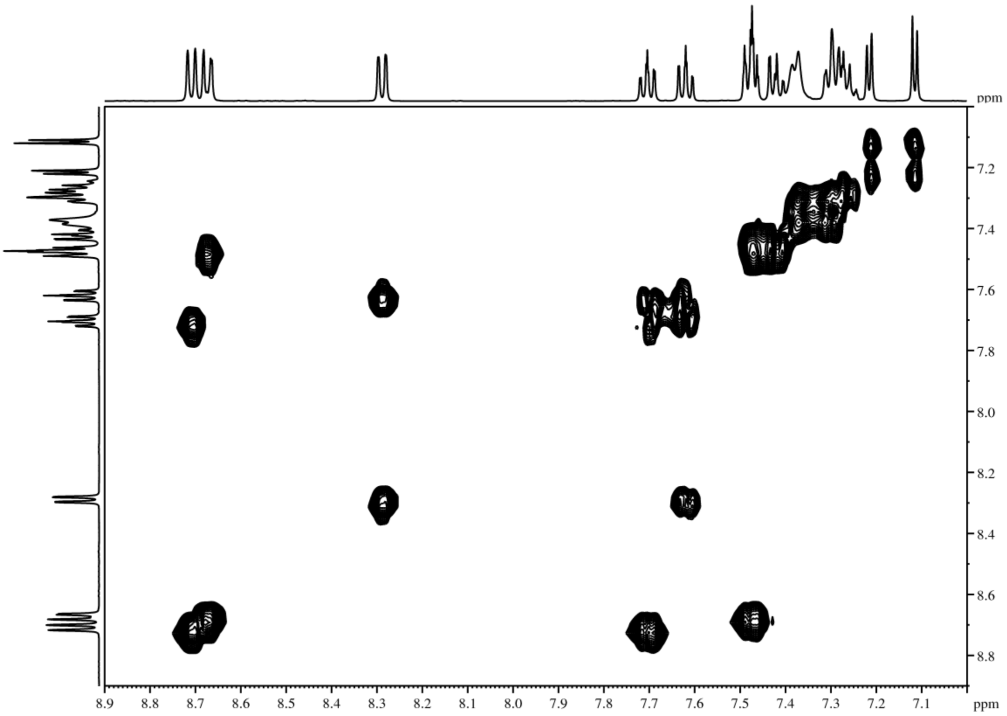

Figure S85. H,H-COSY NMR spectrum of **15**, expansion

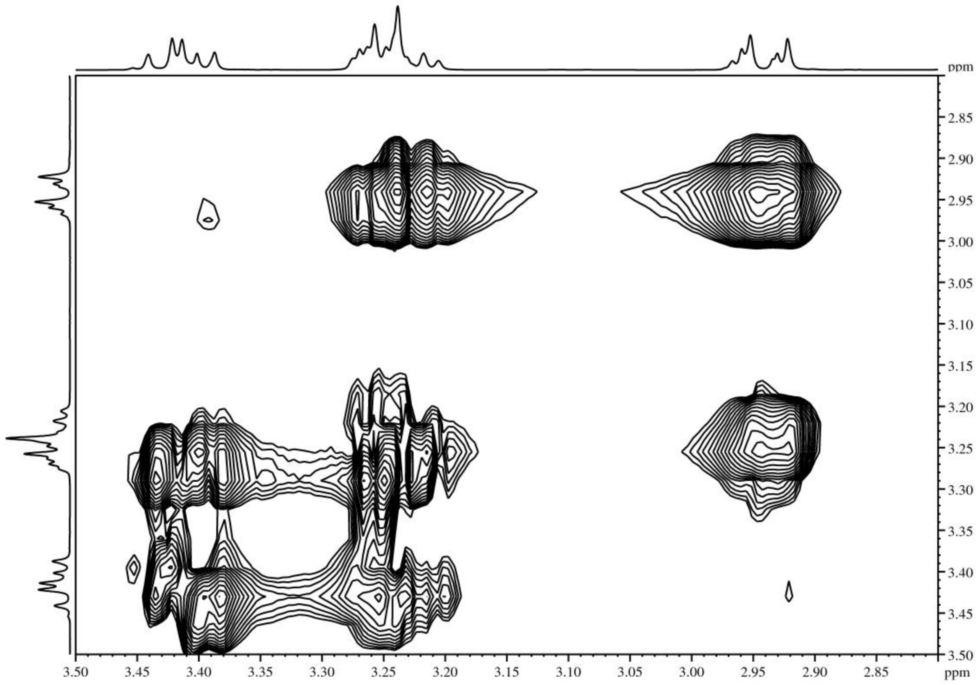

Figure S86. H,H-COSY NMR spectrum of **15**, expansion

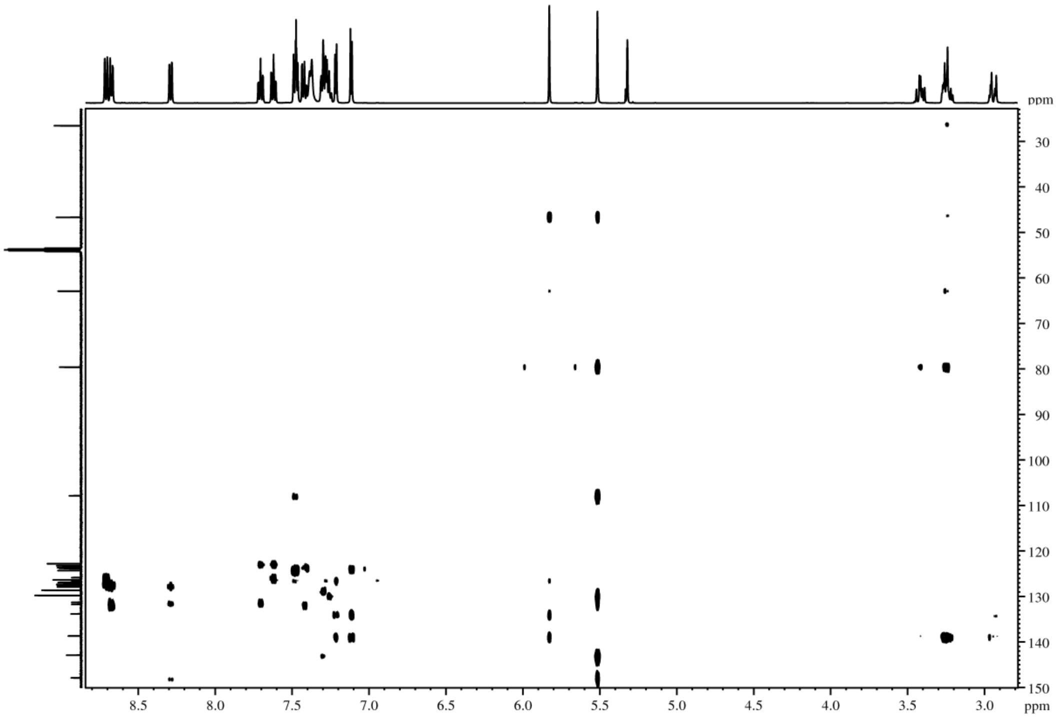

Figure S87. HMBC NMR spectrum of **15**

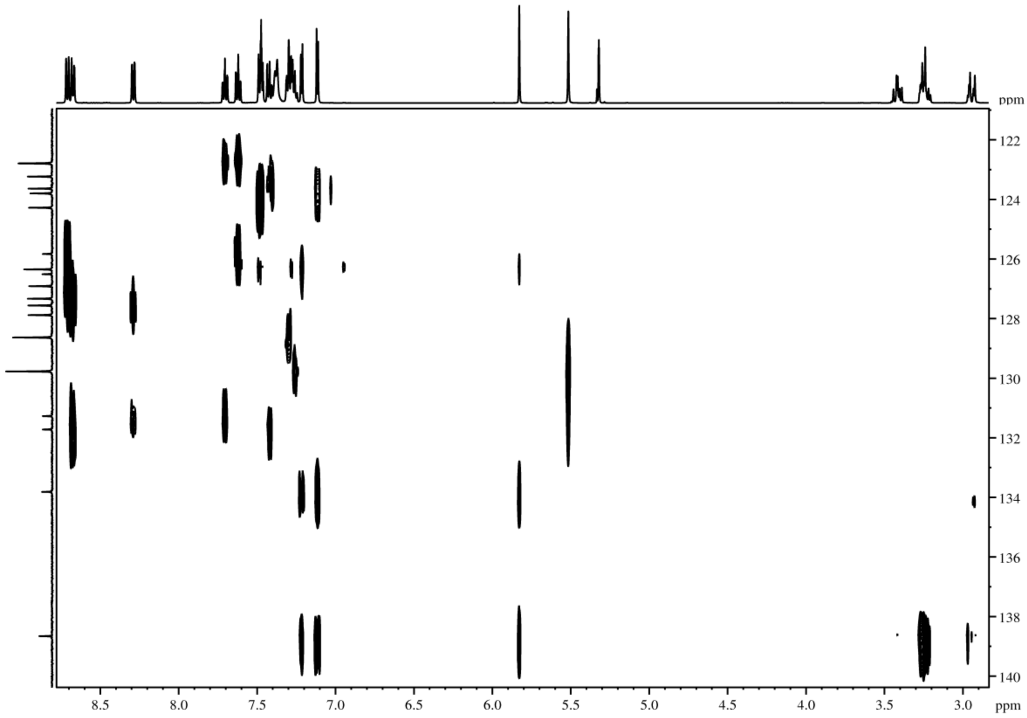

Figure S88. HMBC NMR spectrum of **15**, expansion

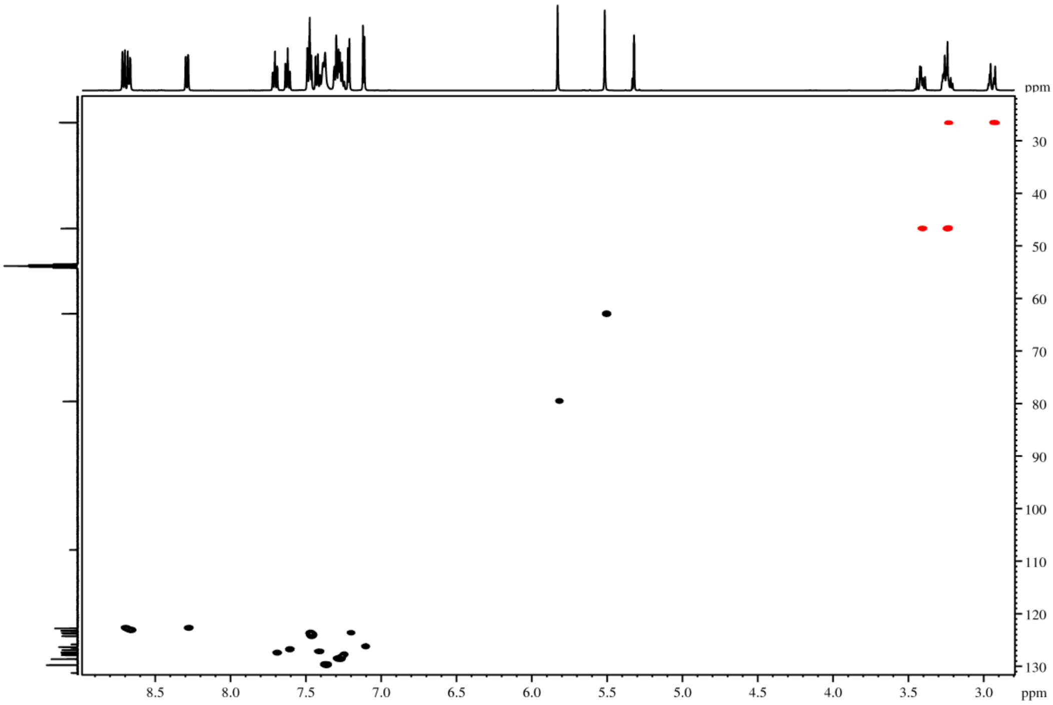

Figure S89. edited HSQC NMR spectrum of **15** (black: positive, red: negative signals)

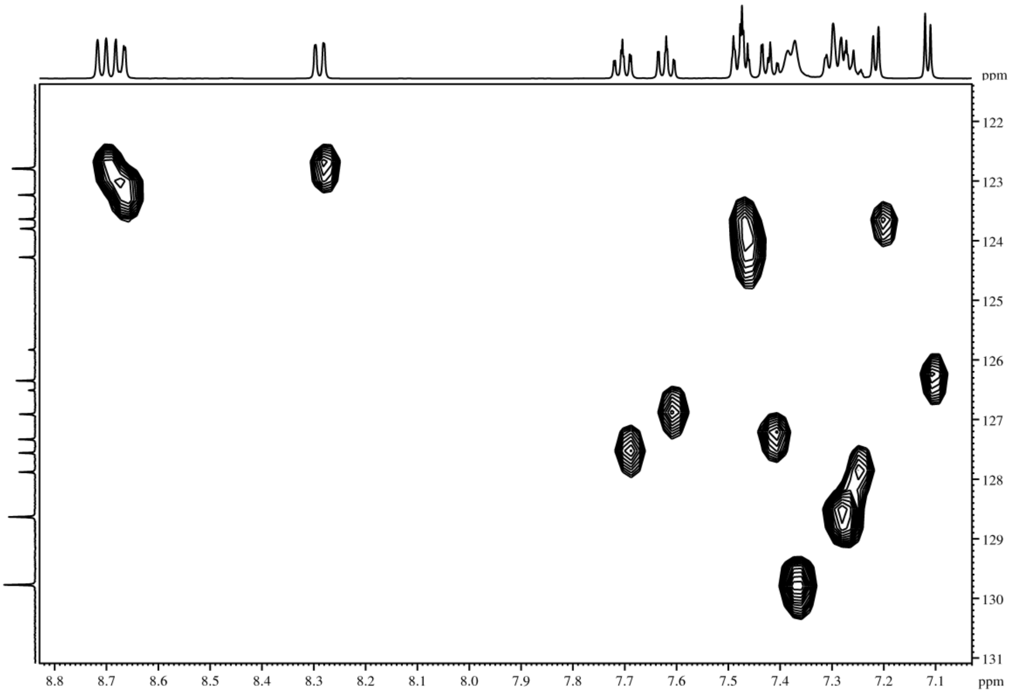

Figure S90. edited HSQC NMR spectrum of **15**, expansion (black: positive, red: negative signals)

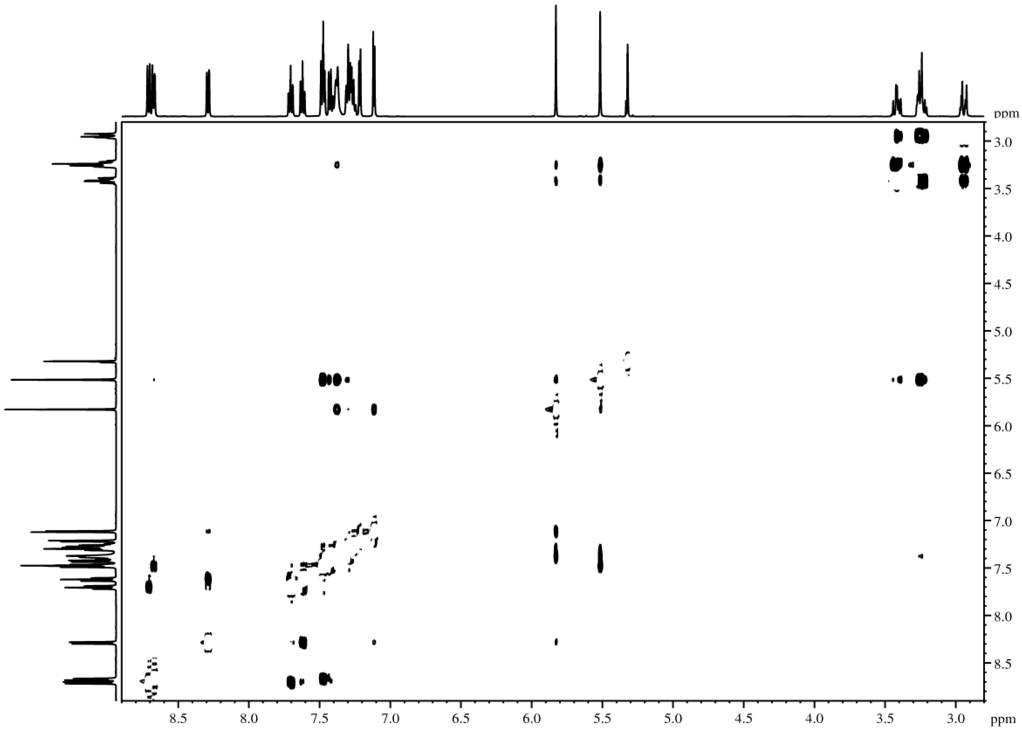

Figure S91. Phase-sensitive NOESY NMR spectrum of **15** (only positive signals, mixing time 800 ms)

9aR*,18S*-19-(Phenyl)-phenanthr[9,10-e]oxazino[2,3-a]-β-carboline (16).

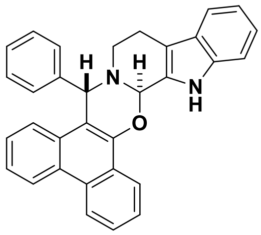

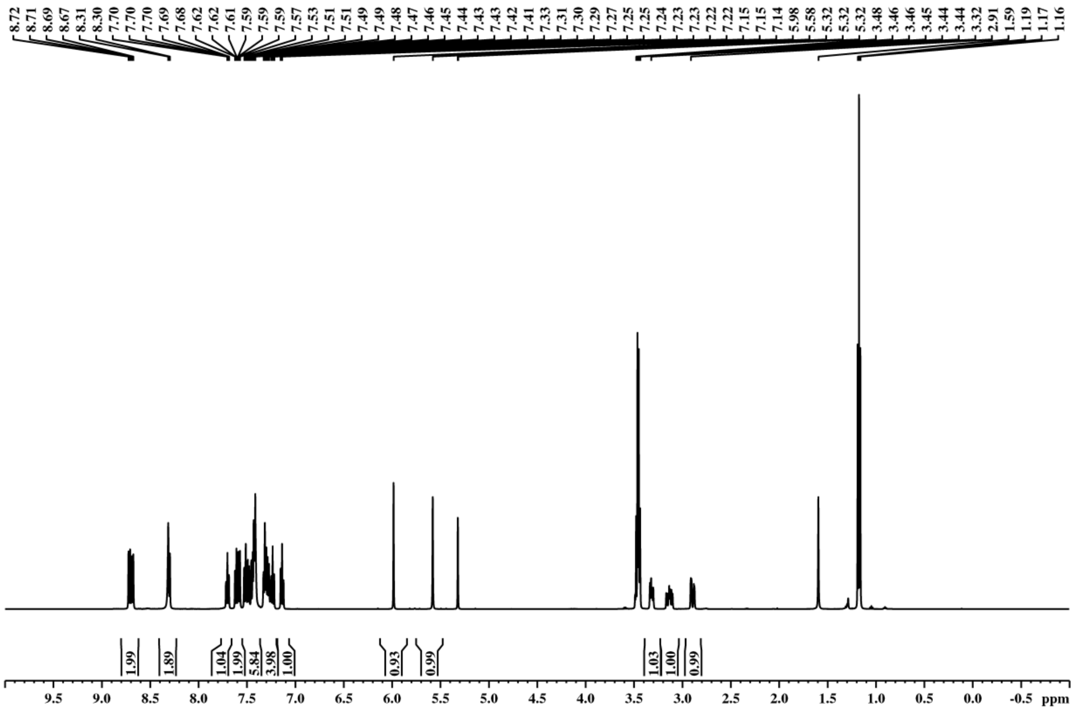

Figure S92. ^{1}H NMR spectrum of **16**

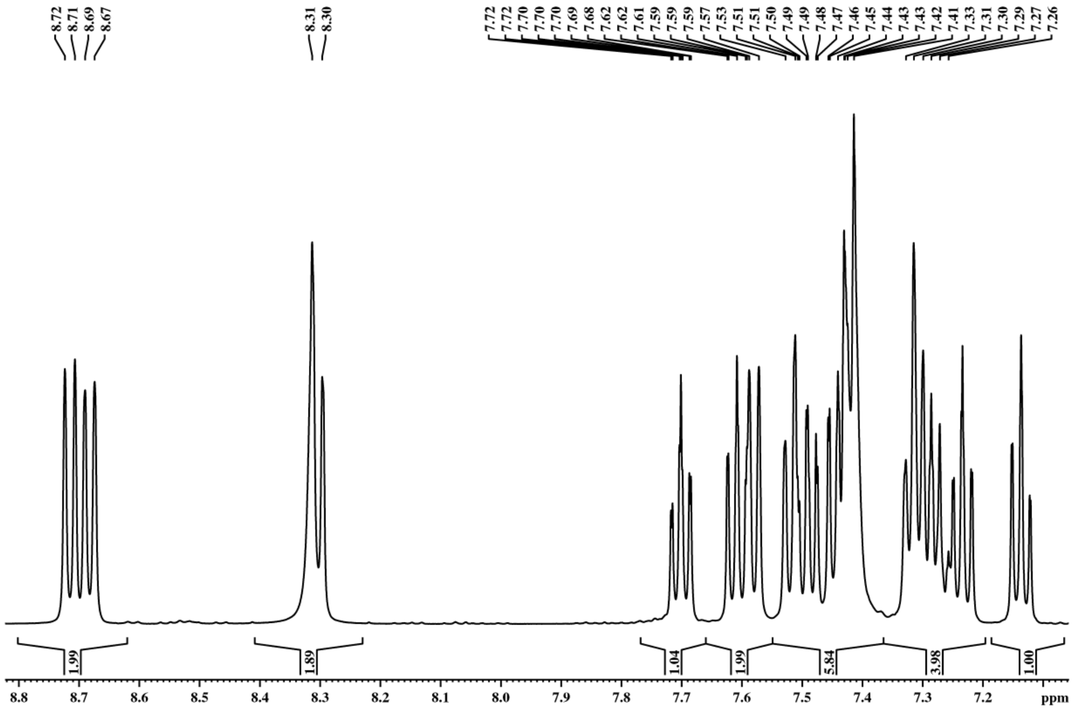

Figure S93. ^{1}H NMR spectrum of **16**, expansion

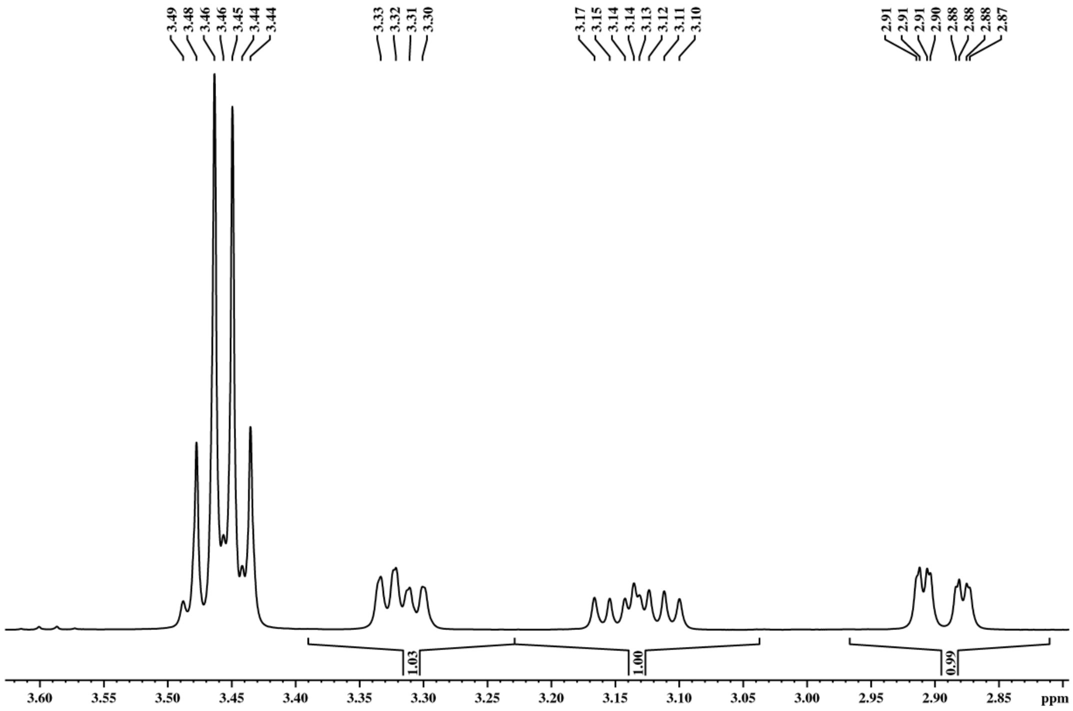

Figure S94. ^{1}H NMR spectrum of **16**, expansion

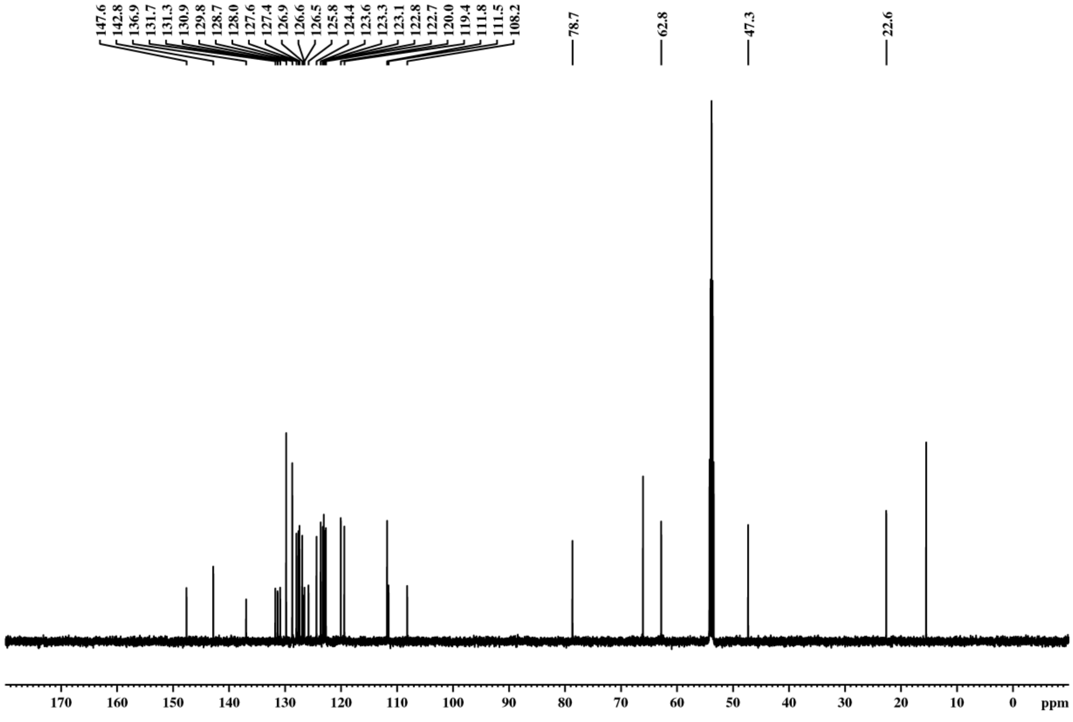

Figure S95. ^{13}C NMR spectrum of **16**

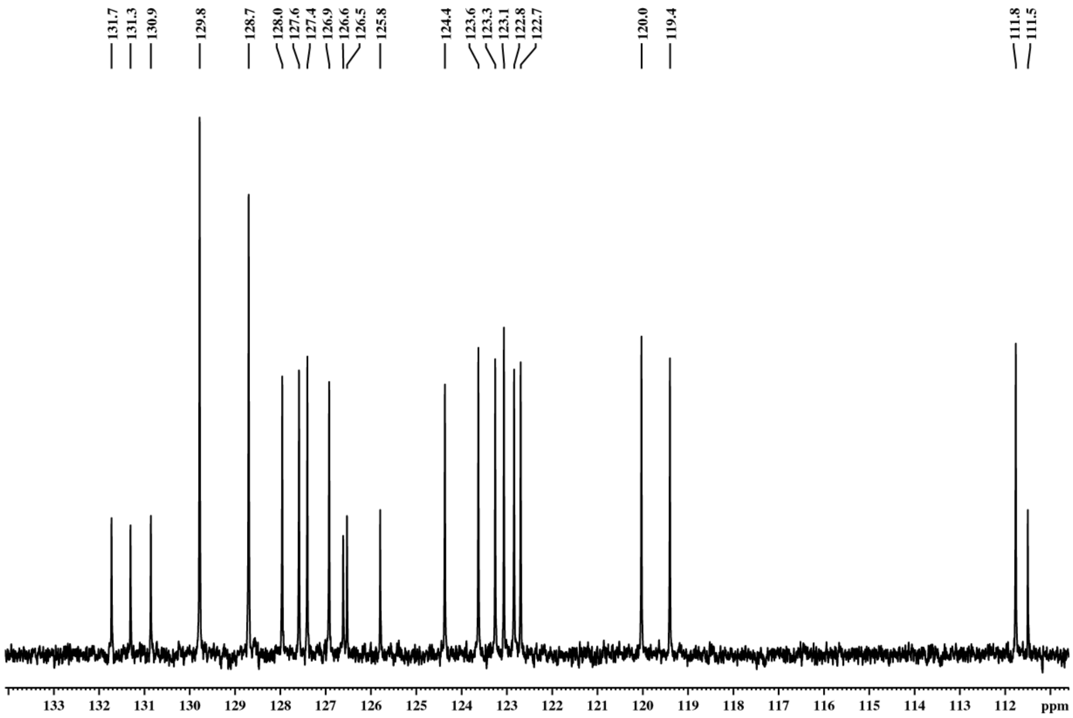

Figure S96. ^{13}C NMR spectrum of **16**, expansion

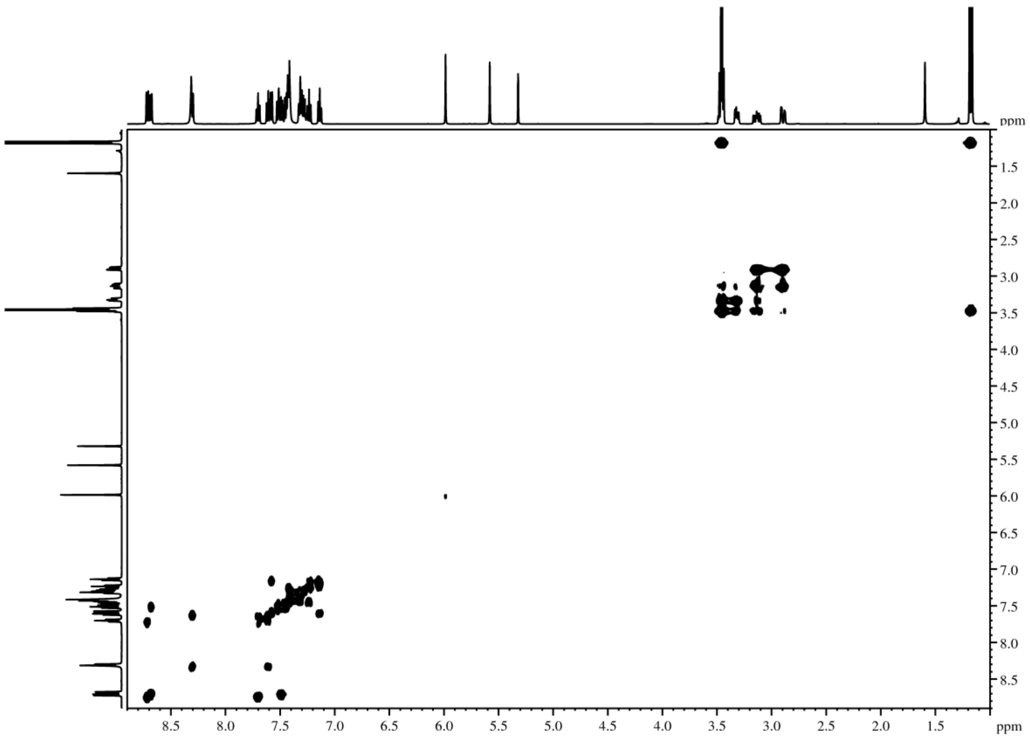

Figure 97. H,H-COSY NMR spectrum of **16**

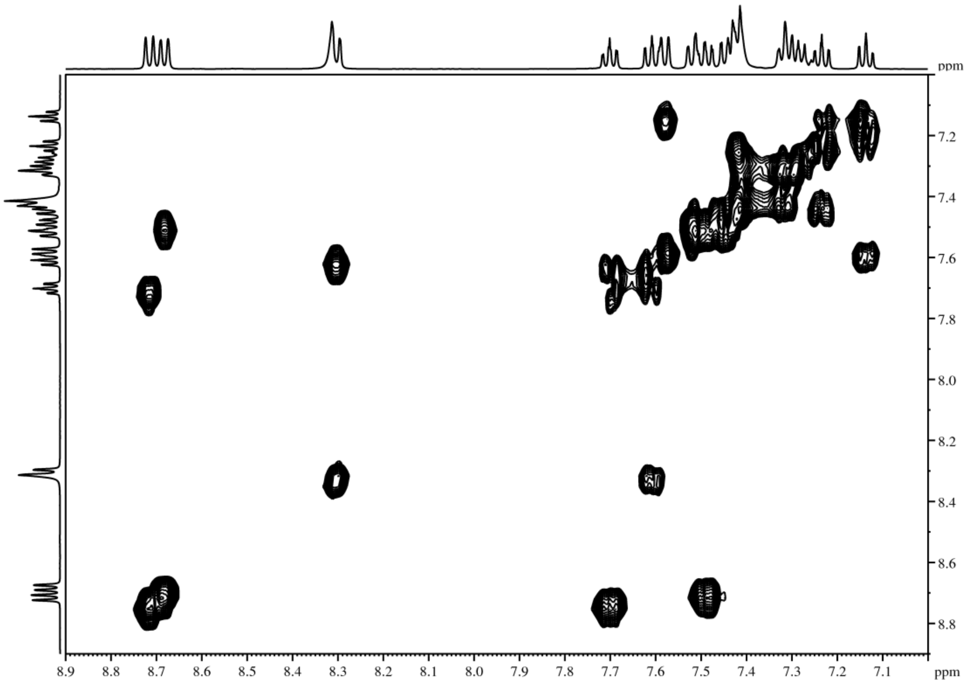

Figure S98. H,H-COSY NMR spectrum of **16**, expansion

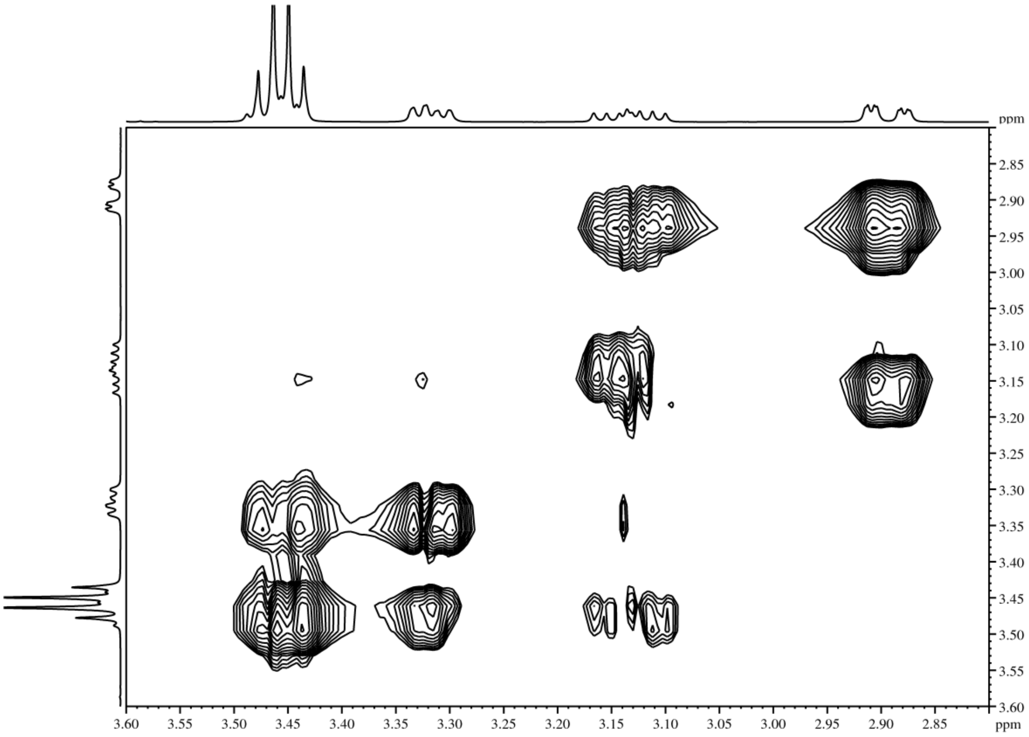

Figure S99. H,H-COSY NMR spectrum of **16**, expansion

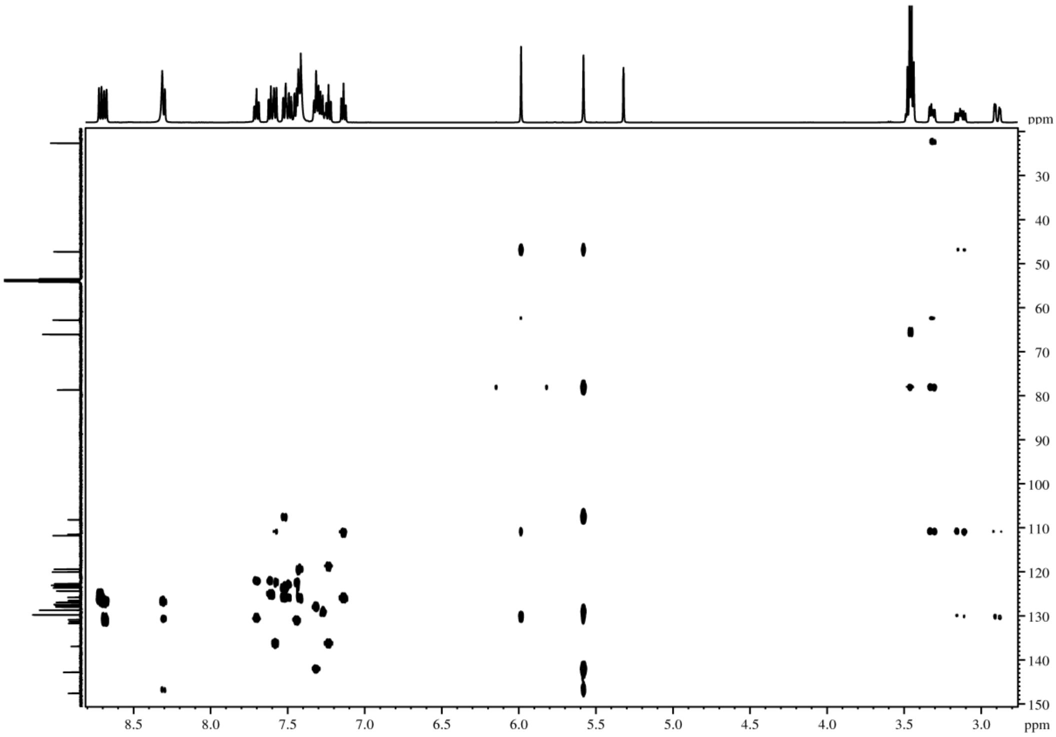

Figure S100. HMBC NMR spectrum of **16**

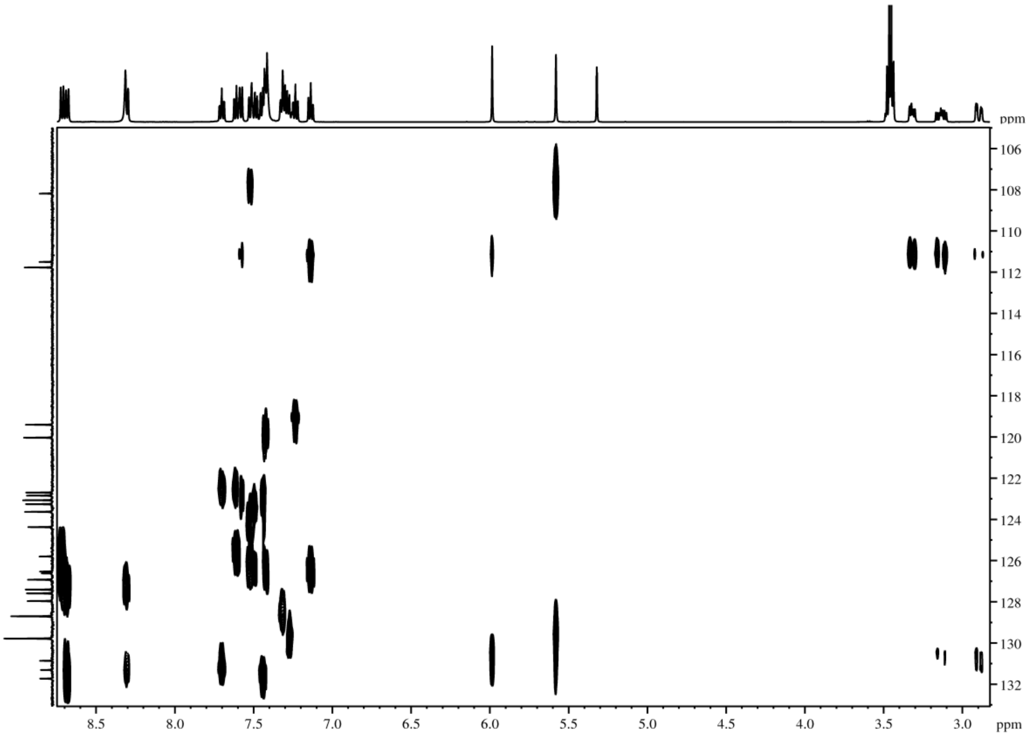

Figure S101. HMBC NMR spectrum of **16**, expansion

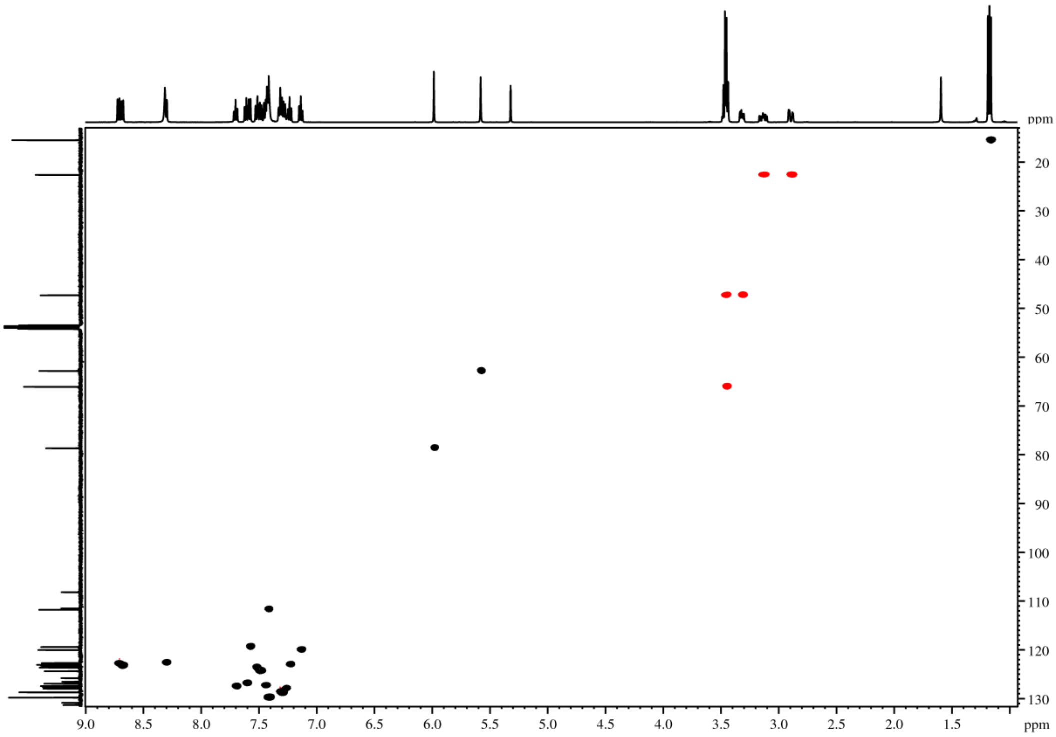

Figure S102. edited HSQC NMR spectrum of **16** (black: positive, red: negative signals)

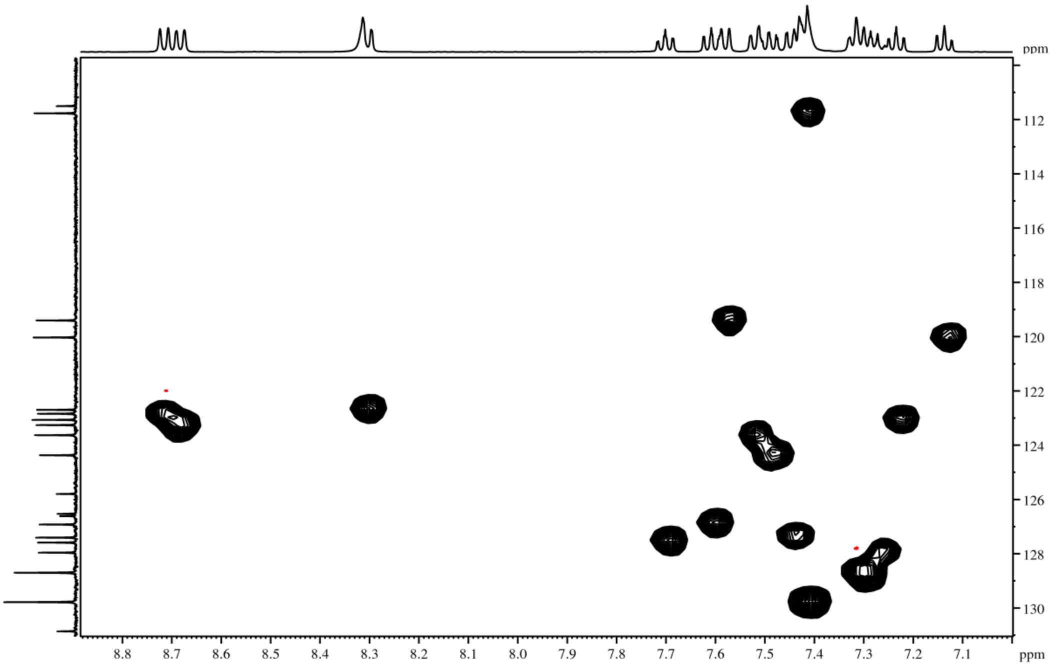

Figure S103. edited HSQC NMR spectrum of **16**, expansion (black: positive, red: negative signals)

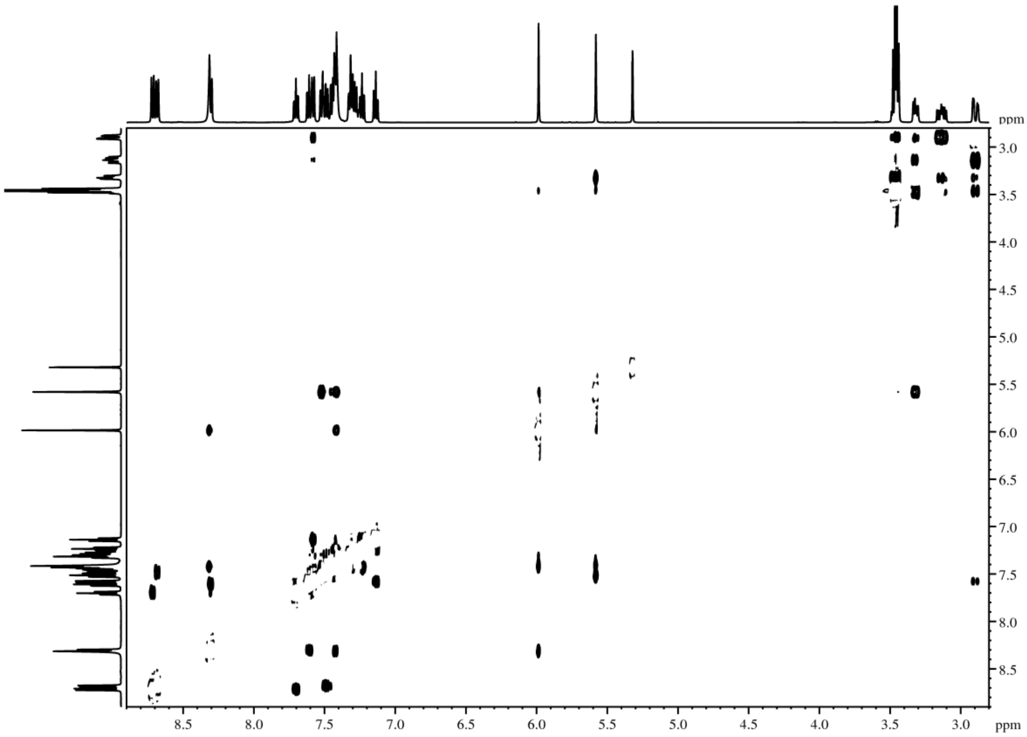

Figure S104. Phase-sensitive NOESY NMR spectrum of **16** (only positive signals, mixing time 800 ms)

14-Morpholin-4-yl-dibenzo[a,c]xanthene (19)

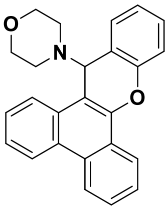

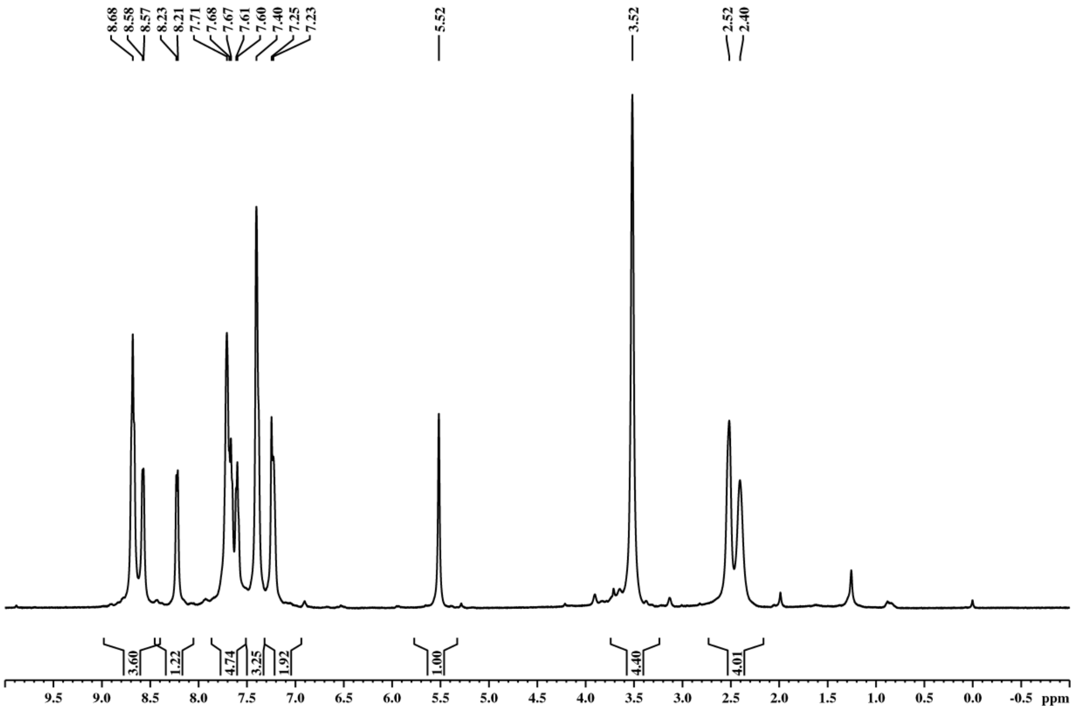

Figure S105. ^{1}H NMR spectrum of **19**

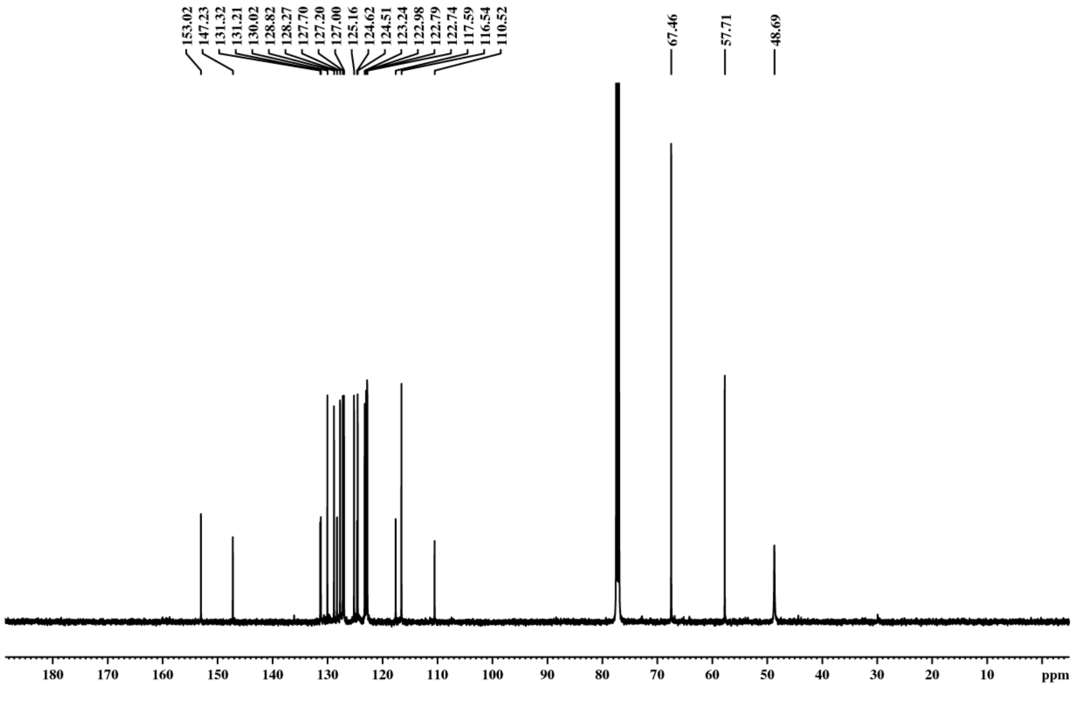

Figure S106. ^{13}C NMR spectrum of **19**

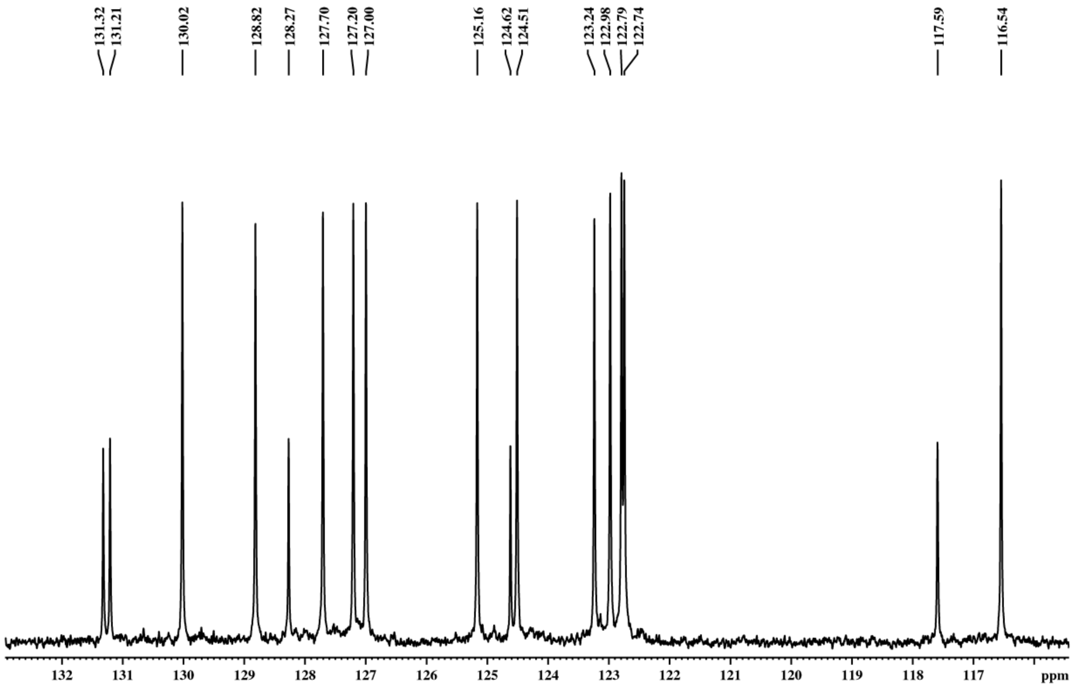

Figure S107. ^{13}C NMR spectrum of **19**, expansion

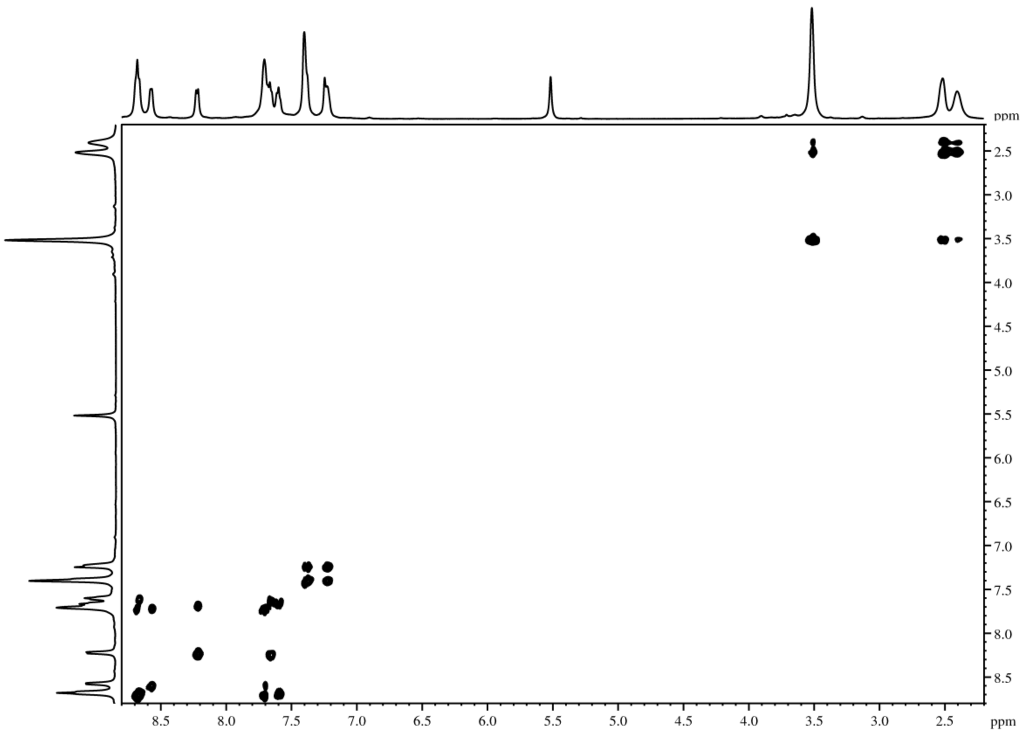

Figure S108. H,H-COSY NMR spectrum of **19**

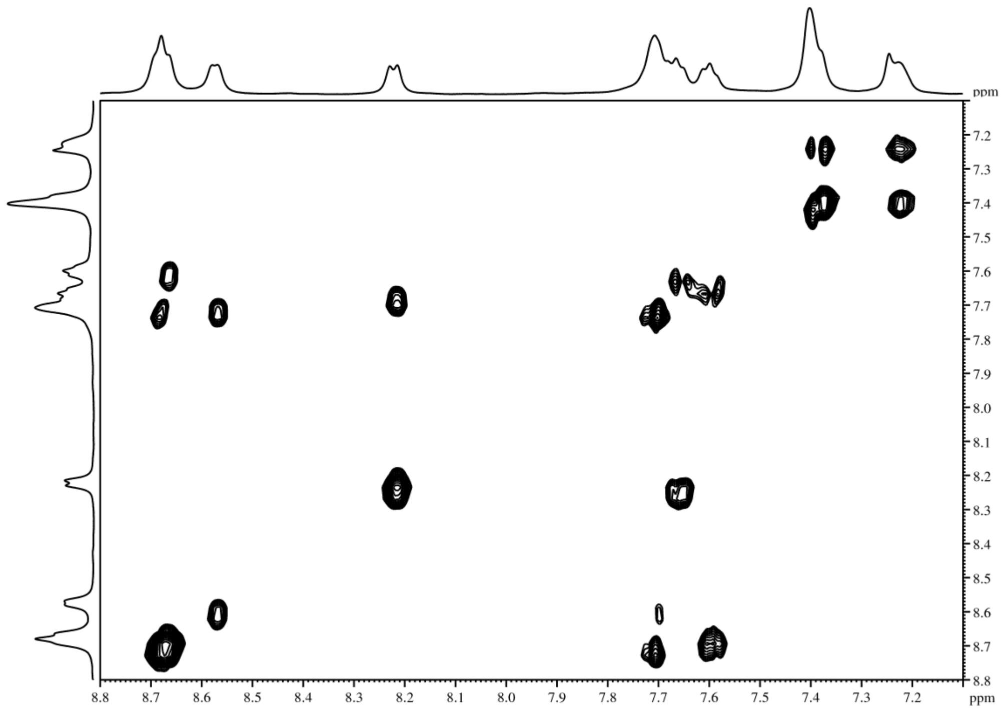

Figure S109. H,H-COSY NMR spectrum of **19**, expansion

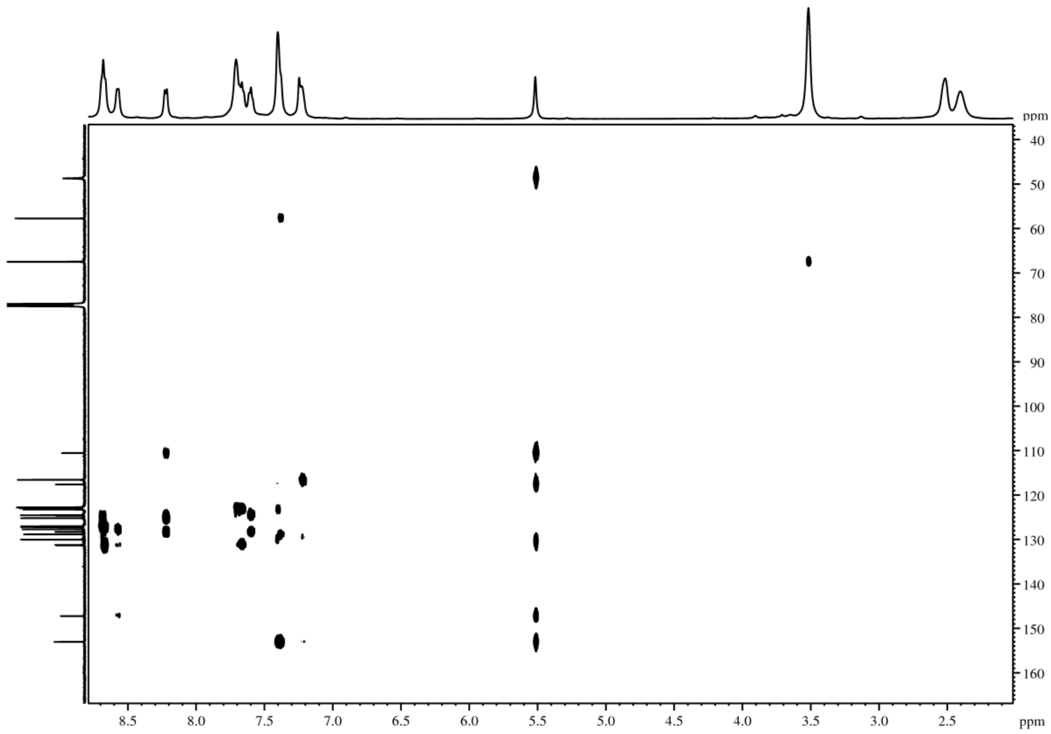

Figure S110. HMBC NMR spectrum of **19**

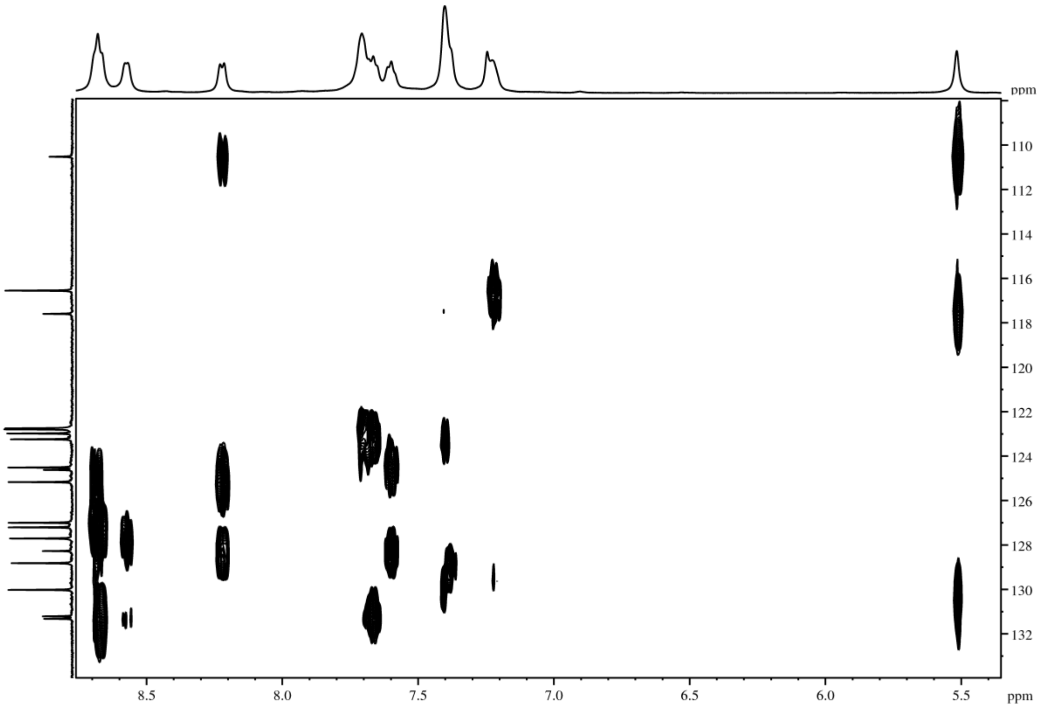

Figure S111. HMBC NMR spectrum of **19**, expansion

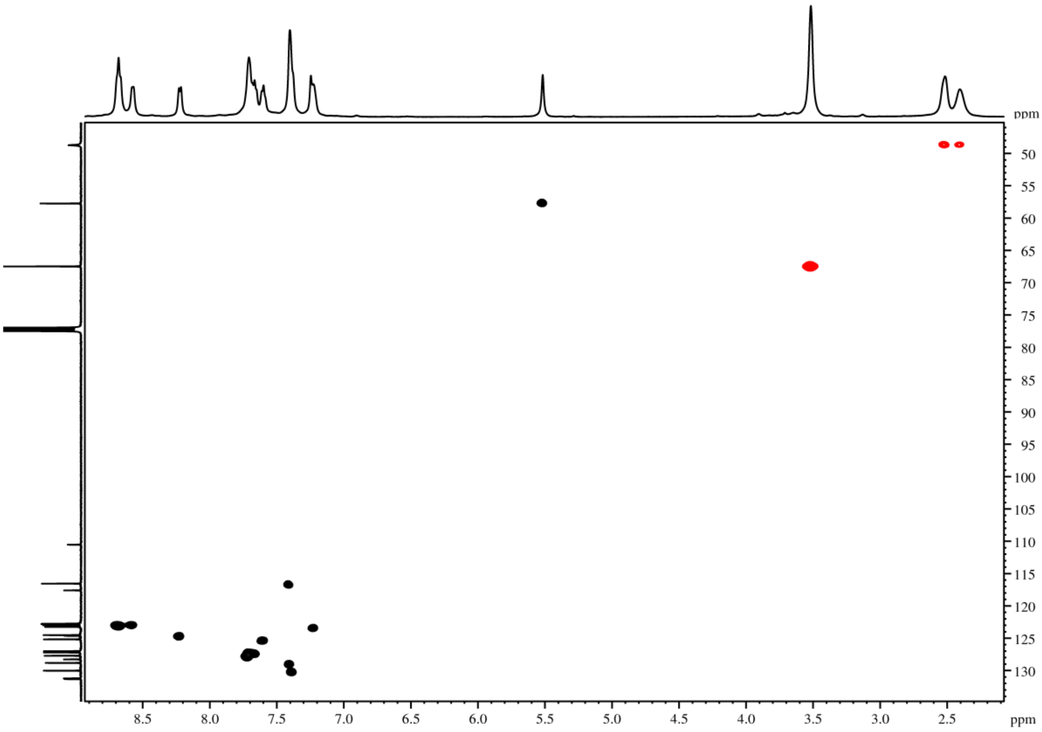

Figure S112. edited HSQC NMR spectrum of **19** (black: positive, red: negative signals)

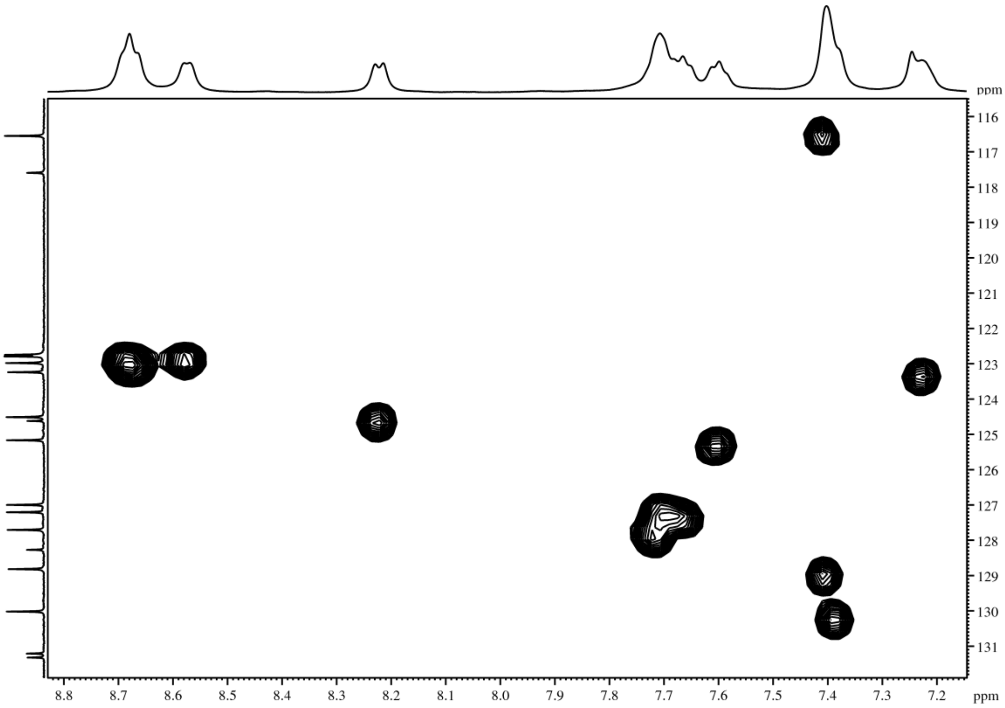

Figure S113. edited HSQC NMR spectrum of **19**, expansion (black: positive, red: negative signals)

10-[(2-Hydroxyphenyl)-pyrrolidin-1-yl-methyl]-9- phenanthrol (21).

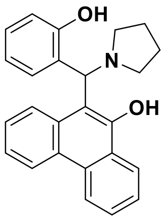

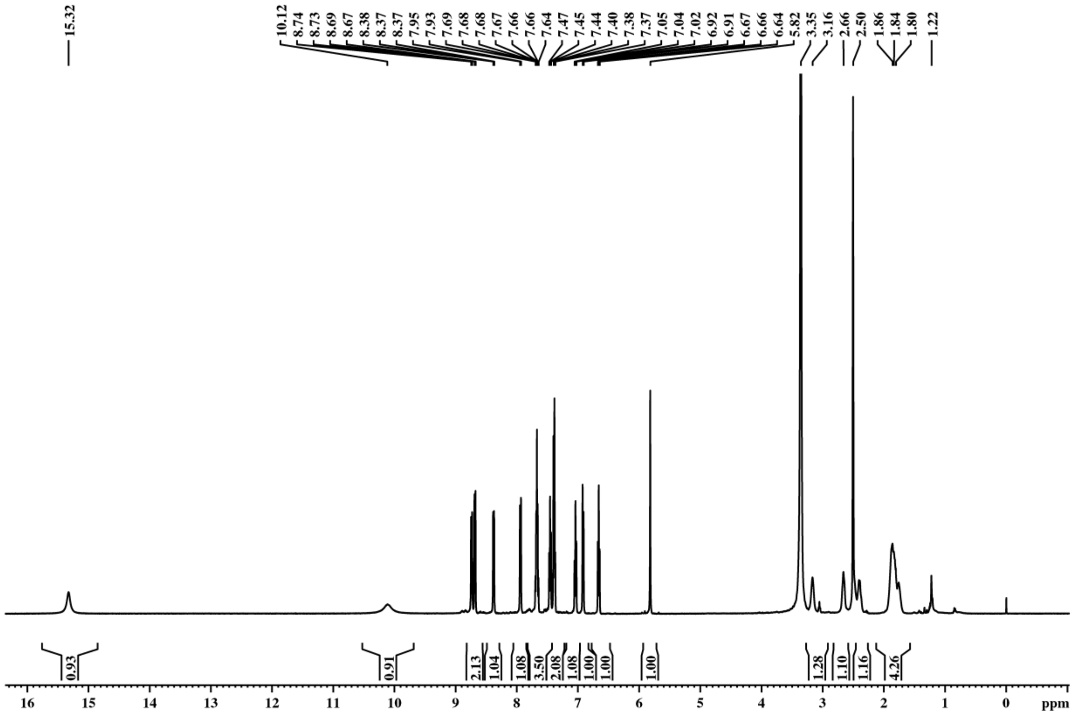

Figure S114. ^{1}H NMR spectrum of **21**

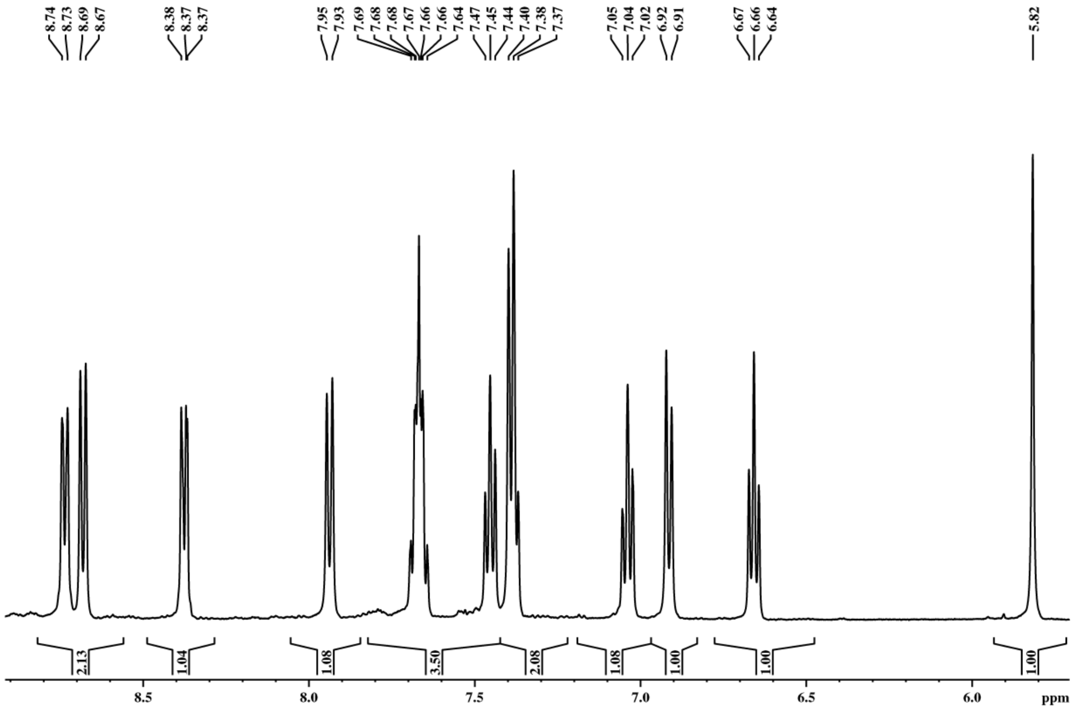

Figure S115. ^{1}H NMR spectrum of **21**, expansion

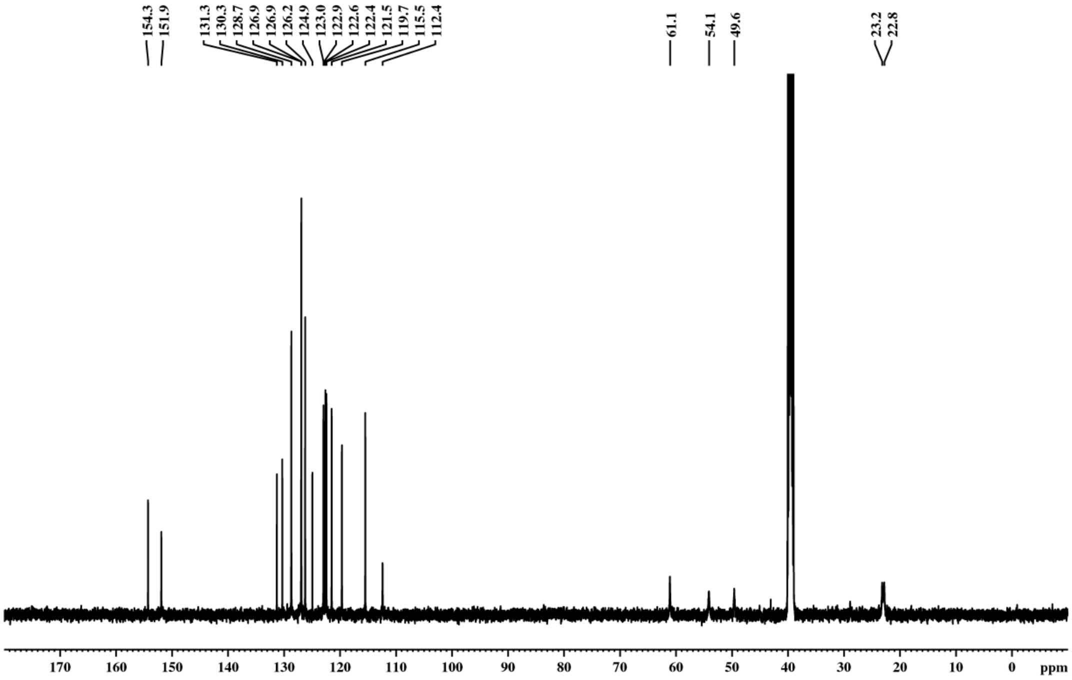

Figure S116. ^{13}C NMR spectrum of **21**

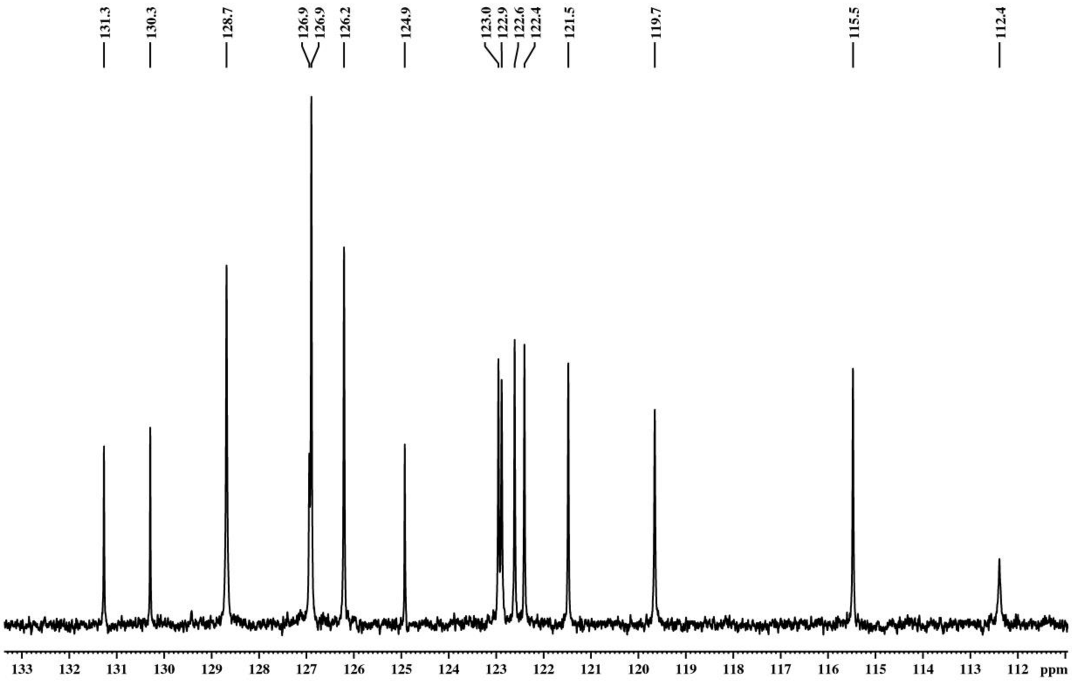

Figure S117. ^{13}C NMR spectrum of **21**, expansion

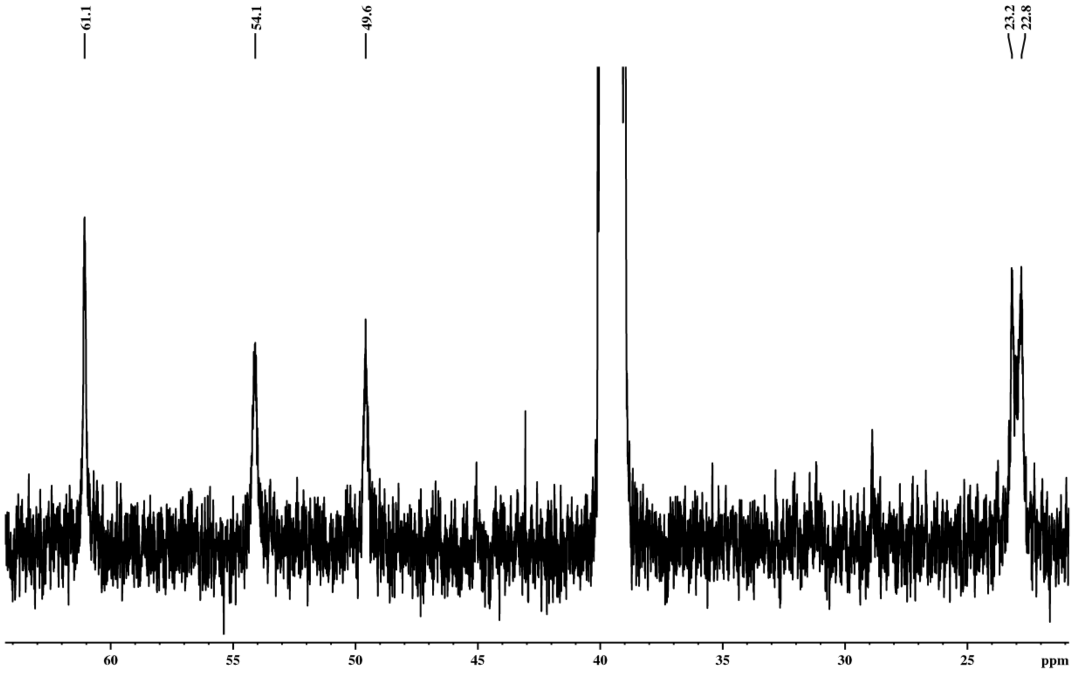

Figure S118. ^{13}C NMR spectrum of **21**, expansion

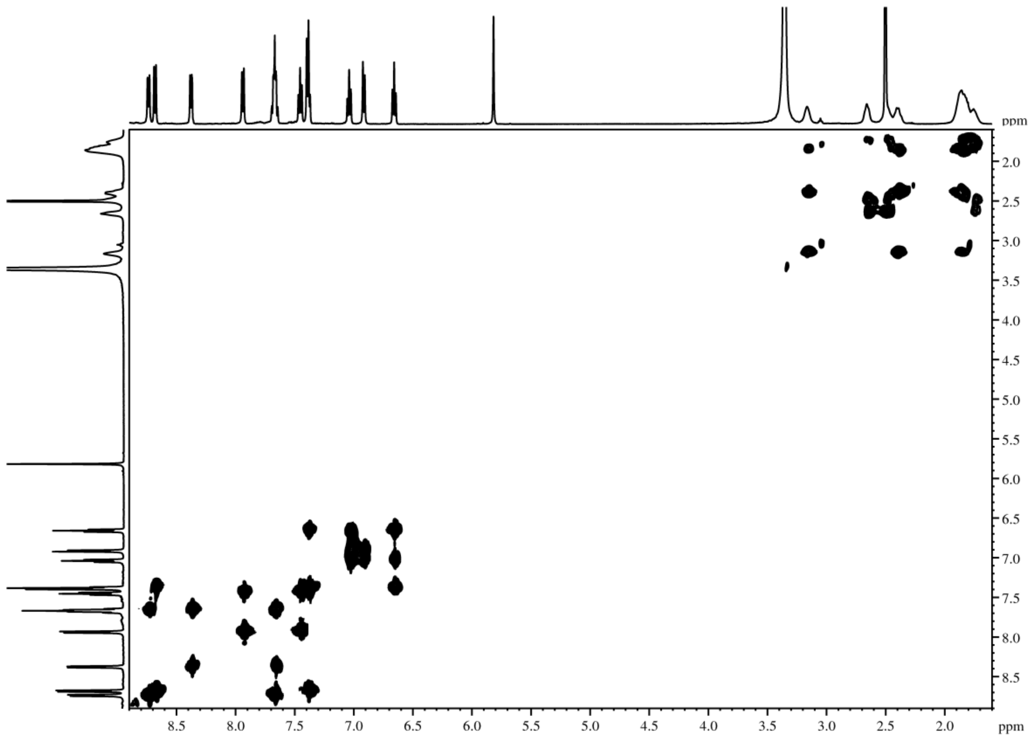

Figure S119. H,H-COSY NMR spectrum of **21**

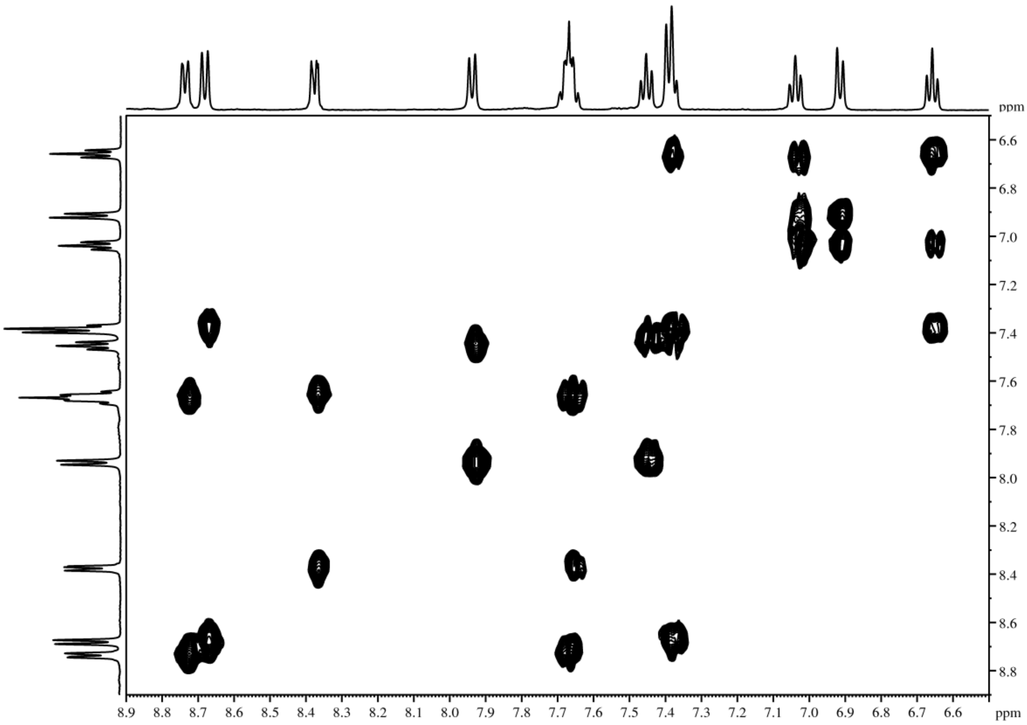

Figure S120. H,H-COSY NMR spectrum of **21**, expansion

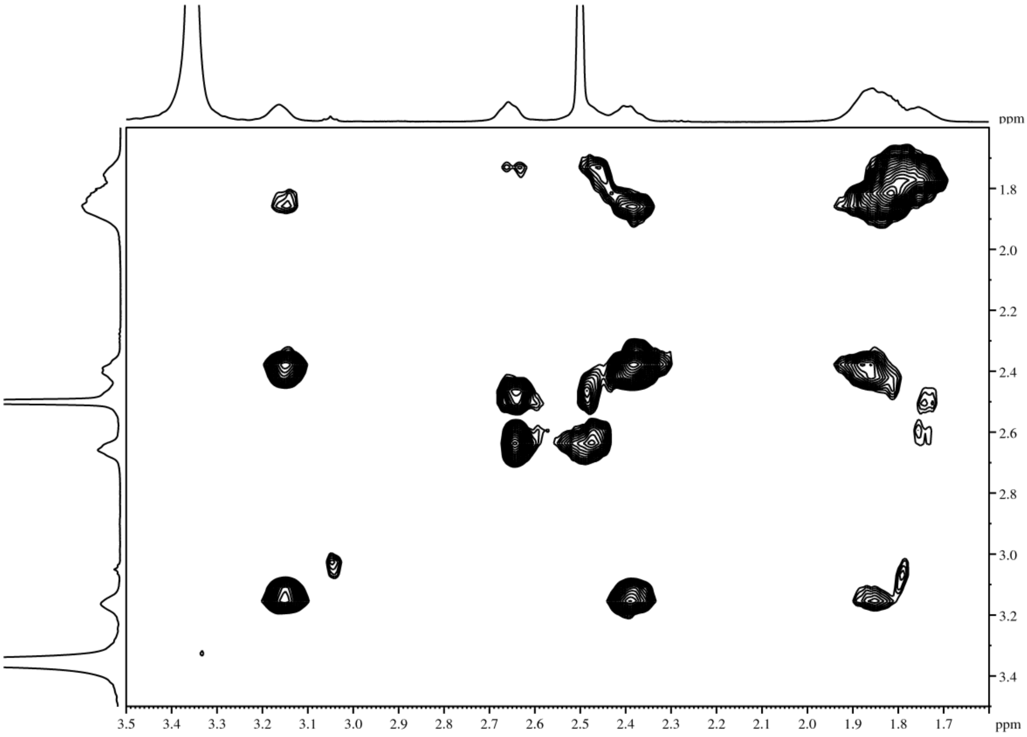

Figure S121. H,H-COSY NMR spectrum of **21**, expansion

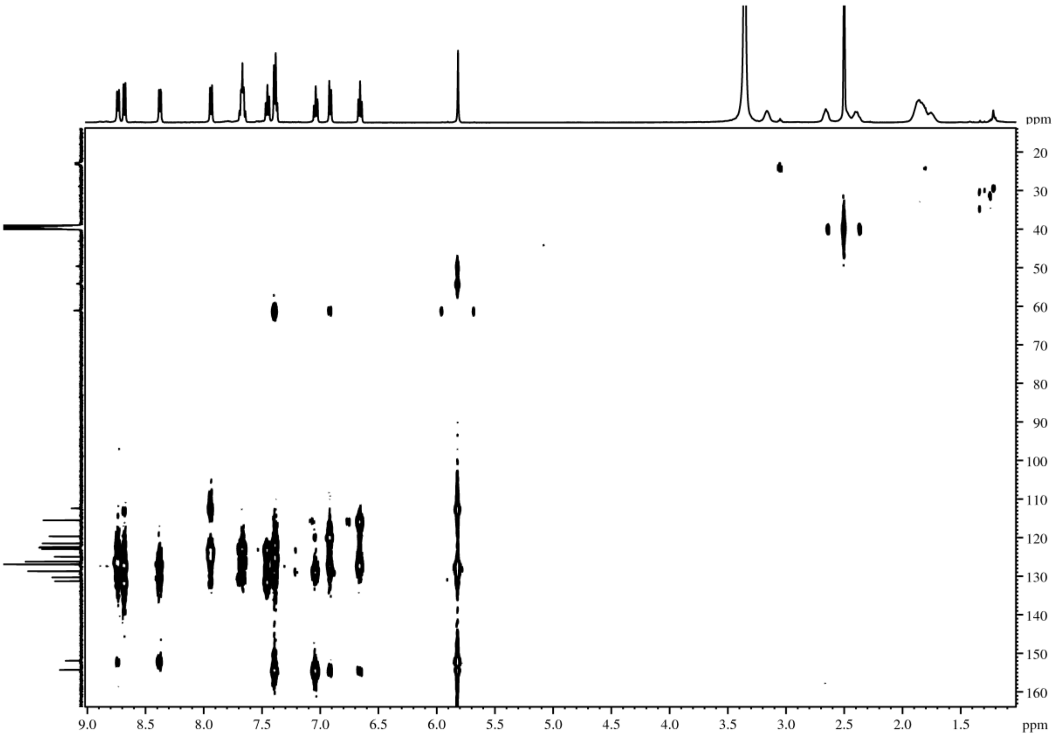

Figure S122. HMBC NMR spectrum of **21**

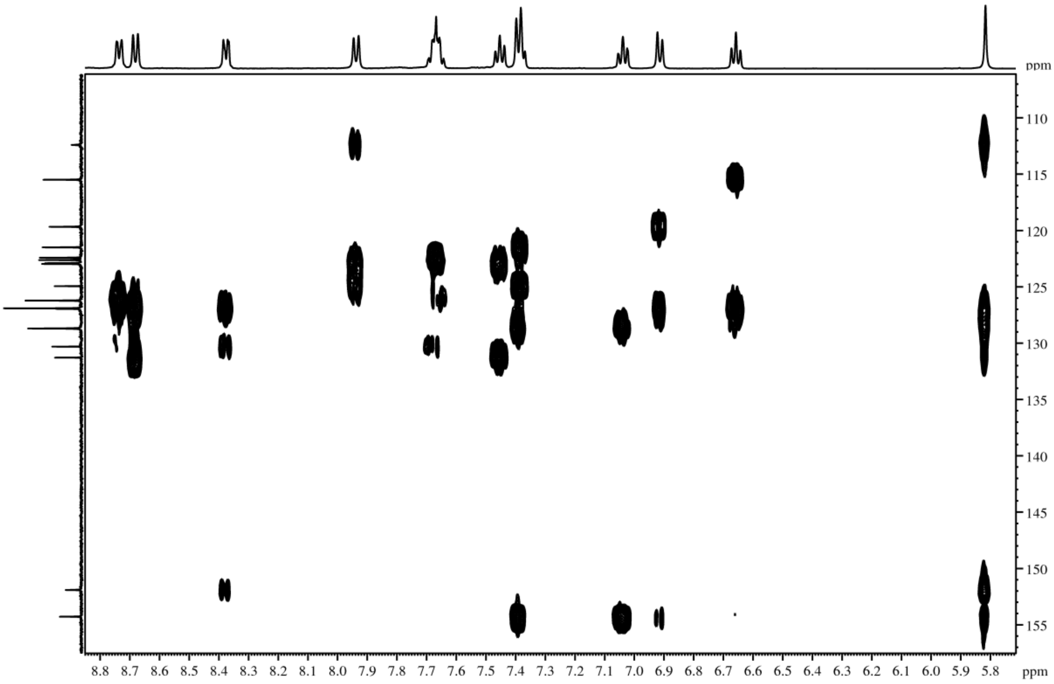

Figure S123. HMBC NMR spectrum of **21**, expansion

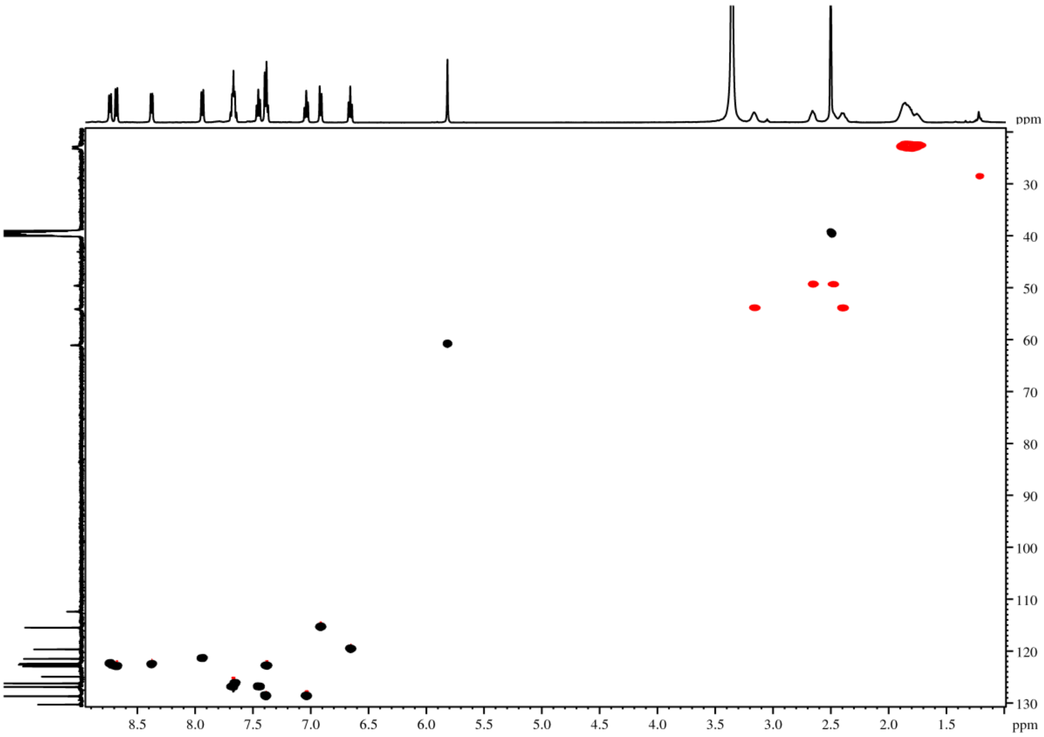

Figure S124. edited HSQC NMR spectrum of **21** (black: positive, red: negative signals)

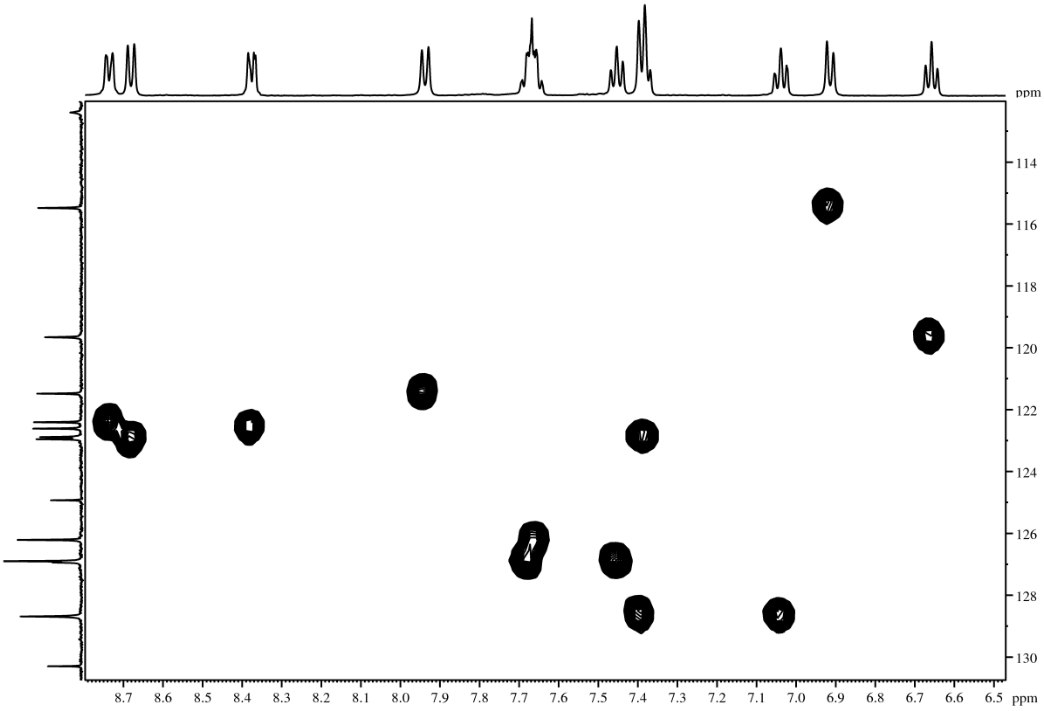

Figure S125. edited HSQC NMR spectrum of **21**, expansion (black: positive, red: negative signals)

9aR*,18S*-19-(2-Hydroxyphenyl)-phenanthr[9,10-e]oxazino[2,3-a]–β–carboline (24).

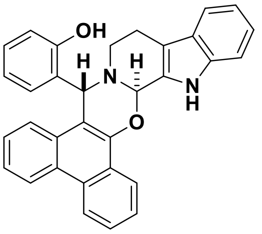

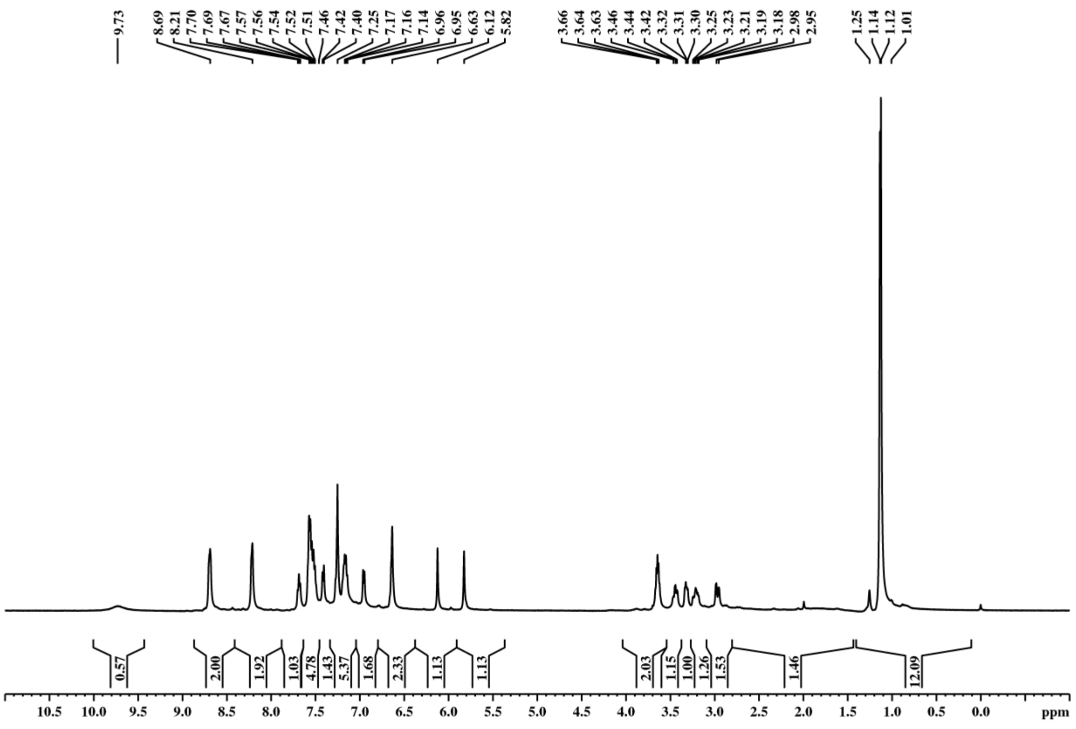

Figure S126. ^{1}H NMR spectrum of **24a**

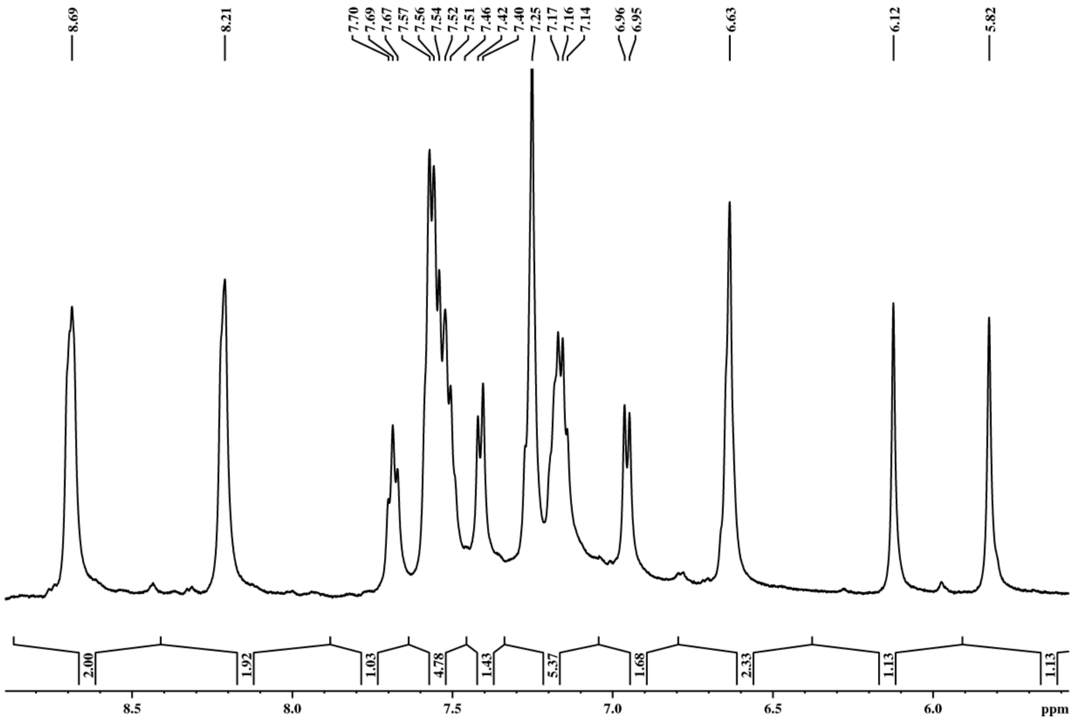

Figure S127. ^{1}H NMR spectrum of **24a**, expansion

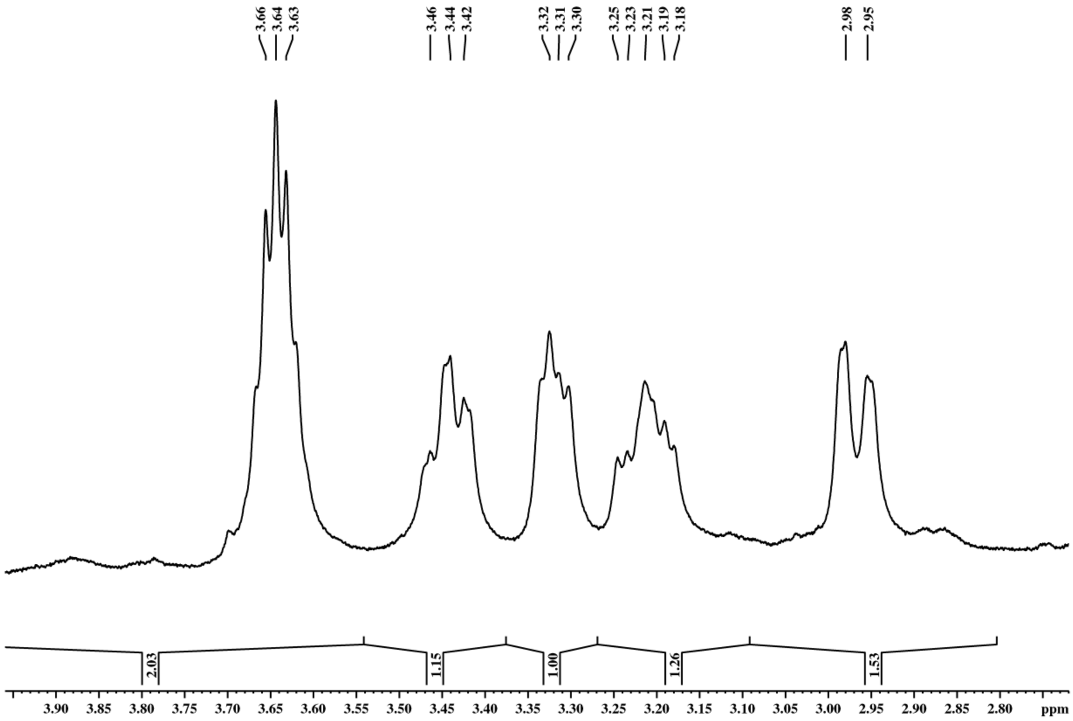

Figure S128. ^{1}H NMR spectrum of **24a**, expansion

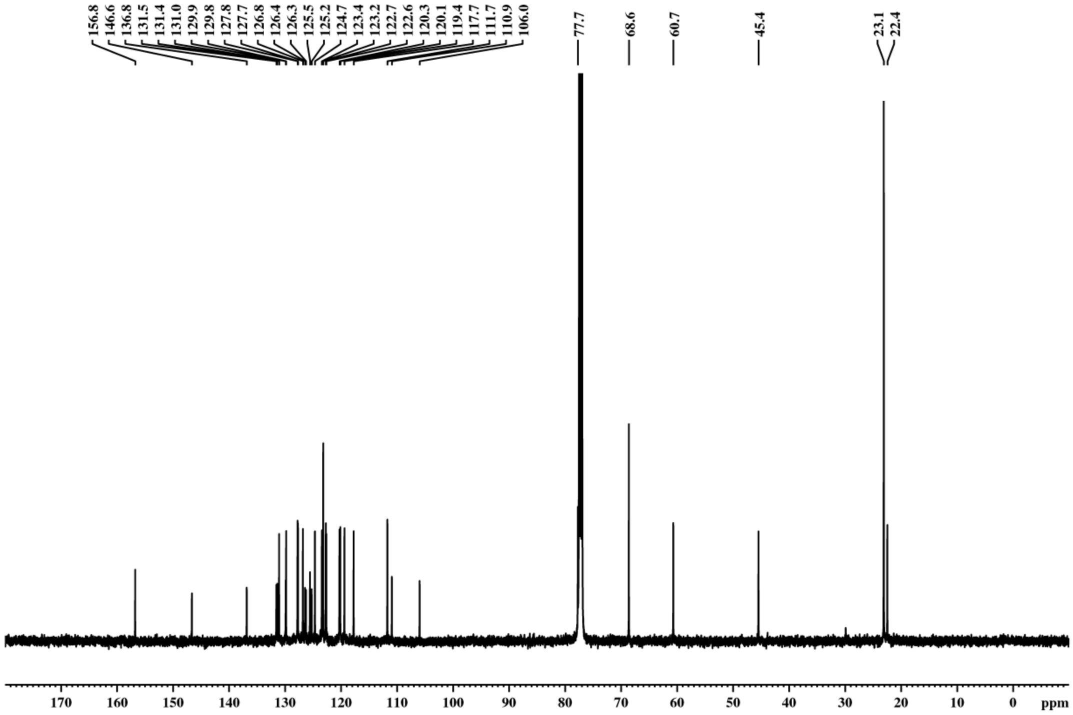

Figure S129. ^{13}C NMR spectrum of **24a**

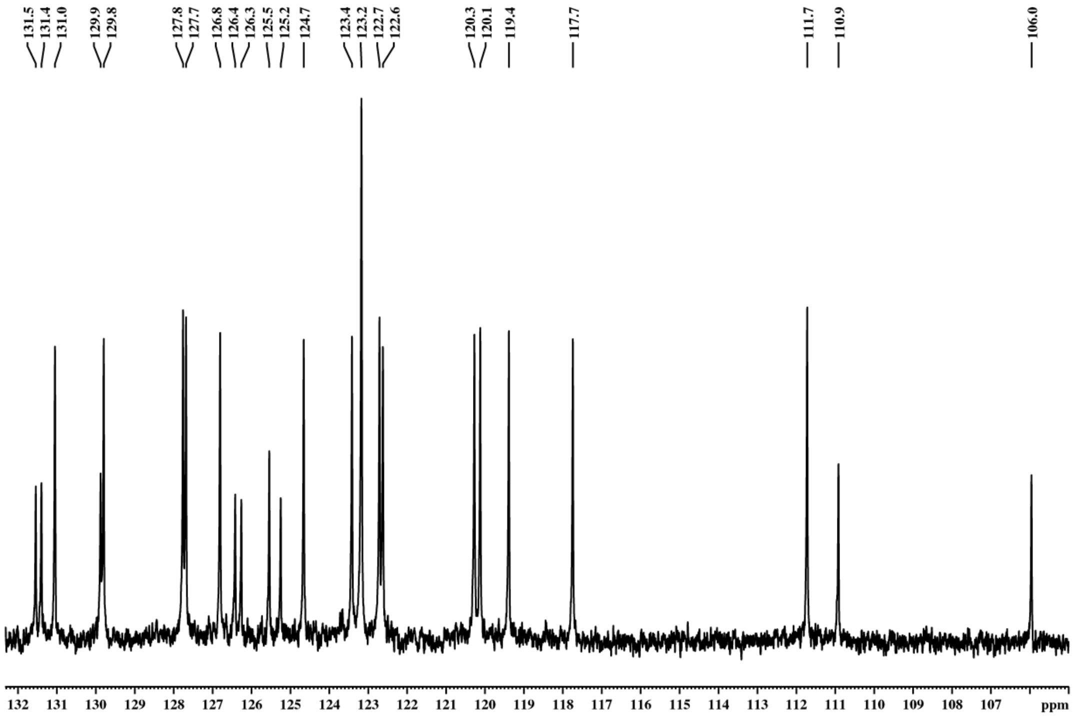

Figure S130. ^{13}C NMR spectrum of **24a**, expansion

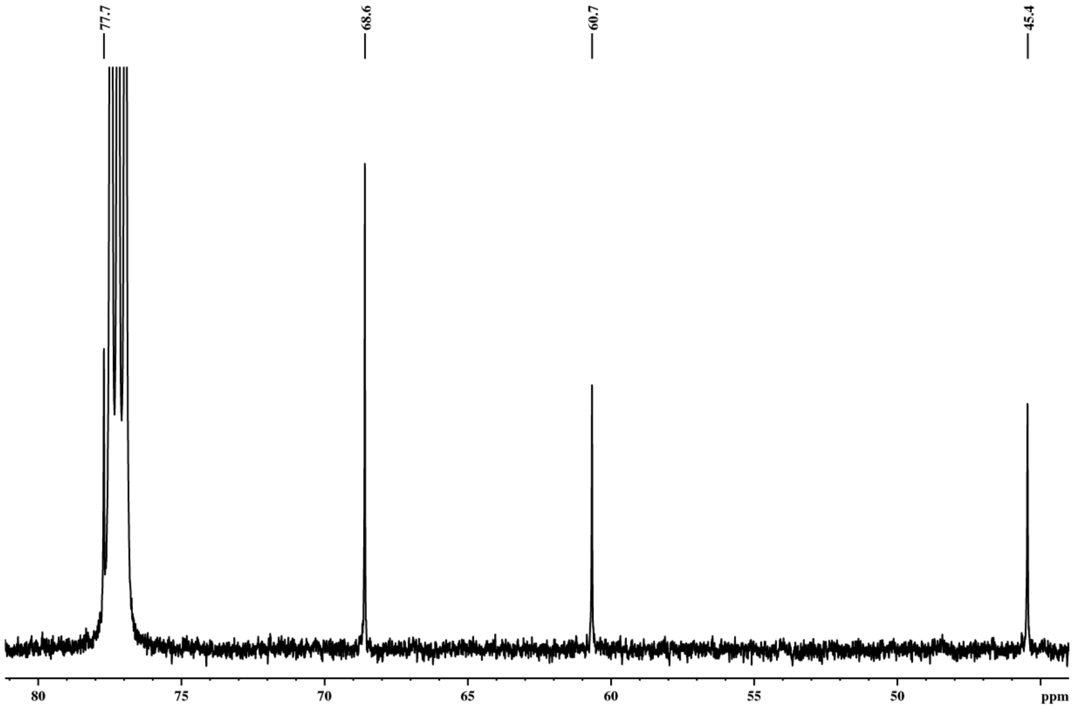

Figure S131. ^{13}C NMR spectrum of **24a**, expansion

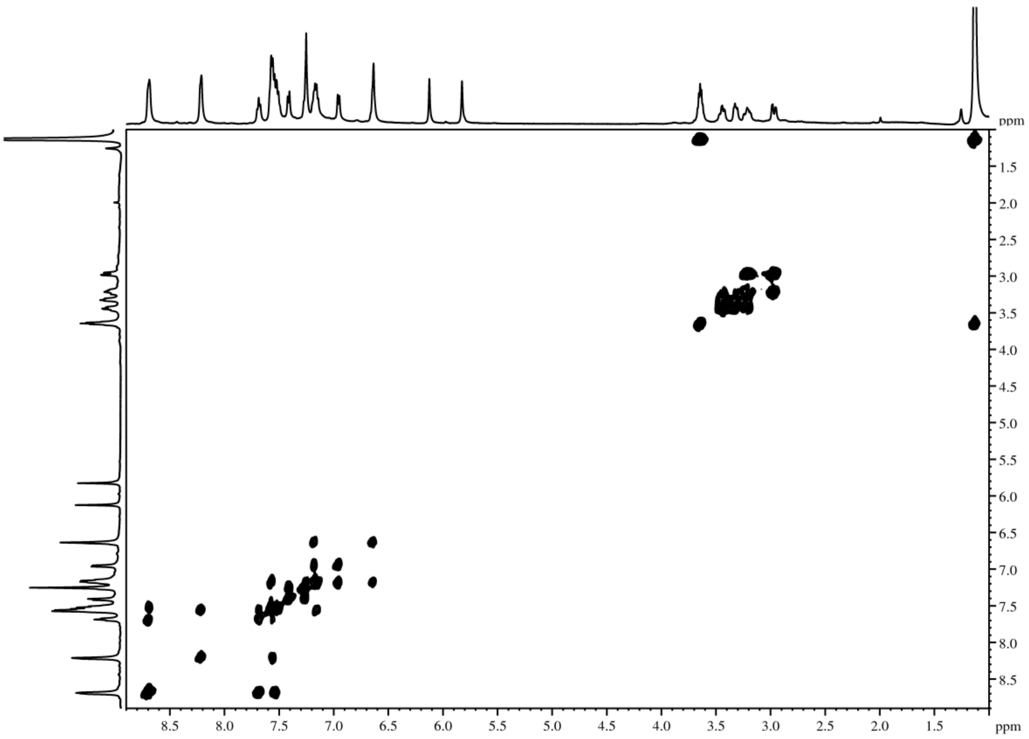

Figure S132. H,H-COSY NMR spectrum of **24a**

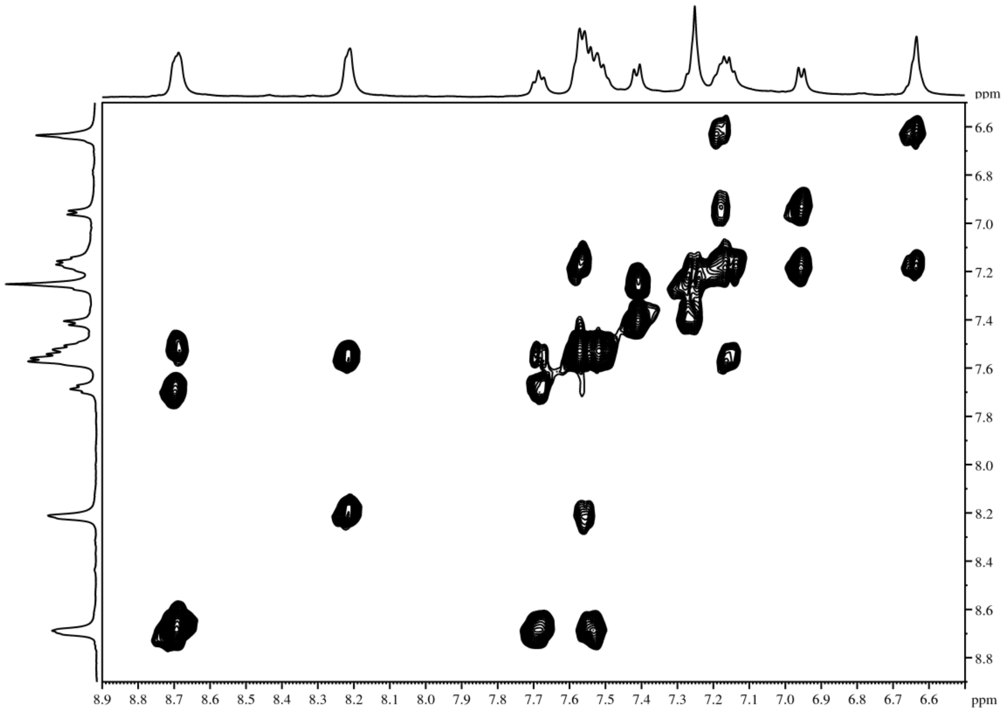

Figure S133. H,H-COSY NMR spectrum of **24a**, expansion

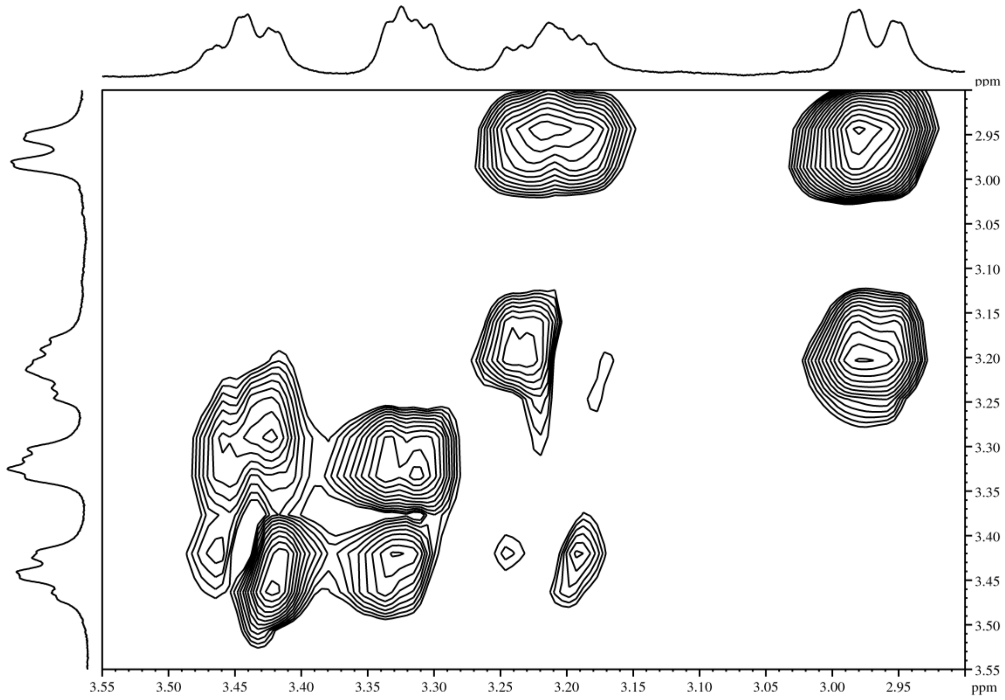

Figure S134. H,H-COSY NMR spectrum of **24a**, expansion

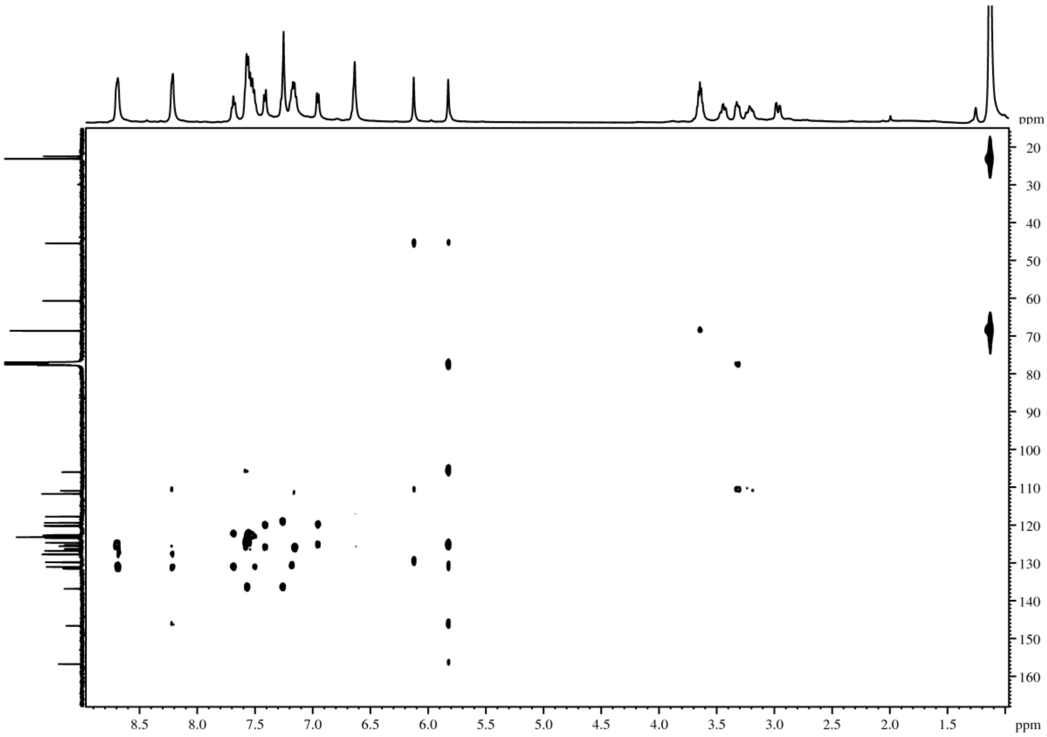

Figure S135. HMBC NMR spectrum of **24a**

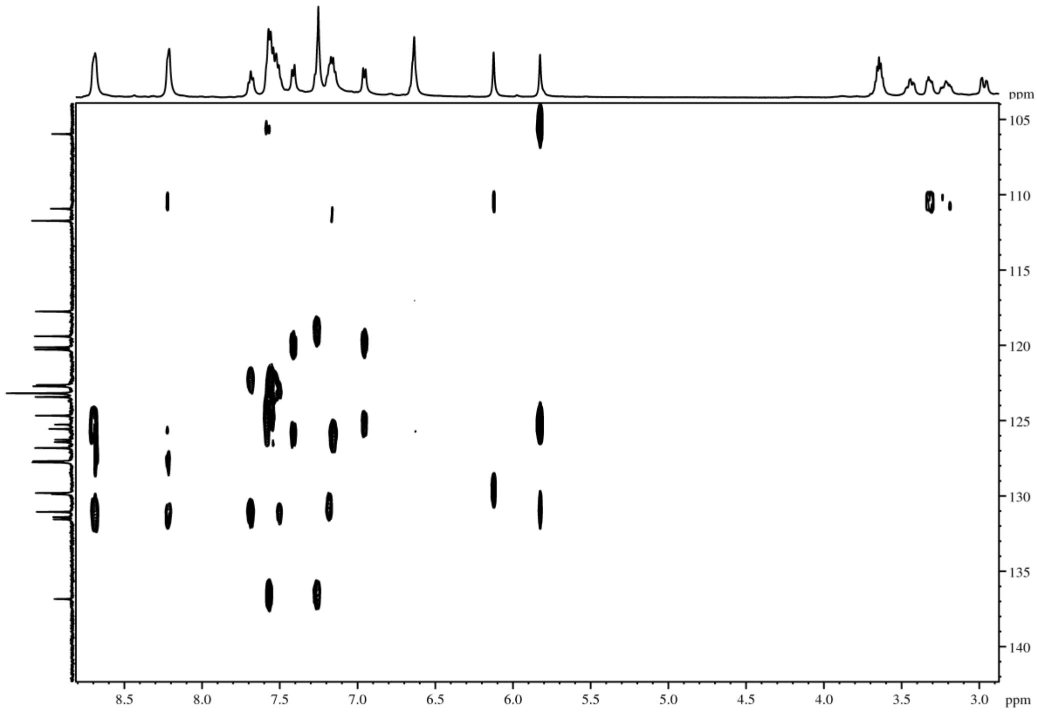

Figure S136. HMBC NMR spectrum of **24a**, expansion

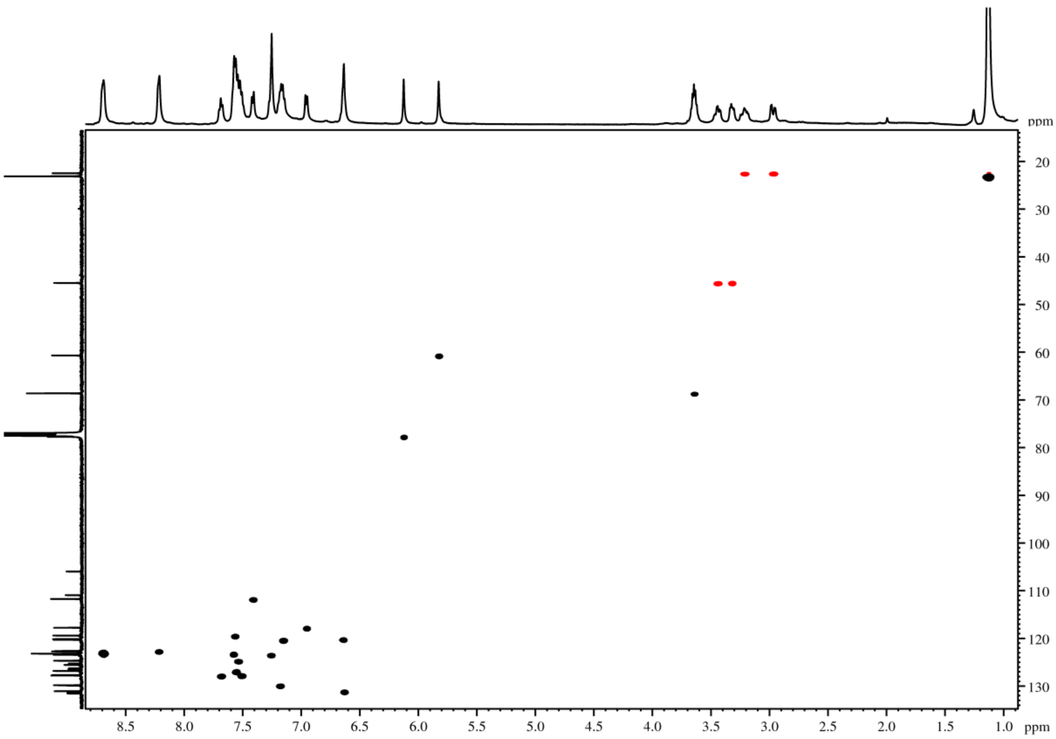

Figure S137. edited HSQC NMR spectrum of **24a** (black: positive, red: negative signals)

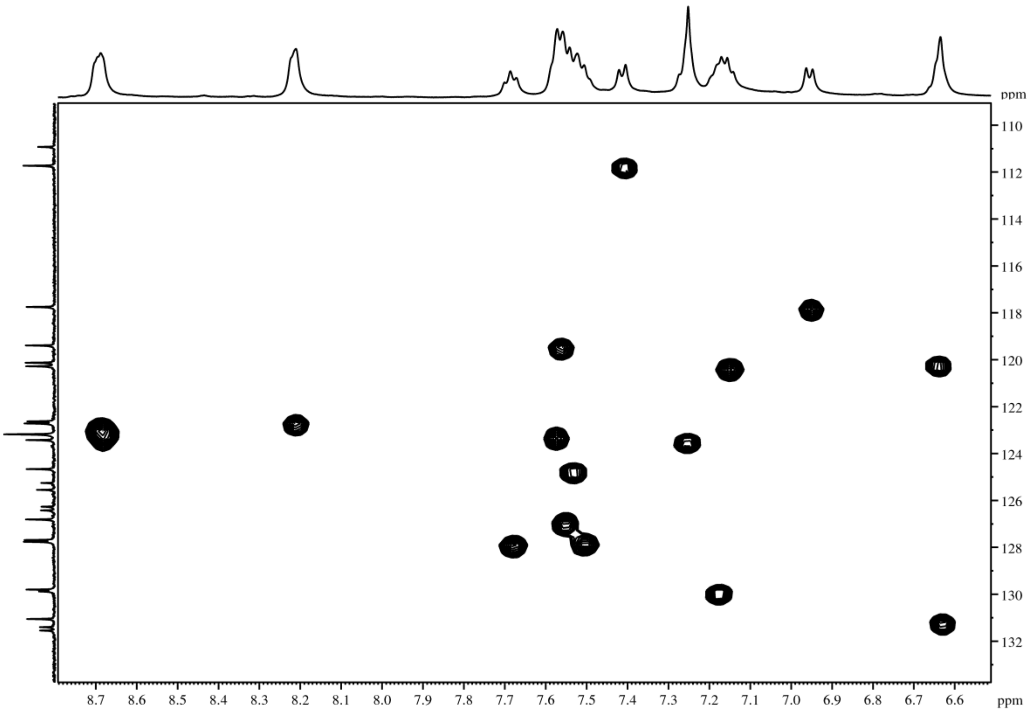

Figure S138. edited HSQC NMR spectrum of **24a**, expansion (black: positive, red: negative signals)

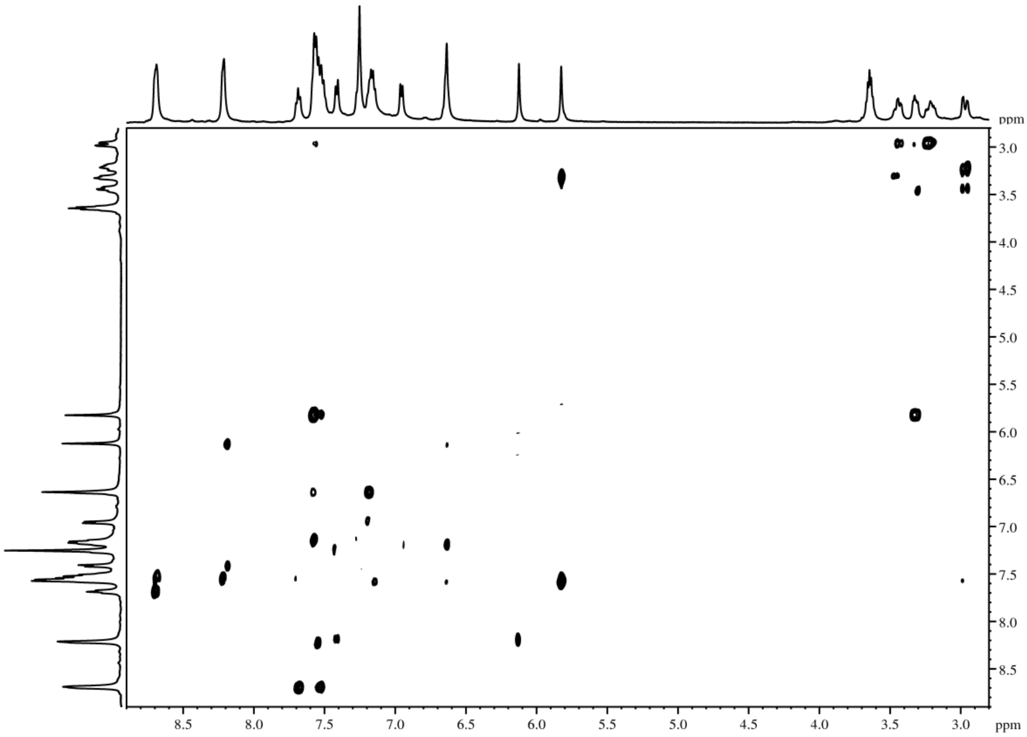

Figure S139. Phase-sensitive NOESY NMR spectrum of **24a** (only positive signals, mixing time 800 ms)

9aR*,17S*-15-(2-Hydroxyphenyl)-phenanthr[9,10-e]oxazino[2,3-a]isoquinoline (26)

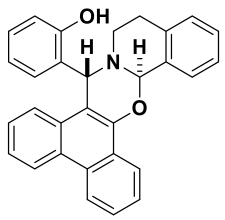

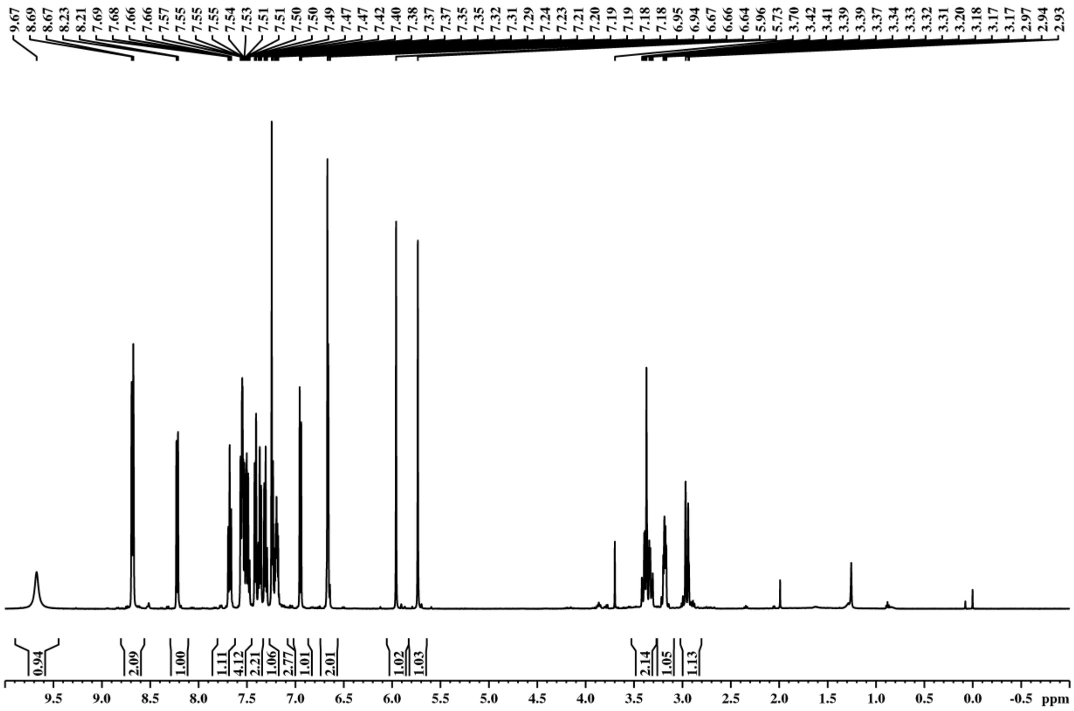

Figure S140. ^{1}H NMR spectrum of **26a**

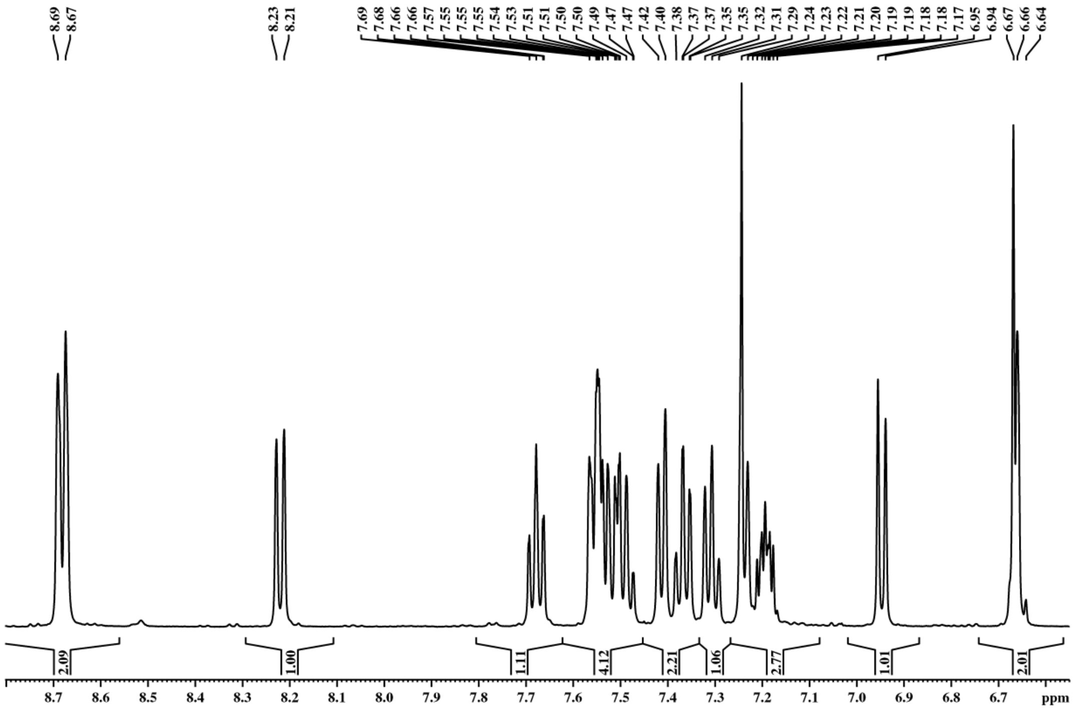

Figure S141. ^{1}H NMR spectrum of **26a**, expansion

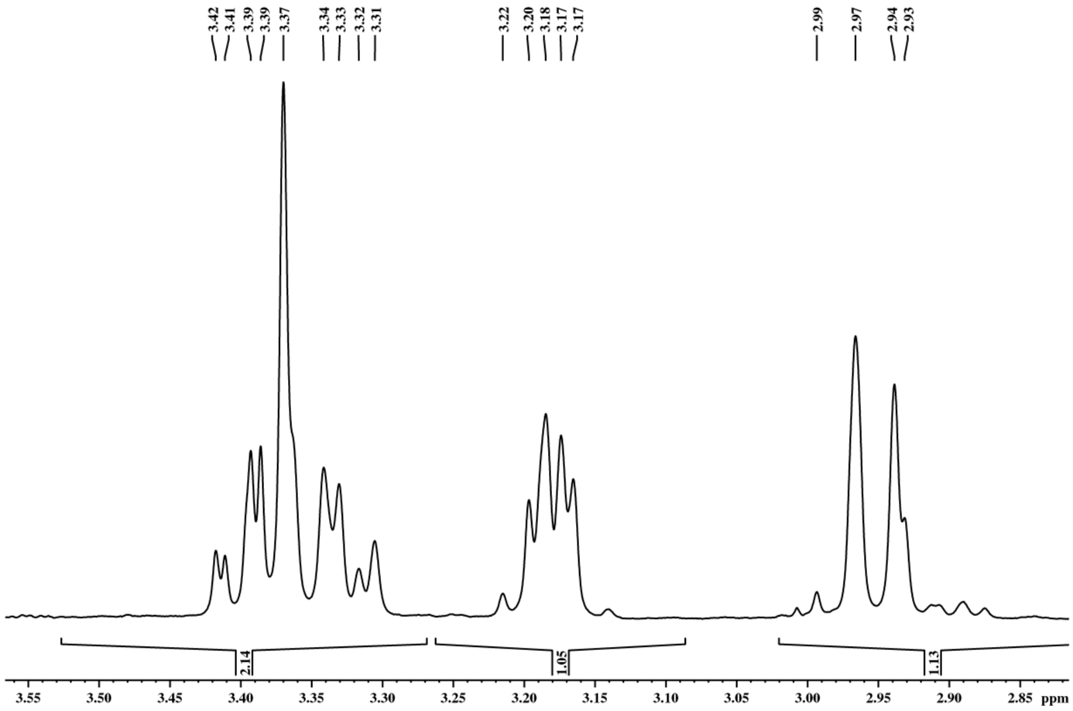

Figure S142. ^{1}H NMR spectrum of **26a**, expansion

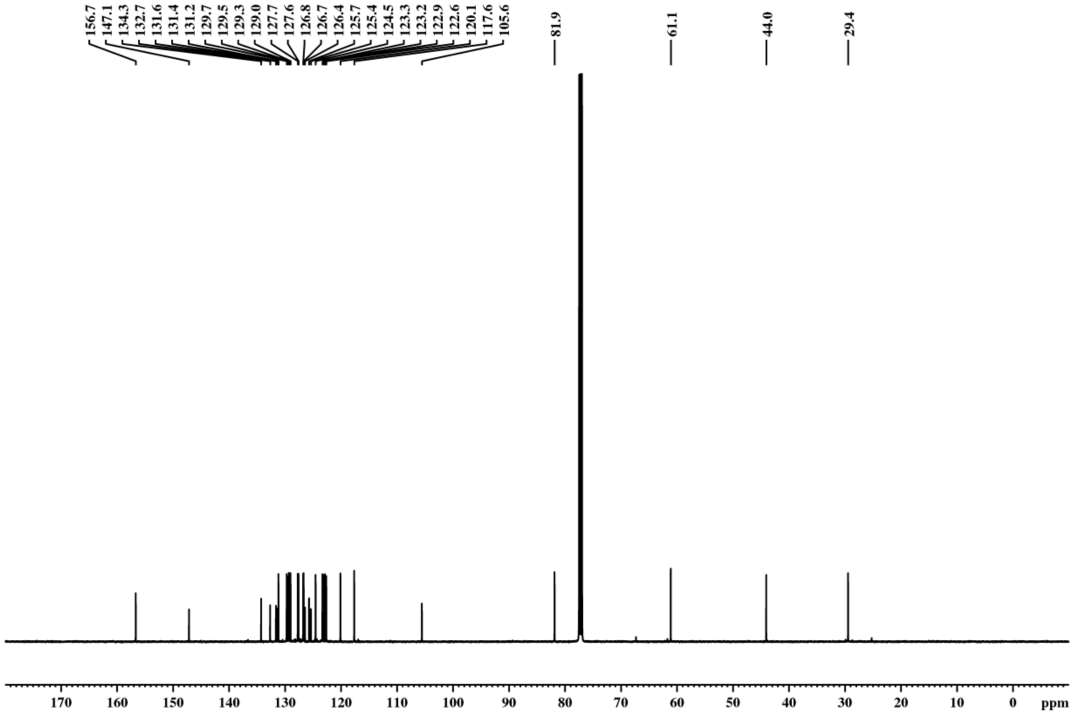

Figure S143. ^{13}C NMR spectrum of **26a**

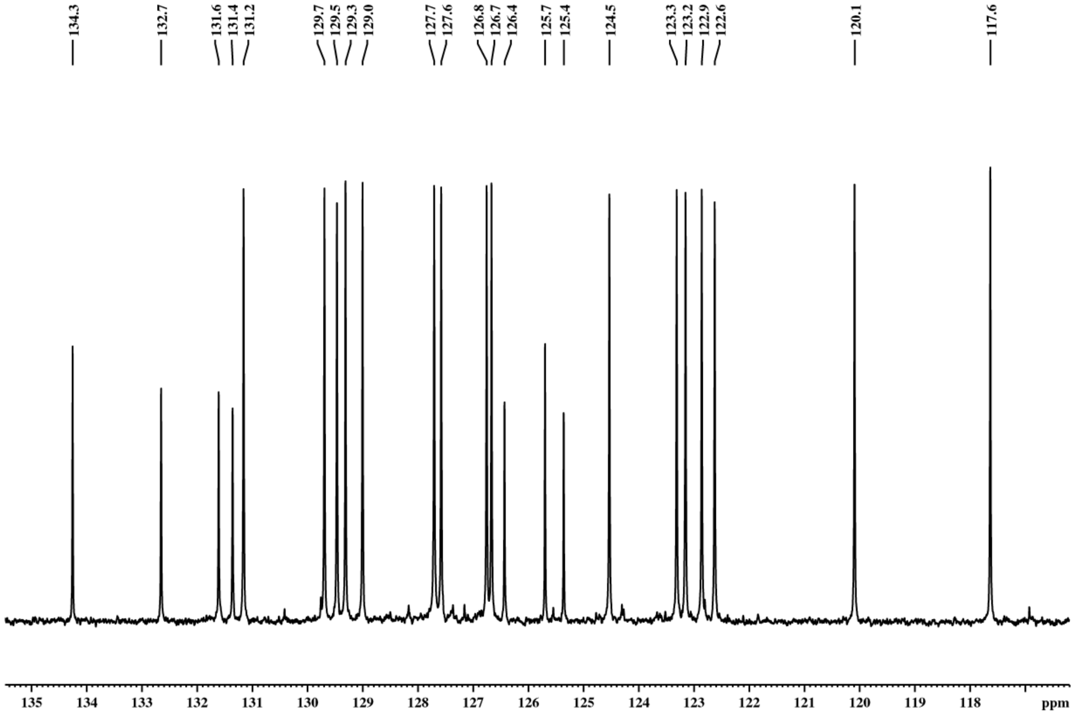

Figure S144. ^{13}C NMR spectrum of **26a**, expansion

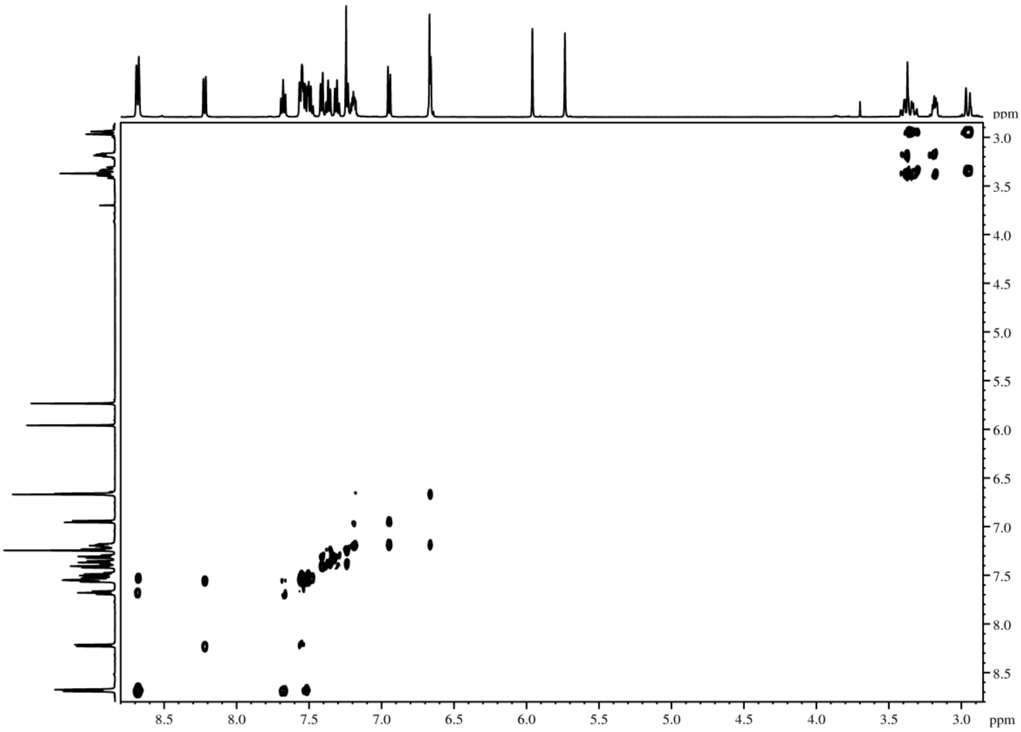

Figure S145. H,H-COSY NMR spectrum of **26a**

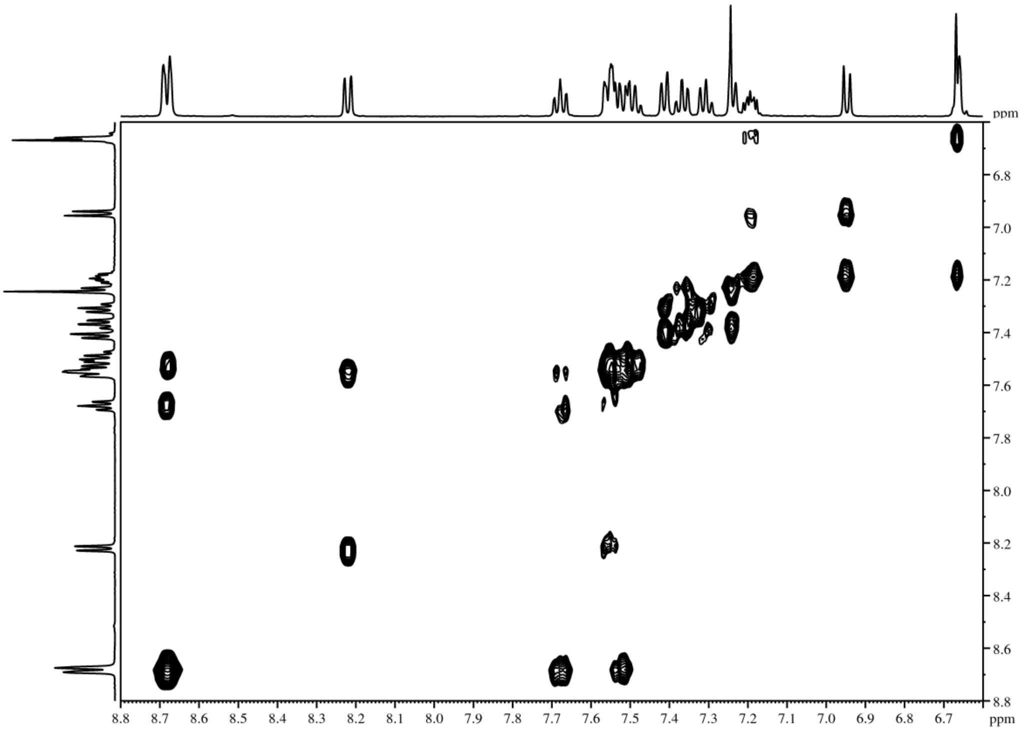

Figure S146. H,H-COSY NMR spectrum of **26a**, expansion

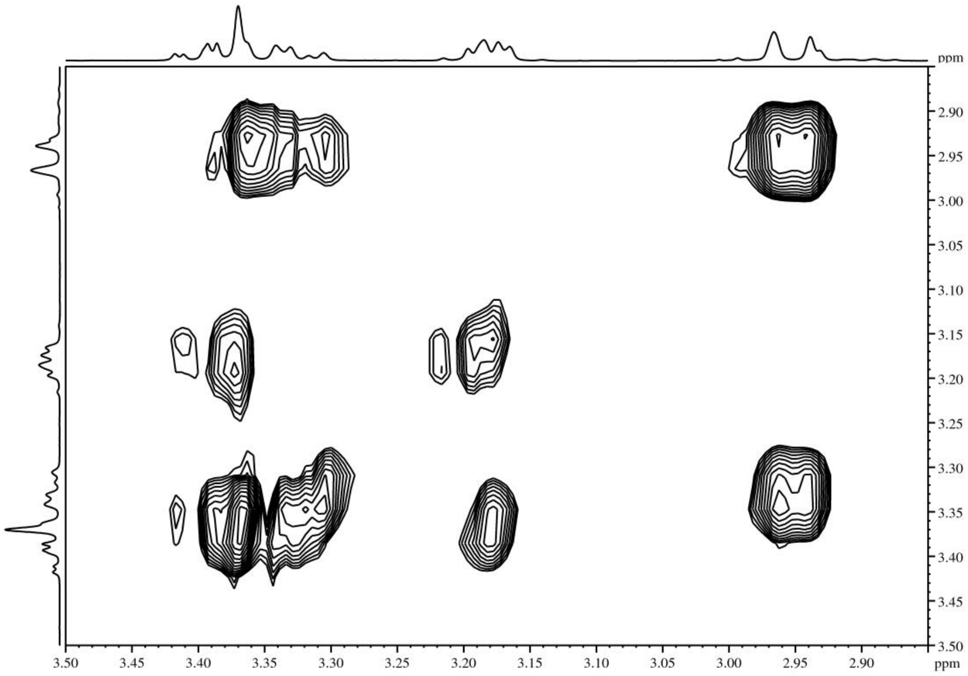

Figure S147. H,H-COSY NMR spectrum of **26a**, expansion

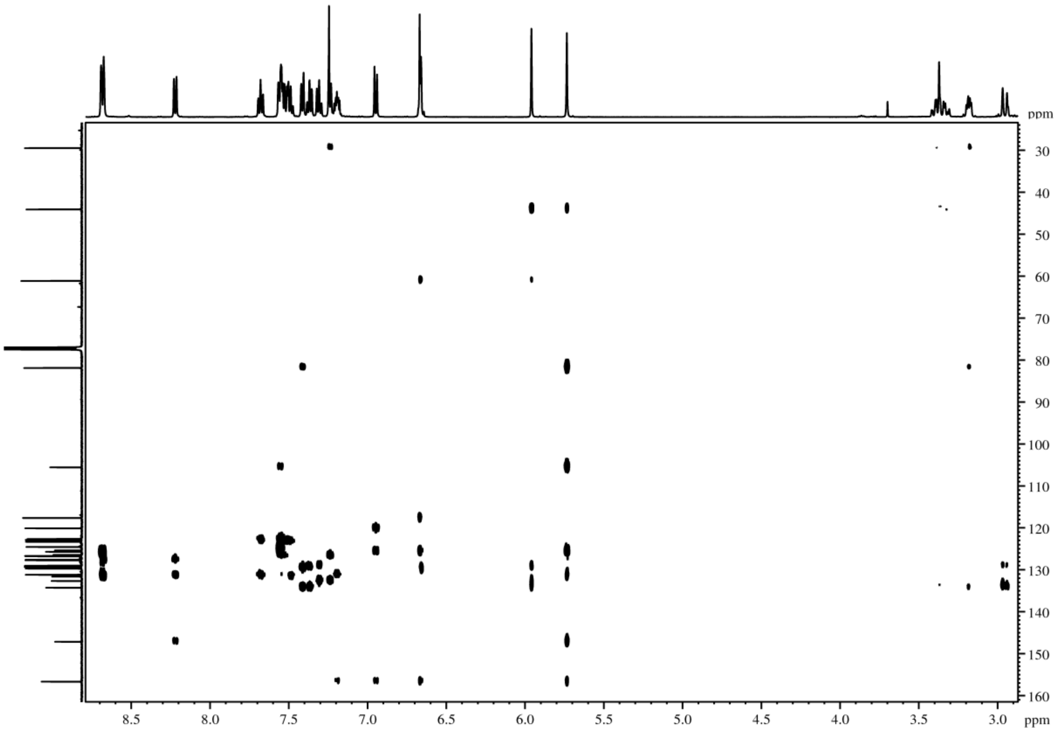

Figure S148. HMBC NMR spectrum of **26a**

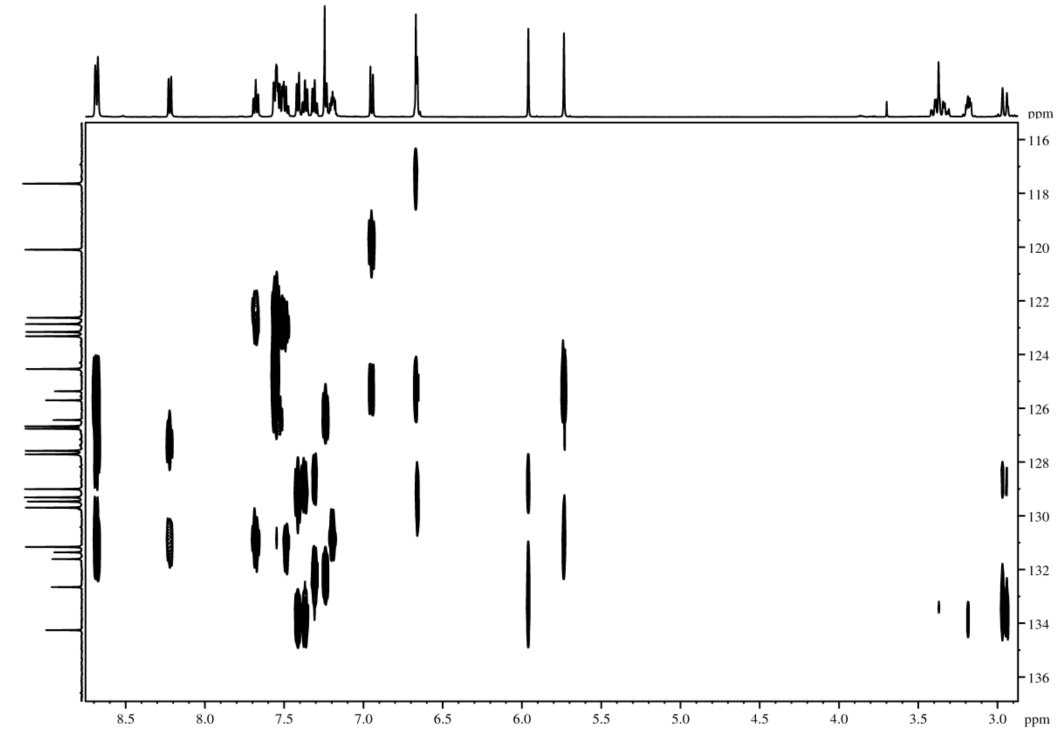

Figure S149. HMBC NMR spectrum of **26a**, expansion

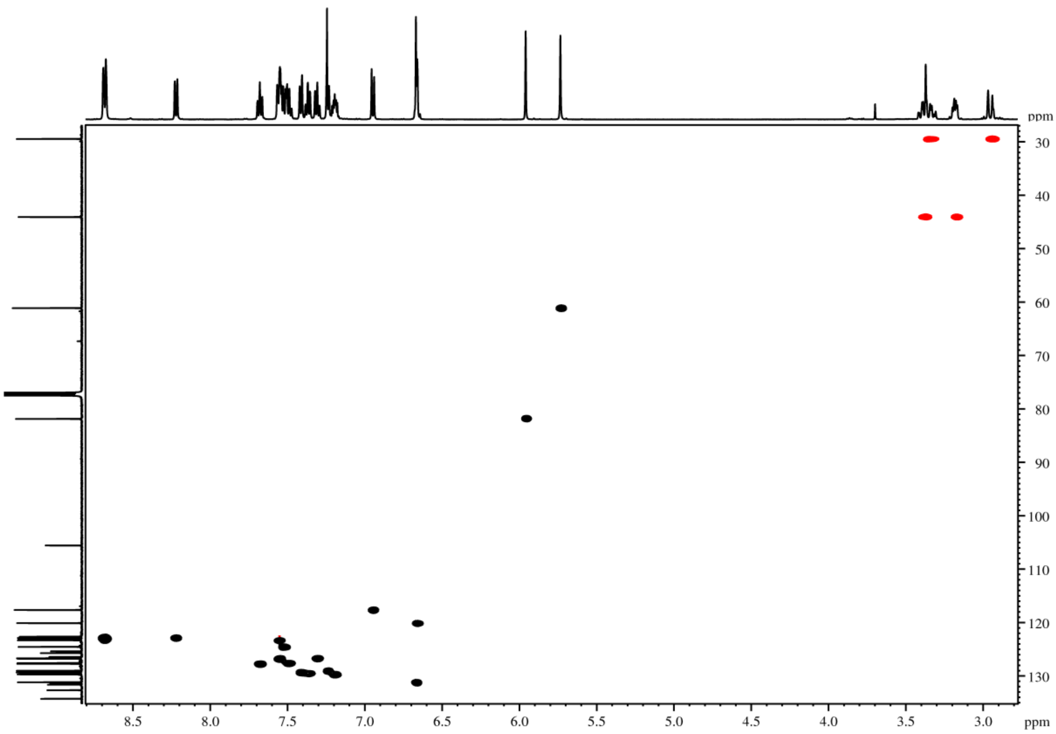

Figure S150. edited HSQC NMR spectrum of **26a** (black: positive, red: negative signals)

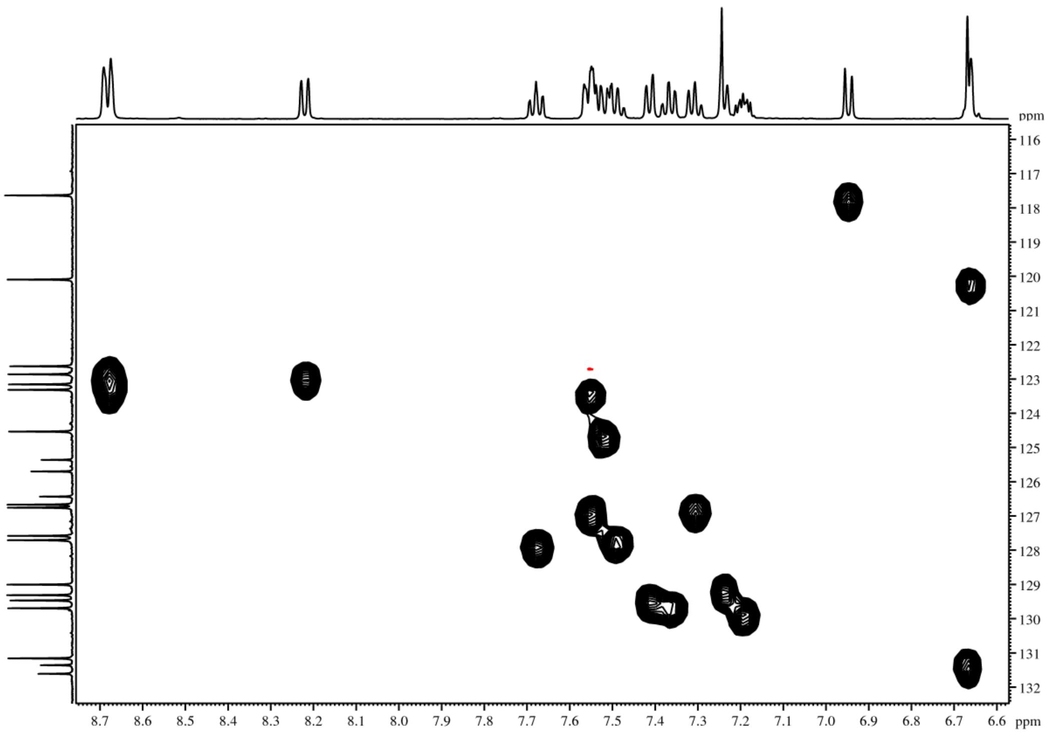

Figure S151. edited HSQC NMR spectrum of **26a**, expansion (black: positive, red: negative signals)

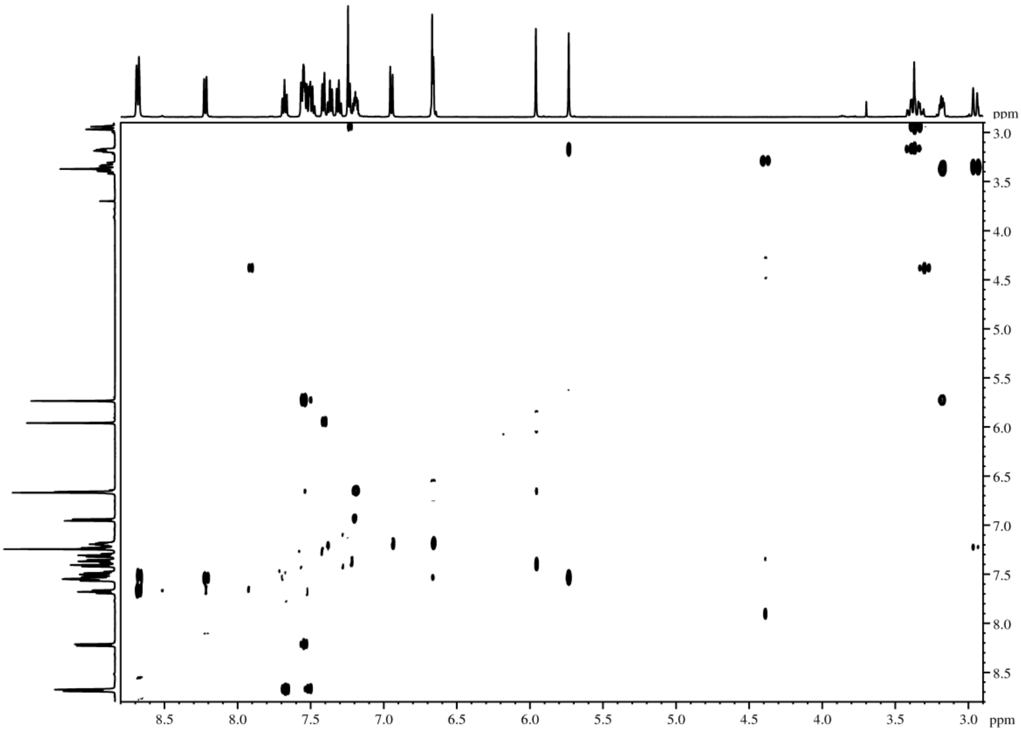

Figure S152. Phase-sensitive NOESY NMR spectrum of **26a** (only positive signals, mixing time 800 ms)

9aR*,16S*-16-(2-Hydroxyphenyl)-phenanthr[9,10-e]oxazino[2,3-a]thieno[3,2-c]pyridine (28)

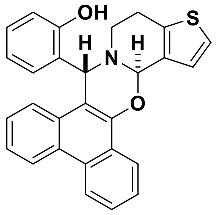

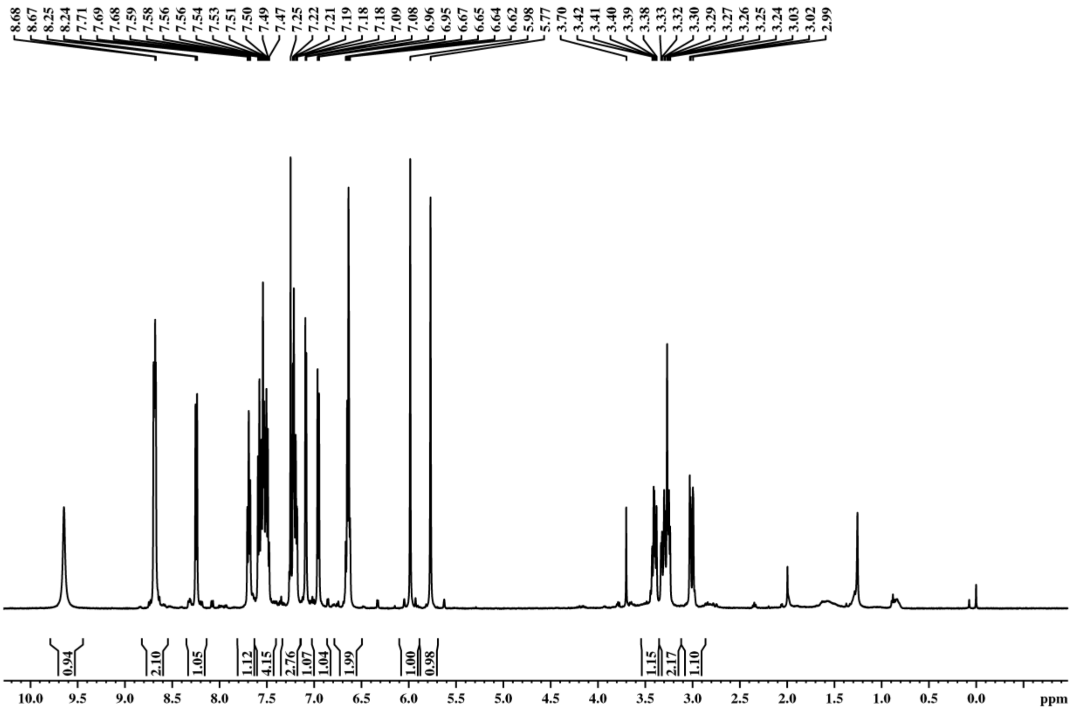

Figure S153. ^{1}H NMR spectrum of **28a**

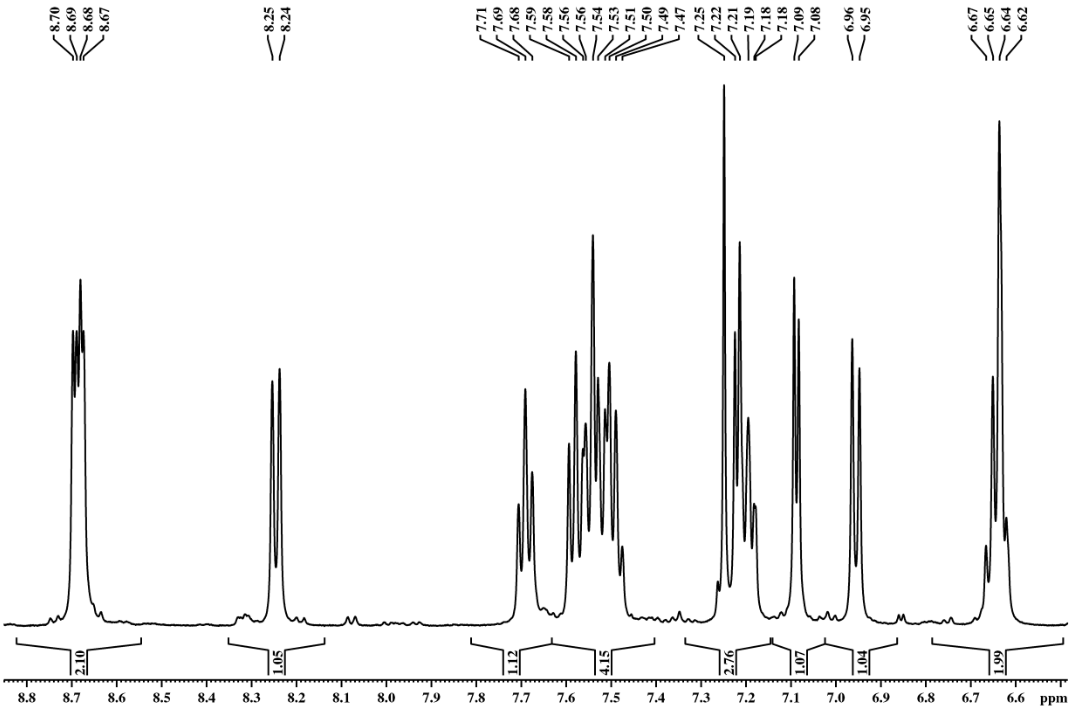

Figure S154. ^{1}H NMR spectrum of **28a**, expansion

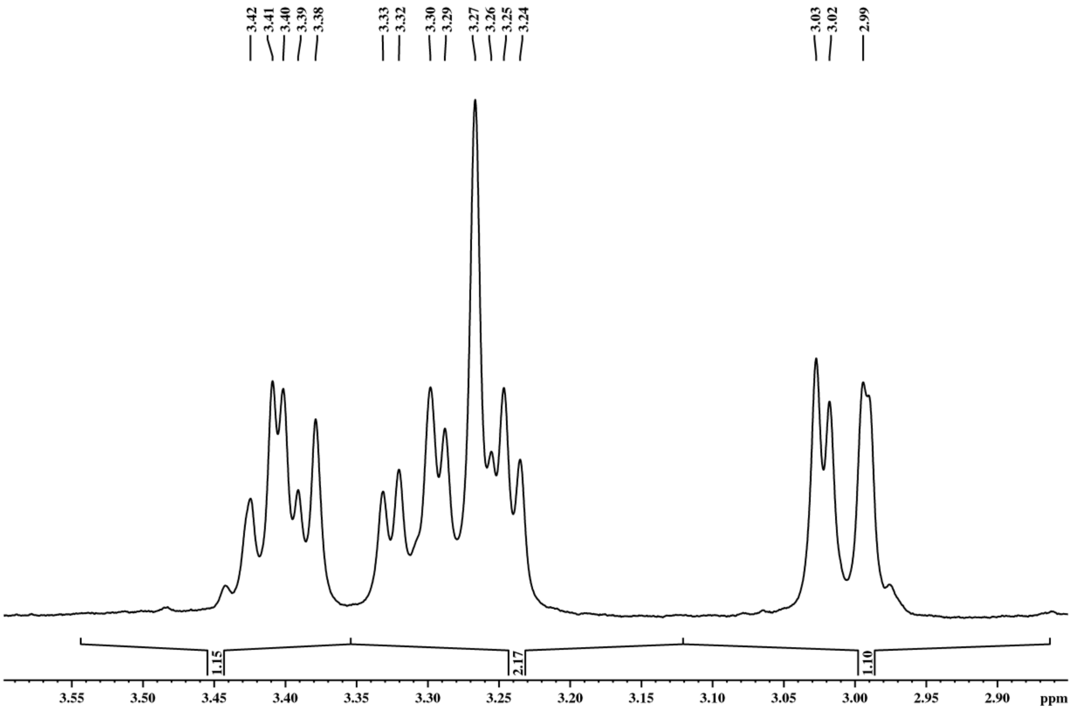

Figure S155. ^{1}H NMR spectrum of **28a**, expansion

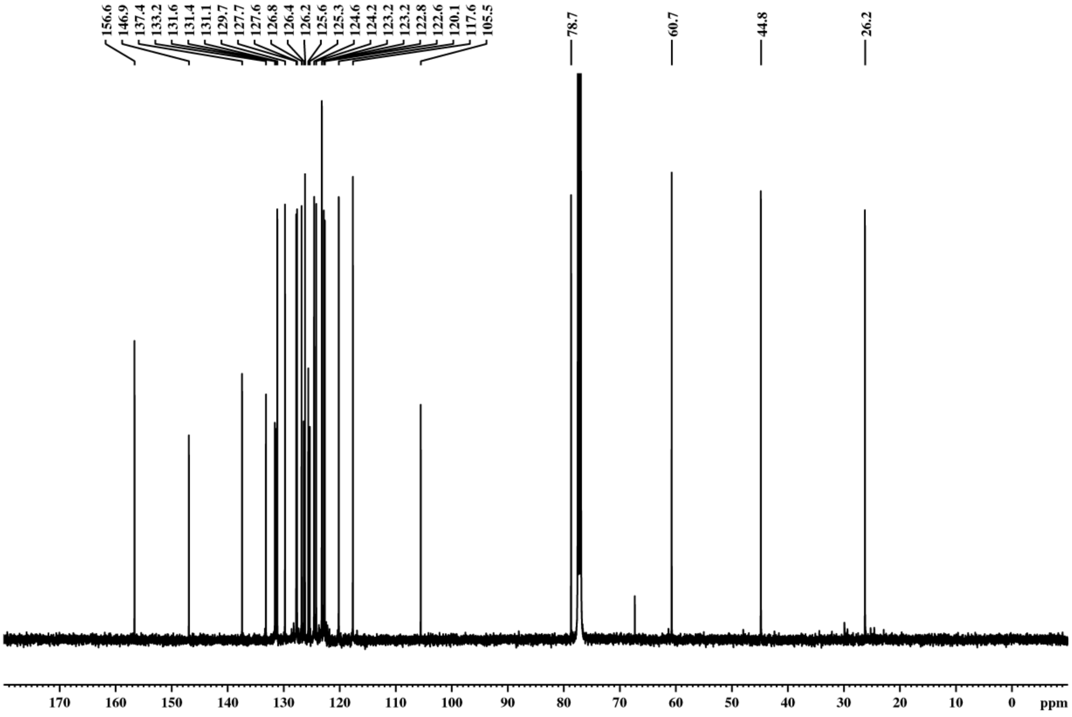

Figure S156. ^{13}C NMR spectrum of **28a**

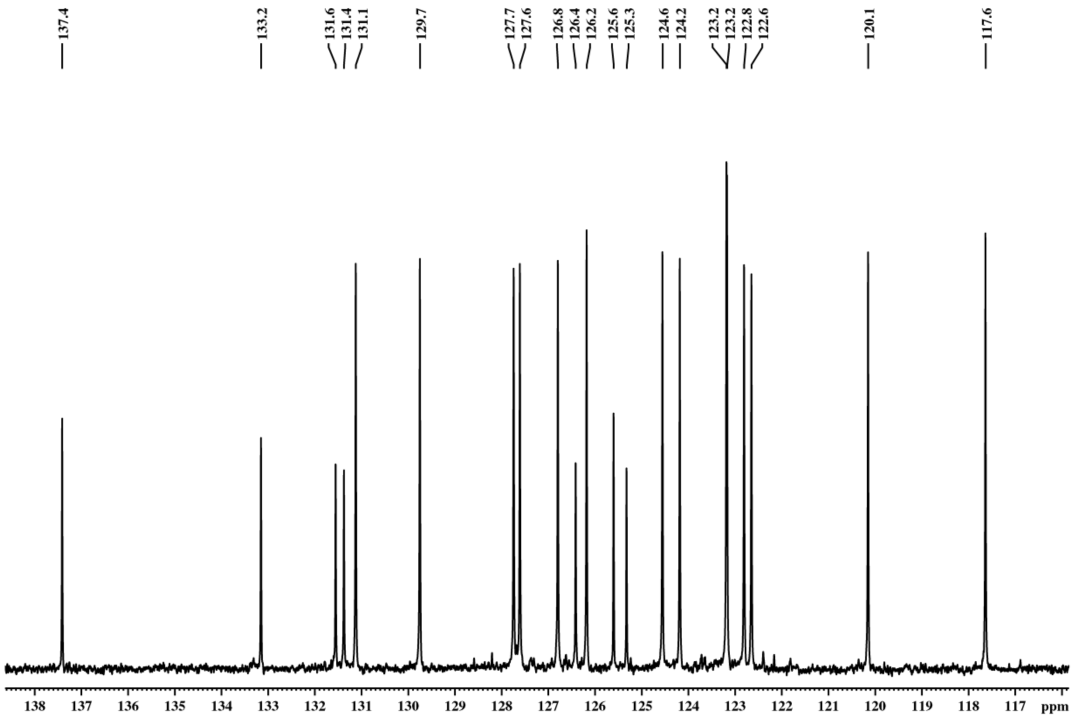

Figure S157. ^{13}C NMR spectrum of **28a**, expansion

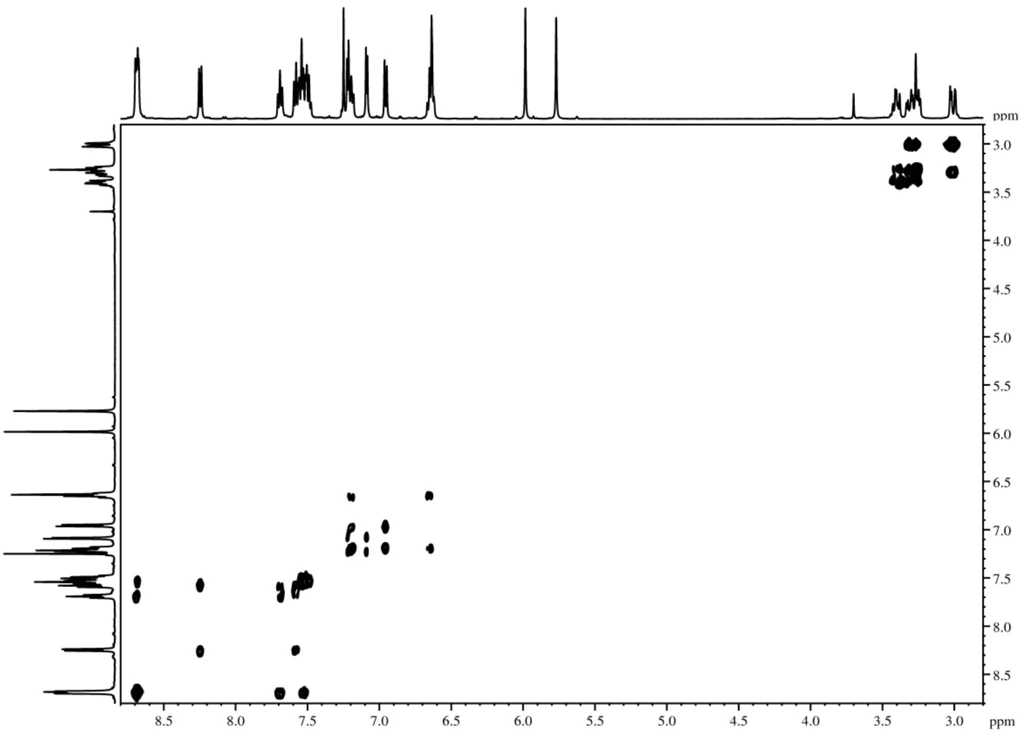

Figure S158. H,H-COSY NMR spectrum of **28a**

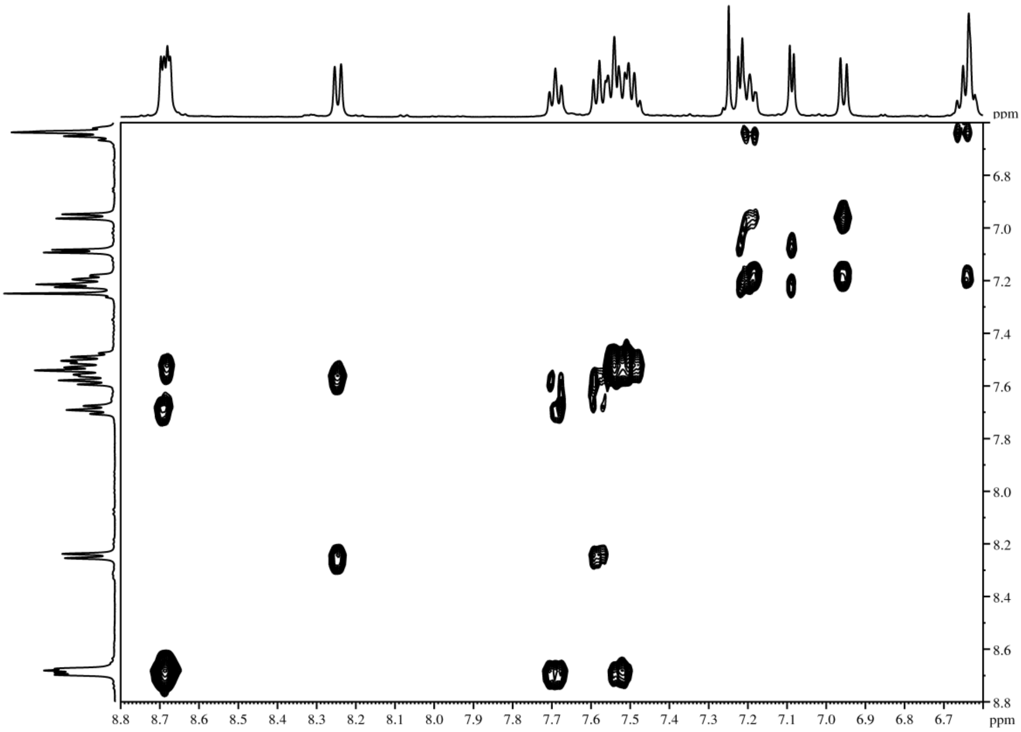

Figure S159. H,H-COSY NMR spectrum of **28a**, expansion

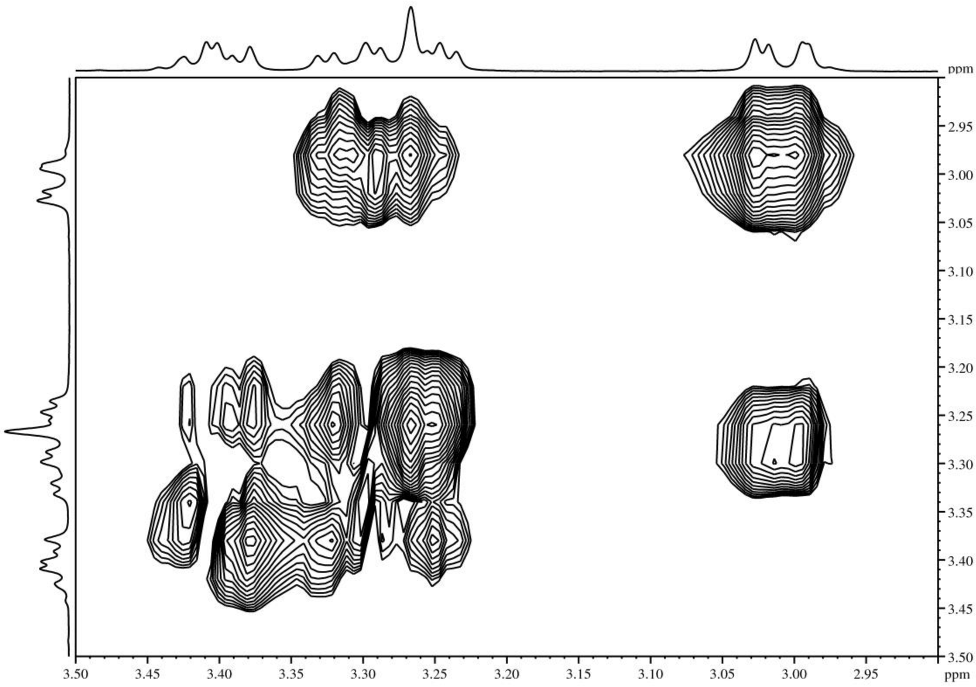

Figure S160. H,H-COSY NMR spectrum of **28a**, expansion

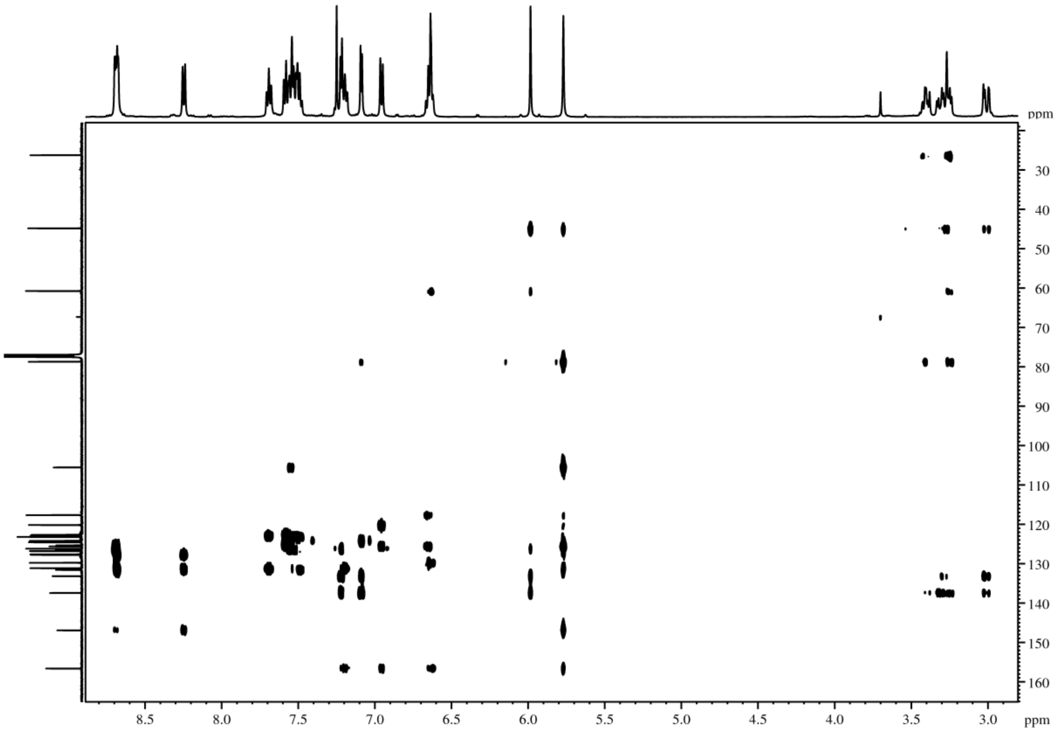

Figure S161. HMBC NMR spectrum of **28a**

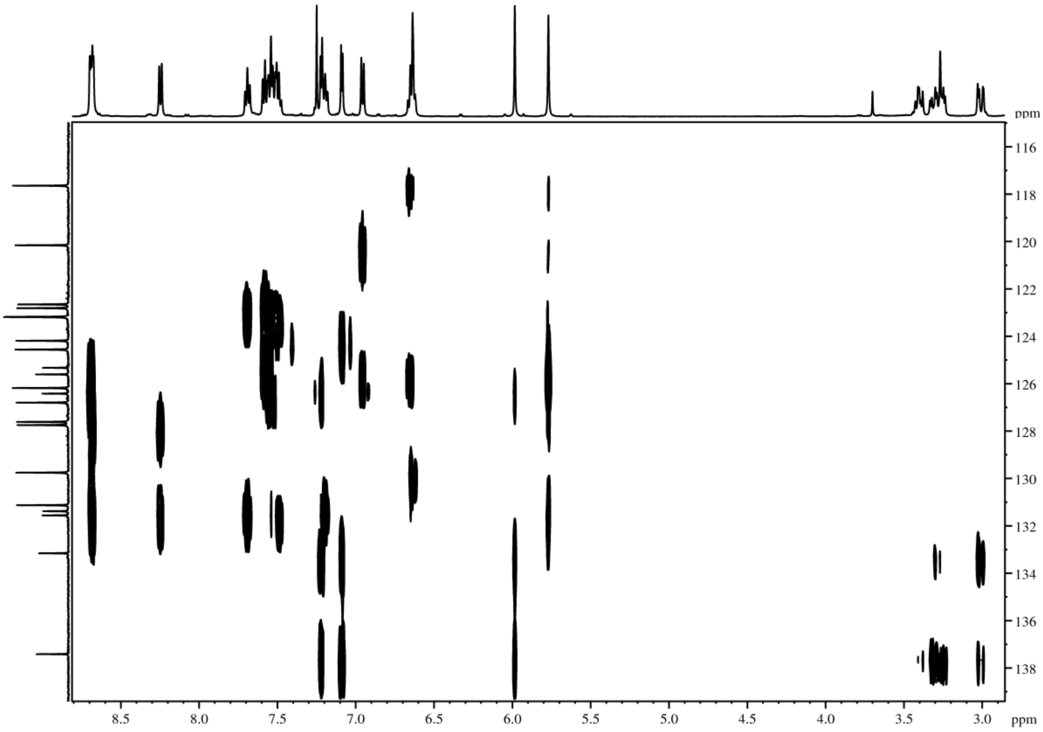

Figure S162. HMBC NMR spectrum of **28a**, expansion

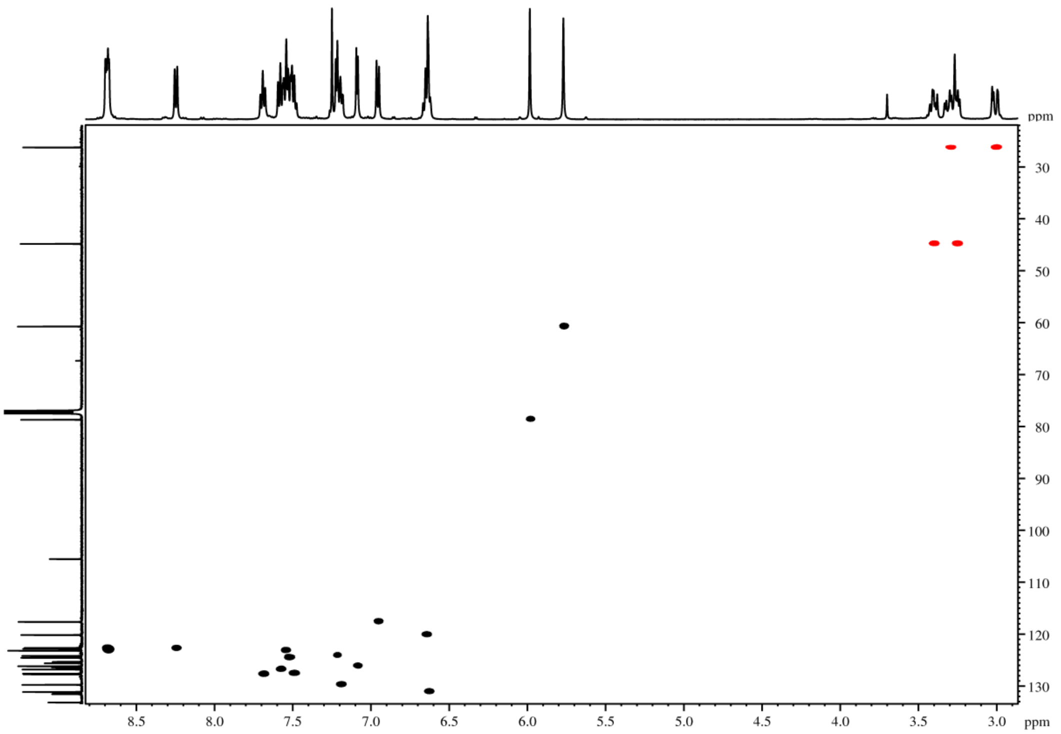

Figure S163. edited HSQC NMR spectrum of **28a** (black: positive, red: negative signals)

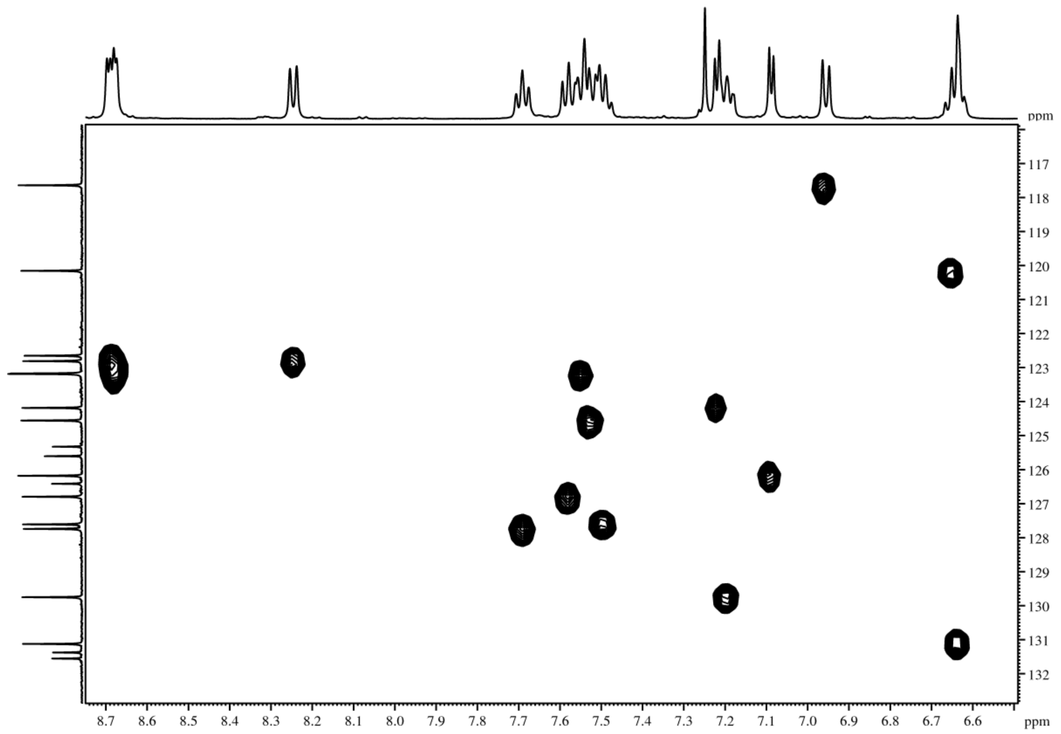

Figure S164. edited HSQC NMR spectrum of **28a**, expansion (black: positive, red: negative signals)

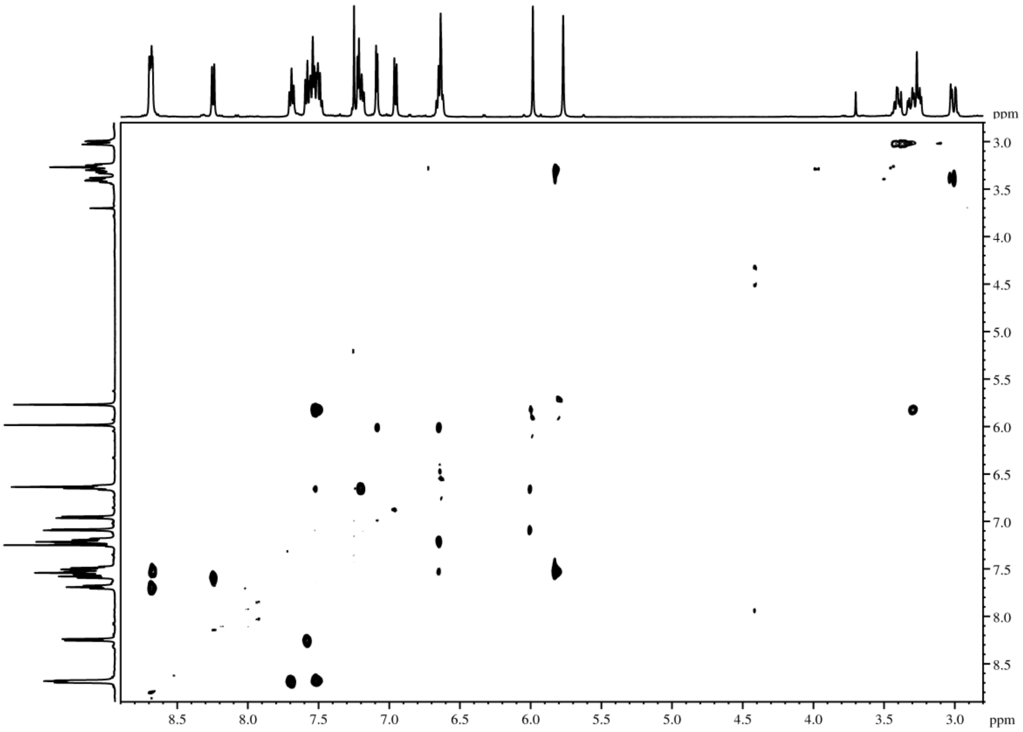

Figure S165. Phase-sensitive NOESY NMR spectrum of **28a** (only positive signals, mixing time 800 ms)
